# Supplementary material for: The invasive red-eared slider turtle is more successful than the native Chinese three-keeled pond turtle: evidence from the gut microbiota
Source: PeerJ. 2020 Oct 29;8:e10271. doi: 10.7717/peerj.10271 (PMC7603792; doi:10.7717/peerj.10271)
Supplement: Supplemental Information 1 [file peerj-08-10271-s001.pdf]

Table S1 The number of operational taxonomic units (OTUs) and different bacterial taxonomic units of each sample for *Chinemys reevesii* (CR) and *Trachemys scripta elegans* (TSE).

Samples	OTUs	Phylum	Class	Order	Family	Genus
CR_1	32	3	4	4	9	8
CR_2	30	4	5	5	8	8
CR_3	25	3	4	5	9	8
TSE_1	29	3	4	4	8	8
TSE_2	30	4	5	6	10	9
TSE_3	40	4	5	5	9	10
Total	50	5	7	7	17	28
