# Supplementary material for: The invasive red-eared slider turtle is more successful than the native Chinese three-keeled pond turtle: evidence from the gut microbiota
Source: PeerJ. 2020 Oct 29;8:e10271. doi: 10.7717/peerj.10271 (PMC7603792; doi:10.7717/peerj.10271)
Supplement: Supplemental Information 2 [file peerj-08-10271-s002.pdf]

Table S2 A summary of diversity indexes and results of Student's t tests.

Diversity indexes	*Chinemys reevesii*	*Trachemys scripta elegans*	Student's t test
Ace	46.5862±1.8417	54.9056±3.3461	t = 2.11, df = 4, P = 0.10
Shannon	2.3211±0.1785	2.3661±0.0251	t = 0.25, df= 2.08, P = 0.83
Shannoneven	0.6091±0.0486	0.6093±0.0086	t < 0.01, df = 4, P > 0.09
Good's Coverage	0.9996±0.0001	0.9989±0.0004	t = 1.67, df = 4, P = 0.17
