# Supplementary material for: The invasive red-eared slider turtle is more successful than the native Chinese three-keeled pond turtle: evidence from the gut microbiota
Source: PeerJ. 2020 Oct 29;8:e10271. doi: 10.7717/peerj.10271 (PMC7603792; doi:10.7717/peerj.10271)
Supplement: Supplemental Information 3 [file peerj-08-10271-s003.pdf]

Table S3 Results of linear discriminatory analysis (LDA) on the relative abundance between *Chinemys reevesii* (CR) and *Trachemys scripta elegans* (TSE).

Taxon	Group	LDA value	*P* value
Firmicutes.Clostridia.Clostridiales.Lachnospiraceae	CR	5.010648	0.049535
Bacteroidetes.Bacteroidia.Bacteroidales.Porphyromonadaceae	TSE	5.205142	0.049535
Bacteroidetes	TSE	5.154239	0.049535
Firmicutes	CR	5.148316	0.049535
Firmicutes.Clostridia	CR	5.110004	0.049535
Firmicutes.Clostridia.Clostridiales	CR	5.10834	0.049535
Bacteroidetes.Bacteroidia.Bacteroidales	TSE	5.105383	0.049535
Bacteroidetes.Bacteroidia	TSE	5.058165	0.049535
Bacteroidetes.Bacteroidia.Bacteroidales.Bacteroidaceae	CR	5.044687	0.049535
Fusobacteria.Fusobacteriia.Fusobacteriales	TSE	4.799418	0.046302
Fusobacteria.Fusobacteriia	TSE	4.778695	0.046302
Fusobacteria.Fusobacteriia.Fusobacteriales.Fusobacteriaceae	TSE	4.764831	0.046302
Fusobacteria	TSE	4.750134	0.046302
Firmicutes.Clostridia.Clostridiales.Clostridiaceae	CR	4.455754	0.049535
