# Supplementary figures and images for: The invasive red-eared slider turtle is more successful than the native Chinese three-keeled pond turtle: evidence from the gut microbiota
Source: PeerJ. 2020 Oct 29;8:e10271. doi: 10.7717/peerj.10271 (PMC7603792; doi:10.7717/peerj.10271)

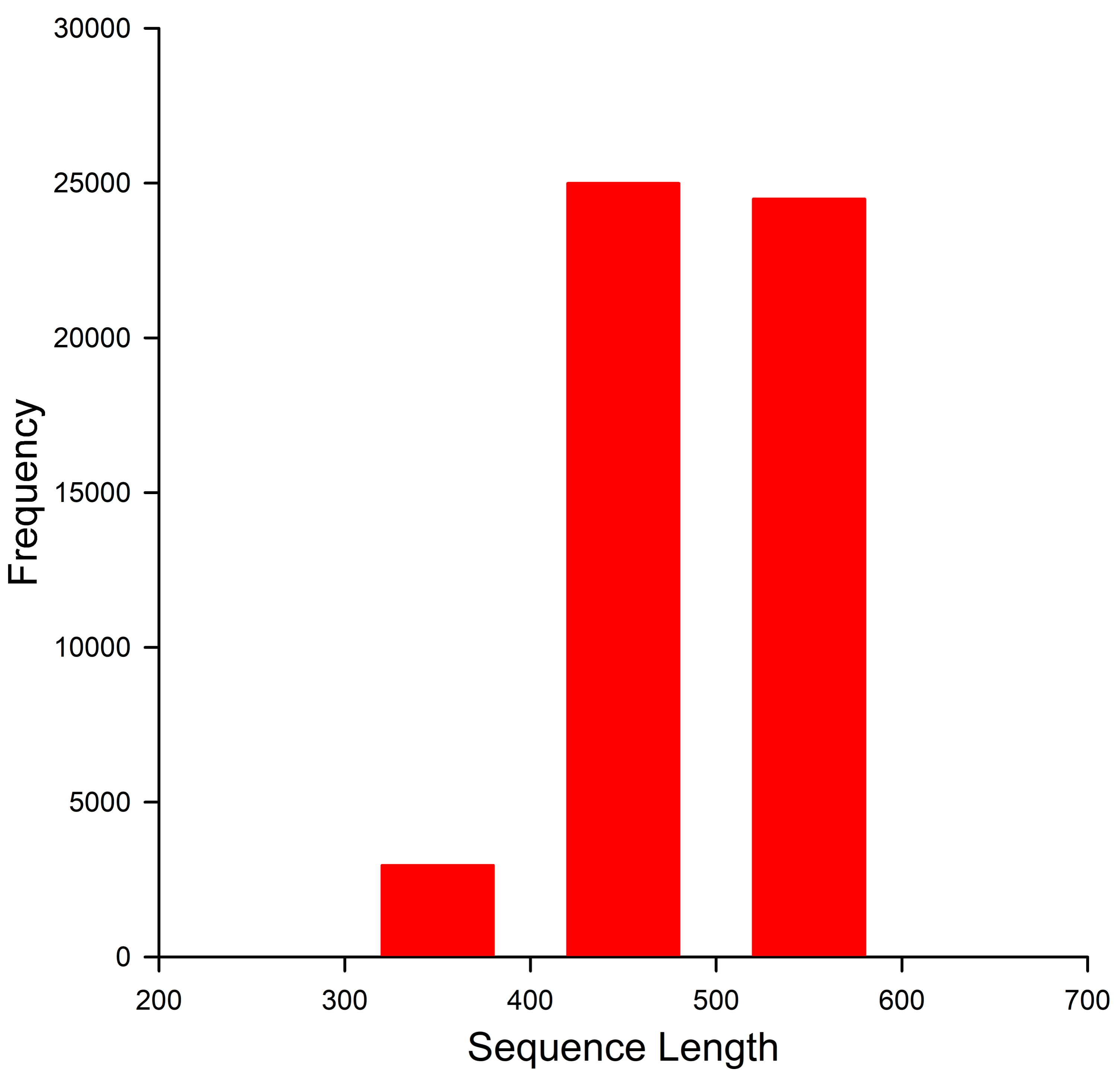

Supplement: Supplemental Information 4 [file peerj-08-10271-s004.pdf]

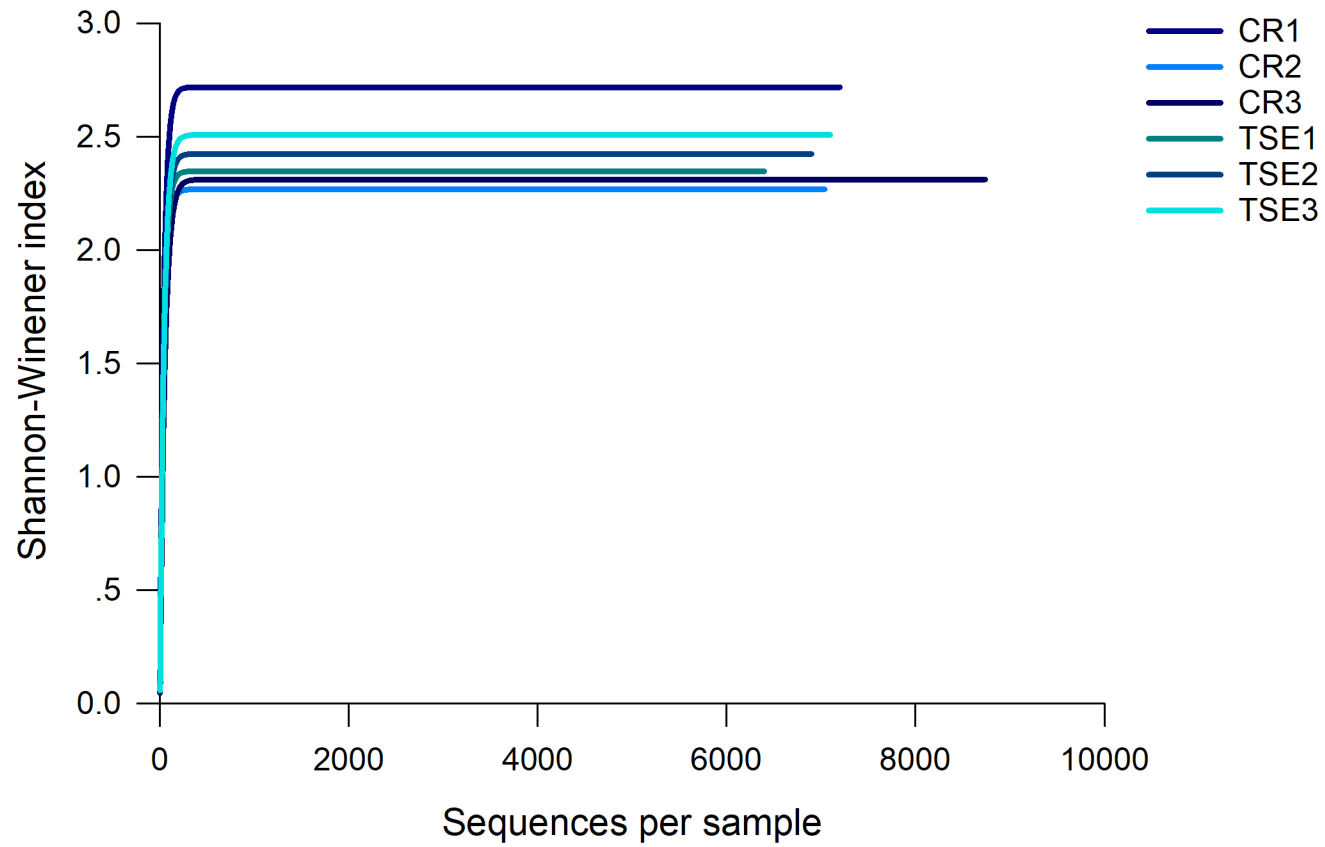

Supplement: Supplemental Information 5 — Each color line represents a sample. [file peerj-08-10271-s005.pdf]

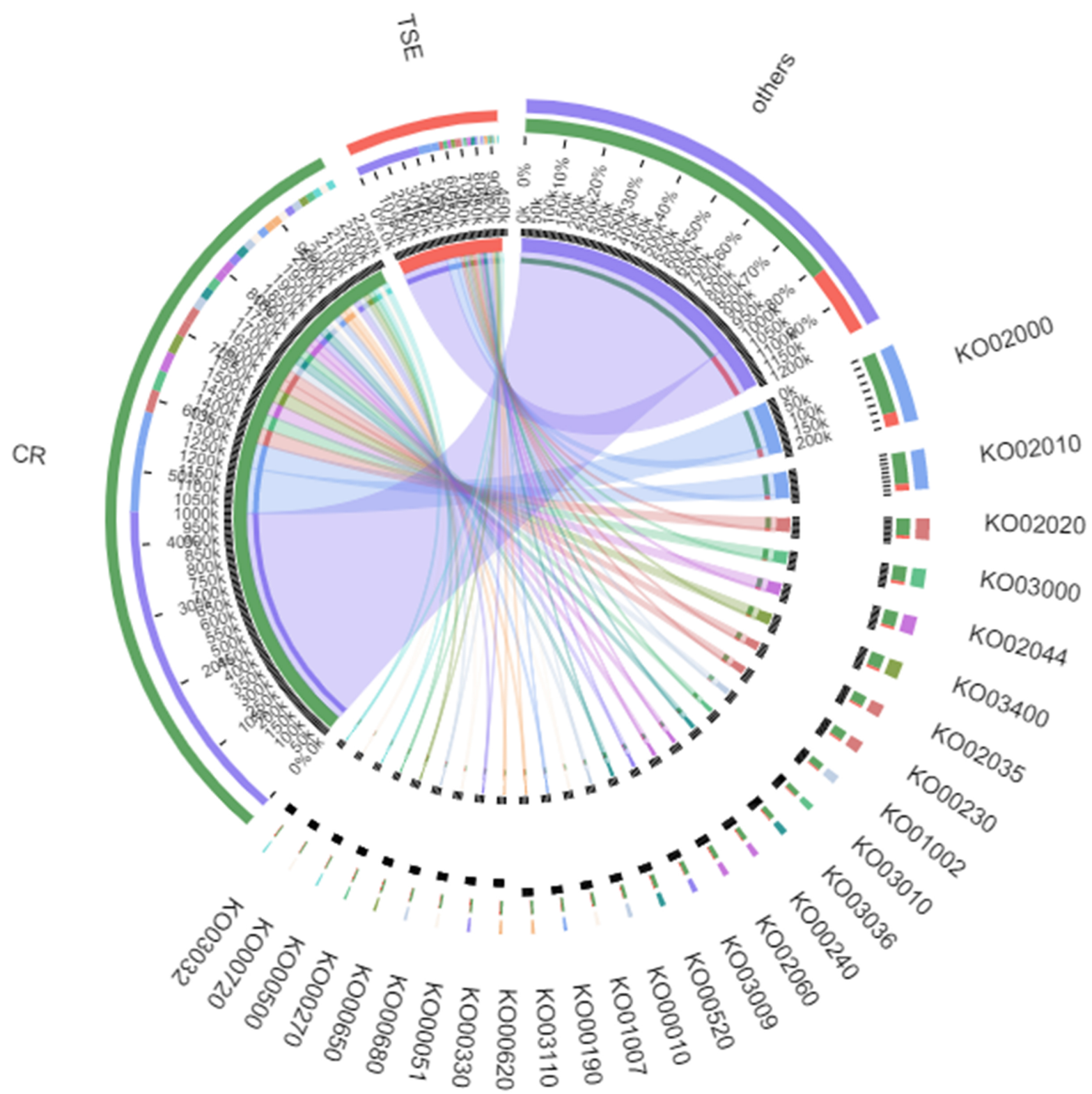

Supplement: Supplemental Information 6 — Different colors indicate the higher proportion KOs of the gut microbiota in the correspond species. [file peerj-08-10271-s006.pdf]
