# Supplementary material for: The invasive red-eared slider turtle is more successful than the native Chinese three-keeled pond turtle: evidence from the gut microbiota
Source: PeerJ. 2020 Oct 29;8:e10271. doi: 10.7717/peerj.10271 (PMC7603792; doi:10.7717/peerj.10271)
Supplement: Supplemental Information 7 — Each sample is started with a “¿”. The file includes six samples and they are CR1, CR2, CR3, TSE1, TSE2 and TSE3. [file peerj-08-10271-s007.docx]

All sequencing is contained in this file. Each sample is started with a “>”. The file includes six samples and they are CR1, CR2, CR3, TSE1, TSE2 and TSE3.

>CR1

@D0950.29_1000

ATTGAACGCTGGCGGCAGGCCTAACACATGCAAGTCGAACGGTAGCACAGAGAGCTTGCTCTCGGGTGACGAGTGGCGGACGGGTGAGTAATGTCTGGGAAACTGCCCGATGGAGGGGGATAACTACTGGAAACGGTAGCTAATACCGCATAATGTCGCAAGACCAAAGAGGGGGACCTTCGGGCCTCTTGCCAATCGGATGTGCCCAGATGGGATTAGCTAGTAGGTGGGGTAACGGCTCACCTAGGCGACGATCCCTAGCTGGTCTGAGAGGATGACCAGCCACACTGGAACTGAGACACGGTCCAGACTCCTACGGGAGGCAGCAGTGGGGAATATTGCACAATGGGCGCAAGCCTGATGCAGCCATGCCGCGTGTATGAAGAAGGCCTCGGGTTGTAAAGTACTTCAGCGAGAGGAAGGCGGTGAGGTTAATAACCTCATCGATTGACGTTACTCGCAGAAGAAGCA

+

:::::::::::::::::::::::::::::::::::::::::::::::::::::::::::::::::::::::::::::::::::::::::::::::::::::::::::::::::::::::::::::::::::::::::::::::::::::::::::::::::::::::::::::::::::::::::::::::::::::::::::::::::::::::::::::::::::::::::::::::::::::::::::::::::::::::::::::::::::::::::::::::::::::::::::::::::::::::::::::::::::::::::::::::::::::::::::::::::::::::::::::::::::::::::::::::::::::::::::::::::::::::::::::::::::::::::::::::::::::::::::::::::::::::::::::::::::::::

@D0950.29_100125

GATGAACGCTGGCGGCGTGCTTAACACATGCAAGTCGAGCGAGGAATCACCTTCGGGTGTGAACTAGCGGCGGACGGGTGAGTAACACGTGGGCAACCTGCCTTACAGAGGGGGATAGCCTTCCGAAAGGAAGATTAATACCGCATATTATGAGTTTTCTGCATGGGGAATTCATGAAAGGAGAAATCCGCTGTAAGATGGGCCCGCGGCGCATTAGCTAGTTGGTGAGGTAACGGCTCACCAAGGCGACGATGCGTAGCCGACCTGAGAGGGTGATCGGCCACATTGGGACTGAGACACGGCCCAGACTCCTACGGGAGGCAGCAGTGGGGAATATTGCACAATGGGGGAAACCCTGATGCAGCAACGCCGCGTGAGTGATGAAGGCCTTCGGGTTGTAAAGCTCTGTCTTCAGGGACGATAATGACGGTACCTGAGGAGGAAGCCACGGCTAACTACGTGCCAGCAGCCGCGGTAA

+

::::::::::::::::::::::::::::::::::::::::::::::::::::::::::::::::::::::::::::::::::::::::::::::::::::::::::::::::::::::::::::::::::::::::::::::::::::::::::::::::::::::::::::::::::::::::::::::::::::::::::::::::::::::::::::::::::::::::::::::::::::::::::::::::::::::::::::::::::::::::::::::::::::::::::::::::::::::::::::::::::::::::::::::::::::::::::::::::::::::::::::::::::::::::::::::::::::::::::::::::::::::::::::::::::::::::::::::::::::::::::::::::::::::::::::::::::::::::::::::

@D0950.29_100148

GATGAACGCTGGCGGCGTGCCTAACACATGCAAGTCGAGCGATTTCCTTCGGGAAAGAGCGGCGGACGGGTGAGTAACGCGTGGGTAACCTGCCCTATACACATGGATAACATACCGAAAGGTATGCTAATACAGGATAATATGAAAGAGTCGCATGGCACTTTCATCAAAGCTCCGGCGGTATAGGATGGACCCGCGTCTGATTAGCTAGTTGGTAAGGTAATGGCTTACCAAGGCGACGATCAGTAGCCGACCTGAGAGGGTGATCGGCCACATTGGAACTGAGACACGGTCCAAACTCCTACGGGAGGCAGCAGTGGGGAATATTGCACAATGGGCGAAAGCCTGATGCAGCAACGCCGCGTGAGCGATGAAGGCCTTCGGGTCGTAAAGCTCTGTCCTCAAGGAAGATAATGACGGTACTTGAGGAGGAAGCCCCGGCTAACTACGTGCCAGCAGCCGCGGTAA

+

::::::::::::::::::::::::::::::::::::::::::::::::::::::::::::::::::::::::::::::::::::::::::::::::::::::::::::::::::::::::::::::::::::::::::::::::::::::::::::::::::::::::::::::::::::::::::::::::::::::::::::::::::::::::::::::::::::::::::::::::::::::::::::::::::::::::::::::::::::::::::::::::::::::::::::::::::::::::::::::::::::::::::::::::::::::::::::::::::::::::::::::::::::::::::::::::::::::::::::::::::::::::::::::::::::::::::::::::::::::::::::::::::::::::::::::::::::

@D0950.29_100199

GATGAACGCTAGCTACAGGCTTAACACATGCAAGTCGAGGGGTAGCATGAAACTTAGCAATAAGTTTTGATGACGACCGGCGCACGGGTGAGTAACACGTATCCAACCTGCCTTTTACTCATGGATAGCCTTCTGAAAAGAAGATTAATACATGATGGTATTCAGAGTTTTCATGGACACTGAATTAAAGATTTTATCGGTAAGAGATGGGGATGCGTTCCATTAGATAGTAGGCGGGGTAACGGCCCACCTAGTCAACGATGGATAGGGGTTCTGAGAGGAAGGTCCCCCACATTGGAACTGAGACACGGTCCAAACTCCTACGGGAGGCAGCAGTGAGGAATATTGGTCAATGACGTAAGTCTGAACCAGCCAAGTAGCGTGAAGGATGAAGGCTCTATGGGTCGTAAACTTCTTTTATAAAAGGAATAAAGTATGCCACGTGTGGTGTTTTTGTATGTACTTTATGAATAAGGATCGGCTAACTCCGTGCCAGCAGCCGC

+

:::::::::::::::::::::::::::::::::::::::::::::::::::::::::::::::::::::::::::::::::::::::::::::::::::::::::::::::::::::::::::::::::::::::::::::::::::::::::::::::::::::::::::::::::::::::::::::::::::::::::::::::::::::::::::::::::::::::::::::::::::::::::::::::::::::::::::::::::::::::::::::::::::::::::::::::::::::::::::::::::::::::::::::::::::::::::::::::::::::::::::::::::::::::::::::::::::::::::::::::::::::::::::::::::::::::::::::::::::::::::::::::::::::::::::::::::::::::::::::::::::::::::::::::::::::::

@D0950.29_100201

GATGAACGCTAGCGACAGGCCTAACACATGCAAGTCGAGGGGTAGCACAAGGTAGCAATACTGAGGTGACGACCGGCGCACGGGTGAGTAACGCGTATGCAACCTACCTGTAAGAGTGGGATAGCCTCTCGAAAGAGAGATTAATACCGCATAATACCATTTCACTGCATGGTGAGATGGTTAAAGATTTATTGCTTACAGATGGGCATGCGTAACATTAGCTAGTTGGTGAGGTAACGGCTCACCAAGGCAACGATGTTTAGGGGTTCTGAGAGGAAGGTCCCCCACACTGGTACTGAGACACGGACCAGACTCCTACGGGAGGCAGCAGTGAGGAATATTGGTCAATGGACGAGAGTCTGAACCAGCCAAGTCGCGTGAAGGATGAAGGTCTTATGGATTGTAAACTTCTTTTATACGGGAATAAAAATACCACGTGTGGTATATTGCATGTACCGTATGAATAAGGATCGGCTAACTCCGT

+

::::::::::::::::::::::::::::::::::::::::::::::::::::::::::::::::::::::::::::::::::::::::::::::::::::::::::::::::::::::::::::::::::::::::::::::::::::::::::::::::::::::::::::::::::::::::::::::::::::::::::::::::::::::::::::::::::::::::::::::::::::::::::::::::::::::::::::::::::::::::::::::::::::::::::::::::::::::::::::::::::::::::::::::::::::::::::::::::::::::::::::::::::::::::::::::::::::::::::::::::::::::::::::::::::::::::::::::::::::::::::::::::::::::::::::::::::::::::::::::::::::

@D0950.29_100259

ATTGAACGCTGGCGGCAGGCCTAACACATGCAAGTCGAACGGTAGCACAGAGAGCTTGCTCTTGGGTGACGAGTGGCGGACGGGTGAGTAATGTCTGGGAAACTGCCCGATGGAGGGGGATAACTACTGGAAACGGTAGCTAATACCGCATAATGTCGCAAGACCAAAGAGGGGGACCTTCGGGCCTCTTGCCATCGGATGTGCCCAGATGGGATTAGCTAGTAGGTGGGGTAATGGCTCACCTAGGCGACGATCCCTAGCTGGTCTGAGAGGATGACCAGCCACACTGGAACTGAGACACGGTCCAGACTCCTACGGGAGGCAGCAGTGGGGAATATTGCACAATGGGCGCAAGCCTGATGCAGCCATGCCGCGTGTATGAAGAAGGCCTTCGGGTTGTAAAGTACTTTCAGCGAGGAGGAAGGTGTTGAGGTTTAATAACCTCAGCAATTGACGTTACTCGCAGAAGAAGCACCGGCTAACTCCGTGCCAGCAGCCGCGGTAA

+

:::::::::::::::::::::::::::::::::::::::::::::::::::::::::::::::::::::::::::::::::::::::::::::::::::::::::::::::::::::::::::::::::::::::::::::::::::::::::::::::::::::::::::::::::::::::::::::::::::::::::::::::::::::::::::::::::::::::::::::::::::::::::::::::::::::::::::::::::::::::::::::::::::::::::::::::::::::::::::::::::::::::::::::::::::::::::::::::::::::::::::::::::::::::::::::::::::::::::::::::::::::::::::::::::::::::::::::::::::::::::::::::::::::::::::::::::::::::::::::::::::::::::::::::::::::::::

@D0950.29_100319

GATGAACGCTAGCTACAGGCTTAACACATGCAAGTCGAGGGTAGCATGAAACTTAGCAATAAGTTTTGATGACGACCGGCGCACGGGTGAGTAACACGTATCCAACCTGCCTTTTACTCATGGATAGCCTTCTGAAAAGAAGATTAATACATGATGGTATTCAGAGTTTTCATGGACACTGAATTAAAGATTTATCGGTAAGAGATGGGGATGCGTTCCATTAGATAGTAGGCGGGGTAACGGCCCACCTAGTCAACGATGGATAGGGGTTCTGAGAGGAAGGTCCCCCACATTGGAACTGAGACACGGTCCAAACGTCCTACGGGAGGCAGCAGTGAGGAATATTGGTCAATGGACGTAAGTCTGAACCAGCCAAGTAGCGTGAAGGATGAAGGCTCTATGGGTCGTAAACTTC

+

:::::::::::::::::::::::::::::::::::::::::::::::::::::::::::::::::::::::::::::::::::::::::::::::::::::::::::::::::::::::::::::::::::::::::::::::::::::::::::::::::::::::::::::::::::::::::::::::::::::::::::::::::::::::::::::::::::::::::::::::::::::::::::::::::::::::::::::::::::::::::::::::::::::::::::::::::::::::::::::::::::::::::::::::::::::::::::::::::::::::::::::::::::::::::::::::::::::::::::::::::::::::::::::::

@D0950.29_100325

GATGAACGCTAGCTACAGGCTTAACACATGCAAGTCGAGGGGTAGCATGAAACTTAGCAATAAGTTTTGATGACGACCGGCGCACGGGTGAGTAACACGTATCCAACCTGCCTTTTACTCATGGATAGCCTTCTGAAAAGAAGATTAATACATGATGGTATTCAGAGTTTTCATGGACACTGAATTAAAGATTTTATCGGTAAGAGATGGGGATGCGTTCCATTAGATAGTAGGCGGGGTAACGGCCCACCTAGTCAACATGGATAGGGGTTCTGAGAGGAAGGTCCCCCACATTGGAACTGAGACACGGTCCAAACGTCCTACGGGAGGCAGCAGTGAGGAATATTGGTCAATGGACGTAAGTCTGAACCAGCCAAGT

+

:::::::::::::::::::::::::::::::::::::::::::::::::::::::::::::::::::::::::::::::::::::::::::::::::::::::::::::::::::::::::::::::::::::::::::::::::::::::::::::::::::::::::::::::::::::::::::::::::::::::::::::::::::::::::::::::::::::::::::::::::::::::::::::::::::::::::::::::::::::::::::::::::::::::::::::::::::::::::::::::::::::::::::::::::::::::::::::::::::::::::::::::::::::::::::

@D0950.29_100352

GATGAACGCTAGCGACAGGCCTAACACATGCAAGTCGAGGGGTAGCACAAGGTAGCAATACTGAGGTGACGACCGGCGCACGGGTGAGTAACGCGTATGCAACCTACCTGTAAGAGTGGGATAGCCTCTCGAAAGAGAGATTAATACCGCATAATACCATTTCACTGCATGGTGAGATGGTTAAAGATTTATTGCTTACAGATGGGCATGCGTAACATTAGCTAGTTGGTGAGGTAACGGCTCACCAAGGCAACGATGTTTAGGGGTTCTGAGAGGAAGGTCCCCCACACTGGTACTGAGACACGGACCAGACTCCTACGGGAGGCAGCAGTGAGGAATATTGGTCAATGGACGAGAGTCTGAACCAGCCAAGTCGCGTGAAGGATGAAGGTCTTATGGATTGTAAACTTCTTTTATACGGGAATAAAAATGCCACGTGTGGCATATTGCATGTACCGTATGAATAAGGATCGGCTAACTCCGTGCCAGCAGCCGCGGTAA

+

:::::::::::::::::::::::::::::::::::::::::::::::::::::::::::::::::::::::::::::::::::::::::::::::::::::::::::::::::::::::::::::::::::::::::::::::::::::::::::::::::::::::::::::::::::::::::::::::::::::::::::::::::::::::::::::::::::::::::::::::::::::::::::::::::::::::::::::::::::::::::::::::::::::::::::::::::::::::::::::::::::::::::::::::::::::::::::::::::::::::::::::::::::::::::::::::::::::::::::::::::::::::::::::::::::::::::::::::::::::::::::::::::::::::::::::::::::::::::::::::::::::::::::::::::::::

@D0950.29_100377

GATGAACGCTGGCGGCGTGCTTAACACATGCAAGTCGAACGAAGCGCTGGAGGAGCTTGCTCCAAAGGTGACTGAGTGGCGGACGGGTGAGTAACGCGTGGGTAACCTGCCTTACACTGGGGGATAACAGTTGGAAACGACTGCTAATACCGCATAAGCGCACAGTATTGCATGATACAGTGTGAAAAACTCCGGTGGTGTAAGATGGACCCGCGTCTGATTAGCTAGTTGGTGAGGTAATGGCTCACCAAGGCAACGATCAGTAGCCGGCTTGAGAGAGTGAACGGCCACATTGGGACTGAGACACGGCCCAAACTCCTACGGGAGGCAGCAGTGGGGAATATTGCACAATGGGGGAAACCCTGATGCAGCAACGCCGCGTGAGTGAAGAAGTATTTCGGTATGTAAAGCTCTATCAGCAGGGAAGATAATGACGGTACCTGACTAAGAAGCCCCGGCTAACTACGTGCCAGCAGCCGCGGTAA

+

:::::::::::::::::::::::::::::::::::::::::::::::::::::::::::::::::::::::::::::::::::::::::::::::::::::::::::::::::::::::::::::::::::::::::::::::::::::::::::::::::::::::::::::::::::::::::::::::::::::::::::::::::::::::::::::::::::::::::::::::::::::::::::::::::::::::::::::::::::::::::::::::::::::::::::::::::::::::::::::::::::::::::::::::::::::::::::::::::::::::::::::::::::::::::::::::::::::::::::::::::::::::::::::::::::::::::::::::::::::::::::::::::::::::::::::::::::::::::::::::::::::

@D0950.29_10059

GGTGAACGCTGGCGGCGTGCCTAAGACATGCAAGTCGAACGACGCAGCTTGCTGCGTAGTGGCGCACGGGTGAGTAACACGTAACTGACCTACCCCGAAGTCCGGCATAACAGTCCGAAAGGACTGCTAATTCAGGATGTGAAGCCAGCTTCTGAGCTGTCTTTAAAGATTCATCGCTTCGGGATGGGGTTGCGGCGCATCAGCTTGTTGGTGAGGTAAAGGCTCACCAAGGCAACGACGCGTAACCGGCCTGAGAGGGTGGTCGGTCACAGGGGCACTGAGACACGGGTCCCACTCCTACGGGAGGCAGCAGTTAGGAATCTTCCCCAATGGGCGCAAGCCTGAGGGAGCGACGCCGCGTGCGGGATGAAGGCCCTCGGGTCGTAAACCGCTGAAACGAGGACGAAAACCCCGCAAGGGATCTGACGGTACTCGTGTAATAGCACCGGCTAACTCCGTGCCAGCAGCCGCGG

+

:::::::::::::::::::::::::::::::::::::::::::::::::::::::::::::::::::::::::::::::::::::::::::::::::::::::::::::::::::::::::::::::::::::::::::::::::::::::::::::::::::::::::::::::::::::::::::::::::::::::::::::::::::::::::::::::::::::::::::::::::::::::::::::::::::::::::::::::::::::::::::::::::::::::::::::::::::::::::::::::::::::::::::::::::::::::::::::::::::::::::::::::::::::::::::::::::::::::::::::::::::::::::::::::::::::::::::::::::::::::::::::::::::::::::::::::::::::::::

@D0950.29_100593

GATGAACGCTAGCGACAGGCCTAACACATGCAAGTCGAGGGGTAGCACAAGGTAGCAATACTGAGGTGACGACCGGCGCACGGGTGAGTAACGCGTATGCAACCTACCTGTAAGAGTGGGATAGCCTCTCGAAAGAGAGATTAATACCGCATAATACCATTTCACTGCATGGTGAGATGGTTAAAGATTTATTGCTTACAGATGGGCATGCGTAACATTAGCTAGTTGGTGAGGTAACGGCTCACCAAGGCAACGATGTTTAGGGGTTCTGAGAGGAAGGTCCCCCACATTGGAACTGAGACACGGTCCAAACTCCTACGGGAGGCAGCAGTGAGGAATATTGGTCAATGGACGTAAGTCTGAACCAGCCAAGTAGCGTGAAGGATGAAGGCTCTATGGGTCGTAAACTTCTTTTATAAAGGAATAAAGTATGCCACGTGTGGTGTTTTGTATGTAC

+

:::::::::::::::::::::::::::::::::::::::::::::::::::::::::::::::::::::::::::::::::::::::::::::::::::::::::::::::::::::::::::::::::::::::::::::::::::::::::::::::::::::::::::::::::::::::::::::::::::::::::::::::::::::::::::::::::::::::::::::::::::::::::::::::::::::::::::::::::::::::::::::::::::::::::::::::::::::::::::::::::::::::::::::::::::::::::::::::::::::::::::::::::::::::::::::::::::::::::::::::::::::::::::::::::::::::::::::::::::::::::::::::::::::::::

@D0950.29_100642

GATGAACGCTGGCGGCGTGCTTAACACATGCAAGTCGAACGAAGCAGCTTTCTTGCTTGCAAGAAAGCTGACTTAGTGGCGGACGGGTGAGTAACGCGTGGGTAACCTGCCTCATACAGGGGATAACAGTTGGAAACGACTGCTAAGACCGCATAACCCGCTAGTGTCGCATGACACGGGACGGAAAATATTTTATAGGTATGAGATGGGCCCGCGTCTGATTAGCTAGTTGGTAAGGTAACGGCTTACCAAGGCGACGATCAGTAGCCGACTTGAGAGAGTGATCGGCCACATTGGGACTGAGACACGGCCCAAACTCCTACGGGAGGCAGCAGTGGGGAATATTGGACAATGGGGGAAACCCTGATCCAGCGACGCCGCGTGAGTGAAGAAGTATTTCGGTATGTAAAGCTCTATCAGCA

+

::::::::::::::::::::::::::::::::::::::::::::::::::::::::::::::::::::::::::::::::::::::::::::::::::::::::::::::::::::::::::::::::::::::::::::::::::::::::::::::::::::::::::::::::::::::::::::::::::::::::::::::::::::::::::::::::::::::::::::::::::::::::::::::::::::::::::::::::::::::::::::::::::::::::::::::::::::::::::::::::::::::::::::::::::::::::::::::::::::::::::::::::::::::::::::::::::::::::::::::::::::::::::::::::::::::

@D0950.29_100666

GATGAACGCTAGCGACAGGCCTAACACATGCAAGTCGAGGGGCAGCGGGAGTGTAGCAATACACTTGCCGGCGACCGGCGCACGGGTGAGTAACACGTATGCGACCTACCCATAGCAGGGGGATAATCGGAAGAAATTCCGTCTAATACCGCGTAATAATTCAGATCTGCATGGATTTGAATTTAAAGGAGCAATCCGGCTATGGATGGGCATGCGGGACATTAGCTAGTTGGCGGGGTAACGGCCCACCAAGGCTTCGATGTCTAGGGGTTCTGAGAGGAAGGTCCCCCACACTGGTACTGAGACACGGACCAGACTCCTACGGGAGGCAGCAGTGAGGAATATTGGTCAATGGTCGAGAGACTGAACCAGCCAAGTCGCGTGAGGGATGAAGGCTCTATGGGTCGTAAACTTCTTTTATAAAGGAATAAAGTATGCCACGTGTGGTGTTTTGTATGTACTTTATGAATAAGGATCGGCTAACTCCGTGCCAGCAGCCGCGGTAA

+

::::::::::::::::::::::::::::::::::::::::::::::::::::::::::::::::::::::::::::::::::::::::::::::::::::::::::::::::::::::::::::::::::::::::::::::::::::::::::::::::::::::::::::::::::::::::::::::::::::::::::::::::::::::::::::::::::::::::::::::::::::::::::::::::::::::::::::::::::::::::::::::::::::::::::::::::::::::::::::::::::::::::::::::::::::::::::::::::::::::::::::::::::::::::::::::::::::::::::::::::::::::::::::::::::::::::::::::::::::::::::::::::::::::::::::::::::::::::::::::::::::::::::::::::::::::::::

@D0950.29_100688

GATGAACGCTAGCGACAGGCCTAACACATGCAAGTCGAGGGGTAGCACAAGGTAGTAATACTGAGGTGACGACCGGCGCACGGGTGAGTAACGCGTATGCAACCTACCTGTAAGAGTGGGATAGCCTCTCGAAAGAGAGATTAATACCGCATAATACCATTTCACTGCATGGTGAGATGGTTAAAGATTTATTGCTTACAGATGGGCATGCGTAACATTAGCTAGTTGGTGAGGTAACGGCTCACCAAGGCAACGATGTTTAGGGGTTCTGAGAGGAAGGTCCCCCACACTGGTACTGAGACACGGACCAGACTCCTACGGGAGGCAGCAGTGAGGAATATTGGTCAATGGACGAGAGTCTGAACCAGCCAAGTCGCGTGAAGGATGAAGGTCTTATGGATTGTAAACTTCTTTTATACGGGAATAAAAAATGCCACGTGTGGCATATTGCATGTACCGTATGAATAAGGATCGGCT

+

:::::::::::::::::::::::::::::::::::::::::::::::::::::::::::::::::::::::::::::::::::::::::::::::::::::::::::::::::::::::::::::::::::::::::::::::::::::::::::::::::::::::::::::::::::::::::::::::::::::::::::::::::::::::::::::::::::::::::::::::::::::::::::::::::::::::::::::::::::::::::::::::::::::::::::::::::::::::::::::::::::::::::::::::::::::::::::::::::::::::::::::::::::::::::::::::::::::::::::::::::::::::::::::::::::::::::::::::::::::::::::::::::::::::::::::::::::::::::::::

@D0950.29_100731

GATGAACGCTAGCTACAGGCTTAACACATGCAAGTCGAGGGGTAGCATGAAACTTAGCAATAAGTTTTGATGACGACCGGCGCACGGGTGAGTAACACGTATCCAACCTGCCTTTTACTCATGGATAGCCTTCTGAAAAGAAGATTAATACATGATGGTATTCAGAGTTTTCATGGACACTGAATTAAAGATTTTATCGGTAAGAGATGGGGATGCGTTCCATTAGATAGTAGGCGGGGTAACGGCCCACCTAGTCAACGATGGATAGGGGTTCTGAGAGGAAGGTCCCCCACATTGGAACTGAGACACGGTCCAAACGTCCTACGGGAGGCAGCAGTGAGGAATATTGGTCAATGGACGTAAGTCTGAACCAGCCAAGTAGCGTGAAGGATGAAGGCTCTATGGGTCGTAAACTTCTTTTTATAAAAGGAATAAAGTATGCCACGTGTGGTGTTTTTGTATGT

+

::::::::::::::::::::::::::::::::::::::::::::::::::::::::::::::::::::::::::::::::::::::::::::::::::::::::::::::::::::::::::::::::::::::::::::::::::::::::::::::::::::::::::::::::::::::::::::::::::::::::::::::::::::::::::::::::::::::::::::::::::::::::::::::::::::::::::::::::::::::::::::::::::::::::::::::::::::::::::::::::::::::::::::::::::::::::::::::::::::::::::::::::::::::::::::::::::::::::::::::::::::::::::::::::::::::::::::::::::::::::::::::::::::::::::::::::

@D0950.29_100743

GATGAACGCTAGCTACAGGCTTAACACATGCAAGTCGAGGGGTAGCATGAAACTTAGCAATAAGTTTTGATGACGACCGGCGCACGGGTGAGTAACACGTATCCAACCTGCCTTTTACTCATGGATAGCCTTCTGAAAAGAAGATTAATACATGATGGTATTCAGAGTTTTCATGGACACTGAATTAAAGATTTTATCGGTAAGAGATGGGGATGCGTTCCATTAGATAGTAGGCTGGGGTAACGGCCCACCTAGTCGAACGATGGATAGGGGTTCTGAGAGGAAGGTCCCCCACATTGGAACTGAGACACGGTCCAAACGTCCTACGGGAGGCAGCAGTGAGGAATATTGGTCAATGGACGTAAGTCTGAACCAGCCAAGTAGCGTGAAGGATGAAGGCTCTATGGGTCGTAAACTTCTTTTATAAAAGGAATAAAGTATGCCACGTGTGGTGTTTTGTATGTACTTTATGAATAAGGATCGGCT

+

::::::::::::::::::::::::::::::::::::::::::::::::::::::::::::::::::::::::::::::::::::::::::::::::::::::::::::::::::::::::::::::::::::::::::::::::::::::::::::::::::::::::::::::::::::::::::::::::::::::::::::::::::::::::::::::::::::::::::::::::::::::::::::::::::::::::::::::::::::::::::::::::::::::::::::::::::::::::::::::::::::::::::::::::::::::::::::::::::::::::::::::::::::::::::::::::::::::::::::::::::::::::::::::::::::::::::::::::::::::::::::::::::::::::::::::::::::::::::::::::::::::

@D0950.29_10075

GATGAACGCTAGCTACAGGCTTAACACATGCAAGTCGAGGGGTAGCATGAAACTTAGCAATAAGTTTTGATGACGACCGGCGCACGGGTGAGTAACACGTATCCAACCTGCCTTTTACTCATGGATAGCCTTCTGAAAAGAAGATTAATACATGATGGTATTCAGAGTTTCCATGGACACTGAATTAAAGATTTATCGGTAAGAGATGGGGATGCGTTCCATTAGATAGTAGGCGGGGTAACGGCCCACCTAGTCAACGATGGATAGGGGTTCTGAGAGGAAGGTCCCCCACATTGGAACTGAGACACGGTCCAAACGTCCTACGGGAGGCAGCAGTGAGGAATATTGGTCAATGGACGTAAGTCTGAACCAGCCAAGTAGCGTGAAGGATGAAGGCTCTATGGGTCGTAAACTTCTTTTATAAAAGGAATAAAGTATGCCACGTGTGGTGTTTTGTATGTACTTTATGAATAAGGATCGGCTAACTCCGTGCCAGCAGCCGC

+

:::::::::::::::::::::::::::::::::::::::::::::::::::::::::::::::::::::::::::::::::::::::::::::::::::::::::::::::::::::::::::::::::::::::::::::::::::::::::::::::::::::::::::::::::::::::::::::::::::::::::::::::::::::::::::::::::::::::::::::::::::::::::::::::::::::::::::::::::::::::::::::::::::::::::::::::::::::::::::::::::::::::::::::::::::::::::::::::::::::::::::::::::::::::::::::::::::::::::::::::::::::::::::::::::::::::::::::::::::::::::::::::::::::::::::::::::::::::::::::::::::::::::::::::::::::::

@D0950.29_100794

GATGAACGCTAGCTACAGGCTTAACACATGCAAGTCGAGGGGTAGCATGAAACTTAGCAATAAGTTTTGATGACGACCGGCGCACGGGTGAGTAACACGTATCCAACCTGCCTGTAAGAGTGGGATAGCCTCTCGAAAGAGAGATTAATACCGCATAATACCATTTCACTGCATGGTGAGATGGTTAAAGATTTATTGCTTACAGATGGGCATGCGTAACATTAGCTAGTTGGTGAGGTAACGGCTCACCAAGGCAACGATGTTTAGGGGTTCTGAGAGGAAGGTCCCCCACACTGGTACTGAGACACGGACCAGACTCCTACGGGAGGCAGCAGTGAGGAATATTGGTCAATGGACGAGAGTCTGAACCAGCCAAGTCGCGTGAAGGATGAAGGTCTTATGGATTGTAAACTTCTTTTATACGGGGAATAAAAAATGCCACGTGTGGCATATTGCATGTACCGTATGAATAAGGATCGGCTAACTCCGTGCCAGCAGCCG

+

:::::::::::::::::::::::::::::::::::::::::::::::::::::::::::::::::::::::::::::::::::::::::::::::::::::::::::::::::::::::::::::::::::::::::::::::::::::::::::::::::::::::::::::::::::::::::::::::::::::::::::::::::::::::::::::::::::::::::::::::::::::::::::::::::::::::::::::::::::::::::::::::::::::::::::::::::::::::::::::::::::::::::::::::::::::::::::::::::::::::::::::::::::::::::::::::::::::::::::::::::::::::::::::::::::::::::::::::::::::::::::::::::::::::::::::::::::::::::::::::::::::::::::::::::::::

@D0950.29_100819

GATGAACGCTAGCTACAGGCTTAACACATGCAAGTCGAGGGGTAGCATGAAACTTAGCAATAAGTTTTGATGACGACCGGCGCACGGGTGAGTAACACGTATCCAACCTGCCTTTTACTCATGGATAGCCTTCTGAAAAGAAGATTAATACATGATGGTATTCAGAGTTTTCATGGACACTGAATTAAAGATTTTATCGGTAAGAGATGGGGATGCGTTCCATTAGATAGTAGGCGGGGTAACGGCCCACCTAGTCAACGATGGATAGGGGTTCTGAGAGGAAGGTCCCCACATTGGAACTGAGACACGGTCCAAACTCCTACGGGAGGCAGCAGTGAGGAATATTGGTCAATGGACGTAAGTCTGAACCAGCCAAGTAGCGTGAAGGATGAAGGCTCTATGGGTCGTAAACTTCTTTTTATAAAAGGAATAAAGTATGCCACGTGTGGTGTTTTTGTATGTAACTTTATGAATAAGGATCGGCTAACTCCGTGCCAGCAGCC

+

:::::::::::::::::::::::::::::::::::::::::::::::::::::::::::::::::::::::::::::::::::::::::::::::::::::::::::::::::::::::::::::::::::::::::::::::::::::::::::::::::::::::::::::::::::::::::::::::::::::::::::::::::::::::::::::::::::::::::::::::::::::::::::::::::::::::::::::::::::::::::::::::::::::::::::::::::::::::::::::::::::::::::::::::::::::::::::::::::::::::::::::::::::::::::::::::::::::::::::::::::::::::::::::::::::::::::::::::::::::::::::::::::::::::::::::::::::::::::::::::::::::::::::::::::::::::

@D0950.29_100836

GATGAACGCTAGCTACAGGCTTAACACATGCAAGTCGAGGGGTAGCATGAAACTTAGCAATAAGTTTTGATGACGACCGGCGCACGGGTGAGTAACACGTATCCAACCTGCCTTTTACTCATGGATAGCCTTCTGAAAAGAAGATTAATACATGATGGTATTCAGAGTTTTCATGGACACTGAATTAAAGATTTTATCGGTAAGAGATGGGGATGCGTTCCATTAGATAGTAGGCGGGGTAACGGCCCACCTAGTCAACGAATGGATAGGGGTTCTGAGAGGAAGGTCCCCCACATTGGAACTGAGACACGGTCCAAACTCCGTACGGGAGGCAGCAGTGAGGAATATTGGTCAATGGACGTAAGTCTGAACCAGCCAAGTAGCGTGAAGGATGAAGGCTC

+

:::::::::::::::::::::::::::::::::::::::::::::::::::::::::::::::::::::::::::::::::::::::::::::::::::::::::::::::::::::::::::::::::::::::::::::::::::::::::::::::::::::::::::::::::::::::::::::::::::::::::::::::::::::::::::::::::::::::::::::::::::::::::::::::::::::::::::::::::::::::::::::::::::::::::::::::::::::::::::::::::::::::::::::::::::::::::::::::::::::::::::::::::::::::::::::::::::::::::::::::::

@D0950.29_100841

GATGAACGCTGGCGGCGTGCTTAACACATGCAAGTCGAACGAAGCGCTGGAGGAGCTTGCTCCAAAGGTGACTGAGTGGCGGACGGGTGAGTAACGCGTGGGTAACCTGCCTTACACTGGGGGATAACAGTTGGAAACGACTGCTAATACCGCATAAGCGCACAGTATTGCATGATACAGTGTGAAAAACTCCGGTGGTGTAAGATGGACCCGCGTCTGATTAGCTAGTTGGTGAGGTAATGGCTCACCAAGGCAACGATCAGTAGCCGGCTTGAGAGAGTGAACGGCCACATTGGGACTGAGACACGGCCCAAACTCCTACGGGAGGCAGCAGTGGGGAATATTGCACAATGGGGGAAACCCTGATGCAGCGACGCCGCGTGAAGGAAGAAGTATTTCGGTATGTAAACTTCTATCAGCAGGGAAGAAAATGACGGTACCTGACTAAGAAGCCCGGCTAACTACGTGCCAGCAGCCGCGGTAA

+

::::::::::::::::::::::::::::::::::::::::::::::::::::::::::::::::::::::::::::::::::::::::::::::::::::::::::::::::::::::::::::::::::::::::::::::::::::::::::::::::::::::::::::::::::::::::::::::::::::::::::::::::::::::::::::::::::::::::::::::::::::::::::::::::::::::::::::::::::::::::::::::::::::::::::::::::::::::::::::::::::::::::::::::::::::::::::::::::::::::::::::::::::::::::::::::::::::::::::::::::::::::::::::::::::::::::::::::::::::::::::::::::::::::::::::::::::::::::::::::::::::

@D0950.29_100844

GATGAACGCTAGCTACAGGCTTAACACATGCAAGTCGAGGGGTAGCATGAAACTTAGCAATAAGTTTTGATGACGACCGGCGCACGGGTGAGTAACACGTATCCAACCTGCCTTTTACTCATGGATAGCCTTCTGAAAAGAAGATTAATACATGATGGTATTCAGAGTTTTCATGGACACTGAATTAAAGATTTTATCGGTAAGAGATGGGGATGCGTTCCATTAGATAGTAGGCGGGGTAACGGCCCACCTAGTCAACGATGGATAGGGGTTCTGAGAGGAGGTCCCCACATTGGAACTGAGACACGGTCCAAACGTCCTACGGGAGGCAGCAGTGAGGAATATTGGTCAATGGACGTAAGTCTGAACCAGCCAAGTAGCGTGAAGGATGAAGGCTCTATGGGTCGTAAACTTCTTTTATAAAAGGAATAAAGTATGCCACGTGTGGTGTTTTTGTATGTACTTTATGAATAAGGATCGGCTAACTCCGTGCCAGCAGCCGC

+

:::::::::::::::::::::::::::::::::::::::::::::::::::::::::::::::::::::::::::::::::::::::::::::::::::::::::::::::::::::::::::::::::::::::::::::::::::::::::::::::::::::::::::::::::::::::::::::::::::::::::::::::::::::::::::::::::::::::::::::::::::::::::::::::::::::::::::::::::::::::::::::::::::::::::::::::::::::::::::::::::::::::::::::::::::::::::::::::::::::::::::::::::::::::::::::::::::::::::::::::::::::::::::::::::::::::::::::::::::::::::::::::::::::::::::::::::::::::::::::::::::::::::::::::::::::::

@D0950.29_100854

GATGAACGCTGGCGGCGTGCTTAACACATGCAAGTCGAACGAAGCAGCTTTCTTGCTTGCAAGAAAGCTGACTTAGTGGCGGACGGGTGAGTAACGCGTGGGTAACCTGCCTCATACAGGGGGATAACAGTTGGAAACGACTGCTAAGACCGCATAACCCGCTAGTGTCGCATGACACGGACGGAAAATATTTTATAGGTATGAGATGGGCCCGCGTCTGATTAGCTAGTTGGTAAGGTAACGGCTTACCAAGGCGACGATCAGTAGCCGACTTGAGAGAGTGATCGGCCACATTGGGACTGAGACACGGCCCAAACTCCTACGGGAGGCAGCAGTGGGGAATATTGGACAATGGGGGAAACCCTGATCCAGCGACGCCGCGTGAGTGAAGAAGTATTTCGGTATGTAAAGCTCTATCAGCAGGGAAGATAATGACAGTACCTGACTAAGAAGCCCCCGGCTAACTACGTGCCAGCAGCCGC

+

::::::::::::::::::::::::::::::::::::::::::::::::::::::::::::::::::::::::::::::::::::::::::::::::::::::::::::::::::::::::::::::::::::::::::::::::::::::::::::::::::::::::::::::::::::::::::::::::::::::::::::::::::::::::::::::::::::::::::::::::::::::::::::::::::::::::::::::::::::::::::::::::::::::::::::::::::::::::::::::::::::::::::::::::::::::::::::::::::::::::::::::::::::::::::::::::::::::::::::::::::::::::::::::::::::::::::::::::::::::::::::::::::::::::::::::::::::::::::::::::::

@D0950.29_100883

GATGAACGCTAGCTACAGGCTTAACACATGCAAGTCGAGGGGTAGCATGAAACTTAGCAATAAGTTTTGATGACGACCGGCGCACGGGTGAGTAACACGTATCCAACCTGCCTTTTACTCATGGATAGCCTTCTGAAAAGAAGATTAATACATGATGGTATTCAGAGTTTTCATGGACACTGAATTAAAGATTTTATCGGTAAGAGATGGGGATGCGTTCCATTAGATAGTAGGCGGGGTAACGGCCCACCTAGTCAACGATGGATAGGGGTTCTGAGAGGAAGGTCCCCCACATTGGAACTGAGACACGGTCCAAACGTCCGTACGGGAGGCAGCAGTGAGGAATATTGTCAATGGACGTAAGTCTGAACCAGCCAAGTAGCGTGAAGGATGAAGGCTCTATGGGTCGTAAACTTCTTTTTATAAAAGG

+

::::::::::::::::::::::::::::::::::::::::::::::::::::::::::::::::::::::::::::::::::::::::::::::::::::::::::::::::::::::::::::::::::::::::::::::::::::::::::::::::::::::::::::::::::::::::::::::::::::::::::::::::::::::::::::::::::::::::::::::::::::::::::::::::::::::::::::::::::::::::::::::::::::::::::::::::::::::::::::::::::::::::::::::::::::::::::::::::::::::::::::::::::::::::::::::::::::::::::::::::::::::::::::::::::::::::::::::

@D0950.29_100894

GATGAACGCTAGCTACAGGCTTAACACATGCAAGTCGAGGGGTAGCATGAAACTTAGCAATAAGTTTTGATGACGACCGGCGCACGGGTGAGTAACACGTATCCAACCTGCCTTTTACTCATGGATAGCCTTCTGAAAAGAAGATTAATACATGATGGTATTCAGAGTTTTCATGGACACTGAAATTAAAGATTTTATCGGTAAGAGATGGGGATGCGTTCCATTAGATAGTAGGCGGGGTAACGGCCCACCTAGTCAACGATGGATAGGGGTTCTGAGAGGAAGGTACCCCCACATTGGAACTGAGACACGGTCCAAACGTCCTACGGGAGGCAGCAGTGAGGAATATTGGTCAATGGACGTAAGTCTGAACCAGCCAAGTAGCGTGAAGGATGAAGGCTCTATGGGTCGTAAAACTTCTTTTATAAAAGGAATAAAGTATGCCACGTGTGGTGTTTTTGTATGT

+

::::::::::::::::::::::::::::::::::::::::::::::::::::::::::::::::::::::::::::::::::::::::::::::::::::::::::::::::::::::::::::::::::::::::::::::::::::::::::::::::::::::::::::::::::::::::::::::::::::::::::::::::::::::::::::::::::::::::::::::::::::::::::::::::::::::::::::::::::::::::::::::::::::::::::::::::::::::::::::::::::::::::::::::::::::::::::::::::::::::::::::::::::::::::::::::::::::::::::::::::::::::::::::::::::::::::::::::::::::::::::::::::::::::::::::::::::

@D0950.29_100898

GATGAACGCTAGCGACAGGCCTAACACATGCAAGTCGAGGGGTAGCACAAGGAAGCTTGCTTCTGAGGTGACGACCGGCGCACGGGTGAGTAACGCGTATGCAACCTACCTGTAAGAGTGGGATAGCCTCTCGAAAGAGAGATTAATACCGCATAATACCATTTCACTGCATGGTGAGATGGTTAAAGATTTATTGCTTACAGATGGGCATGCGTAACATTAGCTAGTTGGTGAGGTAACGGCTCACCAAGGCAACGATGTTTAGGGGTTCTGAGAGGAAGGTCCCCCACACTGGTACTGAGACACGGACCAGACTCCTACGGGAGGCAGCAGTGAGGAATATTGGTCAATGGACGAGAGTCTGAACCAGCCAAGTCGCGTGAAGGATGAAGGTCTTATGGATTGTAAACTTCTTTTATACGGGAATAAAAATGCCACGTGTGGCATATTGCATGTACCGTATGAATAAGGATCGGCTAACTCCGTGCCAGCAGCCGCGGTAA

+

:::::::::::::::::::::::::::::::::::::::::::::::::::::::::::::::::::::::::::::::::::::::::::::::::::::::::::::::::::::::::::::::::::::::::::::::::::::::::::::::::::::::::::::::::::::::::::::::::::::::::::::::::::::::::::::::::::::::::::::::::::::::::::::::::::::::::::::::::::::::::::::::::::::::::::::::::::::::::::::::::::::::::::::::::::::::::::::::::::::::::::::::::::::::::::::::::::::::::::::::::::::::::::::::::::::::::::::::::::::::::::::::::::::::::::::::::::::::::::::::::::::::::::::::::::::::

@D0950.29_100903

GATGAACGCTGGCGGCGTGCTTAACACATGCAAGTCGAACGAAGCACTTAAGGAGCTTGCTCCAAAAGTGACTGAGTGGCGGACGGGTGAGTAACGCGTGGGTAACCTGCCTTACACTGGGGGATAACAGTTGGAAACGACTGCTAATACCGCATAAGCGCACAGTATTGCATGATACAGTGTGAAAAACTCCGGTGGTGTAAGATGGACCCGCGTCTGATTAGCTAGTTGGTGAGGTAATGGCTCACCAAGGCAACGATCAGTAGCCGGCTTGAGAGAGTGAACGGCCACATTGGGACTGAGACACGGCCCAAACTCCTACGGGAGGCAGCAGTGGGGAATATTGCACAATGGGGGAAACCCTGATGCAGCAACGCCGCGTGAGTGAAGAAGTATTTGCGGTATGTAAAGCTCTATCAGCAGGGAAGATAATGACGGTACCTGACTAAGAAGCCCCCGGCTAACTACGTGCCAGCAGCCG

+

:::::::::::::::::::::::::::::::::::::::::::::::::::::::::::::::::::::::::::::::::::::::::::::::::::::::::::::::::::::::::::::::::::::::::::::::::::::::::::::::::::::::::::::::::::::::::::::::::::::::::::::::::::::::::::::::::::::::::::::::::::::::::::::::::::::::::::::::::::::::::::::::::::::::::::::::::::::::::::::::::::::::::::::::::::::::::::::::::::::::::::::::::::::::::::::::::::::::::::::::::::::::::::::::::::::::::::::::::::::::::::::::::::::::::::::::::::::::::::::::::

@D0950.29_100907

GATGAACGCTAGCTACAGGCTTAACACATGCAAGTCGAGGGGTAGCATGAAACTTAGCAATAAGTTTTGATGACGACCGGCGCACGGGTGAGTAACACGTATCCAACCTGCCTTTTACTCATGGATAGCCTTCTGAAAAGAAGATTAATACATGATGGTATTCAGAGTTTTCATGGACACTGAATTAAAGATTTTATCGGTAAGAGATGGGGATGCGTTCCATTAGATAGTAGGCGGGGTAACGGCCCACCTAGTCAACGATGGATAGGGGTTCTGAGAGGAAGGTCCCCCACATTGGAACTGAGACACGGTCCAAACTCCTACGGGAGGCAGCAGTGAGGAATATTGGTCAATGGACGTAAGTCTGAACCAGCCAAGTAGCGTGAAGGATGAAGGCTCTATGGGTCGTAAACTTCTTTTTATAAAAGGAATAAAGTATGCCACGTGTGGTGTTTTTGTATGTACTTTATGAATAAGGATCGGCTAACTCCGTGCCAGCAGCCGC

+

:::::::::::::::::::::::::::::::::::::::::::::::::::::::::::::::::::::::::::::::::::::::::::::::::::::::::::::::::::::::::::::::::::::::::::::::::::::::::::::::::::::::::::::::::::::::::::::::::::::::::::::::::::::::::::::::::::::::::::::::::::::::::::::::::::::::::::::::::::::::::::::::::::::::::::::::::::::::::::::::::::::::::::::::::::::::::::::::::::::::::::::::::::::::::::::::::::::::::::::::::::::::::::::::::::::::::::::::::::::::::::::::::::::::::::::::::::::::::::::::::::::::::::::::::::::::::

@D0950.29_100980

GACGAACGCTGGCGGCGTGCTTAACACATGCAAGTCGAGCGATGAAGCTTCTTCGGAAGTGGATTAGCGGCGGACGGGTGAGTAACACGTGGGTAACCTGCCTCATAGAGGGGAATAGCCTTTCGAAAGGAAGATTAATACCGCATAAGATTGTAATACCGCATGGTATAGCAATTAAAGGAGTAATCCGCTATGAGATGGACCCGCGTCGCATTAGCTAGTTGGTGAGGTAACGGCTCACCAAGGCGACGATGCGTAGCCGACCTGAGAGGGTGATCGGCCACATTGGGACTGAGACACGGCCCAGACTCCTACGGGAGGCAGCAGTGGGGAATATTGCACAATGGGGGAAACCCTGATGCAGCAACGCCGCGTGAGTGATGACGGCCTCGGGTTGTAAAAACTCTGTCTTTGGGGACGAT

+

::::::::::::::::::::::::::::::::::::::::::::::::::::::::::::::::::::::::::::::::::::::::::::::::::::::::::::::::::::::::::::::::::::::::::::::::::::::::::::::::::::::::::::::::::::::::::::::::::::::::::::::::::::::::::::::::::::::::::::::::::::::::::::::::::::::::::::::::::::::::::::::::::::::::::::::::::::::::::::::::::::::::::::::::::::::::::::::::::::::::::::::::::::::::::::::::::::::::::::::::::::::::::::::::::::::

@D0950.29_101080

GATGAACGCTAGCGACAGGCCTAACACATGCAAGTCGAGGGGTAGCACAAGGTAGCAATACTGAGGTGACGACCGGCGCACGGGTGAGTAACGCGTATGCAACCTACCTGTAAGAGTGGGATAGCCTCTCGAAAGAGAGATTAATACCGCATAATACCATTTCACTGCATGGTGAGATGGTTAAAGATTTATTGCTTACAGATGGGCATGCGTAACATTAGCTAGTTGGTGAGGTAACGGCTCACCAAGGCAACGATGTTTAGGGGTTCTGAGAGGAAGGTCCCCCACACTGGTACTGAGACACGGACCAGACTCCTACGGGAGGCAGCAGTGAGGAATATTGGTCAATGGACGAGAGTCTGAACCAGCCAAGTCGCGTGAAGGATGAAGGTCTTATGGATTGTAAACTTCTTTTATACGGGAATAAAAATTGCCACGTGTGGCATATTGCATGTACCGTATGAATAAGGATCGGCTAACTCCGTGCCAGCAGCCGCGGTAA

+

::::::::::::::::::::::::::::::::::::::::::::::::::::::::::::::::::::::::::::::::::::::::::::::::::::::::::::::::::::::::::::::::::::::::::::::::::::::::::::::::::::::::::::::::::::::::::::::::::::::::::::::::::::::::::::::::::::::::::::::::::::::::::::::::::::::::::::::::::::::::::::::::::::::::::::::::::::::::::::::::::::::::::::::::::::::::::::::::::::::::::::::::::::::::::::::::::::::::::::::::::::::::::::::::::::::::::::::::::::::::::::::::::::::::::::::::::::::::::::::::::::::::::::::::::::::

@D0950.29_101083

GATGAACGCTGGCGGCGTGCTTAACACATGCAAGTCGAACGAAGCAACTTTCTTGCTTGCAAGAAAGTTGACTGAGTGGCGGACGGGTGAGTAACGCGTGGGTAACCTGCCTCATAGCAGGGGGATAACAGTTAGAAATGACTGCTAACACCGCATAACCCGCTAGCATCGCATGATGCAGACGGAAAATATTTATAGGTATGAGATGGGCCCGCGTCTGATTACGCTAGTTGGTGGGGTAACAGCCCACCAAGGCAACGATCAGTAGCCGACTTGAGAGAGTGATCGGCCACATTGGGACTGAGACACGGCCCAAACTCCTACGGGAGGCAGCAGTGGGGAATATTGGACAATGGGGGAAACCCTGATCCAGCGACGCCGCGTGAGTGAAGAAAGTATTTCGGGTATGTAAAAGCTCTATCAGCAGGGGAAGATAATGACAGTACCTGACTAAGAAGCCCCGGCTAACTACGTGCCAGCAGCCGCGGTAA

+

:::::::::::::::::::::::::::::::::::::::::::::::::::::::::::::::::::::::::::::::::::::::::::::::::::::::::::::::::::::::::::::::::::::::::::::::::::::::::::::::::::::::::::::::::::::::::::::::::::::::::::::::::::::::::::::::::::::::::::::::::::::::::::::::::::::::::::::::::::::::::::::::::::::::::::::::::::::::::::::::::::::::::::::::::::::::::::::::::::::::::::::::::::::::::::::::::::::::::::::::::::::::::::::::::::::::::::::::::::::::::::::::::::::::::::::::::::::::::::::::::::::::::::

@D0950.29_101122

GATGAACGCTAGCGACAGGCCTAACACATGCAAGTCGAGGGGCAGCGAGAGAGTAGCAATACTTTTGTCGGCGACCGGCGCACGGGTGAGTAACACGTATGCAACCTGCCCATAACAGGGGGATAATCGGAAGAAATTCCGTCTAATACCGCGTAACCCTGCATTATCTCATGATAACGCAGGTAAAGAAGCAATTCGGTTATGGATGGGCATGCGGAACATTAGGTAGTTGGTGAGGTAACGGCTCACCAAGCCGACGATGTATAGGGGTTCTGAGAGGAAGGTCCCCCACACTGGTACTGAGACACGGACCAGACTCCTACGGGAGGCAGCAGTGAGGAATATTGGTCAATGGGCGCGAGCCTGAACCAGCCAAGTCGCGTGAAGGATGAAGGTTCTATGGATTGTAAACTTCTTTTGTCAGGGGAACAAAGAGCTCACGTGTGAGCAGATGAGTGTACCTGAAG

+

:::::::::::::::::::::::::::::::::::::::::::::::::::::::::::::::::::::::::::::::::::::::::::::::::::::::::::::::::::::::::::::::::::::::::::::::::::::::::::::::::::::::::::::::::::::::::::::::::::::::::::::::::::::::::::::::::::::::::::::::::::::::::::::::::::::::::::::::::::::::::::::::::::::::::::::::::::::::::::::::::::::::::::::::::::::::::::::::::::::::::::::::::::::::::::::::::::::::::::::::::::::::::::::::::::::::::::::::::::::::::::::::::::::::::::::::::::

@D0950.29_101165

GATGAACGCTAGCGACAGGCCTAACACATGCAAGTCGAGGGGTAGCACAAGGAAGCTTGCTTCTGAGGTGACGACCGGCGCACGGGTGAGTAACGCGTATGCAACCTACCTGTAAGAGTGGGATAGCCTCTCGAAAGAGAGATTAATACCGCATAATACCATTTCACTGCATGGTGAGATGGTTAAAGATTTATTGCTTACAGATGGGCATGCGTAACATTAGCTAGTTGGTGAGGTAACGGCTCACCAAGGCAACGATGTTTAGGGGTTCTGAGAGGAAGGTCCCCCACACTGGTACTGAGACACGGACCAGACTCCTACGGGAGGCAGCAGTGAGGAATATTGGTCAATGGACGAGAGTCTGAACCAGCCAAGTCGCGTGAAGGATGAAGGTCTTATGGATTGTAAACTTCTTTTATACGGGAATAAAAAATGCCACGTGTGGCATATTGCATGTACCGTATGAATAAGGATCGGCTAACTCCGTGCCAGC

+

:::::::::::::::::::::::::::::::::::::::::::::::::::::::::::::::::::::::::::::::::::::::::::::::::::::::::::::::::::::::::::::::::::::::::::::::::::::::::::::::::::::::::::::::::::::::::::::::::::::::::::::::::::::::::::::::::::::::::::::::::::::::::::::::::::::::::::::::::::::::::::::::::::::::::::::::::::::::::::::::::::::::::::::::::::::::::::::::::::::::::::::::::::::::::::::::::::::::::::::::::::::::::::::::::::::::::::::::::::::::::::::::::::::::::::::::::::::::::::::::::::::::::::::

@D0950.29_101187

ATTGAACGCTGGCGGCATGCCTTACACATGCAAGTCGAACGGTAACAGGTCTTCGGATGCTGACGAGTGGCGAACGGGTGAGTAATACATCGGAACGTGCCCGATCGTGGGGGATAACGAAGCGAAAGCTTTGCTAATACCGCATACGATCTACGGATGAAAGCAGGGGACCGCAAAGGCCTTGCGCGAACGGAGCGGCCGATGGCAGATTAGGTAGTTGGTGGGATAAAAAGCTTACCAAAGCCGACGATCTGTAGCTGGTCTGAGAGGACGACCAGCCACACTGGGACTGAGACACGGTCCAAAACTCCTACGGGAGGCAGCAGTGAGGAAATATTGGTCAAATGGACGTAAAGTCTGAACCAGCCAAAGTAGCGTGAAGGATGAAAGGCTCTATGGGTCGTAAAACTTCTTTTATAAAAGGAAATAAAAAGTATGCCACGTGTGGTGTTTTGTAATGTACTTTATG

+

:::::::::::::::::::::::::::::::::::::::::::::::::::::::::::::::::::::::::::::::::::::::::::::::::::::::::::::::::::::::::::::::::::::::::::::::::::::::::::::::::::::::::::::::::::::::::::::::::::::::::::::::::::::::::::::::::::::::::::::::::::::::::::::::::::::::::::::::::::::::::::::::::::::::::::::::::::::::::::::::::::::::::::::::::::::::::::::::::::::::::::::::::::::::::::::::::::::::::::::::::::::::::::::::::::::::::::::::::::::::::::::::::::::::::::::::::::::

@D0950.29_101193

GATGAACGCTGGCGGCGTGCTTAACACATGCAAGTCGAACGAAGCACTTTGAAGAGCTTGCTCTTTAAAGTGACTGAGTGGCGGACGGGTGAGTAACGCGTGGGTAACCTGCCTCATACAGGGGGATAACAGTTAGAAATGACTGCTAACACCGCATAACCCGCTAGTGTCGCATGACACAGACGGAAAATATTTATAGGTATGAGATGGGCCCGCGTCTGATTACGCTAGTTGGTGGGGTAACGGCCTACCAAGGCAACGATCAGTAGCCGACTTGAGAGAGTGATCGGCCACATTGGGACTGAGACACGGCCCAAACTCCTACGGGAGGCAGCAGTGGGGAATATTGGACAATGGGGGAAACCCTGATCCAGCGACGCCGCGTGAGTGAAAGAAGTATTTTCGGGTATGTAAAGCTCTATCAGCAGGGGAAGATAATGACAGTACCTGACTAAGAAGCCCCGGCTAACTACGTGCCAGCAGCCGCGGTAA

+

::::::::::::::::::::::::::::::::::::::::::::::::::::::::::::::::::::::::::::::::::::::::::::::::::::::::::::::::::::::::::::::::::::::::::::::::::::::::::::::::::::::::::::::::::::::::::::::::::::::::::::::::::::::::::::::::::::::::::::::::::::::::::::::::::::::::::::::::::::::::::::::::::::::::::::::::::::::::::::::::::::::::::::::::::::::::::::::::::::::::::::::::::::::::::::::::::::::::::::::::::::::::::::::::::::::::::::::::::::::::::::::::::::::::::::::::::::::::::::::::::::::::::::

@D0950.29_101259

GATGAACGCTAGCTACAGGCTTAACACATGCAAGTCGAGGGGTAGCATGAAACTTAGCAATAAGTTTTGATGACGACCGGCGCACGGGTGAGTAACACGTATCCAACCTGCCTTTTACTCATGGATAGCCTTCTGAAAAGAAGATTAATACATGATGGTATTCAGAGTTTTCATGGACACTGAATTAAAGATTTTATCGGTAAGAGATGGGGATGCGTTCCATTAGATAGTAGGCGGGGTAACGGCCCACCTAGTCAACGATGGATAGGGGTTCTGAGAGGAAGGTCCCCCACATTGGAACTGAGACACGGTCCAAACGTCCTACGGGAGGCAGCAGTGAGGAATATTGGTCAATGGACGTAAGTCTGAACCAGCCAAGTAGCGTGAAGGATGAAGGCTCTATGGGTCGTAAACTTCTTTTTATAAAAGGAATAAAGTATGCCACGTGTGGTGTTTTTGTATGTAACTTTATGAATAAGGATCGGCTAACTCCGTGCCAGCAGCCGC

+

:::::::::::::::::::::::::::::::::::::::::::::::::::::::::::::::::::::::::::::::::::::::::::::::::::::::::::::::::::::::::::::::::::::::::::::::::::::::::::::::::::::::::::::::::::::::::::::::::::::::::::::::::::::::::::::::::::::::::::::::::::::::::::::::::::::::::::::::::::::::::::::::::::::::::::::::::::::::::::::::::::::::::::::::::::::::::::::::::::::::::::::::::::::::::::::::::::::::::::::::::::::::::::::::::::::::::::::::::::::::::::::::::::::::::::::::::::::::::::::::::::::::::::::::::::::::::::

@D0950.29_101390

GATGAACGCTGGCGGCGTGCTTAACACATGCAAGTCGAACGAAGCAGCTTTCTTGCTTGCAAGAAAGCTGACTTAGTGGCGGACGGGTGAGTAACGCGTGGGTAACCTGCCCTCATACAGGGGGATAACGAGTTGGAAACGACTGCTAAGACCGCATAACCCGCTAGTGTCGCATGACACGGACGGAAAATATTTATAGGTATGAGATGGGCCCGCGTCTGATTAGCTAGTTGGTAAGGTAACGGCTTACCAAGGCGACGAGTCAGTAGCCGACTTGAGAGAGTGATCGGCCACATTGGGACTGAGACACGGCCCAAACTCCTACGGGAGGCAGCAGTGGGGAATATTGGACAATGGGGGAAACCCTGATCCAGCGACGCCGCGTGAGTGAAGAAGTATTTCGGTATGTAAAAGCTCTATCAGCAGGGAAGATAATGACAGTACCTGACT

+

::::::::::::::::::::::::::::::::::::::::::::::::::::::::::::::::::::::::::::::::::::::::::::::::::::::::::::::::::::::::::::::::::::::::::::::::::::::::::::::::::::::::::::::::::::::::::::::::::::::::::::::::::::::::::::::::::::::::::::::::::::::::::::::::::::::::::::::::::::::::::::::::::::::::::::::::::::::::::::::::::::::::::::::::::::::::::::::::::::::::::::::::::::::::::::::::::::::::::::::::::::::::::::::::::::::::::::::::::::::::::::::::::

@D0950.29_101418

GATGAACGCTAGCTACAGGCTTAACACATGCAAGTCGAGGGGTAGCATGAAACTTAGCAATAAGTTTTGATGACGACCGGCGCACGGGTGAGTAACACGTATCCAACCTGCCTTTTACTCATGGATAGCCTTCTGAAAAGAAGATTAATACATGATGGTATTCAGAGTTTTCATGGACACTGAATTAAAGATTTTATCGGTAAGAGATGGGGATGCGTTCCATTAGATAGTAGGCGGGGTAACGGCCCACCTAGTCAACATGGATAGGGGTTCTGAGAGGAAGGTCCCCCACATTGGAACTGAGACACGGTCCAAACGTCCTACGGGAGGCCAGCAGTGAGGAATATTGGTCAATGGACGTAAGTCTGAACCAGCCAAGTAGCGTGAA

+

::::::::::::::::::::::::::::::::::::::::::::::::::::::::::::::::::::::::::::::::::::::::::::::::::::::::::::::::::::::::::::::::::::::::::::::::::::::::::::::::::::::::::::::::::::::::::::::::::::::::::::::::::::::::::::::::::::::::::::::::::::::::::::::::::::::::::::::::::::::::::::::::::::::::::::::::::::::::::::::::::::::::::::::::::::::::::::::::::::::::::::::::::::::::::::::::::::

@D0950.29_101434

GATGAACGCTAGCGACAGGCCTAACACATGCAAGTCGAGGGGTAGCACAAGGTAGTAATACTGAGGTGACGACCGGCGCACGGGTGAGTAACGCGTATGCAACCTACCTGTAAGAGTGGGATAGCCTCTCGAAAGAGAGATTAATACCGCATAATACCATTTCACTGCATGGTGAGATGGTTAAAGATTTATTGCTTACAGATGGGCATGCGTAACATTAGCTAGTTGGTGAGGTAACGGCTCACCAAGGCAACGATGTTTAGGGGTTCTGAGAGGAAGGTCCCCCACACTGGTACTGAGACACGGACCAGACTCCTACGGGAGGCAGCAGTGAGGAATATTGGTCAATGGACGAGAGTCTGAACCAGCCAAGTCGCGTGAAGGATGAAGGTCTTATGGATTGTAAACTTCTTTTATACGGGAATAAAAATGCCACGTGTGGCATATTGCATGTACCGTATGAATAAGGATCGGCTAACTCCGTGCCAGCAGCCGCGGTAA

+

:::::::::::::::::::::::::::::::::::::::::::::::::::::::::::::::::::::::::::::::::::::::::::::::::::::::::::::::::::::::::::::::::::::::::::::::::::::::::::::::::::::::::::::::::::::::::::::::::::::::::::::::::::::::::::::::::::::::::::::::::::::::::::::::::::::::::::::::::::::::::::::::::::::::::::::::::::::::::::::::::::::::::::::::::::::::::::::::::::::::::::::::::::::::::::::::::::::::::::::::::::::::::::::::::::::::::::::::::::::::::::::::::::::::::::::::::::::::::::::::::::::::::::::::::::::

@D0950.29_101470

ATTGAACGCTGGCGGCAGGCCTAACACATGCAAGTCGAACGGTAGCACAGAGAGCTTGCTCTCGGGTGACGAGTGGCGGACGGGTGAGTAATGTCTGGGAAACTGCCCGATGGAGGGGGATAACTACTGGAAACGGTAGCTAATACCGCATAACGTCGCAAGACCAAAGAGGGGACCTTCGGGCCTCTTGCCATCGGATGTGCCCAGATGGGATTAGCTAGTAGGTGGGGTAACGGCTCACCTAGGCGACGATCCCTAGCTGGTCTGAGAGGATGACCAGCCACACTGGAACTGAGACACGGTCCAGACTCCTACGGGAGGCAGCAGTGGGGAATATTGCACAATGGGGAAACCCTGATGCAGCAACGCCGCGTGAGTGATGAA

+

::::::::::::::::::::::::::::::::::::::::::::::::::::::::::::::::::::::::::::::::::::::::::::::::::::::::::::::::::::::::::::::::::::::::::::::::::::::::::::::::::::::::::::::::::::::::::::::::::::::::::::::::::::::::::::::::::::::::::::::::::::::::::::::::::::::::::::::::::::::::::::::::::::::::::::::::::::::::::::::::::::::::::::::::::::::::::::::::::::::::::::::::::::::::::::::::

@D0950.29_1015

GACGAACGCTGGCGGCGTGCTTAACACATGCAAGTCGAACGGAGTGTCTTATTTCGGTAGGATGCTTAGTGGCGAACGGGTGAGTAACGCGTAAACAATCTGCCCTTTAGCTGGGGACAACAGATCGAAAGGTCTGCTAATACCGAATGATGAAAGTTGAACGCATGTTCGACTATTTAAAGATGGCTTAAGAGCTTGCTCTGCTATCACTAAAGGATGAGTTTGCGTCTGATTAGCTAGTTGGTAAGGTAATGGCTTACCAAGGCAACGATCAGTAGCCGGTCTGAGAGGATGAACGGCCACACTGGGACTGAGACACGGCCCAGACTCCTACGGGAGGCAGCAGTGGGGAATCTTCCGCAAGTGGACGAAATGTCTGACGGAGCAACGCCGCGTGAGTGAAGAAGGTTTTCCGGATCGTAAAATCTCTGTTGTTTAGGACGAATGTTGTGATTGTGAATAATGGTTGCAAATGACGGTACTGAACGAGAAAGCCACGGCTAACTACGTGCCAGCAGCCGC

+

::::::::::::::::::::::::::::::::::::::::::::::::::::::::::::::::::::::::::::::::::::::::::::::::::::::::::::::::::::::::::::::::::::::::::::::::::::::::::::::::::::::::::::::::::::::::::::::::::::::::::::::::::::::::::::::::::::::::::::::::::::::::::::::::::::::::::::::::::::::::::::::::::::::::::::::::::::::::::::::::::::::::::::::::::::::::::::::::::::::::::::::::::::::::::::::::::::::::::::::::::::::::::::::::::::::::::::::::::::::::::::::::::::::::::::::::::::::::::::::::::::::::::::::::::::::::::::::::::::::::::

@D0950.29_101589

GATGAACGCTAGCTACAGGCTTAACACATGCAAGTCGAGGGGTAGCATGAAACTTAGCAATAAGTTTTGATGACGACCGGCGCACGGGTGAGTAACACGTATCCAACCTGCCTTTTACTCATGGATAGCCTTCTGAAAAGAAGATTAATACATGATGGTATTCAGAGTTTTCATGGACACTGAATTAAAGATTTTATCGGTAAGAGATGGGGATGCGTTCCATTAGATAGTAGGCTGGGGTAACGGCCCACCTAGTCGAACGATCGGATAGGGGTTCTGAGAGGAAGGTCCCCCACATTGGAACTGAGACACGGTCCAAACGTCCTACGGGAGGCGAGCAGTGAGGAATATTGGTCCAATGGACGTAAGTCTGAACCAGCCAAGTAGCGTGAAGGATGAAGGCTCTATGGGTCGTAAACTTCTTTTAT

+

::::::::::::::::::::::::::::::::::::::::::::::::::::::::::::::::::::::::::::::::::::::::::::::::::::::::::::::::::::::::::::::::::::::::::::::::::::::::::::::::::::::::::::::::::::::::::::::::::::::::::::::::::::::::::::::::::::::::::::::::::::::::::::::::::::::::::::::::::::::::::::::::::::::::::::::::::::::::::::::::::::::::::::::::::::::::::::::::::::::::::::::::::::::::::::::::::::::::::::::::::::::::::::::::::::::::::::

@D0950.29_10160

GAAAACGCAAGAGACGTGCCTAACACATGCGAATTGAATAGTTCTCGACAACTATAGTGAATGGGTGAGTAATATATGAAAATTTAAGTTTAGATTAGAAGTATCTCTAGGAAACTAGTGAAAATCTCTGATATGCGCATATGTGCTTTAAAAAGTTACAACTGTCTAAGAAAAAGTTCATATCTGATTAGCTATTTAGTACTGTAAAAGAGTATTAAAGCGAAGATCAGTAGCTGCCTTGAGAGGGGGATCAGCCACATTGGGATTGAAATACCGCCCAAACTCTTACGGGAGGCTGCAGTGGGGAATCTTCTGCAATGAGCGCAAGCTTGACAGAGCGTCGTCACGTGGAGGAAGACAGCTTAATAAAGTTGTAAACTTCTTTTATTTAATAAAGAATACTGACGTTTAAGTAAATAAAGTATCGGCAAA

+

::::::::::::::::::::::::::::::::::::::::::::::::::::::::::::::::::::::::::::::::::::::::::::::::::::::::::::::::::::::::::::::::::::::::::::::::::::::::::::::::::::::::::::::::::::::::::::::::::::::::::::::::::::::::::::::::::::::::::::::::::::::::::::::::::::::::::::::::::::::::::::::::::::::::::::::::::::::::::::::::::::::::::::::::::::::::::::::::::::::::::::::::::::::::::::::::::::::::::::::::::::::::::::::::::::::::::::::::

@D0950.29_101644

GATGAACGCTGGCGGCGTGCTTAACACATGCAAGTCGAACGAAGCACTTTGAAGAGCTTGCTCTTTAAAGTGACTGAGTGGCGGACGGGTGAGTAACGCGTGGGTAACCTGCCTCATACAGGGGGATAACAGTTAGAAATGACTGCTAACACCGCATAACCCGCTAGTGTCGCATGACACAGACGGAAAATATTTATAGGTATGAGATGGGCCCGCGTCTGATTAGCTAGTTGGTGGGGTAACGGCCTACCAAGGCAACGATCAGTAGCCGACTTGAGAGAGTGATCGGCCACATTGGGACTGAGACACGGCCCAAACTCCTACGGGAGGCAGCAGTGGGGAATATTGGACAATGGGGGAAACCCTGATCCAGCGACGCCGCGTGAGTGAAGAAGTATTTCGGTATGTAAAGCTCTATCAGCAGGGAAGATAATGACAGTACCTGACTAAGAAGCCCCGGCTAACTACGTGCCAGCAGCCGCGGTAA

+

:::::::::::::::::::::::::::::::::::::::::::::::::::::::::::::::::::::::::::::::::::::::::::::::::::::::::::::::::::::::::::::::::::::::::::::::::::::::::::::::::::::::::::::::::::::::::::::::::::::::::::::::::::::::::::::::::::::::::::::::::::::::::::::::::::::::::::::::::::::::::::::::::::::::::::::::::::::::::::::::::::::::::::::::::::::::::::::::::::::::::::::::::::::::::::::::::::::::::::::::::::::::::::::::::::::::::::::::::::::::::::::::::::::::::::::::::::::::::::::::::::::::

@D0950.29_101668

GATGAACGCTAGCTACAGGCTTAACACATGCAAGTCGAGGGGTAGCATGAAACTTAGCAATAAGTTTTGATGACGACCGGCGCACGGGTGAGTAACACGTATCCAACCTGCCTTTTACTCATGGATAGCCTTCTGAAAAGAAGATTAATACATGATGGTATTCAGAGTTTTCATGGACACTGAATTAAAGATTTTATCGGTAAGAGATGGGGATGCGTTCCATTAGATAGTAGGCGGGGTAACGGCCCACCTAGTCAACGATGGATAGGGGTTCTGAGAGGAAGGTCCCCCACATTGGAACTGAGACACGGTCCAAACGTCCTACGGGAGGCAGCAGTGAGGAATATTGGTCAATGGACGTAAGTCTGAACCAGCCAAGTAGCGTGAAGGATGAAGGCTCTATGGGTCGTAAACTTC

+

:::::::::::::::::::::::::::::::::::::::::::::::::::::::::::::::::::::::::::::::::::::::::::::::::::::::::::::::::::::::::::::::::::::::::::::::::::::::::::::::::::::::::::::::::::::::::::::::::::::::::::::::::::::::::::::::::::::::::::::::::::::::::::::::::::::::::::::::::::::::::::::::::::::::::::::::::::::::::::::::::::::::::::::::::::::::::::::::::::::::::::::::::::::::::::::::::::::::::::::::::::::::::::::::::

@D0950.29_101680

GATGAACGCTAGCTACAGGCTTAACACATGCAAGTCGAGGGGTAGCATGAAACTTAGCAATAAGTTTTGATGACGACCGGCGCACGGGTGAGTAACACGTATCCAACCTGCCTTTTACTCATGGATAGCCTTCTGAAAAGAAGATTAATACATGATGGTATTCAGAGTTTTCATGGACACTGAATTAAAGATTTATCGGTAAGAGATGGGGATGCGTTCCATTAGATAGTAGGCGGGGTAACGGCCCACCTAGTCAACGATGGATAGGGGTTCTGAGAGGAAGGTCCCCCACATTGGAACTGAGACACGGTCCAAACGTCCTACGGGAGGCAGCAGTGAGGAATATTGGTCAATGGACGTAAGTCTGAACCAGCCAAGTAGCGTGAAGGATGAAGGCTCTATGGGTCGTAAACTTCTTTTATAAAAGGAATAAAGTATGCCACGTGTGGTGTTTTTGTATGTACTTTATG

+

::::::::::::::::::::::::::::::::::::::::::::::::::::::::::::::::::::::::::::::::::::::::::::::::::::::::::::::::::::::::::::::::::::::::::::::::::::::::::::::::::::::::::::::::::::::::::::::::::::::::::::::::::::::::::::::::::::::::::::::::::::::::::::::::::::::::::::::::::::::::::::::::::::::::::::::::::::::::::::::::::::::::::::::::::::::::::::::::::::::::::::::::::::::::::::::::::::::::::::::::::::::::::::::::::::::::::::::::::::::::::::::::::::::::::::::::::::::

@D0950.29_101683

GATGAACGCTGGCGGCGTGCTTAACACATGCAAGTCGAACGAAGCACTTTGAAGAGCTTGCTCTTTAAAGTGACTGAGTGGCGGACGGGTGAGTAACGCGTGGGTAACCTGCCTCATACAGGGGGATAACAGTTAGAAATGGACTGCTAACACCGCATAACCCGCTAGTGTCGCATGACACAGACGGAAAATATTTATAGGTATGAGATGGGCCCGCGTCTGATTACGCTAGTTGGTGGGGTAACGGCCTACCAAGGCAACGATCAGTAGCCGACTTGAGAGAGTGATCGGCCACATTGGGACTGAGACACGGCCCAAACTCCTACGGGAGGCAGCAGTGGGGAATATTGGACAATGGGGGAAACCCTGATCCAGCGACGCCGCGTGAGTGAAGAAGTATTTCGGTATGTAAAGCTCTATCAGCAGGGAAGATAATGACAGTACCTGACTAAGAAGCCCCGGCTAACTACGTGCCAGCAGCCGCGGTAA

+

:::::::::::::::::::::::::::::::::::::::::::::::::::::::::::::::::::::::::::::::::::::::::::::::::::::::::::::::::::::::::::::::::::::::::::::::::::::::::::::::::::::::::::::::::::::::::::::::::::::::::::::::::::::::::::::::::::::::::::::::::::::::::::::::::::::::::::::::::::::::::::::::::::::::::::::::::::::::::::::::::::::::::::::::::::::::::::::::::::::::::::::::::::::::::::::::::::::::::::::::::::::::::::::::::::::::::::::::::::::::::::::::::::::::::::::::::::::::::::::::::::::::::

@D0950.29_1017

GATGAACGCTAGCGACAGGCCTAACACATGCAAGTCGAGGGGTAGCACAAGGTAGCAATACTGAGGTGACGACCGGCGCACGGGTGAGTAACGCGTATGCAACCTACCTGTAAGAGTGGGATAGCCTCTCGAAAGAGAGATTAATACCGCATAATACCATTTACTGCATGGTGAGATGGTTAAAGATTTGTTGCTTACAGATGGGCATGCGTAACATTAGCTAGTTGGTGAGGTAACGGCTCACCAAGGCAACGATGTTTAGGGGTTCTGAGAGGAAGGTCCCCCACACTGGTACTGAGACACGGACCAGACTCCTACGGGAGGCAGCAGTGAGGAATATTGGTCAATGGACGAGAGTCTGAACCAGCCAAGTCGCGTGAAGGATGAAGGTCTTATGGATTGTAAACTTCTTTTATACGGGAATAAAAAATGCCACGTGTGGCATATTGCATGTACCGTATGAATAAGGATCGGCTAACTCCGTGCCAGCAGCCGCGGTAA

+

:::::::::::::::::::::::::::::::::::::::::::::::::::::::::::::::::::::::::::::::::::::::::::::::::::::::::::::::::::::::::::::::::::::::::::::::::::::::::::::::::::::::::::::::::::::::::::::::::::::::::::::::::::::::::::::::::::::::::::::::::::::::::::::::::::::::::::::::::::::::::::::::::::::::::::::::::::::::::::::::::::::::::::::::::::::::::::::::::::::::::::::::::::::::::::::::::::::::::::::::::::::::::::::::::::::::::::::::::::::::::::::::::::::::::::::::::::::::::::::::::::::::::::::::::::::

@D0950.29_10173

GATGAACGCTAGCGACAGGCCTAACACATGCAAGTCGAGGGGTAGCACAAGGTAGTAATACTGAGGTGACGACCGGCGCACGGGTGAGTAACGCGTATGCAACCTACCTGTAAGAGTGGGATAGCCTCTCGAAAGAGAGATTAATACCGCATAATACCATTTCACTGCATGGTGAGATGGTTAAAGATTTATTGCTTACAGATGGGCATGCGTAACATTAGCTAGTTGGTGAGGTAACGGCTCACCAAGGCAACGATGTTTAGGGGTTCTGAGAGGAAGGTCCCCCACACTGGTACTGAGACACGGACCAGACTCCTACGGGAGGCAGCAGTGAGGAATATTGGTCAATGGACGAGAGTCTGAACCAGCCAAGTCGCGTGAAGGATGAAGGTCTTATGGATTGTAAACTTCTTTTATACGGGAATAAAAAGAGCCACGTGTGGCTTATTGCATGTACCGTATGAATAAGGATCGGCTAACTCCGTGCCAGCAGCCGCGGTAA

+

::::::::::::::::::::::::::::::::::::::::::::::::::::::::::::::::::::::::::::::::::::::::::::::::::::::::::::::::::::::::::::::::::::::::::::::::::::::::::::::::::::::::::::::::::::::::::::::::::::::::::::::::::::::::::::::::::::::::::::::::::::::::::::::::::::::::::::::::::::::::::::::::::::::::::::::::::::::::::::::::::::::::::::::::::::::::::::::::::::::::::::::::::::::::::::::::::::::::::::::::::::::::::::::::::::::::::::::::::::::::::::::::::::::::::::::::::::::::::::::::::::::::::::::::::::::

@D0950.29_101759

GATGAACGCTAGCGACAGGCCTAACACATGCAAGTCGAGGGGTAGCACAAGGTAGTAATACTGAGGTGACGACCGGCGCACGGGTGAGTAACGCGTATGCAACCTACCTGTAAGAGTGGGATAGCCTCTCGAAAGAGAGATTAATACCGCATAATACCATTTCACTGCATGGTGAGATGGTTAAAGATTTATTGCTTACAGATGGGCATGCGTAACATTAGCTAGTTGGTGAGGTAACGGCTCACCAAGGCAACGATGTTTAGGGGTTCTGAGAGGAAGGTCCCCCACACTGGTACTGAGACACGGACCAGACTCCTACGGGAGGCAGCAGTGAGGAATATTGGTCAATGGACGAGAGTCTGAACCAGCCAAGTCGCGTGAAGGATGAAGGTCTTATGGATTGTAAACTTCTTTTATACGGGAATAAAAAATGCCACGTGTGGCATATTGCATGTACCGTATGAATAAGGATCGGCTAACTCCGTGCCAGCAGCCGCGGTAA

+

::::::::::::::::::::::::::::::::::::::::::::::::::::::::::::::::::::::::::::::::::::::::::::::::::::::::::::::::::::::::::::::::::::::::::::::::::::::::::::::::::::::::::::::::::::::::::::::::::::::::::::::::::::::::::::::::::::::::::::::::::::::::::::::::::::::::::::::::::::::::::::::::::::::::::::::::::::::::::::::::::::::::::::::::::::::::::::::::::::::::::::::::::::::::::::::::::::::::::::::::::::::::::::::::::::::::::::::::::::::::::::::::::::::::::::::::::::::::::::::::::::::::::::::::::::::

@D0950.29_101778

GATGAACGCTAGCGACAGGCCTAACACATGCAAGTCGAGGGGTAGCACAAGGTAGCAATACTGAGGTGACGACCGGCGCACGGGTGAGTAACGCGTATGCAACCTACCTGTAAGAGTGGGATAGCCTCTCGAAAGAGAGATTAATACCGCATAATACCATTTCACTGCATGGTGAGATGGTTAAAGATTTATTGCTTACAGATGGGCATGCGTAACATTAGCTAGTTGGTGAGGTAACGGCTCACCAAGGCAACGATGTTTAGGGGTTCTGAGAGGAAGGTCCCCCACACTGGTACTGAGACACGGACCAGACTCCTACGGGAGGCAGCAGTGAGGAATATTGGTCAATGGACGAGAGTCTGAACCAGCCAAGTCGCGTGAAGGATGAAGGTCTTATGGATTGTAAACTTCTTTTATACGGGAATAAAAATGCCACGTGTGGCATATTGCATGTACCGTATGAATAAGGATCGGCTAACTCCGTGCCAGCAGCCGCGGTAA

+

:::::::::::::::::::::::::::::::::::::::::::::::::::::::::::::::::::::::::::::::::::::::::::::::::::::::::::::::::::::::::::::::::::::::::::::::::::::::::::::::::::::::::::::::::::::::::::::::::::::::::::::::::::::::::::::::::::::::::::::::::::::::::::::::::::::::::::::::::::::::::::::::::::::::::::::::::::::::::::::::::::::::::::::::::::::::::::::::::::::::::::::::::::::::::::::::::::::::::::::::::::::::::::::::::::::::::::::::::::::::::::::::::::::::::::::::::::::::::::::::::::::::::::::::::::::

@D0950.29_101815

ATTGAACGCTGGCGGCAGGCCTAACACATGCAAGTCGAACGGTAGCACAGAGAGCTTGCTCTTGGGTGACGAGTGGCGGACGGGTGAGTAATGTCTGGGAAACTGCCCGATGGAGGGGGCATAACTACTGGAAACGGTAGCTAATACCGCATAATGTCGCAAGACCAAAGAGGGGGACCTTCGGGCCTCTTGCCATCGGATGTGCCCAGGATGGGATTAGCTAGTAGGTGGGGTAATGGCTCACCTAGGCGACGATCCCTAGCTGGTCTGAGAGGATGACCAGCCACACTGGAACTGAGACACGGTCCAGACTCCTACGGGAGGCAGCAGTGGGGAATATTGCACAATGGGCGCAAGCCTGATGCAGCCATGCCGCGTGTATGAAGAAGGCCTTCGGGTTGTAAAGTACTTTCAGCGAGGAGAAGGTGTTGAGGTTTAATTAACCTCAGCAATTGACGTTACTCGCAG

+

::::::::::::::::::::::::::::::::::::::::::::::::::::::::::::::::::::::::::::::::::::::::::::::::::::::::::::::::::::::::::::::::::::::::::::::::::::::::::::::::::::::::::::::::::::::::::::::::::::::::::::::::::::::::::::::::::::::::::::::::::::::::::::::::::::::::::::::::::::::::::::::::::::::::::::::::::::::::::::::::::::::::::::::::::::::::::::::::::::::::::::::::::::::::::::::::::::::::::::::::::::::::::::::::::::::::::::::::::::::::::::::::::::::::::::::::::::

@D0950.29_101826

GATGAACGCTGGCGGCGTGCTTAACACATGCAAGTCGAACGAAGCACTTTGAAGAGCTTGCTCTTTAAAGTGACTGAGTGGCGGACGGGTGAGTAACGCGTGGGTAACCTGCCTCATACAGGGGGATAACAGTTAGAAATGGACTGCTAACACCGCATAACCCGCTAGCATCGCATGATGCGGACGGAAAAATATTTATAGGTATGAGATGGGCCCGCGTCTGATTAGCTAGTTGGTGGGGTAACAGCCTACCAAGGCAACGATCAGTAGCCGACTTGAGAGAGTGATCGGCCACATTGGGACTGAGACACGGCCCAAACTCCTACGGGAGGCAGCAGTGGGGAATATTGGACAATGGGGGAAACCCTGATCCAGCGACGCCGCGTGAGTGAAGAAGTATTTCGGTATGTAAAGCTCTATCAGCAGGGAAGATAATGACAGTACCTGACTAAGAAGCCCCCGGCTAACTACGTGCCAGCAGCCGC

+

:::::::::::::::::::::::::::::::::::::::::::::::::::::::::::::::::::::::::::::::::::::::::::::::::::::::::::::::::::::::::::::::::::::::::::::::::::::::::::::::::::::::::::::::::::::::::::::::::::::::::::::::::::::::::::::::::::::::::::::::::::::::::::::::::::::::::::::::::::::::::::::::::::::::::::::::::::::::::::::::::::::::::::::::::::::::::::::::::::::::::::::::::::::::::::::::::::::::::::::::::::::::::::::::::::::::::::::::::::::::::::::::::::::::::::::::::::::::::::::::::::::

@D0950.29_101906

GATGAACGCTAGCGACAGGCCTAACACATGCAAGTCGAGGGGCAGCGAGAGAGTAGCAATACTTTTGTCGGCGACCGGCGCACGGGTGAGTAACACGTATGCAACCTGCCCATAACAGGGGGATAATCGGAAGAAATTCCGTCTAATACCGCGTAACCCTGCATTATCTCATGATAACGCAGGTAAAGAAGCAATTCGGTTATGGATGGGCATGCGGAACATTAGGTAGTTGGTGAGGTAACGGCTCACCAAGCCGACGATGTATAGGGGTTCTGAGAGGAAGGTCCCCCACACTGGTACTGAGACACGGACCAGACTCCTACGGGAGGCAGCAGTGAGGAATATTGGTCAATGGGCGCGAGCCTGAACCAGCCAAGTCGCGTGAAGGATGAAGGTTCTATGGGTCGTAAACTTCTTTTATAAAGGAATAAAGTATGCCACGTGTGGTGTTTTGTATGTACTTTATGAATAAGGATCGGCTAACTCCGTGCCAGCAGCCGCGGTAA

+

::::::::::::::::::::::::::::::::::::::::::::::::::::::::::::::::::::::::::::::::::::::::::::::::::::::::::::::::::::::::::::::::::::::::::::::::::::::::::::::::::::::::::::::::::::::::::::::::::::::::::::::::::::::::::::::::::::::::::::::::::::::::::::::::::::::::::::::::::::::::::::::::::::::::::::::::::::::::::::::::::::::::::::::::::::::::::::::::::::::::::::::::::::::::::::::::::::::::::::::::::::::::::::::::::::::::::::::::::::::::::::::::::::::::::::::::::::::::::::::::::::::::::::::::::::::::::

@D0950.29_101939

GATGAACGCTAGCGACAGGCCTAACACATGCAAGTCGAGGGGTAGCACAAGGTAGTAATACTGAGGTGACGACCGGCGCACGGGTGAGTAACGCGTATGCAACCTACCTGTAAGAGTGGGATAGCCTCTCGAAAGAGAGATTAATACCGCATAATACCATTTCACTGCATGGTGAGATGGTTAAAGATTTATTGCTTACAGATGGGCATGCGTAACATTAGCTAGTTGGTGAGGTAACGGCTCACCAAGGCAACGATGTTTAGGGGTTCTGAGAGGAAGGTCCCCCACACTGGTACTGAGACACGGACCAGACTCCTACGGGAGGCAGCAGTGAGGAATATTGGTCAATGGACGAGAGTCTGAACCAGCCAAGTCGCGTGAAGGATGAAGGTCTTATGGATTGTAAACTTCTTTTATACGGGAATAAAAATGCCACGTGTGGCATATTGCATGTACCGTATGAATAAGGATCGGCTAACTCCGTGCCAGCAGGCGCGGTAA

+

:::::::::::::::::::::::::::::::::::::::::::::::::::::::::::::::::::::::::::::::::::::::::::::::::::::::::::::::::::::::::::::::::::::::::::::::::::::::::::::::::::::::::::::::::::::::::::::::::::::::::::::::::::::::::::::::::::::::::::::::::::::::::::::::::::::::::::::::::::::::::::::::::::::::::::::::::::::::::::::::::::::::::::::::::::::::::::::::::::::::::::::::::::::::::::::::::::::::::::::::::::::::::::::::::::::::::::::::::::::::::::::::::::::::::::::::::::::::::::::::::::::::::::::::::::::

@D0950.29_101944

ATTGAACGCTGGCGGCAGGCCTAACACATGCAAGTCGAACGGTAGCACAGAGGAGCTTGCTCCTTGGGTGACGAGTGGCGGACGGGTGAGTAATGTCTGGGAAACTGCCCGATGGAGGGGGATAACTACTGGAAACGGTAGCTAATACCGCATAATGTCGCAAGACCAAAGAGGGGGACCTTCGGGCCTCTTGCCATCGGATGTGCCCAGATGGGATTAGCTAGTAGGTGGGGTAACGGCTCACCTAGGCCGACGGATCCCTAGCTGGTCTGAAGAGGATGACCAGCCACGACTGGAACTGAGACACGGTCCAGACTCCTACGGGAGGCAGCAGTGGGAATATTGCACAATGGGCGCAAGCCTGATGCAGCCATGCCGCGTTGTATGAAGAAGGCCTTCGGGTTGTAAAGTACTTTCAGCGAGGAGGAAGGCGTTGTGGTTAATAACCGCAACGATTGACGTTACTCGCAGAAGAAGCACCGGCTAACTCCGTGCCAGCAGCCGCGGTAA

+

::::::::::::::::::::::::::::::::::::::::::::::::::::::::::::::::::::::::::::::::::::::::::::::::::::::::::::::::::::::::::::::::::::::::::::::::::::::::::::::::::::::::::::::::::::::::::::::::::::::::::::::::::::::::::::::::::::::::::::::::::::::::::::::::::::::::::::::::::::::::::::::::::::::::::::::::::::::::::::::::::::::::::::::::::::::::::::::::::::::::::::::::::::::::::::::::::::::::::::::::::::::::::::::::::::::::::::::::::::::::::::::::::::::::::::::::::::::::::::::::::::::::::::::::::::::::::::::

@D0950.29_101985

ATTGAACGCTGGCGGCATGCCTTACACATGCAAGTCGAACGGTAACAGGTCTTCGGATGCTGACGAGTGGCGAACGGGTGAGTAATACATCGGAACGTGCCCGATCGTGGGGGATAACGAAGCGAAAGCTTTGCTAATACCGCATACGATCTACGGATGAAAGCAGGGGACCGCAAGGCCTTGCGCGAACGGAGCGGCCGATGGCAGATTAGGTAGTTGGTGGGATAAAATGCTTACCAAGCCGACGATCTGTAGCTGGTCTGAGAGGACGACCAGCCACACTGGGACTGAGACACGGCCCAGACTCCTACGGGAGGCAGCAGTGGGGAATTTTGGACAATGGGCGAAAGCCTGATCCAGCCATGCCGCGTGCAGGATGAAGGCCTTCGGGTTGTAACTGCTTTTGTACGGAACGAAAAGACTCTGGTTATACCTGGGGTCCATGACGGTACCGTAAGAATAAGCACCGGCTAACTACGTGCCAGCAGCCGCGGTAA

+

:::::::::::::::::::::::::::::::::::::::::::::::::::::::::::::::::::::::::::::::::::::::::::::::::::::::::::::::::::::::::::::::::::::::::::::::::::::::::::::::::::::::::::::::::::::::::::::::::::::::::::::::::::::::::::::::::::::::::::::::::::::::::::::::::::::::::::::::::::::::::::::::::::::::::::::::::::::::::::::::::::::::::::::::::::::::::::::::::::::::::::::::::::::::::::::::::::::::::::::::::::::::::::::::::::::::::::::::::::::::::::::::::::::::::::::::::::::::::::::::::::::::::::::::::

@D0950.29_1020

GATGAACGCTAGCGACAGGCCTAACACATGCAAGTCGAGGGGTAGCACAAGGAAGCTTGCTTCTGAGGTGACGACCGGCGCACGGGTGAGTAACGCGTATGCAACCTGCCTATAAGAAGGGGATAGCCTCTCGAAAGAGAGATTAATACCGTATAACACTATGAAGCCGCATGGTTTTATAGTTAAAGATTTATTGCTTATAGATGGGCATGCGTAACATTAGCTAGTTGGTGAGGTAACGGCTCACCAAGGCAACGATGTTTAGGGGTTTCTGAGAGGAAGGTCCCCCACACTGGTACTGAGACACGGACCAGACTCCTACGGGAGGCAGCAGTGAGGAATATTGGGTCAATGGACGAGAGTCTGAACCAGCCAAGTCGCGTGAAGGATGAAGGTCTTATGGATTGTAAAC

+

::::::::::::::::::::::::::::::::::::::::::::::::::::::::::::::::::::::::::::::::::::::::::::::::::::::::::::::::::::::::::::::::::::::::::::::::::::::::::::::::::::::::::::::::::::::::::::::::::::::::::::::::::::::::::::::::::::::::::::::::::::::::::::::::::::::::::::::::::::::::::::::::::::::::::::::::::::::::::::::::::::::::::::::::::::::::::::::::::::::::::::::::::::::::::::::::::::::::::::::::::::::::::::

@D0950.29_1021

GATGAACGCTGGCGGCGTGCTTAACACATGCAAGTCGAACGAAGCAGCTTTCTTGCTTGCAAGAAAGCTGACTTAGTGGCGGACGGGTGAGTAACGCGTGGGTAACCTGCCTCATACAGGGGGATAACAGTTGGAAACGACTGCTAAGACCGCATAACCCGCTAGTGTCGCATGACACGGACGGAAAATATTTATAGGTATGAGATGGGCCCGCCGTCTGATTAGCTAGTTGGTAAGGTAACGGCTTACCAAGGCGACGATCAGTAGCCGACTTGAGAGAGTGATCGGCCACATTGGGACTGAGACACGGCCCAAACTCCTACGGGAGGCAGCAGTGGGAATATTGGACAATGGGGGAAACCCTGATCCAGCGACGCCGCGTGAGTGAAGAAGTATTTCGGTATGTAAAGCTCTATCAGCAGGGAAGATAATGACAGTACCTGACTAAGAAGCCCCCGGGCTAACTACGTGCCAGCAGCCGC

+

::::::::::::::::::::::::::::::::::::::::::::::::::::::::::::::::::::::::::::::::::::::::::::::::::::::::::::::::::::::::::::::::::::::::::::::::::::::::::::::::::::::::::::::::::::::::::::::::::::::::::::::::::::::::::::::::::::::::::::::::::::::::::::::::::::::::::::::::::::::::::::::::::::::::::::::::::::::::::::::::::::::::::::::::::::::::::::::::::::::::::::::::::::::::::::::::::::::::::::::::::::::::::::::::::::::::::::::::::::::::::::::::::::::::::::::::::::::::::::::::::

@D0950.29_10210

GATGAACGCTAGCTACAGGCTTAACACATGCAAGTCGAGGGGTAGCATGAAACTTAGCAATAAGTTTTGATGACGACCGGCGCACGGGTGAGTAACACGTATCCAACCTGCCTTTTACTCATGGATAGCCTTCTGAAAAGAAGATTAATACATGATGGTATTCAGAGTTTTCATGGACACTGAATTTAAAGATTTTATCGGTAAGAGATGGGGATGCGTTCCATTAGATAGTAGGCGGGGTAACGGCCCACCTAGTCAACGATGGATAGGGGTTCTGAGAGGAAGGTCCCCACATTGAACTGAGACACGGTCCAAACGTCCTACGGGAGGCAGCAGTGAGAATATTGGTCAATGACGTAAGTCTGAACCAGCCAAGTAGCGTGAAGGATGAAGGCTCTAT

+

::::::::::::::::::::::::::::::::::::::::::::::::::::::::::::::::::::::::::::::::::::::::::::::::::::::::::::::::::::::::::::::::::::::::::::::::::::::::::::::::::::::::::::::::::::::::::::::::::::::::::::::::::::::::::::::::::::::::::::::::::::::::::::::::::::::::::::::::::::::::::::::::::::::::::::::::::::::::::::::::::::::::::::::::::::::::::::::::::::::::::::::::::::::::::::::::::::::::::::::::

@D0950.29_102136

GATGAACGCTGGCGGCGTGCTTAACACATGCAAGTCGAACGAAGCACTTAACTTAGAATCTTCGGATGAAGAGTTTTGTGACTTAGTGGCGGACGGGTGAGTAACGCGTGGGTAACCTGCCTTATACTGGGGGATAACAGTTAGAAATGACTGCTAATACCGCATAAGCGCACAGTATCACATGATACAGTGCGAAAAACTCCGGTGGTATAAGATGGACCCGCGTCTGATTAGCTAGTTGGTAAGGTAACGGCTTACCAAGGCGACGATCAGTAGCCGACTTGAGAGAGTGATCGGCCACATTGGGACTGAGACACGGCCCAAACTCCTACGGGAGGCAGCAGTGGGGAATATTGCACAAGTGGGCGAAAGCCTGATGCAGCGACGCCGCGTGAAGGATGAAGTATTTCGGTACGTAAAACTTCTATCAGCAAGGAAGATAATGACGGTACTTGACTAAGAAGCCCCGGCTAACTACGTGCCAGCAGCCGCGGTAA

+

:::::::::::::::::::::::::::::::::::::::::::::::::::::::::::::::::::::::::::::::::::::::::::::::::::::::::::::::::::::::::::::::::::::::::::::::::::::::::::::::::::::::::::::::::::::::::::::::::::::::::::::::::::::::::::::::::::::::::::::::::::::::::::::::::::::::::::::::::::::::::::::::::::::::::::::::::::::::::::::::::::::::::::::::::::::::::::::::::::::::::::::::::::::::::::::::::::::::::::::::::::::::::::::::::::::::::::::::::::::::::::::::::::::::::::::::::::::::::::::::::::::::::::::::::

@D0950.29_102188

ATTGAACGCTGGCGGCAGGCCTAACACATGCAAGTCGAACGGTAGCACAGAGGAGCTTGCTCCTTGGGTGACGAGTGGCGGACGGGTGAGTAATGTCTGGGAAACTGCCCGATGGAGGGGGATAACTACTGGAAACGGTAGCTAATACCGCATAACGTCGCAAGACCAAAGAGGGGGACTTCGGGCCTCTTGCCATCGGATGTCCCAGATGGGATTAGCTAGTAGGTGGGGTAACGGCTCACCTAGGCGACGATCCTAGCTGGTCTGAGAGGATGACCAGCCACACTGGAACTGAGACACGGTCCAGACTCCTACGGGAGGCAGCAGTGGGGAATATTGCACAATGGGCGCAAGCCTGATGCAGCCATGCCGCGTGTATGAAGAAGGCCTTCGGGTTGTAAAGTACTTTCAGCGAGGAGGAAGGTGTTGTGGTTAATAACCGCAGCAATTGACGTTACTCGCAGAAGAAGCACCGGCTAACTCCGTGCCAGCAGCCGCGGTAA

+

:::::::::::::::::::::::::::::::::::::::::::::::::::::::::::::::::::::::::::::::::::::::::::::::::::::::::::::::::::::::::::::::::::::::::::::::::::::::::::::::::::::::::::::::::::::::::::::::::::::::::::::::::::::::::::::::::::::::::::::::::::::::::::::::::::::::::::::::::::::::::::::::::::::::::::::::::::::::::::::::::::::::::::::::::::::::::::::::::::::::::::::::::::::::::::::::::::::::::::::::::::::::::::::::::::::::::::::::::::::::::::::::::::::::::::::::::::::::::::::::::::::::::::::::::::::::

@D0950.29_102202

GATGAACGCTAGCTACAGGCTTAACACATGCAAGTCGAGGGGTAGCATGAAACTTAGCAATAAGTTTTGATGACGACCGGCGCACGGGTGAGTAACACGTATCCAACCTGCCTTTTACTCATGGATAGCCTTCTGAAAAGAAGATTAATACATGATGGTATTCAGAGTTTTCATGGACACTGAATTAAAGATTTATCGGTAAGAGATGGGGATGCGTTCCATTAGATAGTAGGCGGGGTAACGGCCCACCTAGTCAACGATGGATAGGGGTTCTGAGAGGAAGGTCCCCCACATTGGAACTGAGACACGGTCCAAACTCCTACGGGAGGCAGCAGTGAGGAATATTGGTCAATGGACGTAAGTCTGAACCAGCCAAGTAGCGTGAAGGATGAAGGCTCTATGGGTCGTAAACTTCTTTTATAAAAGGAATAAAGTATGCCACGTGTGGTGTTTTTGTATGTACTTTATGAATAAGGATCGGCTAACTCCGTGCCAGCAG

+

:::::::::::::::::::::::::::::::::::::::::::::::::::::::::::::::::::::::::::::::::::::::::::::::::::::::::::::::::::::::::::::::::::::::::::::::::::::::::::::::::::::::::::::::::::::::::::::::::::::::::::::::::::::::::::::::::::::::::::::::::::::::::::::::::::::::::::::::::::::::::::::::::::::::::::::::::::::::::::::::::::::::::::::::::::::::::::::::::::::::::::::::::::::::::::::::::::::::::::::::::::::::::::::::::::::::::::::::::::::::::::::::::::::::::::::::::::::::::::::::::::::::::::::::::::

@D0950.29_102205

GACGAACGCTGGCGGCGTGCCTAACACATGCAAGTCGAGCGATGAAGTTCTTCGGAACGGATTAGCGGCGGACGGGTGAGTAACACGTGGGTAACCTGCCTCATAGAGGGGAATAGCCTTTCGAAAGGAAGATTAATACCGCATAAGATTGTAATACCGCATGGTATAGCAATTAAAGGAGTAATCCGCTATGAGATGGACCCGCGTCGCATTAGCTAGTTGGTGAGGTAACGGCTCACCAAGGCGACGATGCGTAGCCGACCTGAGAGGGTGATCGGCCACATTGGGACTGAGGACACGGCCCAGACTCCTACGGGAGGCAGCAGTGGGGAATTGTTCGCAATGGGCGCAAGCCTGACGACGCAACGCCGCGTGGGGGATGAAGGTCTTCGGTTGTAAACCCCTGTTGGGTGGACGAACACCGCCGAGGTGAATATCCTTGGCGGCTGACGGTACCACCTGAGGAAGCCCCGGCTAACTCTGTGCCAGC

+

::::::::::::::::::::::::::::::::::::::::::::::::::::::::::::::::::::::::::::::::::::::::::::::::::::::::::::::::::::::::::::::::::::::::::::::::::::::::::::::::::::::::::::::::::::::::::::::::::::::::::::::::::::::::::::::::::::::::::::::::::::::::::::::::::::::::::::::::::::::::::::::::::::::::::::::::::::::::::::::::::::::::::::::::::::::::::::::::::::::::::::::::::::::::::::::::::::::::::::::::::::::::::::::::::::::::::::::::::::::::::::::::::::::::::::::::::::::::::::::::::::::::::

@D0950.29_102236

GATGAACGCTAGCTACAGGCTTAACACATGCAAGTCGAGGGGTAGCATGAAACTTAGCAATAAGTTTTGATGACGACCGGCGCACGGGTGAGTAACACGTATCCAACCTGCCTTTTACTCATGGATAGCCTTCTGAAAAGAAGATTAATACATGATGGTATTCAGAGTTTTCATGGACACTGAATTAAAGATTTTATCGGTAAGAGATGGGGATGCGTTCCATTAGATAGTAGGCTGGGGTAACGGCCCACCTAGTCGAACGATGGATAGGGGTTCTGAGAGGAAGGTCCCCCACATTGGAACTGAGACACGGTCCAAACTCCTACGGGAGGCAGCAGTGAGGAATATTGGTCAATGGACGTAAGTCTGAACCAGCCAAGTAGCGTGAAGGATGAAGGCTCTATGGGTCGTAAACTTCTTTTTATAAAAGGAATAAAGTATGCCACGTGTGGTGTTTTTGTATGTACTTTATGAATAAGGATCGGCTAACTCCGTGCCAGCAGCCGC

+

:::::::::::::::::::::::::::::::::::::::::::::::::::::::::::::::::::::::::::::::::::::::::::::::::::::::::::::::::::::::::::::::::::::::::::::::::::::::::::::::::::::::::::::::::::::::::::::::::::::::::::::::::::::::::::::::::::::::::::::::::::::::::::::::::::::::::::::::::::::::::::::::::::::::::::::::::::::::::::::::::::::::::::::::::::::::::::::::::::::::::::::::::::::::::::::::::::::::::::::::::::::::::::::::::::::::::::::::::::::::::::::::::::::::::::::::::::::::::::::::::::::::::::::::::::::::::::

@D0950.29_102303

GATGAACGCTGGCGGCGTGCTTAACACATGCAAGTCGAACGAAGCACTTAAGGAGCTTGCTCCAAAAGTGACTGAGTGGCGGACGGGTGAGTAACGCGTGGGTAACCTGCCTTACACTGGGGGATAACAGTTGGAAACGACTGCTAATACCGCATAAGCGCACAGTATTGCATGATACAGTGTGAAAAACTCCGGTGGTGTAAGATGGACCCGCGTCTGATTAGCTAGTTGGTGAGGTAATGGCTCACCAAGGCAACGATCAGTAGCCGGCTTGAGAGAGTGAACGGCCACATTGGGACTGAGACACGGCCCAAACTCCTACGGGAGGCAGCAGTGGGGAATATTGCACAATGGGGGAAACCCTGATGCAGCAACGCCGCGTGAGTGAAGAAGTATTTGCGGTATGTAAAGCTCTATCAGCAGGGAAGATAATGACGGTACCTGACTAAGAAGCCCCCGGCTAACTACGTGCCAGCAGCCGC

+

::::::::::::::::::::::::::::::::::::::::::::::::::::::::::::::::::::::::::::::::::::::::::::::::::::::::::::::::::::::::::::::::::::::::::::::::::::::::::::::::::::::::::::::::::::::::::::::::::::::::::::::::::::::::::::::::::::::::::::::::::::::::::::::::::::::::::::::::::::::::::::::::::::::::::::::::::::::::::::::::::::::::::::::::::::::::::::::::::::::::::::::::::::::::::::::::::::::::::::::::::::::::::::::::::::::::::::::::::::::::::::::::::::::::::::::::::::::::::::::::::

@D0950.29_10233

GATGAACGCTAGCGACAGGCCTAACACATGCAAGTCGAGGGGTAGCACAAGGTAGTAATACTGAGGTGACGACCGGCGCACGGGTGAGTAACGCGTATGCAACCTACCTGTAAGAGTGGGATAGCCTCTCGAAAGAGAGATTAATACCGCATAATACCATTTCACTGCATGGTGAGATGGTTAAAGATTTATTGCTTACAGATGGGCATGCGTAACATTAGCTAGTTGGTGAGGTAACGGCTCACCAAGGCAACGATGTTTAGGGGTTCTGAGAGGAAGGTCCCCCACACTGGTACTGAGACACGGACCAGACTCCTACGGGAGGCAGCAGTGAGGAATATTGGTCAATGGACGAGAGTCTGAACCAGCCAAGTCGCGTGAAGGATGAAGGTCTTATGGATTGTAAACTTCTTTTATACGGGAATAAAAATGCCACGTGTGGCATATTGCATGTACCGTATGAATAAGGATCGGCTAACTCCGTGCCAGCAGCCGCGGTAA

+

:::::::::::::::::::::::::::::::::::::::::::::::::::::::::::::::::::::::::::::::::::::::::::::::::::::::::::::::::::::::::::::::::::::::::::::::::::::::::::::::::::::::::::::::::::::::::::::::::::::::::::::::::::::::::::::::::::::::::::::::::::::::::::::::::::::::::::::::::::::::::::::::::::::::::::::::::::::::::::::::::::::::::::::::::::::::::::::::::::::::::::::::::::::::::::::::::::::::::::::::::::::::::::::::::::::::::::::::::::::::::::::::::::::::::::::::::::::::::::::::::::::::::::::::::::::

@D0950.29_102361

GATGAACGCTAGCGACAGGCTTAACACATGCAAGTCGAGGGGCAGCACAAGGTAGCAATACTGAGGTGGCGACCGGCGCACGGGTGAGTAACGCGTATGCAACCTACCTCTTAGCGGGGGATAACCCGGCGAAAGTCGGACTAATACCGCATAATACTCTTTCTCCGCATGGAGGGAGATTTAAAGATTAATTGCTAAGAGATGGGCATGCGTTCCATTAGGTAGTTGGTAGAGGTAACGGCCTACCAAGCCATCGATGGATAGGGGTTCTGAGAGGAAGGTCCCCCACACTGGTACTGAGACACGGACCAGACTCCTACGGGAGGCAGCAGTGAGGAATATTGGTCAATGGACGAGAGTCTGAACCAGCCAAGTCGCGTGAAGGAAGAAGGTTCTATGGATTGTAAAACTTCTTTTATAGGGGAATAAAAGTGAGAGACGTGTCTCTTTTTTGTATGTACCCTA

+

:::::::::::::::::::::::::::::::::::::::::::::::::::::::::::::::::::::::::::::::::::::::::::::::::::::::::::::::::::::::::::::::::::::::::::::::::::::::::::::::::::::::::::::::::::::::::::::::::::::::::::::::::::::::::::::::::::::::::::::::::::::::::::::::::::::::::::::::::::::::::::::::::::::::::::::::::::::::::::::::::::::::::::::::::::::::::::::::::::::::::::::::::::::::::::::::::::::::::::::::::::::::::::::::::::::::::::::::::::::::::::::::::::::::::::::::::

@D0950.29_102395

GATGAACGCTAGCGACAGGCCTAACACATGCAAGTCGAGGGGTAGCACAAGGAAGCTTGCTTCTGAGGTGACGACCGGCGCACGGGTGAGTAACGCGTATGCAACCTGCCTATAAGAAGGGGATAGCCTCTCGAAAGAGAGATTAATACCGTATAACACTATGAAGCCGCATGGTTTTATAGTTAAAGATTTATTGCTTATAGATGGGCATGCGTAACATTAGCTTGTTGGTGAGGTAACGGCTCACCAAGGCAACGATGTTTAGGGGTTCTGAGAGGAAGGTCCCCCACACTGGTACTGAGACACGGACCAGACTCCTACGGGAGGCAGCAGTGAGGAATATTGGTCAATGGACGAGAGTCTGAACCAGCCAAGTCGCGTGAAGGATGAAGGTCTTATGGATTGTAAACTTCTTTTATAC

+

:::::::::::::::::::::::::::::::::::::::::::::::::::::::::::::::::::::::::::::::::::::::::::::::::::::::::::::::::::::::::::::::::::::::::::::::::::::::::::::::::::::::::::::::::::::::::::::::::::::::::::::::::::::::::::::::::::::::::::::::::::::::::::::::::::::::::::::::::::::::::::::::::::::::::::::::::::::::::::::::::::::::::::::::::::::::::::::::::::::::::::::::::::::::::::::::::::::::::::::::::::::::::::::::::::::

@D0950.29_102401

GATGAACGCTGGCGGCGTGCTTAACACATGCAAGTCGAACGAAGCAACTTTCTTGCTTGCAAGAAAGTTGACTGAGTGGCGGACGGGTGAGTAACGCGTGGGTAACCTGCCTCATACAGGGGGATAACAGTTGGAAACGACTGCTAAGACCGCATAACCCGCTAGTGTCGCATGACACGGACGGAAAAATATTTATAGGTATGAGATGGGCCCGCGTCTGATTAGCTAGTTGGTAAGGTAACGGCTTACCAAGGCGACGATCAGTAGCCGACTTGAGAGAGTGATCGGCCACATTGGGACTGAGACACGGCCCAAACTCCTACGGGAGGCAGCAGTGGGGAATATTGGACAATGGGGGAAACCCTGATCCAGCGACGCCGCGTGAGTGAAGAAGTATTTCGGTATGTAAAGCTCTATCAGCAGGGGAAGATAAATGACAGTACCTGACTAAGAAGCCCCGGCTAACTACGTGCCAGCAGCCGCGGTAA

+

::::::::::::::::::::::::::::::::::::::::::::::::::::::::::::::::::::::::::::::::::::::::::::::::::::::::::::::::::::::::::::::::::::::::::::::::::::::::::::::::::::::::::::::::::::::::::::::::::::::::::::::::::::::::::::::::::::::::::::::::::::::::::::::::::::::::::::::::::::::::::::::::::::::::::::::::::::::::::::::::::::::::::::::::::::::::::::::::::::::::::::::::::::::::::::::::::::::::::::::::::::::::::::::::::::::::::::::::::::::::::::::::::::::::::::::::::::::::::::::::::::::::

@D0950.29_102449

GATGAACGCTAGCGACAGGCCTAACACATGCAAGTCGAGGGGTAGCACAAGGAAGCTTGCTTCTGAGGTGACGACCGGCGCACGGGTGAGTAACGCGTATGCAACCTACCTGTAAGAGTGGGATAGCCTCTCGAAAGAGAGATTAATACCGCATAATACCATTTCACTGCATGGTGAGATGGTTAAAGATTTATTGCTTACAGATGGGCATGCGTAACATTAGCTAGTTGGTGAGGTAACGGCTCACCAAGGCAACGATGTTTAGGGGTTCTGAGAGGAAGGTCCCCCACACTGGTACTGAGACACGGACCAGACTCCTACGGGAGGCAGCAGTGAGGAATATTGGTCAATGGACGAGAGTCTGAACCAGCCAAGTCGCGTGAAGGATGAAGGTCTTATGGATTGTAAACTTCTTTTATACGGGAATAAAAATGCCACGTGTGGCATATTGCATGTACCGTATGAATAAGGATCGGCTAACTCCGTGCCAGCAGCCGCGGTAA

+

:::::::::::::::::::::::::::::::::::::::::::::::::::::::::::::::::::::::::::::::::::::::::::::::::::::::::::::::::::::::::::::::::::::::::::::::::::::::::::::::::::::::::::::::::::::::::::::::::::::::::::::::::::::::::::::::::::::::::::::::::::::::::::::::::::::::::::::::::::::::::::::::::::::::::::::::::::::::::::::::::::::::::::::::::::::::::::::::::::::::::::::::::::::::::::::::::::::::::::::::::::::::::::::::::::::::::::::::::::::::::::::::::::::::::::::::::::::::::::::::::::::::::::::::::::::::

@D0950.29_102508

GATGAACGCTAGCGACAGGCCTAACACATGCAAGTCGAGGGGTAGCACAAGGTAGCAATACTGAGGTGACGACCGGCGCACGGGTGAGTAACGCGTATGCAACCTACCTGTAAGAGTGGGATAGCCTCTCGAAAGAGAGATTAATACCGCATAATACCATTTATCTGCATGGTGAGATGGTTAAAGATTTATTGCTTACAGATGGGCATGCGTAACATTAGCTAGTTGGTGAGGTAACGGCTCACCAAGGCAACGATGTTTAGGGGTTCTGAGAGGAAGGTCCCCCACACTGGTACTGAGACACGGACCAGACTCCTACGGGAGGCAGCAGTGAGGAATATTGGTCAATGGACGAGAGTCTGAACCAGCCAAGTCGCGTGAAGGATGAAGGTCTTATGGATTGTAAACTTCTTTTATACGGGAATAAAAAATGCCACGTGTGGCATATTGCATGTACCGTATGAATAAGGATCGGCT

+

:::::::::::::::::::::::::::::::::::::::::::::::::::::::::::::::::::::::::::::::::::::::::::::::::::::::::::::::::::::::::::::::::::::::::::::::::::::::::::::::::::::::::::::::::::::::::::::::::::::::::::::::::::::::::::::::::::::::::::::::::::::::::::::::::::::::::::::::::::::::::::::::::::::::::::::::::::::::::::::::::::::::::::::::::::::::::::::::::::::::::::::::::::::::::::::::::::::::::::::::::::::::::::::::::::::::::::::::::::::::::::::::::::::::::::::::::::::::::::::

@D0950.29_102528

GATGAACGCTAGCTACAGGCTTAACACATGCAAGTCGAGGGGTAGCATGAAACTTAGCAATAAGTTTTGATGACGACCGGCGCACGGGTGAGTAACACGTATCCAACCTGCCTTTTACTCATGGATAGCCTTCTGAAAAGAAGATTAATACATGATGGTATTCAGAGTTTTCATGGACACTGAATTAAAGATTTTATCGGTAAAGAGATGGGGATGCGTTCCATTAGATAGTAGGCGGGGTAACGGCCCACCTAGTCAACGATGGATAGGGGTTCTGAGAGGAAGGTCCCCACATTGGAACTGAGACACGGTCCAAACTCCTACGGGAGGCAGCAGTGAGGAATATTGGTCAATGGACGTAAGTCTGAACCAGCCAAGTAGCGTGAAGG

+

:::::::::::::::::::::::::::::::::::::::::::::::::::::::::::::::::::::::::::::::::::::::::::::::::::::::::::::::::::::::::::::::::::::::::::::::::::::::::::::::::::::::::::::::::::::::::::::::::::::::::::::::::::::::::::::::::::::::::::::::::::::::::::::::::::::::::::::::::::::::::::::::::::::::::::::::::::::::::::::::::::::::::::::::::::::::::::::::::::::::::::::::::::::::::::::::::::::

@D0950.29_102567

GATGAACGCTGGCGGCGTGCTTAACACATGCAAGTCGAACGAAGCAACTATCTTGCTTGCAAGAGAGTTGACTTAGTGGCGGACGGGTGAGTAACGCGTGGGTAACCTGCCTCATACAGGGGGATAACAGTTGGAAACGACTGCTAAGACCGCATAACCCGCTAGTGTCGCATGACACGGACGGAAAATATTTTATAGGTATGAGATGGGCCCGCGTCTGATTAGCTAGTTGGTAAGGTAACGGCTTACCAAGGCGACGATCAGTAGCCGACTTGAGAGAGTGATCGGCCACATTGGGACTGAGACACGGCCCAAACTCCTACGGGAGGCAGCAGTGGGGAATATTGGACAATGGGGGAAACCCTGATCCAGCGACGCCGCGTGAGTGAAGAAGTATTTCGGTATGTAAAGCTCTATCAGCAGGGAAGATAATGACAGTACCTGACTAAGAAGCCCCCGGCTAACTACGTGCCAGCAG

+

::::::::::::::::::::::::::::::::::::::::::::::::::::::::::::::::::::::::::::::::::::::::::::::::::::::::::::::::::::::::::::::::::::::::::::::::::::::::::::::::::::::::::::::::::::::::::::::::::::::::::::::::::::::::::::::::::::::::::::::::::::::::::::::::::::::::::::::::::::::::::::::::::::::::::::::::::::::::::::::::::::::::::::::::::::::::::::::::::::::::::::::::::::::::::::::::::::::::::::::::::::::::::::::::::::::::::::::::::::::::::::::::::::::::::::::::::::::::::::::

@D0950.29_102581

GATGAACGCTAGCGACAGGCCTAACACATGCAAGTCGAGGGGTAGCACAAGGTAGCAATACTGAGGTGACGACCGGCGCACGGGTGAGTAACGCGTATGCAACCTACCTGTAAGAGTGGGATAGCCTCTCGAAAGAGAGATTAATACCGCATAATACCATTTCACTGCATGGTGAGATGGTTAAAGATTTATTGCTTACAGATGGGCATGCGTAACATTAGCTTGTTGGTGAGGTAACGGCTCACCAAGGCAACGATGTTTAGGGGTTCTGAGAGGAAGGTCCCCCACACTGGTACTGAGACACGGACCAGACTCCTACGGGAGGCAGCAGTGAGGAATATTGGTCAATGGACGAGAGTCTGAACCAGCCAAGTCGCGTGAAGGATGAAGGTCTTATGGATTGTAAACTTCTTTTATACGGGAATAAAAATGCCACGTGTGGCATATTGCATGTACCGTATGAATAAGGATCGGCTAACTCCGTGCCAGCAGCCGCGGTAA

+

:::::::::::::::::::::::::::::::::::::::::::::::::::::::::::::::::::::::::::::::::::::::::::::::::::::::::::::::::::::::::::::::::::::::::::::::::::::::::::::::::::::::::::::::::::::::::::::::::::::::::::::::::::::::::::::::::::::::::::::::::::::::::::::::::::::::::::::::::::::::::::::::::::::::::::::::::::::::::::::::::::::::::::::::::::::::::::::::::::::::::::::::::::::::::::::::::::::::::::::::::::::::::::::::::::::::::::::::::::::::::::::::::::::::::::::::::::::::::::::::::::::::::::::::::::::

@D0950.29_102635

GATGAACGCTGGCGGCGTGCTTAACACATGCAAGTCGAACGAAGCACTTTGAAGAGCTTGCTCTTTAAAGTGACTGAGTGGCGGACGGGTGAGTAACGCGTGGGTAACCTGCCTCATACAGGGGGATAACAGTTAGAAATGACTGCTAACACCGCATAACCCGCTAGTGTCGCATGACACAGACGGAAAATATTTATAGGTATGAGATGGGCCCGCGTCTGATTAGCTAGTTGGTGGGTAACGGCCTACCAAGGCAACGATCAGTAGCCGACTTGAGAGAGTGATCGGCCACATTGGGACTGAGACACGGCCCAAACTCCTACGGGAGGCAGCAGTGGGGAATATTGGACAATGGGGGAAACCCTGATCCAGCGACGCCGCGTGAGTGAAGAAGTATTTCGGTATGTAAAAGCTCTATCAGCAGGGGAAGATAATGACAGTACCTGACTAAGAAGCCCCGGCTAACTACGTGCCAGCAGCCGCGGTAA

+

::::::::::::::::::::::::::::::::::::::::::::::::::::::::::::::::::::::::::::::::::::::::::::::::::::::::::::::::::::::::::::::::::::::::::::::::::::::::::::::::::::::::::::::::::::::::::::::::::::::::::::::::::::::::::::::::::::::::::::::::::::::::::::::::::::::::::::::::::::::::::::::::::::::::::::::::::::::::::::::::::::::::::::::::::::::::::::::::::::::::::::::::::::::::::::::::::::::::::::::::::::::::::::::::::::::::::::::::::::::::::::::::::::::::::::::::::::::::::::::::::::::::

@D0950.29_102652

GACGAACGCTGGCGGCGTGCTTAACACATGCAAGTCGAACGGAATTACGCTTAACACCGATCTTTCGCTAAGAAGGCTCGATGTTAAGCCGCGATGCGGTGAATGAACGAAGTGAATTCACCACGCGTTTTAAAAGTGCTAACACTATCGAGGGATGGGTGTTAAGCGTAATTTAGTGGCGAACGGGTGAGTAACGCGTAAACAATCTGCCCTTTAGATGGGGACAACAGATCGAAAGGTCTGCTAATACCGAATGTTGTAAGTAGAACGCATGTTTTACTTATTAAAGATGGCCTCTACATGTAAGCTATCGCTAAAGGATGAGTTTGCGTCTGATTAGCTAGTTGGTAAAGGTAATGGCTTACCAAGGCAACGATCAGTAGCCGGGTCTGAGAGGATGAACGGCCACACTGGGACTGAGACACGGCCCAGACTCCTACGGGAGGCAGCAGTGGGGGAATCTTCCGCAATGGACGAAAGTCTGACGGAGCAACGCCGCGTGAGTGAAGAAGGTTTTCGGATCGTAAAAGCTCTGTTATTAGGGGACGAATGTTAATATTGTGAATAATGATAATAAATGACGGT

+

:::::::::::::::::::::::::::::::::::::::::::::::::::::::::::::::::::::::::::::::::::::::::::::::::::::::::::::::::::::::::::::::::::::::::::::::::::::::::::::::::::::::::::::::::::::::::::::::::::::::::::::::::::::::::::::::::::::::::::::::::::::::::::::::::::::::::::::::::::::::::::::::::::::::::::::::::::::::::::::::::::::::::::::::::::::::::::::::::::::::::::::::::::::::::::::::::::::::::::::::::::::::::::::::::::::::::::::::::::::::::::::::::::::::::::::::::::::::::::::::::::::::::::::::::::::::::::::::::::::::::::::::::::::::::::::::::::::::::::::::::::::::::::::::::::::::::

@D0950.29_102707

GATGAACGCTGGCGGCGTGCTTAACACATGCAAGTCGAACGAAGCAGCTTTCTTGCTTGCAAGAAAGCTGACTTAGTGGCGGACGGGTGAGTAACGCGTGGGTAACCTGCCTCATACAGGGGGATAACAGTTGGAAACGACTGCTAAGACCGCATAACCCGCTAGTGTCGCATGACACGGACGGAAAAATATTTTATAGGTATGAGATGGGGCCCGCGTCTGATTAGCTAGTTGGTAAGGTAACGGCTTACCAAGGCGACGATCAGTAGCCGACTTGAGAGAGTGATCGGCCACATTGGGACTGAGACACGGCCCAAACTCCTACGGGAGGCAGCAGTGGGGAATATTGGACAATGGGGGAAACCCTGATCCAGCGACGCCGCGTGAGTGAAGAAGTATTTCGGTATGTAAAGCTCTATCAGCAGGGAAGATAATGACAGTACCTGACT

+

:::::::::::::::::::::::::::::::::::::::::::::::::::::::::::::::::::::::::::::::::::::::::::::::::::::::::::::::::::::::::::::::::::::::::::::::::::::::::::::::::::::::::::::::::::::::::::::::::::::::::::::::::::::::::::::::::::::::::::::::::::::::::::::::::::::::::::::::::::::::::::::::::::::::::::::::::::::::::::::::::::::::::::::::::::::::::::::::::::::::::::::::::::::::::::::::::::::::::::::::::::::::::::::::::::::::::::::::::::::::::::::::::

@D0950.29_10275

GATGAACGCTAGCGACAGGCCTAACACATGCAAGTCGAGGGGTAGCACAAGGTAGCAATACTGAGGTGACGACCGGCGCACGGGTGAGTAACGCGTATGCAACCTACCTGTAAGAGTGGGATAGCCTCTCGAAAGAGAGATTAATACCGCATAATACCATTTCACTGCATGGTGAGATGGTTAAAGATTTATTGCTTACAGATGGGCATGCGTAACATTAGCTTGTTGGTGAGGTAACGGCTCACCAAGGCAACGATGTTTAGGGGTTCTGAGAGGAAGGTCCCCCACACTGGGACTGAGACACGGCCCAAACTCCTACGGGAGGCAGCAGTGGGGAATATTGGACAATGGGGGAAACCCTGATCCAGCGACGCCGCGTGAGTGAAGAAGTATTTCGGTATGTAAAGCTCTATCAGCAGGGAAGATAATGACAGTACCTGACTAAGAAGCCCCCGGCTAACTACGTGCCAGCAGCCG

+

:::::::::::::::::::::::::::::::::::::::::::::::::::::::::::::::::::::::::::::::::::::::::::::::::::::::::::::::::::::::::::::::::::::::::::::::::::::::::::::::::::::::::::::::::::::::::::::::::::::::::::::::::::::::::::::::::::::::::::::::::::::::::::::::::::::::::::::::::::::::::::::::::::::::::::::::::::::::::::::::::::::::::::::::::::::::::::::::::::::::::::::::::::::::::::::::::::::::::::::::::::::::::::::::::::::::::::::::::::::::::::::::::::::::::::::::::::::::::::::

@D0950.29_102754

GATGAACGCTAGCTACAGGCTTAACACATGCAAGTCGAGGGGTAGCATGAAACTTAGCAATAAGTTTTGATGACGACCGGCGCACGGGTGAGTAACACGTATCCAACCTGCCTTTTACTCATGGATAGCCTTCTGAAAAGAAGATTAATACATGATGGTATTCAGAGTTTTCATGGACACTGAATTAAAGATTTTATCGGTAAGAGATGGGGATGCGTTCCATTAGATAGTAGGCGGGGTAACGGCCCACCTGAGTCAACGATGGATAGGGGTTCTGAGAGGAAGGTCCCCCACATTGGAACTGAGACACGGTCCAAACTCCTACGGGAGGCAGCAGTGAGGAATATTGGTCAATGGACGTAAGTCTGAACCAGCCAAGTAGCGTGAAGGATGAAGGCTCTATGGGTCGTAAACTTCTTTTATACGGGAATAAAAAGAGTCACGTGTGGCTTATTGCATGTACCGTATGAATAAGGATCGGCTAACTCCGTGCCAGCAGCCGC

+

:::::::::::::::::::::::::::::::::::::::::::::::::::::::::::::::::::::::::::::::::::::::::::::::::::::::::::::::::::::::::::::::::::::::::::::::::::::::::::::::::::::::::::::::::::::::::::::::::::::::::::::::::::::::::::::::::::::::::::::::::::::::::::::::::::::::::::::::::::::::::::::::::::::::::::::::::::::::::::::::::::::::::::::::::::::::::::::::::::::::::::::::::::::::::::::::::::::::::::::::::::::::::::::::::::::::::::::::::::::::::::::::::::::::::::::::::::::::::::::::::::::::::::::::::::::::

@D0950.29_102755

GATGAACGCTGGCGGCGTGCTTAACACATGCAAGTCGAGCGAGGAATCACCTTCGGGTGTGAACTAGCGGCGGACGGGTGAGTAACACGTGGGCAACCTGCCTTACAGAGGGGGATAGCCTTCCGAAAGGAAGATTAATACCGCATATTATGAGTTTTCTGCATGGGGAATTCATGAAAGGAGTAATCCGCTGTAAGATGGGCCCGCGGCGCATTAGCTAGTTGGTGAGGTAACGGCTCACCAAGGCGACGATGCGTAGCCGACCTGAGAGGGTGATCGGCCACATTGGGACTGAGACACGGCCCAGACTCCTACGGGAGGCAGCAGTGGGGAATATTGCACAATGGGGGAAACCCTGATGCAGCAACGCCGCGTGAGTGATGAAGGCCTTCGGGTTGTAAAGCTCTGTCTTCAGGGACGATAATGACGGTACCTGAGGAGGAAGCCACGGCTAACTACGTGCCAGCAGCCGCGGTAA

+

::::::::::::::::::::::::::::::::::::::::::::::::::::::::::::::::::::::::::::::::::::::::::::::::::::::::::::::::::::::::::::::::::::::::::::::::::::::::::::::::::::::::::::::::::::::::::::::::::::::::::::::::::::::::::::::::::::::::::::::::::::::::::::::::::::::::::::::::::::::::::::::::::::::::::::::::::::::::::::::::::::::::::::::::::::::::::::::::::::::::::::::::::::::::::::::::::::::::::::::::::::::::::::::::::::::::::::::::::::::::::::::::::::::::::::::::::::::::::::::

@D0950.29_102820

GATGAACGCTGGCGGCGTGCTTAACACATGCAAGTCGAACGAAGCGCTGGAGGAGCTTGCTCCAAAGGTGACTGAGTGGCGGACGGGTGAGTAACGCGTGGGTAACCTGCCTTACACTGGGGGATAACAGTTGGAAACGACTGCTAATACCGCATAAGCGCACAGTATTGCATGATACAGTGTGAAAAACTCCGGTGGTGTAAGATGGACCCGCGTCTGATTAGCTAGTTGGTGAGGTAATGGCTCACCAAGGCAACGATCAGTAGCCGGCTTGAGAGAGTGAACGGCCACATTGGGACTGAGACACGGCCCAAACTCCTACGGGAGGCAGCAGTGGGGAATATTGCACAATGGGGGAAACCCTGATGCAGCAACGCCGCGTGAGTGAAGAAGTATTTCGGTATGTAAAGCTCTATCAGCAGGGAAGATAATGACGGTACCTGACTAAGAAGCCCCGGCTAACTACGTGCCAGCAGCCGCGGTAA

+

:::::::::::::::::::::::::::::::::::::::::::::::::::::::::::::::::::::::::::::::::::::::::::::::::::::::::::::::::::::::::::::::::::::::::::::::::::::::::::::::::::::::::::::::::::::::::::::::::::::::::::::::::::::::::::::::::::::::::::::::::::::::::::::::::::::::::::::::::::::::::::::::::::::::::::::::::::::::::::::::::::::::::::::::::::::::::::::::::::::::::::::::::::::::::::::::::::::::::::::::::::::::::::::::::::::::::::::::::::::::::::::::::::::::::::::::::::::::::::::::::::::

@D0950.29_102852

GACGAACGCTGGCGGCGTGCTTAACACATGCAAGTCGAGCGATGAAGCTTCTTCGGAAGTGGATTAGCGGCGGACGGGTGAGTAACACGTGGGTAACCTGCCTCATAGAGGGGAATAGCCTTTCGAAAGGAAGATTATACCGCATAGATTGTAATACCGCATGGTATAGCAATTAAAGGAGTAATCCGCTATGAGATTGGGACCCGCGTCGCATTAGCTAGTTGGTGAGGTAATGGCTCACCAAGGCGACGATGCGTAGCCGACCTGAGAGGGTGATCGGCCACATTGGGACTGAGACACGGCCCAGACTCCTACGGGAGGCAGCAGTGGGGAATATTGCACAATGGGGGAAACCCTGATGCAGCAACGCCGCGTGAGTGATGACGGCCTTCGGGTTGTAAAACGTCTGTCTTTGGGGACGATAATGACGGTACCCAAGGAGGAAGCCAC

+

::::::::::::::::::::::::::::::::::::::::::::::::::::::::::::::::::::::::::::::::::::::::::::::::::::::::::::::::::::::::::::::::::::::::::::::::::::::::::::::::::::::::::::::::::::::::::::::::::::::::::::::::::::::::::::::::::::::::::::::::::::::::::::::::::::::::::::::::::::::::::::::::::::::::::::::::::::::::::::::::::::::::::::::::::::::::::::::::::::::::::::::::::::::::::::::::::::::::::::::::::::::::::::::::::::::::::::::::::::::::::::::::::

@D0950.29_102891

GATGAACGCTAGCTACAGGCTTAACACATGCAAGTCGAGGGGTAGCATGAAACTTAGCAATAAGTTTTGATGACGACCGGCGCACGGGTGAGTAACACGTATCCAACCTGCCTTTTACTCATGGATAGCCTTCTGAAAAGAAGATTAATACATGATGGTATTCAGAGTTTTCATGGACACTGAATTAAAGATTTATCGGTAAGAGATGGGGAATGCGTTCCATTAGATAGTAGGCGGGGTAACGGCCCACCTAGTCAACGATGGATAGGGGTTCTGAGAGGAAGGTCCCCACATTGGAACTGAGACACGGTCCAAACTCCTACGGGAGGCAGCAGTGAGGAATATTGGTCAATGGACGTAAGTCTGAACCAGCCAAGTAGCGTGAAGGATGAAGGCTCTATGGGTCTAAACTTCTTTTAT

+

::::::::::::::::::::::::::::::::::::::::::::::::::::::::::::::::::::::::::::::::::::::::::::::::::::::::::::::::::::::::::::::::::::::::::::::::::::::::::::::::::::::::::::::::::::::::::::::::::::::::::::::::::::::::::::::::::::::::::::::::::::::::::::::::::::::::::::::::::::::::::::::::::::::::::::::::::::::::::::::::::::::::::::::::::::::::::::::::::::::::::::::::::::::::::::::::::::::::::::::::::::::::::::::::::::

@D0950.29_102939

GATGAACGCTAGCGACAGGCCTAACACATGCAAGTCGAGGGGTAGCACAAGGAAGCTTGCTTCTGAGGTGACGACCGGCGCACGGGTGAGTAACGCGTATGCAACCTGCCTATAAGAAGGGGATAGCCTCTCGAAAGAGAGATTAATACCGTATAACACTATGAAACCGCATGGTTTTACAGTTAAAGATTTATTGCTTATAGATGGGCATGCGTAACATTAGCTAGTTGGTGAGGTAACGGCTCACCAAGGCAACGATGTTTAGGGGTTCTGAGAGGAAGGTCCCCCACACTGGTACTGAGACACGGACCAGACTCCTACGGGAGGCAGCAGTGAGGAATATTGGTCAATGGACGAGAGTCTGAACCAGCCAAGTCGCGTGAAGGATGAAGGTCTTATGGATTGTAAACTTCTTTTATACGGGGAAATAAAAAGAGCCACGTGTGGCTTATTGCATGTACCGTATGAATAAGGATCGGCTAACTCCGTGCCAGCAGCAGC

+

:::::::::::::::::::::::::::::::::::::::::::::::::::::::::::::::::::::::::::::::::::::::::::::::::::::::::::::::::::::::::::::::::::::::::::::::::::::::::::::::::::::::::::::::::::::::::::::::::::::::::::::::::::::::::::::::::::::::::::::::::::::::::::::::::::::::::::::::::::::::::::::::::::::::::::::::::::::::::::::::::::::::::::::::::::::::::::::::::::::::::::::::::::::::::::::::::::::::::::::::::::::::::::::::::::::::::::::::::::::::::::::::::::::::::::::::::::::::::::::::::::::::::::::::::::::

@D0950.29_102941

GATGAACGCTAGCTACAGGCTTAACACATGCAAGTCGAGGGGTAGCATGAAACTTAGCAATAAGTTTTGATGACGACCGGCGCACGGGTGAGTAACACGTATCCAACCTGCCTTTTACTCATGGATAGCCTTCTGAAAAGAAGATTAATACATGATGGTATTCAGAGTTTTCATGGACACTGAATTAAAGATTTATCGGTAAGAGATGGGGATGCGTTCCATTAGATAGTAGGCTGGGGTAACGGCCCACCTAGTCGAACGATGGATAGGGGTTCTGAGAGGAAGGTCCCCCACATTGGAACTGAGACACGGTCCAAACGTCCTACGGGAGGCAGCAGTGAGGAATATTGGTCAATGGACGTAAGTCTGAACCAGCCAAGTAGCGTGAAGGATGAAGGCTCTATGGGTCGTAAACTTCTTTTTATAAAA

+

:::::::::::::::::::::::::::::::::::::::::::::::::::::::::::::::::::::::::::::::::::::::::::::::::::::::::::::::::::::::::::::::::::::::::::::::::::::::::::::::::::::::::::::::::::::::::::::::::::::::::::::::::::::::::::::::::::::::::::::::::::::::::::::::::::::::::::::::::::::::::::::::::::::::::::::::::::::::::::::::::::::::::::::::::::::::::::::::::::::::::::::::::::::::::::::::::::::::::::::::::::::::::::::::::::::::::::::

@D0950.29_102982

GATGAACGCTAGCTACAGGCTTAACACATGCAAGTCGAGGGGTAGCATGAAACTTAGCAATAAGTTTTGATGACGACCGGCGCACGGGTGAGTAACACGTATCCAACCTGCCTTTTACTCATGGATAGCCTTCTGAAAAGAAGATTAATACATGATGGTATTCAGAGTTTTCATGGACACTGAATTAAAGATTTGATCGGTAAGAGATGGGGATGCGTTCCATTAGATAGTAGGCGGGGTAACGGCCCACCTAGTCAACGATGGATAGGGGTTCTGAGAGGAAGGTCCCCCACACTGGTACTGAGACACGGACCAGACTCCCTACGGGAGGCAGCAGTGAGGAATATTGGTCAATGGGCGCGAGCCTGAACCAGCCAAGTCGCGTGAAGGATGAAGGTCTTATGGATTGTAAACTTCTTTTATACGGGAATAAAAAATTGCCACGTGTGGCATATTGCATGTACCGTATGAATAAGGATCGGCTAACTCCGTGCC

+

:::::::::::::::::::::::::::::::::::::::::::::::::::::::::::::::::::::::::::::::::::::::::::::::::::::::::::::::::::::::::::::::::::::::::::::::::::::::::::::::::::::::::::::::::::::::::::::::::::::::::::::::::::::::::::::::::::::::::::::::::::::::::::::::::::::::::::::::::::::::::::::::::::::::::::::::::::::::::::::::::::::::::::::::::::::::::::::::::::::::::::::::::::::::::::::::::::::::::::::::::::::::::::::::::::::::::::::::::::::::::::::::::::::::::::::::::::::::::::::::::::::::::::::::

@D0950.29_103002

GATGAACGCTAGCGACAGGCCTAACACATGCAAGTCGAGGGGTAGCACAAGGGAGCTTGCTTCTGAGGTGACGACCGGCGCACGGGTGAGTAACGCGTATGCAACCTGCCTATAAGAAGGGGATAGCCTCTCGAAAGAGAGATTAATACCGTATAACACTATGAGGCCGCATGGTTTTATAGTTAAAGATTTATTGCTTATAGATGGGCATGCGTAACATTAGCTAGTTGGTAAGGTAACGGCTTACCAAGGCAACGATGTTTAGGGGTTCTGAGAGGAAGGTCCCCCACACTGGTACTGAGACACGGACCAGACTCCTACGGGAGGCAGCAGTGAGGAATATTGGTCAATGGACGAGAGTCTGAACCAGCCAAGTCGCGTGAAGGATGAAGGTCTTATGGATTGTAAACTTCTTTTATACGGGGAAATAAAAAACTACCACGTGTGGTATATTGCATGTACCGTATGAATAAGGATCGGCTAACTCCGTGCCAGCAGCCGC

+

::::::::::::::::::::::::::::::::::::::::::::::::::::::::::::::::::::::::::::::::::::::::::::::::::::::::::::::::::::::::::::::::::::::::::::::::::::::::::::::::::::::::::::::::::::::::::::::::::::::::::::::::::::::::::::::::::::::::::::::::::::::::::::::::::::::::::::::::::::::::::::::::::::::::::::::::::::::::::::::::::::::::::::::::::::::::::::::::::::::::::::::::::::::::::::::::::::::::::::::::::::::::::::::::::::::::::::::::::::::::::::::::::::::::::::::::::::::::::::::::::::::::::::::::::::::

@D0950.29_103060

GATGAACGCTAGCGACAGGCCTAACACATGCAAGTCGAGGGGTAGCACAAGGTAGCAATACTGAGGTGACGACCGGCGCACGGGTGAGTAACGCGTATGCAACCTACCTGTAAGAGTGGGATAGCCTCTCGAAAGAGAGATTAATACCGCATAATACCATTTTACTGCATGGTGAGATGGTTAAAGATTTATTGCTTACAGATGGGCATGCGTAACATTAGCTAGTTGGTGAGGTAACGGCTCACCAAGGCAACGATGTTTAGGGGTTCTGAGAGGAAGGTCCCCCACACTGGTACTGAGACACGGACCAGACTCCTACGGGAGGCAGCAGTGAGGAATATTGGTCAATGGACGAGAGTCTGAACCAGCCAAGTCGCGTGAAGGATGAAGGTCTTATGGATTGTAAACTTCTTTTATACGGGAATAAAAATGCCACGTGTGGCATATTGCATGTACCGTATGAATAAGGATCGGCTAACTCCGTGCCAGCAGCCGC

+

::::::::::::::::::::::::::::::::::::::::::::::::::::::::::::::::::::::::::::::::::::::::::::::::::::::::::::::::::::::::::::::::::::::::::::::::::::::::::::::::::::::::::::::::::::::::::::::::::::::::::::::::::::::::::::::::::::::::::::::::::::::::::::::::::::::::::::::::::::::::::::::::::::::::::::::::::::::::::::::::::::::::::::::::::::::::::::::::::::::::::::::::::::::::::::::::::::::::::::::::::::::::::::::::::::::::::::::::::::::::::::::::::::::::::::::::::::::::::::::::::::::::::::::::

@D0950.29_103088

GATGAACGCTAGCTACAGGCTTAACACATGCAAGTCGAGGGGTAGCATGAAACTTAGCAATAAGTTTTGATGACGACCGGCGCACGGGTGAGTAACACGTATCCAACCTGCCTTTTACTCATGGATAGCCTTCTGAAAGAAGATTAATACATGATGGTATTCAGAGTTTTCATGGACACTGAATTAAAGATTTATCGGTAAGAGATGGGGATGCGTTCCATTAGATAGTAGGCGGGGTAACGGCCCACCTAGTCAACGATGGATAGGGGTTCTGAGAGGAAGGTCCCCAGCATTGGAACTGAGACACGGTCCAAAACTCCTACGGGAGGCAGCAGTGGGGAATATTGCACAATGGGCGAAAGCCTGATGCAGCAACGCCGCGTGAGTGATGAAGGCCTTCGGGTCGTAAAACTCTGTCCTCAAGG

+

:::::::::::::::::::::::::::::::::::::::::::::::::::::::::::::::::::::::::::::::::::::::::::::::::::::::::::::::::::::::::::::::::::::::::::::::::::::::::::::::::::::::::::::::::::::::::::::::::::::::::::::::::::::::::::::::::::::::::::::::::::::::::::::::::::::::::::::::::::::::::::::::::::::::::::::::::::::::::::::::::::::::::::::::::::::::::::::::::::::::::::::::::::::::::::::::::::::::::::::::::::::::::::::::::::::::::

@D0950.29_103106

GATGAACGCTGGCGGCGTGCTTAACACATGCAAGTCGAACGAAGCACTTTGAAGAGCTTGCTCTTTAAAGTGACTGAGTGGCGGACGGTGAGTAACGCGTGGGTAACCTGCCTCATACAGGGGGATAACAGTTAGAAATGACCTGCTAACACCGCATAACCCGCTAGTGCGCATGACACAGACGGAAAATATTTATAGGTATGAGATGGGCCCGCGTCTGATTAGCTAGTTGGTGGGGTAACGGCCTACCAAGGCAACGATCAGTAGCCGACTTGAGAGAGTGATCGGCCACATTGGGACTGAGACACGGCCCAAACTCCTACGGGAGGCAGCAGTGGGGAATATTGGACAATGGGGGAAACCCTGATCCAGCGACGCCGCGTGAGTGAAGAAGTATTTTCGGTATGTAAAGCTCTATCAGCAGGGGAAAGATAATGACAGTACCTGACTAAGAAGCCCCGGCTAACTACGTGCCAGCAGCCGCGGTAA

+

:::::::::::::::::::::::::::::::::::::::::::::::::::::::::::::::::::::::::::::::::::::::::::::::::::::::::::::::::::::::::::::::::::::::::::::::::::::::::::::::::::::::::::::::::::::::::::::::::::::::::::::::::::::::::::::::::::::::::::::::::::::::::::::::::::::::::::::::::::::::::::::::::::::::::::::::::::::::::::::::::::::::::::::::::::::::::::::::::::::::::::::::::::::::::::::::::::::::::::::::::::::::::::::::::::::::::::::::::::::::::::::::::::::::::::::::::::::::::::::::::::::::::

@D0950.29_103108

GATGAACGCTAGCTACAGGCTTAACACATGCAAGTCGAGGGGTAGCATGAAACTTAGCAATAAGTTTTGATGACGACCGGCGCACGGGTGAGTAACACGTATCCAACCTGCCTATAAGAAGGGGATAGCCTCTCGAAAGAGAGATTAATACCGTATAACACTATGAAGCCGCATGGTTTTATAGTTAAAGATTTATTGCTTATAGATGGGCATGCGTAACATTAGCTAGTTGGTAAGGTAACGGCTTACCAAGGCAACGATGTTTAGGGGTTCTGAGAGGAAGGTCCCCCACACTGGTACTGAGACACGGACCAGACTCCTACGGGAGGCAGCAGTGAGGAATATTGGTCAATGGACGAGAGTCTGAACCAGCCAAGTCGCGTGAAGGATGAAGGTCTTATGGATTGTAAACTTCTTTTATACGGGGAAATAAAAAACTACCACGTGTGGTATATTGCAATGTACCGTATGAATAAGGATCGGCTAACTCCGTGCCAGCAGCCGC

+

:::::::::::::::::::::::::::::::::::::::::::::::::::::::::::::::::::::::::::::::::::::::::::::::::::::::::::::::::::::::::::::::::::::::::::::::::::::::::::::::::::::::::::::::::::::::::::::::::::::::::::::::::::::::::::::::::::::::::::::::::::::::::::::::::::::::::::::::::::::::::::::::::::::::::::::::::::::::::::::::::::::::::::::::::::::::::::::::::::::::::::::::::::::::::::::::::::::::::::::::::::::::::::::::::::::::::::::::::::::::::::::::::::::::::::::::::::::::::::::::::::::::::::::::::::::::::

@D0950.29_103149

GATGAACGCTGGCGGCGTGCTTAACACATGCAAGTCGAGCGAAGCATTAAAACAGATTTCTTCGGATTGAAGTTTTGTGACTGAGCGGCGGACGGGTGAGTAACGCGTGGGTAACCTGCCTTACACTGGGGGATAACAGTTGGAAACGACTGCTAATACCGCATAAGCGCACAGTATTGCATGATACAGTGTGAAAAACTCCGGTGGTGTAAGATGGACCCGCGTCTGATTAGCTAGTTGGTGAGGTAATGGCTCACCAAGGCAACGATCAGTAGCCGGCTTGAGAGAGTGAACGGCCACATTGGGACTGAGACACGGCCCAAACTCCTACGGGAGGCAGCAGTGGGGAATATTGCACAATGGGGGAAACCCTGATGCAGCAACGCCGCGTGAGTGAAGAAGTATTTCGGTATGTAAAGCTCTATCAGCAGGGAAGATAATGACGGTACCTGACTAAGAAGCCCCCGGCTAACTACGTGCCAGCAGCCGC

+

::::::::::::::::::::::::::::::::::::::::::::::::::::::::::::::::::::::::::::::::::::::::::::::::::::::::::::::::::::::::::::::::::::::::::::::::::::::::::::::::::::::::::::::::::::::::::::::::::::::::::::::::::::::::::::::::::::::::::::::::::::::::::::::::::::::::::::::::::::::::::::::::::::::::::::::::::::::::::::::::::::::::::::::::::::::::::::::::::::::::::::::::::::::::::::::::::::::::::::::::::::::::::::::::::::::::::::::::::::::::::::::::::::::::::::::::::::::::::::::::::::::::::

@D0950.29_103211

GATGAACGCTAGCTACAGGCTTAACACATGCAAGTCGAGGGGTAGCATGAAACTTAGCAATAAGTTTTGATGACGACCGGCGCACGGGTGAGTAACACGTATCCAACCTGCCTTTTACTCATGGATAGCCTTCTGAAAAGAAGATTAATACATGATGGTATTCAGAGTTTTCATGGACACTGAATTAAAGATTTTATCGGTAAGAGATGGGGATGCGTTCCATTAGATAGTAGGCTGGGGTAACGGCCCACCTAGTCGAACGATCGGATAGGGGTTCTGAGAGGAAGGTCCCCCACATTGGAACTGAGACACGGTCCAAACGTCCGTACGGGAGGCAGCAGTGAGGGAATATTGGTCAATGGACGTAAGTCTGAACCAGCCAAGTAGCGTGAAGGATGAAGGCTCTATGGGTCGTAAACTTCTTTTTATAAAA

+

:::::::::::::::::::::::::::::::::::::::::::::::::::::::::::::::::::::::::::::::::::::::::::::::::::::::::::::::::::::::::::::::::::::::::::::::::::::::::::::::::::::::::::::::::::::::::::::::::::::::::::::::::::::::::::::::::::::::::::::::::::::::::::::::::::::::::::::::::::::::::::::::::::::::::::::::::::::::::::::::::::::::::::::::::::::::::::::::::::::::::::::::::::::::::::::::::::::::::::::::::::::::::::::::::::::::::::::::::

@D0950.29_103231

GATGAACGCTAGCTACAGGCTTAACACATGCAAGTCGAGGGGTAGCATGAAACTTAGCAATAAGTTTTGATGACGACCGGCGCACGGGTGAGTAACACGTATCCAACCTGCCTTTTACTCATGGATAGCCTTCTGAAAAGAAGATTAATACATGATGGTATTCAGAGTTTTCATGGACACTGAATTAAAGATTTTATCGGTAAGAGATGGGGATGCGTTCCATTAGATAGTAGGCGGGGTAACGGCCCACCTAGTCAACGATGGATAGGGGTTCTGAGAGGAAGGTCCCCCACATTGGAACTGAGACACGGTCCAAACTCCGTACGGGAGGCAGCAGTGAGGGAATATTGGTCAATGGACGTAAGTCTGAACCAGCCAAGTAGCGTGAAGGATGAAGGCTCTATGGGTCGTAAACTTC

+

::::::::::::::::::::::::::::::::::::::::::::::::::::::::::::::::::::::::::::::::::::::::::::::::::::::::::::::::::::::::::::::::::::::::::::::::::::::::::::::::::::::::::::::::::::::::::::::::::::::::::::::::::::::::::::::::::::::::::::::::::::::::::::::::::::::::::::::::::::::::::::::::::::::::::::::::::::::::::::::::::::::::::::::::::::::::::::::::::::::::::::::::::::::::::::::::::::::::::::::::::::::::::::::::::

@D0950.29_103295

GATGAACGCTAGCTACAGGCTTAACACATGCAAGTCGAGGGGTAGCATGAAACTTAGCAATAAGTTTTGATGACGACCGGCGCACGGGTGAGTAACACGTATCCAACCTGCCTTTTACTCATGGATAGCCTTCTGAAAAGAAGATTAATACATGATGGTATTCAGAGTTTTCATGGACACTGAATTAAAGATTTATCGGTAAGAGATGGGGATGCGTTCCATTAGATAGTAGGCGGGGTAACGGCCCACCTAGTCAACGATGGATAGGGGTTCTGAGAGGAAGGTCCCCCACATTGGAACTGAGACACGGTCCAAACGTCCTACGGGAGGCAGCAGTGAGGAATATTGGTCAATGGACGTAAGTCTGAACCAGCCAAGTAGCGTGAAGGATGAAGGCTCTATGGGTCGTAAACTTCTTTTTATAAAAGGAATAAAGTATGCCACGTGTGGTGTTTTGTATGTACTTTATGAATAAGGATCGGCTAACTCCGTGCCAGCAGCCGC

+

::::::::::::::::::::::::::::::::::::::::::::::::::::::::::::::::::::::::::::::::::::::::::::::::::::::::::::::::::::::::::::::::::::::::::::::::::::::::::::::::::::::::::::::::::::::::::::::::::::::::::::::::::::::::::::::::::::::::::::::::::::::::::::::::::::::::::::::::::::::::::::::::::::::::::::::::::::::::::::::::::::::::::::::::::::::::::::::::::::::::::::::::::::::::::::::::::::::::::::::::::::::::::::::::::::::::::::::::::::::::::::::::::::::::::::::::::::::::::::::::::::::::::::::::::::::::

@D0950.29_103309

GATGAACGCTAGCGACAGGCCTAACACATGCAAGTCGAGGGGTAGCACAAGGTAGCAATACTGAGGTGACGACCGGCGCACGGGTGAGTAACGCGTATGCAACCTACCTGTAAGAGTGGGATAGCCTCTCGAAAGAGAGATTAATACCGCATAATACCATTTTACTGCATGGTGAGATGGTTAAAGATTTGTTGCTTACAGATGGGCATGCGTAACATTAGCTAGTTGGTGAGGTAACGGCTCACCAAGGCAACGATGTTTAGGGGTTCTGAGAGGAAGGTCCCCCACACTGGTACTGAGACACGGACCAGACTCCTACGGGAGGCAGCAGTGAGGAATATTGGTCAATGGACGAGAGTCTGAACCAGCCAAGTCGCGTGAAGGATGAAGGTCTTATGGATTGTAAACTTCTTTTATACGGGAATAAAAATGCCACGTGTGGCATATTGCATGTACCGTATGAATAAGGATCGGCTAACTCCGTGCCAGCAGCCGCGGTAA

+

:::::::::::::::::::::::::::::::::::::::::::::::::::::::::::::::::::::::::::::::::::::::::::::::::::::::::::::::::::::::::::::::::::::::::::::::::::::::::::::::::::::::::::::::::::::::::::::::::::::::::::::::::::::::::::::::::::::::::::::::::::::::::::::::::::::::::::::::::::::::::::::::::::::::::::::::::::::::::::::::::::::::::::::::::::::::::::::::::::::::::::::::::::::::::::::::::::::::::::::::::::::::::::::::::::::::::::::::::::::::::::::::::::::::::::::::::::::::::::::::::::::::::::::::::::::

@D0950.29_103401

GATGAACGCTAGCGACAGGCCTAACACATGCAAGTCGAGGGGTAGCACAAGGAAGCTTGCTTCTGAGGTGACGACCGGCGCACGGGTGAGTAACGCGTATGCAACCTGCCTATAAGAAGGGGATAGCCTCTCGAAAGAGAGATTAATACCGTATAACACTATGAAACCGCATGGTTTTACAGTTAAAGATTTATTGCTTATAGATGGGCATGCGTAACATTAGCTAGTTGGTGAGGTAACGGCTCACCAAGGCAACGATGTTTAGGGGTTCTGAGAGGAAGGTCCCCCACACTGGTACTGAGACACGGACCAGACTCCTACGGGAGGCAGCAGTGAGGAATATTGGTCAATGGACGAGAGTCTGAACCAGCCAAGTCGCGTGAAGGATGAAGGTCTTATGGATTGTAAACTTCTTTTATACGGGGAAATAAAAAGAGCCACGTGTGGCTTATTGCATGTACCGTATGAATAAGGATCGGCTAACTCCGTGCCAGCAGCCG

+

::::::::::::::::::::::::::::::::::::::::::::::::::::::::::::::::::::::::::::::::::::::::::::::::::::::::::::::::::::::::::::::::::::::::::::::::::::::::::::::::::::::::::::::::::::::::::::::::::::::::::::::::::::::::::::::::::::::::::::::::::::::::::::::::::::::::::::::::::::::::::::::::::::::::::::::::::::::::::::::::::::::::::::::::::::::::::::::::::::::::::::::::::::::::::::::::::::::::::::::::::::::::::::::::::::::::::::::::::::::::::::::::::::::::::::::::::::::::::::::::::::::::::::::::::::

@D0950.29_1035

GATGAACGCTGGCGGCGTGCTTAACACATGCAAGTCGAGCGAGGAATCACCTTCGGTGTGAACTAGCGGCGGACGGGTGAGTAACACGTGGGCAACCTGCCTTACAGAGGGGGATAGCCTTCCGAAAGGAAGATTAATACCGCATATTATGAGTTTTCTGCATGGGGAATTCATGAAAGGAGCAATCCGCTGTAAGATGGGCCCGCGGCGCATTAGCTAGTTGGTGAGGTAACGGCTCACCAAGGCGACGATGCGTAGCCGACCTGAGAGGGTGATCGGCCACATTGGGACTGAGACACGGCCCAGACTCCTACGGGAGGCAGCAGTGGGGAATATTGCACAATGGGGGAAACCCTGATGCAGCAACGCCGCGTGAGTGATGAAGGCCTTCGGGTTGTAAAGCTCTGTCTTCAGGGACGATAATGACGGT

+

::::::::::::::::::::::::::::::::::::::::::::::::::::::::::::::::::::::::::::::::::::::::::::::::::::::::::::::::::::::::::::::::::::::::::::::::::::::::::::::::::::::::::::::::::::::::::::::::::::::::::::::::::::::::::::::::::::::::::::::::::::::::::::::::::::::::::::::::::::::::::::::::::::::::::::::::::::::::::::::::::::::::::::::::::::::::::::::::::::::::::::::::::::::::::::::::::::::::::::::::::::::::::::::::::::::::::::::

@D0950.29_103534

GATGAACGCTAGCTACAGGCTTAACACATGCAAGTCGAGGGGTAGCATGAAACTTAGCAATAAGTTTTGATGACGACCGGCGCACGGGTGAGTAACACGTATCCAACCTGCCTTTTACTCATGGATAGCCTTCTGAAAAGAAGATTAATACATGATGGTATTCAGAGTTTTCATGGACACTGAATTAAAGATTTTATCGGTAAGAGATGGGGATGCGTTCCATTAGATAGTAGGCTGGGGTAACGGCCCACCTAGTCGAACGATCGGATAGGGGTTCTGAGAGGAAGGTCCCCCACATTGGAACTGAGACACGGTCCAAACGTCCTACGGGAGGCAGCAGTGAGGAATATTGGTCAATGGACGTAAGTCTGAACCAGCCAAGTAGCGTGAAGGATGAAGGCTCTATGGGTCGTAAACTTCTTTTATAAAAGGAATAAAGTATGCCACGTGTGGTGTTTTTGTATGTACTTTATGAATAAGGATCGGCTAACTCCGTGCCAGCAGCCGC

+

::::::::::::::::::::::::::::::::::::::::::::::::::::::::::::::::::::::::::::::::::::::::::::::::::::::::::::::::::::::::::::::::::::::::::::::::::::::::::::::::::::::::::::::::::::::::::::::::::::::::::::::::::::::::::::::::::::::::::::::::::::::::::::::::::::::::::::::::::::::::::::::::::::::::::::::::::::::::::::::::::::::::::::::::::::::::::::::::::::::::::::::::::::::::::::::::::::::::::::::::::::::::::::::::::::::::::::::::::::::::::::::::::::::::::::::::::::::::::::::::::::::::::::::::::::::::::::

@D0950.29_103541

GACGAACGCTGGCGGCGTGCTTAACACATGCAAGTCGAGCGATGAAGCTTCTTCGGAAGCGGATTAGCGGCGGACGGGTGAGTAACACGTGGGTAACCTGCCTCATAGAGGGGAATAGCCTTTCGAAAGGAAGATTAATACCGCATAAGATTGTAGTACCGCATGGTACAGCAATTAAAGGAGTAATCCGCTACAAGATGGACCCGCGGCGCATTAGCTAGTTGGTGAGGTAACGGCTCACCAAGGCGACGATGCGTAGCCGACCTGAGAGGGTGATCGGCCACATTGGGAACTGAGACACGGTCCAGACTCCTACGGGAGGCAGCAGTGGGGAATATTGCGCAATGGGGGAAACCCTGACGCAGCAACGCCGCGTGAATGAAGAAGGCCTTAGGGTTGTAAGTTCTGTTTACGGGGACGATAATGACGGTACCCGTGGAGGAAGCCACGGCTAACTACGTGCCAGCAGCCGCGGTAA

+

::::::::::::::::::::::::::::::::::::::::::::::::::::::::::::::::::::::::::::::::::::::::::::::::::::::::::::::::::::::::::::::::::::::::::::::::::::::::::::::::::::::::::::::::::::::::::::::::::::::::::::::::::::::::::::::::::::::::::::::::::::::::::::::::::::::::::::::::::::::::::::::::::::::::::::::::::::::::::::::::::::::::::::::::::::::::::::::::::::::::::::::::::::::::::::::::::::::::::::::::::::::::::::::::::::::::::::::::::::::::::::::::::::::::::::::::::::::::::::::

@D0950.29_1036

ATTGAACGCTGGCGGCATGCCTTACACATGCAAGTCGAACGGTAACAGGTCTTCGGATGCTGACGAGTGGCGAACGGGTGAGTAATACATCGGAACGTGCCCGATCGTGGGGATAACGAAGCGAAAGCTTTGCTAATACCGCATACGATCTACGGATGAAAGCAGGGGACCGCAAGGCCTTGCGCGAACGGAGCGGCCGATGGCAGATTAGGTAGTTGGTGGGATAAAAGCTTACCAAGCCGACGATCTGTAGCTGGTCTGAGAGGACGACCAGCCACACTGGGACTGAGACACGGCCCAGACTCCTACGGGAGGCAGCAGTGGGGAATTTTGGACAATGGGCGAAAGCCTGATCCAGCCATGCCGCGTGCAGGATGAAGGCCTTCGGGTTGTAAACTGCTTTTGTACGGAACGAAAAGACTCTGGTTAATACCTGGGGTCCATGACGGTACCGTAAGAATAAGCACCGGCTAACTACGTGCCAGCAGCCGCGGTAA

+

:::::::::::::::::::::::::::::::::::::::::::::::::::::::::::::::::::::::::::::::::::::::::::::::::::::::::::::::::::::::::::::::::::::::::::::::::::::::::::::::::::::::::::::::::::::::::::::::::::::::::::::::::::::::::::::::::::::::::::::::::::::::::::::::::::::::::::::::::::::::::::::::::::::::::::::::::::::::::::::::::::::::::::::::::::::::::::::::::::::::::::::::::::::::::::::::::::::::::::::::::::::::::::::::::::::::::::::::::::::::::::::::::::::::::::::::::::::::::::::::::::::::::::::::::

@D0950.29_103667

GATGAACGCTAGCGACAGGCCTAACACATGCAAGTCGAGGGGTAGCACAAGGAAGCTTGCTTCTGAGGTGACGACCGGCGCACGGGTGAGTAACGCGTATGCAACCTGCCTATAAGAAGGGGATAGCCTCTCGAAAGAGAGATTAATACCGTATAACACTATGAAACCGCATGGTTTTACAGTTAAAGATTTATTGCTTATAGATGGGCATGCGTAACATTAGCTAGTTGGTAAGGTAACGGCTTACCAAGGCAACGATGTTTAGGGGTTCTGAGAGGAAGGTCCCCCACACTGGTACTGAGACACGGACCAGACTCCTACGGGAGGCAGCAGTGAGGAATATTGGTCAATGGACGAGAGTCTGAACCAGCCAAGTCGCGTGAAGGATGAAGGTCTTATGGATTGTAAACTTCTTTTATACGGGAATAAAAAATGCCACGTGTGGCATATTGCATGTACCGTATGAATAAGGATCGGCTAACTCCGTGCCAGCAGCCG

+

::::::::::::::::::::::::::::::::::::::::::::::::::::::::::::::::::::::::::::::::::::::::::::::::::::::::::::::::::::::::::::::::::::::::::::::::::::::::::::::::::::::::::::::::::::::::::::::::::::::::::::::::::::::::::::::::::::::::::::::::::::::::::::::::::::::::::::::::::::::::::::::::::::::::::::::::::::::::::::::::::::::::::::::::::::::::::::::::::::::::::::::::::::::::::::::::::::::::::::::::::::::::::::::::::::::::::::::::::::::::::::::::::::::::::::::::::::::::::::::::::::::::::::::::::

@D0950.29_103668

GATGAACGCTAGCTACAGGCTTAACACATGCAAGTCGAGGGGTAGCATGAAACTTAGCAATAAGTTTTGATGACGACCGGCGCACGGGTGAGTAACACGTATCCAACCTGCCTTTTACTCATGGATAGCCTTCTGAAAAGAAGATTAATACATGATGGTATTCAGAGTTTTCATGGACACTGAATTAAAGATTTATCGGTAAGAGATGGGGATGCGTTCCATTAGATAGTAGGCGGGGTAACGGCCCACCTAGTCAACGATGGATAGGGGTTCTGAGAGGAAGGTCCCCCACATTGGAACTGAGACACGGTCCAAACGTCCTACGGGAGGCAGCAGTGAGGAATATTGGTCAATGGACGTAAGTCTGAACCAGCCAAGTAGCGTGAAGGATGAAGGCTCTATGGGTCGTAAACTTCTTTTTATAAAAGGAATAAAGTAGCCACGTGTGGTGTTTTTGTATGTACTTTATGAATAAGGATCGGCTAACTCCGTGCCAGCAGCCGC

+

::::::::::::::::::::::::::::::::::::::::::::::::::::::::::::::::::::::::::::::::::::::::::::::::::::::::::::::::::::::::::::::::::::::::::::::::::::::::::::::::::::::::::::::::::::::::::::::::::::::::::::::::::::::::::::::::::::::::::::::::::::::::::::::::::::::::::::::::::::::::::::::::::::::::::::::::::::::::::::::::::::::::::::::::::::::::::::::::::::::::::::::::::::::::::::::::::::::::::::::::::::::::::::::::::::::::::::::::::::::::::::::::::::::::::::::::::::::::::::::::::::::::::::::::::::::::

@D0950.29_103694

GATGAACGCTAGCTACAGGCTTAACACATGCAAGTCGAGGGGTAGCATGAAACTTAGCAATAAGTTTTGATGACGACCGGCGCACGGGTGAGTAACACGTATCCAACCTGCCTTTTACTCATGGATAGCCTTCTGAAAAGAAGATTAATACATGATGGTATTCAGAGTTTTCATGGACACTGAATTAAAGATTTTATCGGTAAGAGATGGGGATGCGTTCCATTAGATAGTAGGCGGGTAACGGCCCACCTAGTCAACGATGGATAGGGGTTCTGAGAGGAAGGTCCCCCACATTGGAACTGAGACACGGTCCAAACGTCCTACGGGAGGCAGCAGTGAGGAATATTGGTCAATGGACGTAAGTCTGAACCAGCCAAGTAGCGTGAAGGATGAAGGCTCTATGGGTCGTAAACTTCTTTTTATAAAAGGAATAAAGTATGCCACGTGTGGTGTTTTTGTATGTACTTTATGAATAAGGATCGGCTAACTCCGTGCCAGCAGCCGC

+

:::::::::::::::::::::::::::::::::::::::::::::::::::::::::::::::::::::::::::::::::::::::::::::::::::::::::::::::::::::::::::::::::::::::::::::::::::::::::::::::::::::::::::::::::::::::::::::::::::::::::::::::::::::::::::::::::::::::::::::::::::::::::::::::::::::::::::::::::::::::::::::::::::::::::::::::::::::::::::::::::::::::::::::::::::::::::::::::::::::::::::::::::::::::::::::::::::::::::::::::::::::::::::::::::::::::::::::::::::::::::::::::::::::::::::::::::::::::::::::::::::::::::::::::::::::::::

@D0950.29_103758

GATGAACGCTGGCGGCGTGCTTAACACATGCAAGTCGAACGAAGCACTTTGAAGAGCTTGCTCTTCAAAGTGACTGAGTGGCGGACGGGTGAGTAACGCGTGGGTAACCTGCCTCATACAGGGGGATAACAGTTAGAAATGACTGCTAACACCGCATAACCCGCTAGTGTCGCATGACACGGACGGAAAATATTTATAGGGTATGAGATGGGCCCGCGTCTGATTACGCTAGTTGGTGGGGTAAAGGCCTACCAAGGCAACGATCAGTAGCCGACTTGAGAGAGTGATCGGCCACATTGGGACTGAGACACGGCCCAAACTCCTACGGGAGGCAGCAGTGGGGAATATTGGACAATGGGGGCAACCCTGATCCAGCGACGCCGCGTGAGTGAAGAAGTATTTTCGGGTATGTAAAGCTCTATCAGCAGGGGAAGATAATGACAGTACCTGACTAAGAAGCCCCGGCTAACTACGTGCCAGCAGCCGCGGTAA

+

::::::::::::::::::::::::::::::::::::::::::::::::::::::::::::::::::::::::::::::::::::::::::::::::::::::::::::::::::::::::::::::::::::::::::::::::::::::::::::::::::::::::::::::::::::::::::::::::::::::::::::::::::::::::::::::::::::::::::::::::::::::::::::::::::::::::::::::::::::::::::::::::::::::::::::::::::::::::::::::::::::::::::::::::::::::::::::::::::::::::::::::::::::::::::::::::::::::::::::::::::::::::::::::::::::::::::::::::::::::::::::::::::::::::::::::::::::::::::::::::::::::::::::

@D0950.29_103772

ATTGAACGCTGGCGGCATGCCTTACACATGCAAGTCGAACGGTAACAGGTCTTCGGATGCTGACGAGTGGCGAACGGGTGAGTAATACATCGGAACGTGCCCGATCGTGGGGGATAACGAAGCGAAAGCTTTGCTAATACCGCATACGATCTACGGATGAAAGCAGGGGACCGCAAGGCCTTGCGCGAACGGAGCGGCCGATGGCAGATTAGGTAGTTGGTGGGATAAAAGCTTACCAAGCCGACGATCTGTAGCTGGTCTGAGAGGACGACCAGCCACACTGGGACTGAGACACGGCCCAGACTCCTACGGGAGGCAGCAGTGGGGAATTTTGGACAATGGGCGAAAGCCTGATCCAGCCATGCCGCGTGCAGGATGAAGGCCTTCGGGTTGTAAACTGCTTTTGTACGGAACGAAAAGACTCTGGTTAATACCTGGGGTCCATGACGGTACCGTAAGAATAAGCACCGGCTAACTACGTGCCAGCAGCCGCGGTAA

+

::::::::::::::::::::::::::::::::::::::::::::::::::::::::::::::::::::::::::::::::::::::::::::::::::::::::::::::::::::::::::::::::::::::::::::::::::::::::::::::::::::::::::::::::::::::::::::::::::::::::::::::::::::::::::::::::::::::::::::::::::::::::::::::::::::::::::::::::::::::::::::::::::::::::::::::::::::::::::::::::::::::::::::::::::::::::::::::::::::::::::::::::::::::::::::::::::::::::::::::::::::::::::::::::::::::::::::::::::::::::::::::::::::::::::::::::::::::::::::::::::::::::::::::::::

@D0950.29_103780

GATGAACGCTGGCGGCGTGCTTAACACATGCAAGTCGAACGAAGCACTTTGAAGAGCTTGCTCTTTAAAGTGACTGAGTGGCGGACGGGTGAGTAACGCGTGGGTAACCTGCCTCATACAGGGGGATAACAGTTAGAAATGGACTGCTAACACCGCATAACCCGCTAGTGTCGCATGACACGGACGGAAAATATTTATAGGTATGAGATGGGCCCGCGTCTGATTAGCTAGTTGGTGGGGTAACAGCCTACCAAGGCAACGATCAGTAGCCGACTTGAGAGAGTGATCGGCCACATTGGGACTGAGACACGGCCCAAACTCCTACGGGAGGCAGCAGTGGGGAATATTGGACAATGGGGGAAACCCTGATCCAGCGACGCCGCGTGAGTGAAGAAGTATTTCGGTATGTAAAGCTCTATCAGCAGGGAAGATAATGACAGTACCTGACTAAGAAGCCCCCGGCTAACTACGTGCCAGCAGCCG

+

:::::::::::::::::::::::::::::::::::::::::::::::::::::::::::::::::::::::::::::::::::::::::::::::::::::::::::::::::::::::::::::::::::::::::::::::::::::::::::::::::::::::::::::::::::::::::::::::::::::::::::::::::::::::::::::::::::::::::::::::::::::::::::::::::::::::::::::::::::::::::::::::::::::::::::::::::::::::::::::::::::::::::::::::::::::::::::::::::::::::::::::::::::::::::::::::::::::::::::::::::::::::::::::::::::::::::::::::::::::::::::::::::::::::::::::::::::::::::::::::::::

@D0950.29_10382

GATGAACGCTGGCGGCGTGCTTAACACATGCAAGTCGAACGAAGCGCTGGAGGAGCTTGCTCCAAAGGTGACTGAGTGGCGGACGGGTGAGTAACGCGTGGGTAACCTGCCTTACACTGGGGGATAACAGTTGGAAACGACTGCTAATACCGCATAAGCGCACAGTATTGCATGATACAGTGTGAAAAACTCCGGTGGTGTAAGATGGACCCGCGTCTGATTAGCTAGTTGGTGAGGTAATGGCTCACCAAGGCAACGATCAGTAGCCGGCTTGAGAGAGTGAACGGCCACATTGGGACTGAGACACGGCCCAAACTCCTACGGGAGGCAGCAGTGGGGAATATTGCACAATGGGGGAAACCCTGATGCAGCAACGCCGCGTGAGTGAAGAAGTATTTCGGTATGTAAAGCTCTATCAGCAGGGAAGATAATGACGGTACCTGACTAAAGAAGCCCCGGCTAACTACGTGCCAGCAGCCGCGGTAA

+

::::::::::::::::::::::::::::::::::::::::::::::::::::::::::::::::::::::::::::::::::::::::::::::::::::::::::::::::::::::::::::::::::::::::::::::::::::::::::::::::::::::::::::::::::::::::::::::::::::::::::::::::::::::::::::::::::::::::::::::::::::::::::::::::::::::::::::::::::::::::::::::::::::::::::::::::::::::::::::::::::::::::::::::::::::::::::::::::::::::::::::::::::::::::::::::::::::::::::::::::::::::::::::::::::::::::::::::::::::::::::::::::::::::::::::::::::::::::::::::::::::::

@D0950.29_103891

GATGAACGCTAGCTACAGGCTTAACACATGCAAGTCGAGGGGTAGCATGAAACTTAGCAATAAGTTTTGATGACGACCGGCGCACGGGTGAGTAACACGTATCCAACCTGCCTTTTACTCATGGATAGCCTTCTGAAAAGAAGATTAATACATGATGGTATTCAGAGTTTTCATGGACACTGAATTAAAGATTTTATCGGTAAAGAGATGGGGATGCGTTCCATTAGATAGTAGGCGGGGTAACGGCCCACCTAGTCAACGATGGATAGGGGTTCTGAGAGGAAGGTCCCCACATTGGAACTGAGACACGGTCCAAACGTCCTACGGGAGGCAGCAGTGAGGAATATTGGTCAATGGACGTAAGTCTGAACCAGCCAAGTAGCGTGAAGGATGAAGGCTCTATGGGTCGTAAAACTTCTTTTTATAAAAGGAAATAAAGTATGCCACGTGTGGTGTTTTTGTATGTACTTTATGAATAAGGATCGGCTAAACTCCGTGCCAGCAGCCGC

+

:::::::::::::::::::::::::::::::::::::::::::::::::::::::::::::::::::::::::::::::::::::::::::::::::::::::::::::::::::::::::::::::::::::::::::::::::::::::::::::::::::::::::::::::::::::::::::::::::::::::::::::::::::::::::::::::::::::::::::::::::::::::::::::::::::::::::::::::::::::::::::::::::::::::::::::::::::::::::::::::::::::::::::::::::::::::::::::::::::::::::::::::::::::::::::::::::::::::::::::::::::::::::::::::::::::::::::::::::::::::::::::::::::::::::::::::::::::::::::::::::::::::::::::::::::::::::::::

@D0950.29_104037

GATGAACGCTGGCGGCGTGCTTAACACATGCAAGTCGAACGAAGCAACTTTCTTGCTTGCAAGAAAGTTGACTGAGTGGCGGACGGGTGAGTAACGCGTGGGTAACCTGCCTCATACAGGGGGATAACAGTTAGAAATGACTGCTAACACCGCATAACCCGCTAGTGTCGCATGACACGGACGGAAAATATTTATAGGTATGAGATGGGCCCGCGTCTGATTAGCTAGTTGGTAAGGTAACGGCTTACCAAGGCGACGATCAGTAGCCGACTTGAGAGAGTGATCGGCCACATTGGGACTGAGACACGGCCCAAACTCCTACGGGAGGCAGCAGTGGGAATATTGGACAATGGGGGAAACCCTGATCCAGCGACGCCGCGTGAGTGAAGAAGTATTTCGGTATGTAAAGCTCTATCAGCAGGGGAAGATAATGACAGTACCTGACTAAGAAGCCCCGGCTAACTACGTGCCAGCAGCCGCGGTAA

+

:::::::::::::::::::::::::::::::::::::::::::::::::::::::::::::::::::::::::::::::::::::::::::::::::::::::::::::::::::::::::::::::::::::::::::::::::::::::::::::::::::::::::::::::::::::::::::::::::::::::::::::::::::::::::::::::::::::::::::::::::::::::::::::::::::::::::::::::::::::::::::::::::::::::::::::::::::::::::::::::::::::::::::::::::::::::::::::::::::::::::::::::::::::::::::::::::::::::::::::::::::::::::::::::::::::::::::::::::::::::::::::::::::::::::::::::::::::::::::::::::::::

@D0950.29_104071

GACGAACGCTGGCGGCGTGCCTAACACATGCAAGTCGAGCGATGAAGTTCTTCGGAACGGATTAGCGGCGGACGGGTGAGTAACACGTGGGTAACCTGCCTTGTAGAGGGGGATAGCCTTCCGAAAGGAAGATTAATACCGCATAACATCTTTTTATCGCATGGTAGAAAGATCAAAGGAGCAATCCGCTACAAGATGGACCCGCGGCGCATTAGCTAGTTGGTGAGGTAACGGCTCACCAAGGCGACGATGCGTAGCCGACCTGAGAGGGTGATCGGCCACATTGGAACTGAGACACGGTCCAGACTCCTACGGGAGGCAGCAGTGGGGAATATTGCGCAATGGGGGAAACCCTGACGCAGCAACGCCGCGTGAATGAAGAAGGCCTTAGGGTTGTAAAGTTCTGTTTACGGGGACGATAATGACGGTACCCGTGGAGGAAGCCACGGCTAACTACGTGCCAGCAGCCGCGGTAA

+

::::::::::::::::::::::::::::::::::::::::::::::::::::::::::::::::::::::::::::::::::::::::::::::::::::::::::::::::::::::::::::::::::::::::::::::::::::::::::::::::::::::::::::::::::::::::::::::::::::::::::::::::::::::::::::::::::::::::::::::::::::::::::::::::::::::::::::::::::::::::::::::::::::::::::::::::::::::::::::::::::::::::::::::::::::::::::::::::::::::::::::::::::::::::::::::::::::::::::::::::::::::::::::::::::::::::::::::::::::::::::::::::::::::::::::::::::::::::::::

@D0950.29_104149

GATGAACGCTAGCGACAGGCCTAACACATGCAAGTCGAGGGGTAGCACAAGGTAGCAATACTGAGGTGACGACCGGCGCACGGGTGAGTAACGCGTATGCAACCTACCTGTAAGAGTGGGATAGCCTCTCGAAAGAGAGATTAATACCGCATAATACCATTTCACTGCATGGTGAGATGGTTAAAGATTTATTGCTTACAGATGGGCATGCGTAACATTAGCTTGTTGGTGAGGTAACGGCTCACCAAGGCAACGATGTTTAGGGGTTCTGAGAGGAAGGTCCCCCACACTGGTACTGAGACACGGACCAGACTCCTACGGGAGGCAGCAGTGAGGAATATTGGTCAATGGACGAGAGTCTGAACCAGCCAAGTCGCGTGAAGGATGAAGGTCTTATGGATTGTAAACTTCTTTTATACGGGAATAAAAATGCCACGTGTGGCATATTGCATGTACCGTATGAATAAGGATCGGCT

+

::::::::::::::::::::::::::::::::::::::::::::::::::::::::::::::::::::::::::::::::::::::::::::::::::::::::::::::::::::::::::::::::::::::::::::::::::::::::::::::::::::::::::::::::::::::::::::::::::::::::::::::::::::::::::::::::::::::::::::::::::::::::::::::::::::::::::::::::::::::::::::::::::::::::::::::::::::::::::::::::::::::::::::::::::::::::::::::::::::::::::::::::::::::::::::::::::::::::::::::::::::::::::::::::::::::::::::::::::::::::::::::::::::::::::::::::::::::::::::

@D0950.29_104155

GATGAACGCTGGCGGCGTGCTTAACACATGCAAGTCGAACGAAGCAGCTTTCTTGCTTGCAAGAAAGCTGACTTAGTGGCGGACGGGTGAGTAACGCGTGGGTAACCTGCCTCATACAGGGGGATAACAGTTGGAAACGACTGCTAAGACCGCATAACCCGCTAGTGTCGCATGACACGGACGGAAAATATTTTATAGGTATGAGATGGGCCCGCGTCTGATTAGCTAGTTGGTAAGGTAACGGCTTACCAAGGCGACGATCAGTAGCCGACTTGAGAGAGTGATCGGCCACATTGGGACTGAGACACGGCCCAAACTCCTACGGGAGGCAGCAGTGGGGAATATTGGACAATGGGGGAAACCCTGATCCAGCGACGCCGCGTGAGTGAAGAAGTATTTCGGTATGTAAAGCTCTATCAGCAGGGAAGATAATGACAGTACCTGACTAAGAAGCCCCCGGCTAACTACGTGCCAGCAGCCGC

+

::::::::::::::::::::::::::::::::::::::::::::::::::::::::::::::::::::::::::::::::::::::::::::::::::::::::::::::::::::::::::::::::::::::::::::::::::::::::::::::::::::::::::::::::::::::::::::::::::::::::::::::::::::::::::::::::::::::::::::::::::::::::::::::::::::::::::::::::::::::::::::::::::::::::::::::::::::::::::::::::::::::::::::::::::::::::::::::::::::::::::::::::::::::::::::::::::::::::::::::::::::::::::::::::::::::::::::::::::::::::::::::::::::::::::::::::::::::::::::::::::

@D0950.29_104238

GATGAACGCTAGCGACAGGCCTAACACATGCAAGTCGAGGGGCAACGGGAGTGTAGCAATACACTTGCCGGCGACCGGCGCACGGGTGAGTAACACGTATGCGACCTACCCATAGCAGGGGGATAATCGGAAGAAATTCCGTCTAATACCGCGTAATAATTCAGATCTGCATGGATTTGAATTTAAAGGAGCAATCCGGCTATGGATGGGCATGCGGGACATTAGCTAGTTGGCGGGGTAACGGCCCACCAAGGCTTCGATGTCTAGGGGTTCTGAGAGGAAGGTCCCCCACACTGGTACTGAGACACGGACCAGACTCCTACGGGAGGCAGCAGTGAGGAATATTGGTCAATGGTCGAGAGACTGAACCAGCCAAGTCGCGTGAGGGATGAAGGTTCTATGGATTGTAAAACCTCTTTTGTCAGGGAGCAACGGCATCCACGAGTGGATGAATGAGAGTACCTGAAGAAAAAGCATCGGCTAACTCCGTGCCAGCAGCCGCGGTAA

+

:::::::::::::::::::::::::::::::::::::::::::::::::::::::::::::::::::::::::::::::::::::::::::::::::::::::::::::::::::::::::::::::::::::::::::::::::::::::::::::::::::::::::::::::::::::::::::::::::::::::::::::::::::::::::::::::::::::::::::::::::::::::::::::::::::::::::::::::::::::::::::::::::::::::::::::::::::::::::::::::::::::::::::::::::::::::::::::::::::::::::::::::::::::::::::::::::::::::::::::::::::::::::::::::::::::::::::::::::::::::::::::::::::::::::::::::::::::::::::::::::::::::::::::::::::::::::::

@D0950.29_104248

GACGAACGCTGGCGGCGTGCCTAACACATGCAAGTCGAGCGAGTTGATCCCTTCGGGGTGAAGCTAGCGGCGGACGGGTGAGTAACACGTGGGCAACCTGCCTCATAGAGGGGAATAGCCTTCCGAAAGGGAGATTAATACCGCATAAGATTGTAGCTTCGCATGAAGTAGCAATTAAAGGAGCAAATCCGCTATGAGATGGGCCCCGCGGCGCATTAGCTAGTTGGTGAGGTAACGGCTCACCAAGGCGACGATGCGTAGCCGACCTGAGAGGGGTGATCGGCCACATTGGGGACTGAGACACGGCCCAGACTCCTACGGGAGGCAGCAGTGGGGAATATTGCACAATGGGGGAAACCCTGATGCAGCAACGCCGCGTGAGTGATGAAGGCCTTCGGGTTGTAAAGCTCTGTCTTCAGGGACGATAATGACGGTACCTGAGGAGGAAGCCACGGCTAACTACGTG

+

::::::::::::::::::::::::::::::::::::::::::::::::::::::::::::::::::::::::::::::::::::::::::::::::::::::::::::::::::::::::::::::::::::::::::::::::::::::::::::::::::::::::::::::::::::::::::::::::::::::::::::::::::::::::::::::::::::::::::::::::::::::::::::::::::::::::::::::::::::::::::::::::::::::::::::::::::::::::::::::::::::::::::::::::::::::::::::::::::::::::::::::::::::::::::::::::::::::::::::::::::::::::::::::::::::::::::::::::::::::::::::::::::::::::::::::::::

@D0950.29_104251

GATGAACGCTAGCGACAGGCCTAACACATGCAAGTCGAGGGGTAGCACAAGGTAGCAATACTGAGGTGACGACCGGCGCACGGGTGAGTAACGCGTATGCAACCTACCTGTAAGAGTGGGATAGCCTCTCGAAAGAGAGATTAATACCGCATAATACCATTTCACTGCATGGTGAGATGGTTAAAGATTTATTGCTTACAGATGGGCATGCGTAACATTAGCTAGTTGGTGAGGTAACGGCTCACCAAGGCACGATGTTTAGGGGTTCTGAGAGGAAGGTCCCCCACACTGGTACTGAGACACGGACCAGACTCCTACGGGAGGCAGCAGTGAGGAATATTGGTCAATGGACGAGAGTCTGAACCAGCCAAGTCGCGTGAAGGATGAAGGTCTTATGGATTGTAAACTTCTTTTATACGGGAATAAAAAATGCCACGTGTGGCATATTGCATGTACCGTATGAATAAGGATCGGCT

+

::::::::::::::::::::::::::::::::::::::::::::::::::::::::::::::::::::::::::::::::::::::::::::::::::::::::::::::::::::::::::::::::::::::::::::::::::::::::::::::::::::::::::::::::::::::::::::::::::::::::::::::::::::::::::::::::::::::::::::::::::::::::::::::::::::::::::::::::::::::::::::::::::::::::::::::::::::::::::::::::::::::::::::::::::::::::::::::::::::::::::::::::::::::::::::::::::::::::::::::::::::::::::::::::::::::::::::::::::::::::::::::::::::::::::::::::::::::::::::

@D0950.29_104274

GATGAACGCTAGCGACAGGCCTAACACATGCAAGTCGAGGGGTAGCACAAGGAAGCTTGCTTCTGAGGTGACGACCGGCGCACGGGTGAGTAACGCGTATGCAACCTGCCTATAAGAAGGGGATAGCCTCTCGAAAGAGAGATTAATACCGTATAACACTATGAAGCCGCATGGTTTTATAGTTAAAGATTTATTGCTTATAGATGGGCATGCGTAACATTAGCTAGTTGGTAAGGTAACGGCTTACCAAGGCAACGATGTTTAGGGGGCCTGAGAGGGTGATCCCCCACACTGGTACTGAGACACGGACCAGACTCCTACGGGAGGCAGCAGTGAGGAATATTGGACAATGGGTGAGAGCCTGATCCAGCCATCCCGCGTGAAGGACGACTGCCCTATGGGTTGTAAACTTCTTTTATACTGGGGATAAACCTACCCTCGTGAGGGTAGCTGAAGGTACAGTATGAATAAGCACCGGCTAACTCCGTGCCAGCAGCCGC

+

::::::::::::::::::::::::::::::::::::::::::::::::::::::::::::::::::::::::::::::::::::::::::::::::::::::::::::::::::::::::::::::::::::::::::::::::::::::::::::::::::::::::::::::::::::::::::::::::::::::::::::::::::::::::::::::::::::::::::::::::::::::::::::::::::::::::::::::::::::::::::::::::::::::::::::::::::::::::::::::::::::::::::::::::::::::::::::::::::::::::::::::::::::::::::::::::::::::::::::::::::::::::::::::::::::::::::::::::::::::::::::::::::::::::::::::::::::::::::::::::::::::::::::::::::::

@D0950.29_104393

ATTGAACGCTGGCGGCATGCCTTACACATGCAAGTCGAACGGTAACAGGTCTTCGGATGCTGACGAGTGGCGAACGGGTGAGTAATACATCGGAACGTGCCCGATCGTGGGGGATAACGAAGCGAAAGCTTTGCTAATACCGCATACGATCTACGGATGAAAGCAGGGGACCGCAAGGCCTTGCGCGAACGGAGCGGCCGATGGCAGATTAGGTAGTTGGTGGGATAAAAGCTTACCAAGCCGACGATCTGTAGCTGGTCTGAGAGGACGACCAGCCACACTGGGACTGAGACACGGCCCAGACTCCTACGGGAGGCAGCAGTGGGGAATTTTGGACAATGGGCGAAAGCCTGATCCAGCCATGCCGCGTGCAGGATGAAGGCCTTCGGGTTGTAAACTGCTTTTGTACGGAACGAAAAGACTCTGGTTAATACCTGGGGGTCCATGACGGTACCGTAAGAATAAGCACCGGCTAACTACGTGCCAGCAGCCGCGGTAA

+

:::::::::::::::::::::::::::::::::::::::::::::::::::::::::::::::::::::::::::::::::::::::::::::::::::::::::::::::::::::::::::::::::::::::::::::::::::::::::::::::::::::::::::::::::::::::::::::::::::::::::::::::::::::::::::::::::::::::::::::::::::::::::::::::::::::::::::::::::::::::::::::::::::::::::::::::::::::::::::::::::::::::::::::::::::::::::::::::::::::::::::::::::::::::::::::::::::::::::::::::::::::::::::::::::::::::::::::::::::::::::::::::::::::::::::::::::::::::::::::::::::::::::::::::::::

@D0950.29_104448

GATGAACGCTGGCGGCGTGCTTAACACATGCAAGTCGAACGAAGCACTTAAGGAGCTTGCTCCAAAGGTGACTGAGTGGCGGACGGGTGAGTAACGCGTGGGTAACCTGCCTTACACTGGGGGATAACAGTTGGAAACGACTGCTAATACCGCATAAGCGCACAGTATTGCATGATACAGTGTGAAAAACTCCGGTGGTGTAAGATGGACCCGCGTCTGATTAGCTAGTTGGTGAGGTAATGGCTCACCAAGGCAACGATCAGTAGCCGGCTTGAGAGAGTGAACGGCCACATTGGGACTGAGACACGGCCCAAACTCCTACGGGAGGCAGCAGTGGGGAATATTGCACAATGGGGGAAACCCTGATGCAGCAACGCCGCGTGAGTGAAGAAGTATTTGCGGTATGTAAAGCTCTATCAGCAGGGAAGATAATGACGGTACCTGACTAAGAAGCCCCGGCTAACTACGTGCCAGCAGCCGC

+

:::::::::::::::::::::::::::::::::::::::::::::::::::::::::::::::::::::::::::::::::::::::::::::::::::::::::::::::::::::::::::::::::::::::::::::::::::::::::::::::::::::::::::::::::::::::::::::::::::::::::::::::::::::::::::::::::::::::::::::::::::::::::::::::::::::::::::::::::::::::::::::::::::::::::::::::::::::::::::::::::::::::::::::::::::::::::::::::::::::::::::::::::::::::::::::::::::::::::::::::::::::::::::::::::::::::::::::::::::::::::::::::::::::::::::::::::::::::::::::::::

@D0950.29_104463

GATGAACGCTAGCGACAGGCCTAACACATGCAAGTCGAGGGGTAGCACAAGGTAGCAATACTGAGGTGACGACCGGCGCACGGGTGAGTAACGCGTATGCAACCTACCTGTAAGAGTGGGATAGCCTCTCGAAAGAGAGATTAATACCGCATAATACCATTTCACTGCATGGTGAGATGGTTAAAGATTTATTGCTTACAGATGGGCATGCGTAACATTAGCTAGTTGGTGAGGTAACGGCTCACCAAGGCAACGATGTTTAGGGGTTCTGAGAGGAAGGTCCCCCACACTGGTACTGAGACACGGACCAGACTCCTACGGGAGGCAGCAGTGAGGAATATTGGTCAATGGACGAGAGTCTGAACCAGCCAAGTCGCGTGAAGGATGAAGGTCTTATGGATTGTAAACTTCTTTTATACGGGAATAAAAATGCCACGTGTGGCATATTGCATGTACCGTATGAATAAGGATCGGCTAACTCCGTGCCAGCAGCCGCGGTAA

+

:::::::::::::::::::::::::::::::::::::::::::::::::::::::::::::::::::::::::::::::::::::::::::::::::::::::::::::::::::::::::::::::::::::::::::::::::::::::::::::::::::::::::::::::::::::::::::::::::::::::::::::::::::::::::::::::::::::::::::::::::::::::::::::::::::::::::::::::::::::::::::::::::::::::::::::::::::::::::::::::::::::::::::::::::::::::::::::::::::::::::::::::::::::::::::::::::::::::::::::::::::::::::::::::::::::::::::::::::::::::::::::::::::::::::::::::::::::::::::::::::::::::::::::::::::::

@D0950.29_104469

GATGAACGCTGGCGGCGTGCTTAACACATGCAAGTCGAACGAAGCACTTAAGGAGCTTGCTCCAAAGGTGACTGAGTGGCGGACGGGTGAGTAACGCGTGGGTAACCTGCCTTACACTGGGGGATAACAGTTGGAAACGACTGCTAATACCGCATAAGCGCACAGTATTGCATGATACAGTGTGAAAAACTCCGGTGGTGTAAGATGGACCCGCGTCTGATTAGCTAGTTGGTGAGGTAATGGCTCACCAAGGCAACGATCAGTAGCCGGCTTGAGAGAGTGAACGGCCACATTGGGACTGAGACACGGCCCAAACTCCTACGGAGGCAGCAGTGGGGAATATTGCACAATGGGGGAAACCCTGATGCAGCAACGCCGCGTGAGTGAAGAAGTATTTGCGGTATGTAAAGCTCTATCAGCAGGGAAGATAATGACGGTACCTGACTAAGAAGCCCCCGGCTAACTACGTGCCAGCAGCCG

+

::::::::::::::::::::::::::::::::::::::::::::::::::::::::::::::::::::::::::::::::::::::::::::::::::::::::::::::::::::::::::::::::::::::::::::::::::::::::::::::::::::::::::::::::::::::::::::::::::::::::::::::::::::::::::::::::::::::::::::::::::::::::::::::::::::::::::::::::::::::::::::::::::::::::::::::::::::::::::::::::::::::::::::::::::::::::::::::::::::::::::::::::::::::::::::::::::::::::::::::::::::::::::::::::::::::::::::::::::::::::::::::::::::::::::::::::::::::::::::::::

@D0950.29_104492

GATGAACGCTGGCGGCGTGCTTAACACATGCAAGTCGAACGAAGATTAGGGAGCTTGCTCATTAATACTTAGTGGCGGACGGGTGAGTAACGCGTGGGTAACCTACCCTATGCAGGGGGATAACGTTTGGAAACGAACGCTAATACCGCATAAACTATCGATAGTCGCATGACTATTATAGCAAAGATGCAGACTTTAGTCTGTTTTCAGCATAGGATGGACCTGCGTTGGATTAGCTAGTTGGTGAGATAACAGCCCACCAAGGCAACGATCCATAGCCGGCCTGAGAGGGTGAACGGCCACATTGGGGACTGAGACACGGCCCAAACTCCTACGGGAGGCAGCAGTGGGGAATATTGCACAATGGGCGAAAGCCTGATGCAGCAACGCCGCGTGAAGGATGAAGGTCTTCGGATTGTAAACTTCTATCAGTAGGGAAGAAAGGTAACTTCGGTTACCCTGACGGTACCTAACTAAGAAGCTCCGGCTAACTACGTGCCAGCAGCCGCGGTAA

+

::::::::::::::::::::::::::::::::::::::::::::::::::::::::::::::::::::::::::::::::::::::::::::::::::::::::::::::::::::::::::::::::::::::::::::::::::::::::::::::::::::::::::::::::::::::::::::::::::::::::::::::::::::::::::::::::::::::::::::::::::::::::::::::::::::::::::::::::::::::::::::::::::::::::::::::::::::::::::::::::::::::::::::::::::::::::::::::::::::::::::::::::::::::::::::::::::::::::::::::::::::::::::::::::::::::::::::::::::::::::::::::::::::::::::::::::::::::::::::::::::::::::::::::::::::::::::::::::::

@D0950.29_104516

GATGAACGCTAGCTACAGGCTTAACACATGCAAGTCGAGGGGTAGCATGAAACTTAGCAATAAGTTTTGATGACGACCGGCGCACGGGTGAGTAACACGTATCCAACCTGCCTTTTACTCATGGATAGCCTTCTGAAAAGAAGATTAATACATGATGGTATTCAGAGTTTTCATGGACACTGAATTAAAGATTTATCGGTAAGAGATGGGGATGCGTTCCATTAGATAGTAGGCGGGGTAACGGCCCACCTAGTCAACGATGGATAGGGGTTCTGAGAGGAAGGTCCCCCACATTGGAACTGAGACACGGTCCAAACGTCCTACGGGAGGCAGCAGTGAGGAATATTGGTCAATGGACGTAAGTCTGAACCAGCCAAGTAGCGTGAAGGATGAAGGCTC

+

:::::::::::::::::::::::::::::::::::::::::::::::::::::::::::::::::::::::::::::::::::::::::::::::::::::::::::::::::::::::::::::::::::::::::::::::::::::::::::::::::::::::::::::::::::::::::::::::::::::::::::::::::::::::::::::::::::::::::::::::::::::::::::::::::::::::::::::::::::::::::::::::::::::::::::::::::::::::::::::::::::::::::::::::::::::::::::::::::::::::::::::::::::::::::::::::::::::::::::::::

@D0950.29_104640

GATGAACGCTAGCTACAGGCTTAACACATGCAAGTCGAGGGGTAGCATGAAACTTAGCAATAAGTTTTGATGACGACCGGCGCACGGGTGAGTAACACGTATCCAACCTGCCTTTTACTCATGGATAGCCTTCTGAAAAGAAGATTAATACATGATGGTATTCAGAGTTTTCATGGACACTGAATTAAAGATTTTATCGGTAAGAGATGGGGATGCGTTCCATTAGATAGTAGGCGGGGTAACGGCCCACCTAGTCAACATGGATAGGGGTTCTGAGAGGAAGGTCCCCACATTGGAACTGAGACACGGTCCAAACGTCCGTACGGGAGGCAGCAGTGAGGAATATTGGTCAATGGACGTAAGTCTGAACCAGCCAAGTAGCGTGAA

+

:::::::::::::::::::::::::::::::::::::::::::::::::::::::::::::::::::::::::::::::::::::::::::::::::::::::::::::::::::::::::::::::::::::::::::::::::::::::::::::::::::::::::::::::::::::::::::::::::::::::::::::::::::::::::::::::::::::::::::::::::::::::::::::::::::::::::::::::::::::::::::::::::::::::::::::::::::::::::::::::::::::::::::::::::::::::::::::::::::::::::::::::::::::::::::::::::::

@D0950.29_104666

GATGAACGCTGGCGGCGTGCTTAACACATGCAAGTCGAACGAACTGCGAGGAGCTTGCTCCTCAAAGTTAGTGGCGGACGGGTGAGTAACGCGTGGGTAACCTGCCTCATACAGGGGATAACAGTTGGAAACGACTGCTAAGACCGCATAACCCGCTAGTGTCGCATGACACGGACGGAAAATATTTATAGGTATGAGATGGGCCCGCGTCTGATTAGCTAGTTGGTAAGGTAACGGCTTACCAAGGCGACGATCAGTAGCCGACTTGAGAGAGTGATCGGCCACATTGGGACTGAGACACGGCCCAAACTCCTACGGGAGGCAGCAGTGGGGAATATTGGACAATGGGGAAACCCTGATCCAGCGACGCCGCGTGAGTGAAGAAGTATTTCGGTATGTAAAGCTCTATCAGCAGGGGAAGATAATGACAGTACCTGACTAAGAAGCCCCGGCTAACTACGTGCCAGCAGCCGCGGTAA

+

:::::::::::::::::::::::::::::::::::::::::::::::::::::::::::::::::::::::::::::::::::::::::::::::::::::::::::::::::::::::::::::::::::::::::::::::::::::::::::::::::::::::::::::::::::::::::::::::::::::::::::::::::::::::::::::::::::::::::::::::::::::::::::::::::::::::::::::::::::::::::::::::::::::::::::::::::::::::::::::::::::::::::::::::::::::::::::::::::::::::::::::::::::::::::::::::::::::::::::::::::::::::::::::::::::::::::::::::::::::::::::::::::::::::::::::::::::::::::::::::

@D0950.29_104675

GATGAACGCTAGCGACAGGCCTAACACATGCAAGTCGAGGGGTAGCACAAGGTAGTAATACTGAGGTGACGACCGGCGCACGGGTGAGTAACGCGTATGCAACCTACCTGTAAGAGTGGGATAGCCTCTCGAAAGAGAGATTAATACCGCATAATACCATTTCACTGCATGGTGAGATGGTTAAAGATTTATTGCTTACAGATGGGCATGCGTAACATTAGCTAGTTGGTGAGGTAACGGCTCACCAAGGCAACGATGTTTAGGGGTTCTGAGAGGAAGGTCCCCCACACTGGTACTGAGACACGGACCAGACTCCTACGGGAGGCAGCAGTGAGGAATATTGGTCAATGGACGAGAGTCTGAACCAGCCAAGTCGCGTGAAGGATGAAGGTCTTATGGATTGTAAACTTCTTTATACGGGAATAAAAATGCCACGTGTGGCATATTGCATGTACCGTATGAATAAGGATCGGCTAACTCCGTGCCAGCAGCCGCGGTAA

+

::::::::::::::::::::::::::::::::::::::::::::::::::::::::::::::::::::::::::::::::::::::::::::::::::::::::::::::::::::::::::::::::::::::::::::::::::::::::::::::::::::::::::::::::::::::::::::::::::::::::::::::::::::::::::::::::::::::::::::::::::::::::::::::::::::::::::::::::::::::::::::::::::::::::::::::::::::::::::::::::::::::::::::::::::::::::::::::::::::::::::::::::::::::::::::::::::::::::::::::::::::::::::::::::::::::::::::::::::::::::::::::::::::::::::::::::::::::::::::::::::::::::::::::::::::

@D0950.29_104705

ATTGAACGCTGGCGGCAGGCCTAACACATGCAAGTCGAACGGTAGCACAGAGAGCTTGCTCTTGGGTGACGAGTGGCGGACGGGTGAGTAATGTCTGGGAAACTGCCCGATGGAGGGGGATAACTACTGGAAACGGTAGCTAATACCGCATAATGTCGCAAGACCAAAGAGGGGGACCTTCGGGCCTCTTGCCATCGGATGTGCCCAGATGGGATTAGCTAGTAGGTGGGGTAATGGCTCACCTAGGCGACGATCCCTAGCTGGTCTGAGAGGATGACCAGCCACACTGGAACTGAGACACGGTCCAGACTCCTACGGGAGGCAGCAGTGGGGAATATTGCACAATGGGCGCAAGCCTGATGCAGCCATGCCGCGTGTATGAAGAAGGCCTTCGGGTTGTAAAGTACTTTCAGCGAGGAGGAAGGTGTTGAGGTTAATAACCTCAGCAATTGACGTTACTCGCAGAAGAAGCACCGGCTAACTCCGTGCCAGCAGCCGCGGTAA

+

::::::::::::::::::::::::::::::::::::::::::::::::::::::::::::::::::::::::::::::::::::::::::::::::::::::::::::::::::::::::::::::::::::::::::::::::::::::::::::::::::::::::::::::::::::::::::::::::::::::::::::::::::::::::::::::::::::::::::::::::::::::::::::::::::::::::::::::::::::::::::::::::::::::::::::::::::::::::::::::::::::::::::::::::::::::::::::::::::::::::::::::::::::::::::::::::::::::::::::::::::::::::::::::::::::::::::::::::::::::::::::::::::::::::::::::::::::::::::::::::::::::::::::::::::::::::

@D0950.29_10479

GATGAACGCTGGCGGCGTGCTTAACACATGCAAGTCGAGCGAGGAATCACCTTCGGGTGTGAACTAGCGGCGGACGGGTGAGTAACACGTGGGCAACCTGCCTTACAGAGGGGGATAGCCTTCCGAAAGGAAGATTAATACCGCATATTATGAGTTTTCTGCATGGAGAATTCATGAAAGGAGTAATCCGCTGTAAGATGGGCCCGCGGCGCATTAGCTAGTTGGTGAGGTAACGGCTCACCAAGGCGACGATGCGTAGCCGACCTGAGAGGGTGATCGGCCACATTGGGACTGAGACACGGCCCAGACTCCTACGGGAGGCAGCAGTGGGGAATATTGCACAATGGGGGAAACCCTGATGCAGCAACGCCGCGTGAGTGATGAAGGCCTTCGGGTTGTAAAAGCTCTGTCTTCAGGGACGATAATGACGGTACCTGAGGAGAAGCCACGGCTAACTACGTGCCAGCAGCCGCGGTAA

+

::::::::::::::::::::::::::::::::::::::::::::::::::::::::::::::::::::::::::::::::::::::::::::::::::::::::::::::::::::::::::::::::::::::::::::::::::::::::::::::::::::::::::::::::::::::::::::::::::::::::::::::::::::::::::::::::::::::::::::::::::::::::::::::::::::::::::::::::::::::::::::::::::::::::::::::::::::::::::::::::::::::::::::::::::::::::::::::::::::::::::::::::::::::::::::::::::::::::::::::::::::::::::::::::::::::::::::::::::::::::::::::::::::::::::::::::::::::::::::::

@D0950.29_104828

GATGAACGCTAGCTACAGGCTTAACACATGCAAGTCGAGGGGTAGCATGAAACTTAGCAATAAGTTTTGATGACGACCGGCGCACGGGTGAGTAACACGTATCCAACCTGCCTTTTACTCATGGATAGCCTTCTGAAAAGAAGATTAATACATGATGGTATTCAGAGTTTTCATGGACACTGAATTAAAGATTTTATCGGTAAGAGATGGGGATGCGTTCCATTAGATAGTAGGCGGGGTAACGGCCCACCTAGTCAACGATGGATAGGGGTTCTGAGAGGAAGGTCCCCCACATTGGAACTGAGACACGGTCCAAACGTCCTACGGGAGGCAGCAGTGAGGAATATTGGTCAATGGACGTAAGTCTGAACCAGCCAAGTAGCGTGAAGGATGAAGGCTCTATGGGTCGTAAACTTCTTTTTATAAAAGGAATAAAGTATGCCACGTGTGGTGTTTTTGTATGTACTTTATGAATAAGGATCGGCTAACTCCGTGCCAGCAGCCGC

+

::::::::::::::::::::::::::::::::::::::::::::::::::::::::::::::::::::::::::::::::::::::::::::::::::::::::::::::::::::::::::::::::::::::::::::::::::::::::::::::::::::::::::::::::::::::::::::::::::::::::::::::::::::::::::::::::::::::::::::::::::::::::::::::::::::::::::::::::::::::::::::::::::::::::::::::::::::::::::::::::::::::::::::::::::::::::::::::::::::::::::::::::::::::::::::::::::::::::::::::::::::::::::::::::::::::::::::::::::::::::::::::::::::::::::::::::::::::::::::::::::::::::::::::::::::::::::

@D0950.29_104876

GATGAACGCTAGCGACAGGCCTAACACATGCAAGTCGAGGGGTAGCACAAGGTAGCAATACTGAGGTGACGACCGGCGCACGGGTGAGTAACGCGTATGCAACCTACCTGTAAGAGTGGGATAGCCTCTCGAAAGAGAGATTAATACCGCATAATACCATTTCACTGCATGGTGAGATGGTTAAAGATTTATTGCTTACAGATGGGCATGCGTAACATTAGCTTGTTGGTGAGGTAACGGCTCACCAAGGCAACGATGTTTAGGGGTTCTGAGAGGAAGGTCCCCCACACTGGTACTGAGACACGGACCAGACTCCTACGGGAGGCAGCAGTGAGGAATATTGGTCAATGGACGAGAGTCTGAACCAGCCAAGTCGCGTGAAGGATGAAGGTCTTATGGATTGTAAACTTCTTTTATACGGGAATAAAAATGCCACGTGTGGCATATTGCATGTACCGTATGAATAAGGATCGGCTAACTCCGTGCCAGCAGCCGCGGTAA

+

:::::::::::::::::::::::::::::::::::::::::::::::::::::::::::::::::::::::::::::::::::::::::::::::::::::::::::::::::::::::::::::::::::::::::::::::::::::::::::::::::::::::::::::::::::::::::::::::::::::::::::::::::::::::::::::::::::::::::::::::::::::::::::::::::::::::::::::::::::::::::::::::::::::::::::::::::::::::::::::::::::::::::::::::::::::::::::::::::::::::::::::::::::::::::::::::::::::::::::::::::::::::::::::::::::::::::::::::::::::::::::::::::::::::::::::::::::::::::::::::::::::::::::::::::::::

@D0950.29_104879

GATGAACGCTAGCGACAGGCCTAACACATGCAAGTCGAGGGGTAGCACAAGGTAGCAATACTGAGGTGACGACCGGCGCACGGGTGAGTAACGCGTATGCAACCTACCTGTAAGAGTGGGATAGCCTCTCGAAAGAGAGATTAATACCGCATAATACCATTTTACTGCATGGTGAGATGGTTAAAGATTTGTTGCTTACAGATGGGCATGCGTAACATTAGCTAGTTGGTGAGGTAACGGCTCACCAAGGCAACGATGTTTAGGGGTTCTGAGAGGAAGGTCCCCCACACTGGTACTGAGACACGGACCAGACTCCTACGGGAGGCAGCAGTGAGGAATATTGGTCAATGGACGAGAGTCTGAACCAGCCAAGTCGCGTGAAGGATGAAGGTCTTATGGATTGTAAACTTCTTTTATACGGGAATAAAAAATACCACGTGTGGTATATTGCATGTACCGTATGAATAAGGATCGGCTAACTCCGT

+

:::::::::::::::::::::::::::::::::::::::::::::::::::::::::::::::::::::::::::::::::::::::::::::::::::::::::::::::::::::::::::::::::::::::::::::::::::::::::::::::::::::::::::::::::::::::::::::::::::::::::::::::::::::::::::::::::::::::::::::::::::::::::::::::::::::::::::::::::::::::::::::::::::::::::::::::::::::::::::::::::::::::::::::::::::::::::::::::::::::::::::::::::::::::::::::::::::::::::::::::::::::::::::::::::::::::::::::::::::::::::::::::::::::::::::::::::::::::::::::::::::::

@D0950.29_104987

GATGAACGCTAGCTACAGGCTTAACACATGCAAGTCGAGGGGTAGCATGAAACTTAGCAATAAGTTTTGATGACGACCGGCGCACGGGTGAGTAACACGTATCCAACCTGCCTTTTACTCATGGATAGCCTTCTGAAAAGAAGATTAATACATGATGGTATTCAGAGTTTTCATGGACACTGAATTAAAGATTTTATCGGTAAGAGATGGGGATGCGTTCCATTAGATAGTAGGCGGGGTAACGGCCCACCTAGTCAACGATGGATAGGGGTTCTGAGAGGAAGGTCCCCCACATTGGAACTGAGACACGGTCCAAACGTCCTACGGGAGGCAGCAGTGAGGAATATTGGTCAATGGACGTAAGTCTGAACCAGCCAAGTAGCGTGAAGGATGAAGGCTCTATGGGTCGTAAACTTCTTTTTATAAAAGGAATAAAGTATGCCACGTGTGGTGTTTTTGTATGTACTTTATGAATAAGGATCGGCTAACTCCGTGCCAGCAGCCGC

+

::::::::::::::::::::::::::::::::::::::::::::::::::::::::::::::::::::::::::::::::::::::::::::::::::::::::::::::::::::::::::::::::::::::::::::::::::::::::::::::::::::::::::::::::::::::::::::::::::::::::::::::::::::::::::::::::::::::::::::::::::::::::::::::::::::::::::::::::::::::::::::::::::::::::::::::::::::::::::::::::::::::::::::::::::::::::::::::::::::::::::::::::::::::::::::::::::::::::::::::::::::::::::::::::::::::::::::::::::::::::::::::::::::::::::::::::::::::::::::::::::::::::::::::::::::::::::

@D0950.29_104999

ATTGAACGCTGGCGGCATGCTTTACACATGCAAGTCGAACGGCAGCACGGGCTTCGGCCTGGTGGCGAGTGGCGAACGGGTGAGTAATGCATCGGAACGTACCGTGTAGTGGGGGATAACGTAGCGAAAGTTACGCTAATACCGCATACGCCCCGAGGGGGAAAGTGGGGGACCGCAAGGCCTCACGCTATATGAGCGGCCGATGTCGGATTAGCTAGTTGGTAGGGTAAAGGCCTACCAAGGCGACGATCCGTAGCGGGTCTGAGAGGATGATCCGCCACACTGGGACTGAGACACGGCCCAGACTCCTACGGGAGGCAGCAGTGGGGAATTTTGGACAATGGGCGCAAGCCTGATCCAGCCATGCCGCGTGAGTGAAGAAGGCCTTCGGGTTGTAAAGCTCTTTCAGACGGAAAGAAATCTCCTGGGCGAATACCCTGGGAGGATGACGGTACCGTAAGAAGAAGCACCGGCTAACTACGTGCCAGCAGCCGCGGTAA

+

::::::::::::::::::::::::::::::::::::::::::::::::::::::::::::::::::::::::::::::::::::::::::::::::::::::::::::::::::::::::::::::::::::::::::::::::::::::::::::::::::::::::::::::::::::::::::::::::::::::::::::::::::::::::::::::::::::::::::::::::::::::::::::::::::::::::::::::::::::::::::::::::::::::::::::::::::::::::::::::::::::::::::::::::::::::::::::::::::::::::::::::::::::::::::::::::::::::::::::::::::::::::::::::::::::::::::::::::::::::::::::::::::::::::::::::::::::::::::::::::::::::::::::::::::::

@D0950.29_10502

GATGAACGCTAGCTACAGGCTTAACACATGCAAGTCGAGGGGTAGCATGAAGCTTAGCAATAAGCTTTGATGACGACCGGCGCACGGGTGAGTAACACGTATCCAACCTGCCTTTTACTCATGGATAGCCTTCTGAAAAGAAGATTAATACATGATGGTATTCAGCGTTTTCATGGACACTGAATTAAAGATTTATCGGTAAGAGATGGGGATGCGTTCCATTAGATAGTAGGCGGGGTAACGGCCCACCATAGTCAACGATGGATAGGGGTTCTGAGAGGAGGTCCCCCACATTGAACTGAGACACGGTCCAAACGTCCTACGGGAGGCAGCAGTGAGGAATATTGGTCAATGGACGTAAGTCTGAACCAGCCAAGTAGCGTGAAGGATGAAGGCTCTAT

+

:::::::::::::::::::::::::::::::::::::::::::::::::::::::::::::::::::::::::::::::::::::::::::::::::::::::::::::::::::::::::::::::::::::::::::::::::::::::::::::::::::::::::::::::::::::::::::::::::::::::::::::::::::::::::::::::::::::::::::::::::::::::::::::::::::::::::::::::::::::::::::::::::::::::::::::::::::::::::::::::::::::::::::::::::::::::::::::::::::::::::::::::::::::::::::::::::::::::::::::::::

@D0950.29_10503

GACGAACGCTGGCGGCGTGCCTAACACATGCAAGTCGAGCGAGTGGAGTTCTTCGGAACAAAGCTAGCGGCGGACGGGTGAGTAACACGTGGGCAACCTGCCTCATAGAGGGGAATAGCCTCCCGAAAGGGAGATTAATACCGCATAAGATTGTAGCTTCGCATGAAGTAGCAATTAAAGGAGCAATCCGCTATGAGATGGGCCCGCGGCGCATTAGCTAGTTGGTGAGGTAACGGCTCACCAAGGCGACGATGCGTAGCCGACCTGAGAGGGGTGATCCGGGCCCACATTTGGGGGAACCTGAGGACAACGGGCCCCAGACTCCTACGGGAGGCAGCATGGGGAATATTGCACAATGGGGGAAACCCTGATGCAGCAACGCCGCGTGAGTGATGACGGCCTTCGGGTTGTAAACTCTGTCTTTGGGGACGATAATGACGGTACCCAAGGAGGAAGCCACGGCTAACTACGTGCCAGCAGCCGCGGTAA

+

:::::::::::::::::::::::::::::::::::::::::::::::::::::::::::::::::::::::::::::::::::::::::::::::::::::::::::::::::::::::::::::::::::::::::::::::::::::::::::::::::::::::::::::::::::::::::::::::::::::::::::::::::::::::::::::::::::::::::::::::::::::::::::::::::::::::::::::::::::::::::::::::::::::::::::::::::::::::::::::::::::::::::::::::::::::::::::::::::::::::::::::::::::::::::::::::::::::::::::::::::::::::::::::::::::::::::::::::::::::::::::::::::::::::::::::::::::::::::::::::::::::::::

@D0950.29_105036

GATGAACGCTGGCGGCGTGCTTAACACATGCAAGTCGAACGAAGCAACTTTCTTGCTTGCAAGAAAGTTGACTGAGTGGCGGACGGGTGAGTAACGCGTGGGTAACCTGCCTCATACAGGGGGATAACAGTTAGAAATGACTGCTAACACCGCATAACCCGCTAGCATCGCATGATGCAGACGGAAAATATTTATAGGTATGAGATGGGCCCGCGTCTGATTAGCTAGTTGGTGGGGTAACAGCCTACCAAGGCAACGATCAGTAGCCGACTTGAGAGAGTGATCGGCCACATTGGGACTGAGACACGGCCCAAACTCCTACGGGAGGCAGCAGTGGGGAATATTGGACAATGGGGGAAACCCTGATCCAGCGACGCCGCGTGAGTGAAGAAGTATTTCGGTATGTAAAGCTCTATCAGCAGGGAAGATAATGACAGTACCTGACTAAGAAGCCCCCGGCTAACTACGTGCCAGCAGCCG

+

::::::::::::::::::::::::::::::::::::::::::::::::::::::::::::::::::::::::::::::::::::::::::::::::::::::::::::::::::::::::::::::::::::::::::::::::::::::::::::::::::::::::::::::::::::::::::::::::::::::::::::::::::::::::::::::::::::::::::::::::::::::::::::::::::::::::::::::::::::::::::::::::::::::::::::::::::::::::::::::::::::::::::::::::::::::::::::::::::::::::::::::::::::::::::::::::::::::::::::::::::::::::::::::::::::::::::::::::::::::::::::::::::::::::::::::::::::::::::::::::

@D0950.29_10506

GATGAACGCTAGCTACAGGCTTAACACATGCAAGTCGAGGGGTAGCATGAAACTTAGCAATAAGTTTTGATGACGACCGGCGCACGGGTGAGTAACACGTATCCAACCTGCCTTTTACTCATGGATAGCCTTCTGAAAAGAAGATTAATACATGATGGTATTCAGAGTTTTCATGGACACTGAATTAAAGATTTATCGGTAAGAGATGGGGATGCGTTCCATTAGATAGTAGGCGGGGTAACGGCCCACCTAGTCAACATGGATAGGGGTTCTGAGAGGAAGGTCCCCCACATTGGAACTGAGGACACGGTCCAAACTCCTACGGGAGGCAGCAGTGAGGAATATTGGTCAATGGACGTAAGTCTGAACCAGCCAAGTAGCGTGAAGGATGAAGGCTCTATGGGTCGTAAACTTCTTTTAT

+

:::::::::::::::::::::::::::::::::::::::::::::::::::::::::::::::::::::::::::::::::::::::::::::::::::::::::::::::::::::::::::::::::::::::::::::::::::::::::::::::::::::::::::::::::::::::::::::::::::::::::::::::::::::::::::::::::::::::::::::::::::::::::::::::::::::::::::::::::::::::::::::::::::::::::::::::::::::::::::::::::::::::::::::::::::::::::::::::::::::::::::::::::::::::::::::::::::::::::::::::::::::::::::::::::::::

@D0950.29_105063

GATGAACGCTAGCGACAGGCTTAACACATGCAAGTCGAGGGGCAGCACAAGGTAGCAATACTGAGGTGGCGACCGGCGCACGGGTGAGTAACGCGTATGCAACCTACCTCTTAGCGGGGGATAACCCGGCGAAAGTCGGACTAATACCGCATAATACTCTTTCTCCGCATGGAGGGAGATTTAAAGATTAATTGCTAAGAGATGGGCATGCGTTCCATTAGGTAGTTGGTAGAGGTAACGGCCTACCAAGCCATCGATGGATAGGGGTTCTGAGAGGAAGGTCCCCCACACTGGTACTGAGACACGGACCAGACTCCTACGGGAGGCAGCAGTGGGGAATATTGCACAATGGGGGAAACCCTGATGCAGCAACGCCGCGTGAGTGAAGAAGTATTTGCGGTATGTAAAGCTCTATCAGCAGGGAAGAAAATGACGGTACCTGACTAAGAAGCACCGGCTAAATACGTGCCAGCAGCCGC

+

:::::::::::::::::::::::::::::::::::::::::::::::::::::::::::::::::::::::::::::::::::::::::::::::::::::::::::::::::::::::::::::::::::::::::::::::::::::::::::::::::::::::::::::::::::::::::::::::::::::::::::::::::::::::::::::::::::::::::::::::::::::::::::::::::::::::::::::::::::::::::::::::::::::::::::::::::::::::::::::::::::::::::::::::::::::::::::::::::::::::::::::::::::::::::::::::::::::::::::::::::::::::::::::::::::::::::::::::::::::::::::::::::::::::::::::::::::::::::::::::

@D0950.29_1051

GATGAACGCTAGCGACAGGCCTAACACATGCAAGTCGAGGGGTAGCACAAGGTAGCAATACTGAGGTGACGACCGGCGCACGGGTGAGTAACGCGTATGCAACCTACCTGTAAGAGTGGGATAGCCTCTCGAAAGAGAGATTAATACCGCATAATACCATTTCACTGCATGGTGAGATGGTTAAAGATTTATTGCTTACAGATGGGCATGCGTAACATTAGCTAGTTGGTGAGGTAACGGCTTACCAAGGCGACGATCAGTAGCCGACTTGAGAGAGTGATCGGCCACATTGGGACTGAGACACGGCCCAAAGCTCCTACGGGAGGCAGCAGTGGGGAAACTATTGGACAATGGGGGAAACCCCGTGATCCAGCGACGCCGCGTGAGTGAAGAAGTATTTCCGGTATGTAAAGCTCTATCAGCAGGGAAGATAATGACAGTACCTGACTAAGAAG

+

:::::::::::::::::::::::::::::::::::::::::::::::::::::::::::::::::::::::::::::::::::::::::::::::::::::::::::::::::::::::::::::::::::::::::::::::::::::::::::::::::::::::::::::::::::::::::::::::::::::::::::::::::::::::::::::::::::::::::::::::::::::::::::::::::::::::::::::::::::::::::::::::::::::::::::::::::::::::::::::::::::::::::::::::::::::::::::::::::::::::::::::::::::::::::::::::::::::::::::::::::::::::::::::::::::::::::::::::::::::::::::::::::::::::

@D0950.29_105113

GATGAACGCTGGCGGCGTGCTTAACACATGCAAGTCGAACGAAGCGCTGGAGGAGCTTGCTCCAAAGGTGACTGAGTGGCGGACGGGTGAGTAACGCGTGGGTAACCTGCCTTACACTGGGGGATAACAGTTGGAAACGACTGCTAATACCGCATAAGCGCACAGTATTGCATGATACAGTGTGAAAAACTCCGGTGGTGTAAGATGGACCCGCGTCTGATTAGCTAGTTGGTGAGGTAATGGCTCACCAAGGCAACGATCAGTAGCCGGCTTGAGAGAGTGAACGGCCACATTGGGACTGAGACACGGCCCAAACTCCTACGGGAGGCAGCAGTGGGGAATATTGCACAATGGGGGAAACCCTGATGCAGCAACGCCGCGTGAGTGAAGAAGTATTTCGGTATGTAAAGCTCTATCAGCAGGGAAGATAATGACAGTACCTGACTAAGAAGCCCCGGCTAACTACGTGCCAGCAGCCGCGGTAA

+

:::::::::::::::::::::::::::::::::::::::::::::::::::::::::::::::::::::::::::::::::::::::::::::::::::::::::::::::::::::::::::::::::::::::::::::::::::::::::::::::::::::::::::::::::::::::::::::::::::::::::::::::::::::::::::::::::::::::::::::::::::::::::::::::::::::::::::::::::::::::::::::::::::::::::::::::::::::::::::::::::::::::::::::::::::::::::::::::::::::::::::::::::::::::::::::::::::::::::::::::::::::::::::::::::::::::::::::::::::::::::::::::::::::::::::::::::::::::::::::::::::::

@D0950.29_105213

GACGAACGCTGGCGGCGTGCCTAACACATGCAAGTCGAGCGATGAAGTTCTTCGGAACGGATTAGCGGCGGACGGGTGAGTAACACGTGGGTAACCTGCCTTGTAGAGGGGGATAGCCTTCCGAAAGGAAGATTAATACCGCATAACATCTTTTTATCGCATGGTAGAAAGATCAAAGGAGCAATCCGCTACAAGATGGACCCGCGGCGCATTAGCTAGTTGGTGAGGTAACGGCTCACCAAGGCGACGATGCGTAGCCGACCTGAGAGGGTGATCGGCCACATTGGAACTGAGACACGGTCCAGACTCCTACGGGAGGCAGCAGTGGGGAATATTGCGCAATGGGGGAAACCCTGACGCAGCAACGCCGCGTGAATGAAGAAGGCCTTAGGGTTGTAAAGTTCTGTTTACGGGGACGATAATGACGGTACCCGTGGAGGAAGCCACGGCTAACTACGTGCCAGCAGCCGCGGTAA

+

::::::::::::::::::::::::::::::::::::::::::::::::::::::::::::::::::::::::::::::::::::::::::::::::::::::::::::::::::::::::::::::::::::::::::::::::::::::::::::::::::::::::::::::::::::::::::::::::::::::::::::::::::::::::::::::::::::::::::::::::::::::::::::::::::::::::::::::::::::::::::::::::::::::::::::::::::::::::::::::::::::::::::::::::::::::::::::::::::::::::::::::::::::::::::::::::::::::::::::::::::::::::::::::::::::::::::::::::::::::::::::::::::::::::::::::::::::::::::::

@D0950.29_105322

GACGAACGCTGGCGGCGTGCTTAACACATGCAAGTCGAGCGATGAAGCTTCTTCGGAACGGATTAGCGGCGGACGGGTGAGTAACACGTGGGTAACCTGCCTTGTAGAGGGGGATAGCCTTCCGAAAGGAAGATTAATACCGCATAACATCTTTTTATCGCATGGTAGAAAGATCAAAGGAGTAATCCGCTACAAGATGGACCCGCGGCGCATTAGCTAGTTGGTGAGGTAACGGCTCACCAAGGCGACGATGCGTAGCCGACCTGAGAGGGTGATCGGCCACATTGGAACTGAGACACGGTCCAGACTCCTACGGGAGGCAGCAGTGGGGAATATTGCGCAATGGGGGAAACCCTGACGCAGCAACGCCGCGTGAATGAAGAAGGCCTTAGGGTTGTAAAGTTCTGTTTACGGGGACGATAATGACGGTACCCGTGGAGGAAGCCACGGCTAACTACGTGCCAGCAGCCGCGGTAA

+

:::::::::::::::::::::::::::::::::::::::::::::::::::::::::::::::::::::::::::::::::::::::::::::::::::::::::::::::::::::::::::::::::::::::::::::::::::::::::::::::::::::::::::::::::::::::::::::::::::::::::::::::::::::::::::::::::::::::::::::::::::::::::::::::::::::::::::::::::::::::::::::::::::::::::::::::::::::::::::::::::::::::::::::::::::::::::::::::::::::::::::::::::::::::::::::::::::::::::::::::::::::::::::::::::::::::::::::::::::::::::::::::::::::::::::::::::::::::::::::

@D0950.29_105335

GATGAACGCTAGCGACAGGCCTAACACATGCAAGTCGAGGGGTAGCACAAGGAAGCTTGCTTCTGAGGTGACGACCGGCGCACGGGTGAGTAACGCGTATGCAACCTACCTGTAAGAGTGGGATAGCCTCTCGAAAGAGAGATTAATACCGCATAATACCATTTCACTGCATGGTGAGATGGTTAAAGATTTATTGCTTACAGATGGGCATGCGTAACATTAGCTAGTTGGTGAGGTAACGGCTCACCAAGGCAACGATGTTTAGGGGTTCTGAGAGGAAGGTCCCCCACACTGGTACTGAGACACGGACCAGACTCCTACGGGAGGCAGCAGTGAGGAATATTGGTCAATGGACGAGAGTCTGAACCAGCCAAGTCGCGTGAAGGATGAAGGTCTTATGGATTGTAAACTTCTTTTATACGGGAATAAAAAATGCCACGTGTGGCATATTGCATGTACCGTATGAATAAGGATCGGCTAAACTCCGTGCCAGCAGCCGCGGTAA

+

:::::::::::::::::::::::::::::::::::::::::::::::::::::::::::::::::::::::::::::::::::::::::::::::::::::::::::::::::::::::::::::::::::::::::::::::::::::::::::::::::::::::::::::::::::::::::::::::::::::::::::::::::::::::::::::::::::::::::::::::::::::::::::::::::::::::::::::::::::::::::::::::::::::::::::::::::::::::::::::::::::::::::::::::::::::::::::::::::::::::::::::::::::::::::::::::::::::::::::::::::::::::::::::::::::::::::::::::::::::::::::::::::::::::::::::::::::::::::::::::::::::::::::::::::::::::::

@D0950.29_1054

GATGAACGCTAGCGACAGGCCTAACACATGCAAGTCGAGGGGTAGCACAAGGTAGTAATACTGAGGTGACGACCGGCGCACGGGTGAGTAACGCGTATGCAACCTACCTGTAAGAGTGGGATAGCCTCTCGAAAGAGAGATTAATACCGCATAATACCATTTCACTGCATGGTGAGATGGTTAAAGATTTATTGCTTACAGATGGGCATGCGTAACATTAGCTAGTTGGTGAGGTAACGGCTCACCAAGGCAACGATGTTTAGGGGGTTCTGAGAGGAAGGTCCCCCGACACTGGTACTGAGACACGGACCAGACTCCTACGGGAGGCAGCAGTGAGGAATATTGGTCAATGGACGAGAGTCTGAACCAGCCAAGTCGCGTGAAGGATGAAGGTCTTATGGATTGTAAA

+

:::::::::::::::::::::::::::::::::::::::::::::::::::::::::::::::::::::::::::::::::::::::::::::::::::::::::::::::::::::::::::::::::::::::::::::::::::::::::::::::::::::::::::::::::::::::::::::::::::::::::::::::::::::::::::::::::::::::::::::::::::::::::::::::::::::::::::::::::::::::::::::::::::::::::::::::::::::::::::::::::::::::::::::::::::::::::::::::::::::::::::::::::::::::::::::::::::::::::::::::::::::::::

@D0950.29_105423

GATGAACGCTAGCTACAGGCTTAACACATGCAAGTCGAGGGGTAGCATGAAACTTAGCAATAAGTTTTGATGACGACCGGCGCACGGGTGAGTAACACGTATCCAACCTGCCTTTTACTCATGGATAGCCTTCTGAAAAGAAGATTAATACATGATGGTATTCAGAGTTTTCATGGACACTGAATTAAAGATTTTATCGGTAAAGAGATGGGGATGCGTTCCATTAGATAGTAGGCGGGGTAACGGCCCACCTAGTCAACGATGGATAGGGGTTCTGAGAGGAAGGTCCCCCACATTGGAACTGAGACACGGTCCAAACTCCTACGGGAGGCAGCAGTGAGGAATATTGGTCAATGGACGTAAGTCTGAACCAGCCAAGTAGCGTGAAGGATGAAGGCTCTATGGGTCGTAAACTTCTTTTTATAAAAGGAATAAAGTATGCCACGTGTGGTGTTTTTGTATGTACTTTATGAATAAGGATCGGCTAACTCCGTGCCAGCAGCCGC

+

::::::::::::::::::::::::::::::::::::::::::::::::::::::::::::::::::::::::::::::::::::::::::::::::::::::::::::::::::::::::::::::::::::::::::::::::::::::::::::::::::::::::::::::::::::::::::::::::::::::::::::::::::::::::::::::::::::::::::::::::::::::::::::::::::::::::::::::::::::::::::::::::::::::::::::::::::::::::::::::::::::::::::::::::::::::::::::::::::::::::::::::::::::::::::::::::::::::::::::::::::::::::::::::::::::::::::::::::::::::::::::::::::::::::::::::::::::::::::::::::::::::::::::::::::::::::::

@D0950.29_105432

GATGAACGCTAGCGACAGGCCTAACACATGCAAGTCGAGGGGTAGCACAAGGTAGCAATACTGAGGTGACGACCGGCGCACGGGTGAGTAACGCGTATGCAACCTACCTGTAAGAGTGGGATAGCCTCTCGAAAGAGAGATTAATACCGCATAATACCATTTCACTGCATGGTGAGATGGTTAAAGATTTATTGCTTACAGATGGGCATGCGTAACATTAGCTAGTTGGTGAGGTAACGGCTCACCAAGGCAACGATGTTTAGGGGTTCTGAGAGGAAGGTCCCCCACACTGGTACTGAGACACGGACCAGACTCCTACGGGAGGCAGCAGTGAGGAATATTGGTCAATGGACGAGAGTCTGAACCAGCCAAGTCGCGTGAAGGATGAAGGTCTTATGGATTGTAAACTTCTTTTATACGGGAATAAAAAATGCCACGTGTGGCATATTGCATGTACCGTATGAATAAGGATCGGCTAACTCCGTGCCAGCAGCCGCGGTAA

+

::::::::::::::::::::::::::::::::::::::::::::::::::::::::::::::::::::::::::::::::::::::::::::::::::::::::::::::::::::::::::::::::::::::::::::::::::::::::::::::::::::::::::::::::::::::::::::::::::::::::::::::::::::::::::::::::::::::::::::::::::::::::::::::::::::::::::::::::::::::::::::::::::::::::::::::::::::::::::::::::::::::::::::::::::::::::::::::::::::::::::::::::::::::::::::::::::::::::::::::::::::::::::::::::::::::::::::::::::::::::::::::::::::::::::::::::::::::::::::::::::::::::::::::::::::::

@D0950.29_105444

ATTGAACGCTGGCGGCATGCCTTACACATGCAAGTCGAACGGTAACAGGTCTTCGGATGCTGACGAGTGGCGAACGGGTGAGTAATACATCGGAACGTGCCCGAGAGTGGGGGATAACGAGGCGAAAGCTTTGCTAATACCGCATACGATCTCAGGATGAAAGCAGGGGACCGCAAGGCCTTGCGCTCACGGAGCGGCCGATGGCAGATTAGGTAGTTGGTGGGATAAAGCTTACCAAGCCGACGATCTGTAGCTGGTCTGAGAGGACGACCAGCCACACTGGGACTGAGACACGGCCCAGACTCCTACGGGAGGCAGCAGTGGGGAATATTGCGCAATGGGGGAAACCCTGACGCAGCAACGCCGCGTGAATGAAGAAGGCCTTAGGGTTGTAAAGTTCTGTTTACGGGGACGATAATGACGGTACCCGTGGAGGAAGCCACGGCTAACTACGTGCCAGCAGCCGCGGT

+

::::::::::::::::::::::::::::::::::::::::::::::::::::::::::::::::::::::::::::::::::::::::::::::::::::::::::::::::::::::::::::::::::::::::::::::::::::::::::::::::::::::::::::::::::::::::::::::::::::::::::::::::::::::::::::::::::::::::::::::::::::::::::::::::::::::::::::::::::::::::::::::::::::::::::::::::::::::::::::::::::::::::::::::::::::::::::::::::::::::::::::::::::::::::::::::::::::::::::::::::::::::::::::::::::::::::::::::::::::::::::::::::::::::::::::::::::::::

@D0950.29_105450

GATGAACGCTAGCGACAGGCCTAACACATGCAAGTCGAGGGGTAGCACAAGGTAGCAATACTGAGGTGACGACCGGCGCACGGGTGAGTAACGCGTATGCAACCTACCTGTAAGAGTGGGATAGCCTCTCGAAAGAGAGATTAATACCGCATAATACCATTTCACTGCATGGTGAGATGGTTAAAGATTTATTGCTTACAGATGGGCATGCGTAACATTAGCTTGTTGGTGAGGTAACGGCTCACCAAGGCAACGATGTTTAGGGGTTCTGAGAGGAAGGTCCCCCACACTGGTACTGAGACACGGACCAGACTCCTACGGGAGGCAGCAGTGAGGAATATTGGTCAATGGACGAGAGTCTGAACCAGCCAAGTCGCGTGAAGGATGAAGGTCTTATGGATTGTAAACTTCTTTTATACGGGAATAAAAATGCCACGTGTGGCATATTGCATGTACCGTATGAATAAGGATCGGCTAACTCCGTGCCAGCAGCCGCGGTAA

+

:::::::::::::::::::::::::::::::::::::::::::::::::::::::::::::::::::::::::::::::::::::::::::::::::::::::::::::::::::::::::::::::::::::::::::::::::::::::::::::::::::::::::::::::::::::::::::::::::::::::::::::::::::::::::::::::::::::::::::::::::::::::::::::::::::::::::::::::::::::::::::::::::::::::::::::::::::::::::::::::::::::::::::::::::::::::::::::::::::::::::::::::::::::::::::::::::::::::::::::::::::::::::::::::::::::::::::::::::::::::::::::::::::::::::::::::::::::::::::::::::::::::::::::::::::::

@D0950.29_105498

GATGAACGCTAGCTACAGGCTTAACACATGCAAGTCGAGGGGTAGCATGAAACTTAGCAATAAGTTTTGATGACGACCGGCGCACGGGTGAGTAACACGTATCCAACCTGCCTTTTACTCATGGATAGCCTTCTGAAAAGAAGATTAATACATGATGGTATTCAGAGTTTTCATGGACACTGAATTAAAGATTTTATCGGTAAGAGATGGGGATGCGTTCCATTAGATAGTAGGCGGGGTAACGGCCCACCTAGTCAACGATGGATAGGGGTTCTGAGAGGAAGGTCCCCCACATTGGAACTGAGACACGGTCCAAACTCCTACGGGAGGCAGCAGTGAGGAATATTGGTCAATGGACGTAAGTCTGAACCAGCCAAGTAGCGTGAAGGATGAAGGCTCTATGGGTCGTAAACTTCTTTTATAAAAGGAATAAAGTATGCCACGTGTGGTGTTTTTGTATGTACTTTATGAATAAGGATCGGCTAACTCCGTGCCAGCAGCCGC

+

::::::::::::::::::::::::::::::::::::::::::::::::::::::::::::::::::::::::::::::::::::::::::::::::::::::::::::::::::::::::::::::::::::::::::::::::::::::::::::::::::::::::::::::::::::::::::::::::::::::::::::::::::::::::::::::::::::::::::::::::::::::::::::::::::::::::::::::::::::::::::::::::::::::::::::::::::::::::::::::::::::::::::::::::::::::::::::::::::::::::::::::::::::::::::::::::::::::::::::::::::::::::::::::::::::::::::::::::::::::::::::::::::::::::::::::::::::::::::::::::::::::::::::::::::::::::

@D0950.29_105510

ATTGAACGCTGGCGGCATGCCTTACACATGCAAGTCGAACGGTAACAGGTCTTCGGATGCTGACGAGTGGCGAACGGGTGAGTAATACATCGGAACGTGCCCGATCGTGGGGATAACGAAGCGAAAGCTTTGCTAATACCGCATACGATCTACGGATGAAAGCAGGGGACCGCAAGGCCTTGCGCGAACGGAGCGGCCGATGGCAGATTAGGTAGTTGGTGGGATAAAAGCTTACCAAGCCGACGATCTGTAGCTGGTCTGAGAGGACGACCAGCCACACTGGGACTGAGACACGGCCCAGACTCCTACGGGAGGCAGCAGTGGGGAATTTTGGACAATGGGCGAAAGCCTGATCCAGCCATGCCGCGTGCAGGATGAAGGCCTTCGGGTTGTAAACTGCTTTTGTACGGAACGAAAAGACTCTGGTTAATACCTGGGGTCCATGACGGTACCGTAAGAATAAGCACCGGCTAACTACGTGCCAGTAGCCGCGGTAA

+

:::::::::::::::::::::::::::::::::::::::::::::::::::::::::::::::::::::::::::::::::::::::::::::::::::::::::::::::::::::::::::::::::::::::::::::::::::::::::::::::::::::::::::::::::::::::::::::::::::::::::::::::::::::::::::::::::::::::::::::::::::::::::::::::::::::::::::::::::::::::::::::::::::::::::::::::::::::::::::::::::::::::::::::::::::::::::::::::::::::::::::::::::::::::::::::::::::::::::::::::::::::::::::::::::::::::::::::::::::::::::::::::::::::::::::::::::::::::::::::::::::::::::::::::::

@D0950.29_105513

GACGAACGCTGGCGGCGTGCCTAACACATGCAAGTCGAGCGATGAAGTTCTTCGGAACGGATTAGCGGCGGACGGGTGAGTAACACGTGGGTAACCTGCCTTGTAGAGGGGGATAGCCTTCCGAAAGGAAGATTAATACCGCATAACATCTTTTTATCGCATGGTAGAAAGATCAAAGGAGCAATCCGCTACAAGATGGACCCGCGGCGCATTAGCTAGTTGGTGAGGTAACGGCTCACCAAGGCGACGATGCGTAGCCGACCTGAGAGGGTGATCGGCCACATTGGAACTGAGACACGGTCCAGACTCCTACGGGAGGCAGCAGTGGGGAATATTGCGCAATGGGGGAAACCCTGACGCAGCAACGCCGCGTGAATGAAGAAGGCCTTAGGGTTGTAAAGTTCTGTTTACGGGGACGATAATGACGGTACCCGTGGAGGAAGCCACGGCTAACTACGTGCCAGCAGCCGCGGTAA

+

::::::::::::::::::::::::::::::::::::::::::::::::::::::::::::::::::::::::::::::::::::::::::::::::::::::::::::::::::::::::::::::::::::::::::::::::::::::::::::::::::::::::::::::::::::::::::::::::::::::::::::::::::::::::::::::::::::::::::::::::::::::::::::::::::::::::::::::::::::::::::::::::::::::::::::::::::::::::::::::::::::::::::::::::::::::::::::::::::::::::::::::::::::::::::::::::::::::::::::::::::::::::::::::::::::::::::::::::::::::::::::::::::::::::::::::::::::::::::::

@D0950.29_105523

GATGAACGCTAGCGACAGGCCTAACACATGCAAGTCGAGGGGTAGCACAAGGTAGCAATACTGAGGTGACGACCGGCGCACGGGTGAGTAACGCGTATGCAACCTACCTGTAAGAGTGGGATAGCCTCTCGAAAGAGAGATTAATACCGCATAATACCATTTCACTGCATGGTGAGATGGTTAAAGATTTATTGCTTACAGATGGGCATGCGTAACATTAGCTAGTTGGTGAGGTAACGGCTCACCAAGGCAACGATGTTTAGGGGTTCTGAGAGGAAGGTCCCCCACACTGGTACTGAGACACGGACCAGACTCCTACGGGAGGCAGCAGTGAGGAATATTGGTCAATGGACGAGAGTCTGAACCAGCCAAGTCGCGTGAAGGATGAAGGTCTTATGGATTGTAAACTTCTTTTATACGGGAATAAAAATGCCACGTGTGGCATATTGCATGTACCGTATGAATAAGGATCGGCTAACTCCGTGCCAGCAGCCGCGGTAA

+

:::::::::::::::::::::::::::::::::::::::::::::::::::::::::::::::::::::::::::::::::::::::::::::::::::::::::::::::::::::::::::::::::::::::::::::::::::::::::::::::::::::::::::::::::::::::::::::::::::::::::::::::::::::::::::::::::::::::::::::::::::::::::::::::::::::::::::::::::::::::::::::::::::::::::::::::::::::::::::::::::::::::::::::::::::::::::::::::::::::::::::::::::::::::::::::::::::::::::::::::::::::::::::::::::::::::::::::::::::::::::::::::::::::::::::::::::::::::::::::::::::::::::::::::::::::

@D0950.29_10564

GATGAACGCTAGCGACAGGCCTAACACATGCAAGTCGAGGGGTAGCACAAGGAAGCTTGCTTCTGAGGTGACGACCGGCGCACGGGTGAGTAACGCGTATGCAACCTACCTATAAGAAGGGGATAGCCTCTCGAAAGAGAGATTAATACCGTATAACACTATGAAGCCGCATGTTTTATAGTTAAAGATTTATTGCTTATAGATGGGCATGCGTAACATTAGCTAGTTGGTAAGGTAACGGCTTACCAAGGCAACGATGTTTAGGGGTTCTGAGAGGAAGGTCCCCCACACTGGTACTGAGACACGGACCAGACTCCTACGGGAGGCAGCAGTGAGGAATATTGGTCAATGGACGAGAGTCTGAACCAGCCAAGTCGCGTGAAGGATGAAGGTCTTATGGATTGTAAACTTCTTTTATACGGGGAAATAAAAAACTACCACGTGTGGTATATTGCAATGTACCGTATGAATAAGGATCGGCTAACTCCGTGCCAGCAGCCGC

+

::::::::::::::::::::::::::::::::::::::::::::::::::::::::::::::::::::::::::::::::::::::::::::::::::::::::::::::::::::::::::::::::::::::::::::::::::::::::::::::::::::::::::::::::::::::::::::::::::::::::::::::::::::::::::::::::::::::::::::::::::::::::::::::::::::::::::::::::::::::::::::::::::::::::::::::::::::::::::::::::::::::::::::::::::::::::::::::::::::::::::::::::::::::::::::::::::::::::::::::::::::::::::::::::::::::::::::::::::::::::::::::::::::::::::::::::::::::::::::::::::::::::::::::::::::::

@D0950.29_10568

GATGAACGCTAGCGACAGGCCTAACACATGCAAGTCGAGGGGCAGCGAGAGAGTAGCAATACTTTTGTCGGCGACCGGCGCACGGGTGAGTAACACGTATGCAACCTGCCCATAACAGGGGATAATCGGAAGAAATTCCGTCTAATACCGCGTAACCCTGCATTATCTCATGATAACGCAGGTAAAGAAGCAATTCGGTTATGGATGGGCATGCGGAACATTAGGTAGTTGGTGAGGTAACGGCTCACCAAGCCGACGATGTATAGGGGTTCTGAGAGGAAGGTCCCCCACACTGGTACTGAGACACGGACCAGACTCCCTACGGGAGGCAGCAGTGAGGAATATTGGTCAATGGGCGCGAGCCTGAACCAGCCAAGTCGCGTGAAGGATGAAGGTTCTATGGATTGTAAACTTCTTTTGTCAGGGAACAAAGAGCTCACGTGTGAGCAGATGAGTGTACCTGAAGAAAAGCATC

+

:::::::::::::::::::::::::::::::::::::::::::::::::::::::::::::::::::::::::::::::::::::::::::::::::::::::::::::::::::::::::::::::::::::::::::::::::::::::::::::::::::::::::::::::::::::::::::::::::::::::::::::::::::::::::::::::::::::::::::::::::::::::::::::::::::::::::::::::::::::::::::::::::::::::::::::::::::::::::::::::::::::::::::::::::::::::::::::::::::::::::::::::::::::::::::::::::::::::::::::::::::::::::::::::::::::::::::::::::::::::::::::::::::::::::::::::::::::::::::

@D0950.29_105745

GATGAACGCTAGCGACAGGCCTAACACATGCAAGTCGAGGGGTAGCACAAGGTAGCAATACTGAGGTGACGACCGGCGCACGGGTGAGTAACGCGTATGCAACCTACCTGTAAGAGTGGGATAGCCTCTCGAAAGAGAGATTAATACCGCATAATACCATTTCACTGCATGGTGAGATGGTTAAAGATTTATTGCTTACAGATGGGCATGCGTAACATTAGCTAGTTGGTGAGGTAACGGCTCACCAAGGCAACGATGTTTAGGGGTTCTGAGAGGAAGGTCCCCCGACACTGGTACTGAGACACGGACCAGACTCCTACGGGAGGCAGCAGTGAGGAATATTGGTCAATGGACGAGAGTCTGAACCAGCCAAGTCGCGTGAAGGATGAAGGTCTTATGGATTGTAAACTTCTTTTATACGGGAATAAAAATGCCACGTGTGGCATATTGCATGTACCGTATGAATAAGGATCGGCTAACTCCGTGCCAGCAGCCGCGGTAA

+

::::::::::::::::::::::::::::::::::::::::::::::::::::::::::::::::::::::::::::::::::::::::::::::::::::::::::::::::::::::::::::::::::::::::::::::::::::::::::::::::::::::::::::::::::::::::::::::::::::::::::::::::::::::::::::::::::::::::::::::::::::::::::::::::::::::::::::::::::::::::::::::::::::::::::::::::::::::::::::::::::::::::::::::::::::::::::::::::::::::::::::::::::::::::::::::::::::::::::::::::::::::::::::::::::::::::::::::::::::::::::::::::::::::::::::::::::::::::::::::::::::::::::::::::::::::

@D0950.29_105747

GATGAACGCTAGCGACAGGCCTAACACATGCAAGTCGAGGGGTAGCACAAGGTAGTAATACTGAGGTGACGACCGGCGCACGGGTGAGTAACGCGTATGCAACCTACCTGTAAGAGTGGGATAGCCTCTCGAAAGAGAGATTAATACCGCATAATACCATTTCACTGCATGGTGAGATGGTTAAAGATTTATTGCTTACAGATGGGCATGCGTAACATTAGCTAGTTGGTGAGGTAACGGCTCACCAAGGCAACGATGTTTAGGGGTTCTGAGAGGAAGGTCCCCCACACTGGTACTGAGACACGGACCAGACTCCTACGGGAGGCAGCAGTGAGGAATATTGGTCAATGGACGAGAGTCTGAACCAGCCAAGTCGCGTGAAGGATGAAGGTCTTATGGATTGTAAACTTCTTTTATACGGGAATAAAAATGCCACGTGTGGCATATTGCATGTACCGTATGAATAAGGATCGGCTAACTCCGTGCCAGCAGCCGCGGTAA

+

:::::::::::::::::::::::::::::::::::::::::::::::::::::::::::::::::::::::::::::::::::::::::::::::::::::::::::::::::::::::::::::::::::::::::::::::::::::::::::::::::::::::::::::::::::::::::::::::::::::::::::::::::::::::::::::::::::::::::::::::::::::::::::::::::::::::::::::::::::::::::::::::::::::::::::::::::::::::::::::::::::::::::::::::::::::::::::::::::::::::::::::::::::::::::::::::::::::::::::::::::::::::::::::::::::::::::::::::::::::::::::::::::::::::::::::::::::::::::::::::::::::::::::::::::::::

@D0950.29_105814

GATGAACGCTGGCGGCGTGCTTAACACATGCAAGTCGAACGAAGCACTTTGAAGAGCTTGCTCTTTGAAGTGACTGAGTGGCGGACGGGTGAGTAACGCGTGGGTAACCTGCCTCATACAGGGGGATAACAGTTAGAAATGACTGCTAACACCGCATAACCCGCTAGCATCGCATGATGCAGACGGAAAATATTTATAGGTATGAGATGGGCCCGCGTCTGATTAGCTAGTTGGTGGGGTAACGCCTACCAAGGCAACGATCAGGTAGCCGACTTGAGAGAGTGATCGGCCACATTGGGACTGAGACACGGCCCAAACTCCTACGGGAGGCAGCAGTGGGGAATATTGGACAATGGGGGAAACCCTGATCCAGCGACGCCGCGTGAGTGAAGAAGTATTTCGGTATGTAAAGCTCTATCAGCAGGGAAGATAATGACAGTACCTGACTAAGAAGCCCCCGGCTAACTACGTGCCAGCAGCCGC

+

:::::::::::::::::::::::::::::::::::::::::::::::::::::::::::::::::::::::::::::::::::::::::::::::::::::::::::::::::::::::::::::::::::::::::::::::::::::::::::::::::::::::::::::::::::::::::::::::::::::::::::::::::::::::::::::::::::::::::::::::::::::::::::::::::::::::::::::::::::::::::::::::::::::::::::::::::::::::::::::::::::::::::::::::::::::::::::::::::::::::::::::::::::::::::::::::::::::::::::::::::::::::::::::::::::::::::::::::::::::::::::::::::::::::::::::::::::::::::::::::::::

@D0950.29_105873

GATGAACGCTAGCTACAGGCTTAACACATGCAAGTCGAGGGGTAGCATGAAACTTAGCAATAAGTTTTGATGACGACCGGCGCACGGGTGAGTAACACGTATCCAACCTGCCTTTTACTCATGGATAGCCTTCTGAAAAGAAGATTAATACATGATGGTATTCAGAGTTTTCATGGACACTGAATTAAAGATTTATCGGTAAGAGATGGGGATGCGTTCCATTAGATAGTAGGCGGGGTAACGGCCCACCTAGTCAACGATGGATAGGGGTTCTGAGAGGAAGGTCCCCACATTGGAACTGAGACACGGTCCAAACTCCTACGGGAGGCAGCAGTGAGGAATATTGGTCAATGGACGTAAGTCTGAACCAGCCAAGTAGCGTGAAGGATGAAGGCTCTATGGGTCGTAAACTTC

+

::::::::::::::::::::::::::::::::::::::::::::::::::::::::::::::::::::::::::::::::::::::::::::::::::::::::::::::::::::::::::::::::::::::::::::::::::::::::::::::::::::::::::::::::::::::::::::::::::::::::::::::::::::::::::::::::::::::::::::::::::::::::::::::::::::::::::::::::::::::::::::::::::::::::::::::::::::::::::::::::::::::::::::::::::::::::::::::::::::::::::::::::::::::::::::::::::::::::::::::::::::::::::::::

@D0950.29_105889

GATGAACGCTAGCGACAGGCCTAACACATGCAAGTCGAGGGGTAGCACAAGGTAGCAATACTGAGGTGACGACCGGCGCACGGGTGAGTAACGCGTATGCAACCTACCTGTAAGAGTGGGATAGCCTCTCGAAAGAGAGATTAATACCGCATAATACCATTTCACTGCATGGTGAGATGGTTAAAGATTTATTGCTTACAGATGGGCATGCGTAACATTAGCTAGTTGGTGAGGTAACGGCTCACCAAGGCAACGATGTTTAGGGGTTCTGAGAGGAAGGTCCCCCACACTGGTACTGAGACACGGACCAGACTCCTACGGGAGGCAGCAGTGAGGAATATTGGTCAATGGACGAGAGTCTGAACCAGCCAAGTCGCGTGAAGGATGAAGGTCTTATGGATTGTAAACTTCTTTTATACGGGAATAAAAATGCCACGTGTGGCATATTGCATGTACCGTATGAATAAGGATCGGCTAAATCCGCTGCCAGCAGCCGCGGTAA

+

::::::::::::::::::::::::::::::::::::::::::::::::::::::::::::::::::::::::::::::::::::::::::::::::::::::::::::::::::::::::::::::::::::::::::::::::::::::::::::::::::::::::::::::::::::::::::::::::::::::::::::::::::::::::::::::::::::::::::::::::::::::::::::::::::::::::::::::::::::::::::::::::::::::::::::::::::::::::::::::::::::::::::::::::::::::::::::::::::::::::::::::::::::::::::::::::::::::::::::::::::::::::::::::::::::::::::::::::::::::::::::::::::::::::::::::::::::::::::::::::::::::::::::::::::::::

@D0950.29_105910

GATGAACGCTAGCGACAGGCCTAACACATGCAAGTCGAGGGGTAGCACAAGGAAGCTTGCTTCTGAGGTGACGACCGGCGCACGGGTGAGTAACGCGTATGCAACCTGCCTATAAGAAGGGGATAGCCTCTCGAAAGAGAGATTAATACCGTATAACACTATGAAGCCGCATGGTTTTATAGTTAAAGATTTATTGCTTATAGATGGGCATGCGTAACATTAGCTAGTTGGTGAGGTAACGGCTCACCAAGGCAACGATGTTTAGGGGTTCTGAGAGGAAGGTCCCCCACACTGGTACTGAGACACGGACCAGACTCCTACGGGAGGCAGCAGTGAGGAATATTGGTCAATGGACGAGAGTCTGAACCAGCCAAGTCGCGTGAAGGATGAAGGTCTTATGGATTGTAAACTTCTTTTATACGGGAATAAAAAACTGCCACGTGTGGCATATTGCATGTACCGTATGAATAAGGATCGGCTAACTCCGTGCCAGCAGCCGC

+

::::::::::::::::::::::::::::::::::::::::::::::::::::::::::::::::::::::::::::::::::::::::::::::::::::::::::::::::::::::::::::::::::::::::::::::::::::::::::::::::::::::::::::::::::::::::::::::::::::::::::::::::::::::::::::::::::::::::::::::::::::::::::::::::::::::::::::::::::::::::::::::::::::::::::::::::::::::::::::::::::::::::::::::::::::::::::::::::::::::::::::::::::::::::::::::::::::::::::::::::::::::::::::::::::::::::::::::::::::::::::::::::::::::::::::::::::::::::::::::::::::::::::::::::::::

@D0950.29_105967

ATTGAACGCTGGCGGCATGCCTTACACATGCAAGTCGAACGGTAACAGGTCTTCGGATGCTGACGAGTGGCGAACGGGTGAGTAATACATCGGAACGTGCCCGATCGTGGGGGATAACGAAGCGAAAGCTTTGCTAATACCGCATACGATCTACGGATGAAAGCAGGGGACCGCAAGGCCTTGCGCGAACGGAGCGGCCGATGGCAGATTAGGTAGTTGGTGGGATAAAAGCTTACCAAGCCGACGATCTGTAGCTGGTCTGAGAGGACGACCAGCCACACTGGGACTGAGACACGGCCCAGACTCCTACGGGAGGCAGCAGTGGGGAATTTTGGACAATGGGCGAAAGCCTGATCCAGCCATGCCGCGTGCAGGATGAAGGCCTTCGGGTTGTAAACTGCTTTTGTACGGAACGAAAAGACTCTGGTTAATACCTGGGGGTCCATGACGGTACCGTAAGAATAAGCACCGGCTAACTACGTGCCAGCAGCCGCGGTAA

+

:::::::::::::::::::::::::::::::::::::::::::::::::::::::::::::::::::::::::::::::::::::::::::::::::::::::::::::::::::::::::::::::::::::::::::::::::::::::::::::::::::::::::::::::::::::::::::::::::::::::::::::::::::::::::::::::::::::::::::::::::::::::::::::::::::::::::::::::::::::::::::::::::::::::::::::::::::::::::::::::::::::::::::::::::::::::::::::::::::::::::::::::::::::::::::::::::::::::::::::::::::::::::::::::::::::::::::::::::::::::::::::::::::::::::::::::::::::::::::::::::::::::::::::::::::

@D0950.29_105998

GATGAACGCTGGCGGCGTGCTTAACACATGCAAGTCGAGCGAAGCACTTTACTTAGAGATCTTCGGATGGAAGAGTTTTGTGACTGAGCGGCGGACGGGTGAGTAACGCGTGGGTAACCTGCCTCATACAGGGGGATAACAGTTAGAAATGACTGCTAATACCGCATAAGACCACAGTATCGCATGATACGGTGGGAAAAACTCCGGTGGTATGAGATGGACCCGCGTCTGATTAGCTAGTTGGTAAGGTAACGGCTTACCAAGGCGACGATCAGTAGCCGACCTGAGAGGGGTGACCGGCCACATTGGGACTGAGACACGGCCCAAACTCCTACGGGAGGCAGCAGTGGGGGAATATTGGACAATGGGGGAAACCCTGATCCAGCGACGCCGCGTGAGTGAAGAAGTATTTCGGTATGTAAAGCTCTATCAGCAGGGAAGTAATGACAGTACCTGACTAAGAAGCCCCCGGTAACTAGTGCCAGCACGC

+

::::::::::::::::::::::::::::::::::::::::::::::::::::::::::::::::::::::::::::::::::::::::::::::::::::::::::::::::::::::::::::::::::::::::::::::::::::::::::::::::::::::::::::::::::::::::::::::::::::::::::::::::::::::::::::::::::::::::::::::::::::::::::::::::::::::::::::::::::::::::::::::::::::::::::::::::::::::::::::::::::::::::::::::::::::::::::::::::::::::::::::::::::::::::::::::::::::::::::::::::::::::::::::::::::::::::::::::::::::::::::::::::::::::::::::::::::::::::::::::::::::::::::

@D0950.29_106056

GATGAACGCTAGCGACAGGCCTAACACATGCAAGTCGAGGGGTAGCACAAGGTAGTAATACTGAGGTGACGACCGGCGCACGGGTGAGTAACGCGTATGCAACCTACCTGTAAGAGTGGGATAGCCTCTCGAAAGAGAGATTAATACCGCATAATACCATTTCACTGCATGGTGAGATGGTTAAAGATTTATTGCTTACAGATGGGCATGCGTAACATTAGCTAGTTGGTGAGGTAACGGCTCACCAAGGCAACGATGTTTAGGGGTTCTGAGAGGAAGGTCCCCCGACACTGGTACTGAGACACGGACCAGACTCCTACGGGAGGCAGCAGTGAGGAATATTGGTCAATGGACGAGAGTCTGAACCAGCCAAGTCGCGTGAAGGATGAAGGTCTTATGGATTGTAAACTTCTTTTATAAAGGAATAAAGTATGCCACGTGTGGTGTTTTTGTAATGTACTTTATGAATAAGGATCGGCTAACTCCGTGCCAGCAGCCGCGGTAA

+

:::::::::::::::::::::::::::::::::::::::::::::::::::::::::::::::::::::::::::::::::::::::::::::::::::::::::::::::::::::::::::::::::::::::::::::::::::::::::::::::::::::::::::::::::::::::::::::::::::::::::::::::::::::::::::::::::::::::::::::::::::::::::::::::::::::::::::::::::::::::::::::::::::::::::::::::::::::::::::::::::::::::::::::::::::::::::::::::::::::::::::::::::::::::::::::::::::::::::::::::::::::::::::::::::::::::::::::::::::::::::::::::::::::::::::::::::::::::::::::::::::::::::::::::::::::::::

@D0950.29_106118

GATGAACGCTAGCTACAGGCTTAACACATGCAAGTCGAGGGGTAGCATGAAACTTAGCAATAAGTTTTGATGACGACCGGCGCACGGGTGAGTAACACGTATCCAACCTGCCTTTTACTCATGGATAGCCTTCTGAAAAGAAGATTAATACATGATGGTATTCAGAGTTTTCATGGACACTGAATTAAAGATTTTATCGGTAAGAGATGGGGATGCGTTCCATTAGATAGTAGGCGGGGTAACGGCCCACCTAGTCAACGATGGATAGGGGTTCTGAGAGGAAGGTCCCCCACATTGGAACTGAGACACGGTCCAAACTCCTACGGGAGGCAGCAGTGAGGAATATTGGTCAATGGACGTAAGTCTGAACCAGCCAAGTAGCGTGAAGGATGAAGGCTCTATGGGTCGTAAACTTCTTTTATACGGGGAATAAAAAATGCCACGTGTGGCATTATTGCATGTACCGTATGAATAAGGATCGGCTAACTCCGTGCCAGCAGCCGC

+

::::::::::::::::::::::::::::::::::::::::::::::::::::::::::::::::::::::::::::::::::::::::::::::::::::::::::::::::::::::::::::::::::::::::::::::::::::::::::::::::::::::::::::::::::::::::::::::::::::::::::::::::::::::::::::::::::::::::::::::::::::::::::::::::::::::::::::::::::::::::::::::::::::::::::::::::::::::::::::::::::::::::::::::::::::::::::::::::::::::::::::::::::::::::::::::::::::::::::::::::::::::::::::::::::::::::::::::::::::::::::::::::::::::::::::::::::::::::::::::::::::::::::::::::::::::::

@D0950.29_106216

GATGAACGCTAGCTACAGGCTTAACACATGCAAGTCGAGGGGTAGCATGAAACTTAGCAATAAGTTTTGATGACGACCGGCGCACGGGTGAGTAACACGTATCCAACCTGCCTTTTACTCATGGATAGCCTTCTGAAAAGAAGATTAATACATGATGGTATTCAGAGTTTTCATGGACACTGAATTAAAGATTTTATCGGTAAGAGATGGGGATGCGTTCCATTAGATAGTAGGCGGGGTAACGGCCCACCTAGTCAACGATGGATAGGGGTTCTGAGAGGAAGGTCCCCCACATTGGAACTGAGACACGGTCCAAACGTCCTACGGGAGGCAGCAGTGAGGAATATTGGTCAATGGACGTAAGTCTGAACCAGCCAAGTAGCGTGAAGGATGAAGGCTCTATGGGTCGTAAACTTCTTTTTATAAAAGG

+

::::::::::::::::::::::::::::::::::::::::::::::::::::::::::::::::::::::::::::::::::::::::::::::::::::::::::::::::::::::::::::::::::::::::::::::::::::::::::::::::::::::::::::::::::::::::::::::::::::::::::::::::::::::::::::::::::::::::::::::::::::::::::::::::::::::::::::::::::::::::::::::::::::::::::::::::::::::::::::::::::::::::::::::::::::::::::::::::::::::::::::::::::::::::::::::::::::::::::::::::::::::::::::::::::::::::::::::

@D0950.29_106234

GATGAACGCTAGCTACAGGCTTAACACATGCAAGTCGAGGGGTAGCATGAAACTTAGCAATAAGTTTTGATGACGACCGGCGCACGGGTGAGTAACACGTATCCAACCTGCCTTTTACTCATGGATAGCCTTCTGAAAAGAAGATTAATACATGATGGTATTCAGAGTTTTCATGGACACTGAATTAAAGATTTTATCGGTAAGAGATGGGGATGCGTTCCATTAGATAGTAGGCGGGGTAACGGCCCACCTAAGTCAACGATGGATAGGGGTTCTGAGAGGAAGGTCCCCCACATTGGAACTGAGACACGGTCCAAACGTCCTACGGGAGGCAGCAGTGAGGAATATTGGTCAATGGACGTAAGTCTGAACCAGCCAAGTAGCGTGAAGGATGAAGGCTCTATGGGTCGTAAACTTCTTTTGTCAGGGAGCAACGACATCCACGAGTGGGTGAATGAGAGTACCTGAAGAAAAAGCATCGGCTAACTCCGTGCCAGCAGCCGCGGTAA

+

:::::::::::::::::::::::::::::::::::::::::::::::::::::::::::::::::::::::::::::::::::::::::::::::::::::::::::::::::::::::::::::::::::::::::::::::::::::::::::::::::::::::::::::::::::::::::::::::::::::::::::::::::::::::::::::::::::::::::::::::::::::::::::::::::::::::::::::::::::::::::::::::::::::::::::::::::::::::::::::::::::::::::::::::::::::::::::::::::::::::::::::::::::::::::::::::::::::::::::::::::::::::::::::::::::::::::::::::::::::::::::::::::::::::::::::::::::::::::::::::::::::::::::::::::::::::::::::

@D0950.29_106283

GACGAACGCTAGCGACAGGCCTAACACATGCAAGTCGAGGGGTAGCACAAGGTAGCAATACTGAGGTGACGACCGGCGCACGGGTGAGTAACGCGTATGCAACCTACCTGTAAGAGTGGGATAGCCTCTCGAAAGAGAGATTAATACCGCATAATACCATTTCACTGCATGGTGAGATGGTTAAAGATTTATTGCTTACAGATGGGCATGCGTAACATTAGCTAGTAGGTGGGGTAACGGCTCACCTAGGCGACGATCCCTAGCTGGTCTGAGAGGATGACCAGCCACACTGGAACTGAGACACGGTCCAGACTCCTACGGGAGGCAGCAGTGGGGAATATTGCACAATGGGCGCAAGCCTGATGCAGCCATGCCGCGTGTATGAAGAAGGCCTTCGGGTTGTAAAGTACTTTCAGCGAGGAGGAAGGCGTTGTGGTTAATAACCGCAACGATTGACGTTACTCGCAGAAGAAGCACCGGCTAACTCCGTGCCAGCAGCCGCGGTAA

+

:::::::::::::::::::::::::::::::::::::::::::::::::::::::::::::::::::::::::::::::::::::::::::::::::::::::::::::::::::::::::::::::::::::::::::::::::::::::::::::::::::::::::::::::::::::::::::::::::::::::::::::::::::::::::::::::::::::::::::::::::::::::::::::::::::::::::::::::::::::::::::::::::::::::::::::::::::::::::::::::::::::::::::::::::::::::::::::::::::::::::::::::::::::::::::::::::::::::::::::::::::::::::::::::::::::::::::::::::::::::::::::::::::::::::::::::::::::::::::::::::::::::::::::::::::::::::::

@D0950.29_106334

GATGAACGCTAGCTACAGGCTTAACACATGCAAGTCGAGGGGTAGCATGAAACTTAGCAATAAGTTTTGATGACGACCGGCGCACGGGTGAGTAACACGTATCCAACCTGCCTTTTACTCATGGATAGCCTTCTGAAAAGAAGATTAATACATGATGGTATTCAGAGTTTTCATGGACACTGAATTAAAGATTTTATCGGTAAGAGATGGGGATGCGTTCCATTAGATAGTAGGCGGGGTAACGGCCCACCTAGTCAACGATGGATAGGGGTTCTGAGAGGAAGGTCCCCCACATTGGAACTGAGACACGGTCCAAACGTCCGTACGGGAGGCAGCAGTGAGGAATATTGGTCAATGGACGTAAGTCTGAACCAGCCAAGTAGCGTGAAGGATGAAGGCTCTATGGGTCGTAAACTTCTTTTTATAAAAGGAATAAAGTATGCCACGTGTGGTGTTTTTGTATGTACTTTATGAATAAGGATCGGCTAACTCCGTGCCAGCAGCCGC

+

:::::::::::::::::::::::::::::::::::::::::::::::::::::::::::::::::::::::::::::::::::::::::::::::::::::::::::::::::::::::::::::::::::::::::::::::::::::::::::::::::::::::::::::::::::::::::::::::::::::::::::::::::::::::::::::::::::::::::::::::::::::::::::::::::::::::::::::::::::::::::::::::::::::::::::::::::::::::::::::::::::::::::::::::::::::::::::::::::::::::::::::::::::::::::::::::::::::::::::::::::::::::::::::::::::::::::::::::::::::::::::::::::::::::::::::::::::::::::::::::::::::::::::::::::::::::::::

@D0950.29_106377

GATGAACGCTAGCTACAGGCTTAACACATGCAAGTCGAGGGGTAGCATGAAACTTAGCAATAAGTTTTGATGACGACCGGCGCACGGGTGAGTAACACGTATCCAACCTGCCTTTTACTCATGGATAGCCTTCTGAAAAGAAGATTAATACATGATGGTATTCAGAGTTTTCATGGACACTGAATTAAAGATTTTATCGGTAAGAGATGGGGATGCGTTCCATTAGATAGTAGGCGGGGTAACGGCCCACCTAGTCAACGATGGATAGGGGTTCTGAGAGGAAGGTCCCCACATTGGAACTGAGACACGGTCCAAACGTCCTACGGGAGGCAGCAGTGAGAATATTGGTCAATGGACGTAAGTCTGAACCAGCCAAGTAGCGTGAAGGATGAAGGCTCTATGGG

+

::::::::::::::::::::::::::::::::::::::::::::::::::::::::::::::::::::::::::::::::::::::::::::::::::::::::::::::::::::::::::::::::::::::::::::::::::::::::::::::::::::::::::::::::::::::::::::::::::::::::::::::::::::::::::::::::::::::::::::::::::::::::::::::::::::::::::::::::::::::::::::::::::::::::::::::::::::::::::::::::::::::::::::::::::::::::::::::::::::::::::::::::::::::::::::::::::::::::::::::::::::

@D0950.29_106379

ATTGAACGCTGGCGGCATGCCTTACACATGCAAGTCGAACGGTAACAGGTCTTCGGATGCTGACGAGTGGCGAACGGGTGAGTAATACATCGGAACGTGCCCGATCGTGGGGGATAACGAAGCGAAAGCTTTGCTAATACCGCATAAGATCTACGGATGAAAGCAGGGGACCGCAAGGCCTTGCGCGAACGGAGCGGCCGATGGCAGATTAGGTAGTTGGTGGGATAAAAGCTTACCAAGCCGACGATCTGTAGCTGGTCTGAGAGGACGACCAGCCACACTGGGACTGAGACACGGCCCAGACTCCTACGGGAGGCAGCAGTGGGGAATTTTGGACAATGGGCGAAAGCCTGATCCAGCCATGCCGCGTGCAGGATGAAGGCCTTCGGGTTGTAAACTGCTTTTGTACGGAACGAAAATGACTCTGGTTAATACCTGGGGTCCATGACGGTACCGTAAGAATAAGCACCGGCTAACTACGTGCCAGCAGCCGCGGTAA

+

:::::::::::::::::::::::::::::::::::::::::::::::::::::::::::::::::::::::::::::::::::::::::::::::::::::::::::::::::::::::::::::::::::::::::::::::::::::::::::::::::::::::::::::::::::::::::::::::::::::::::::::::::::::::::::::::::::::::::::::::::::::::::::::::::::::::::::::::::::::::::::::::::::::::::::::::::::::::::::::::::::::::::::::::::::::::::::::::::::::::::::::::::::::::::::::::::::::::::::::::::::::::::::::::::::::::::::::::::::::::::::::::::::::::::::::::::::::::::::::::::::::::::::::::::::

@D0950.29_106386

ATTGAACGCTGGCGGCAGGCCTAACACATGCAAGTCGAACGGTAGCACAGAGGAGCTTGCTCCTTGGGTGACGAGTGGCGGACGGGTGAGTAATGTCTGGGAAACTGCCCGATGGAGGGGGATAACTACTGGAAACGGTAGCTAATACCGCATAACGTCGCAAGACCAAAGAGGGGGACCTTCGGGCCTCTTGCCATCGGATGTGCCCAGATGGGATTAGCTAGTAGGTGGGGTAACGGCTCACCTAGGCGACGATCCCTAGCTGGTCTGAGAGGATGACCAGCCACACTGGAACTGAGACACGGTCCAGACTCCTACGGGAGGCAGCAGTGGGGAATATTGCACAATGGGCGCAAGCCTGATGCAGCCATGCCGCGTGTATGAAGAAGGCCTTCGGGTTGTAAAGTACTTTCAGCGAGGAGGAAGGCGGTGAGGTTAATAACCTCATCGATTGACGTTACTCGCAGAAGAAGCACCGGCTAACTCCGTGCCAGCAGCCGCGGTAA

+

::::::::::::::::::::::::::::::::::::::::::::::::::::::::::::::::::::::::::::::::::::::::::::::::::::::::::::::::::::::::::::::::::::::::::::::::::::::::::::::::::::::::::::::::::::::::::::::::::::::::::::::::::::::::::::::::::::::::::::::::::::::::::::::::::::::::::::::::::::::::::::::::::::::::::::::::::::::::::::::::::::::::::::::::::::::::::::::::::::::::::::::::::::::::::::::::::::::::::::::::::::::::::::::::::::::::::::::::::::::::::::::::::::::::::::::::::::::::::::::::::::::::::::::::::::::::::

@D0950.29_106390

GATGAACGCTAGCGACAGGCCTAACACATGCAAGTCGAGGGGTAGCACAAGGTAGCAATACTGAGGTGACGACCGGCGCACGGGTGAGTAACGCGTATGCAACCTACCTGTAAGAGTGGGATAGCCTCTCGAAAGAGAGATTAATACCGCATAATACCATTTCACTGCATGGTGAGATGGTTAAAGATTTATTGCTTACAGATGGGCATGCGTAACATTAGCTAGTTGGTGAGGTAACGGCTCACCAAGGCAACGATGTTTAGGGGTTCTGAGAGGAAGGTCCCCCACACTGGTACTGAGACACGGACCAGACTCCTACGGGAGGCAGCAGTGAGGAATATTGGTCAATGGACGAGAGTCTGAACCAGCCAAGTCGCGTGAAGGATGAAGGTCTTATGGATTGTAAACTTCTTTTATACGGGAATAAAAAATGCCACGTGTGGCATATTGCATGTACCGTATGAATAAGGATCGGCT

+

:::::::::::::::::::::::::::::::::::::::::::::::::::::::::::::::::::::::::::::::::::::::::::::::::::::::::::::::::::::::::::::::::::::::::::::::::::::::::::::::::::::::::::::::::::::::::::::::::::::::::::::::::::::::::::::::::::::::::::::::::::::::::::::::::::::::::::::::::::::::::::::::::::::::::::::::::::::::::::::::::::::::::::::::::::::::::::::::::::::::::::::::::::::::::::::::::::::::::::::::::::::::::::::::::::::::::::::::::::::::::::::::::::::::::::::::::::::::::::::

@D0950.29_106412

GATGAACGCTAGCGACAGGCCTAACACATGCAAGTCGAGGGGGTAGCACAAGGTAGCAATACTGAGGTGACGACCGGCGCACGGGTGAGTAACGCGTATGCAACCTGCCTGTAAGAGTGGGGATAGCCTCTCGAAAGAGAGATTAATACCGCATAATACCATTAAGCCGCATGGCTTGATGGTTAAAGATTTATTGCTTACAGATGGGCATGCGTAACATTAGCTTGTTGGTGAGGTAACGGCTCACCAAGGCAACGATGTTTAGGGGTTCTGAGAGGAAGGTCCCCCACACTGGTACTGAGACACGGGACCAGACTCCTACGGGGAGGCAGCAGTGAGGAATATTGGGTCAATGGACGAGAGTCTGAACCAGCCAAGTCGCGTGAAGGATGAAGGTCTTATTGGATTGTAAACTTCTTTTATACGGGGAATAAAAAAGGCCACGTGTGGTTTATTGCATGTA

+

:::::::::::::::::::::::::::::::::::::::::::::::::::::::::::::::::::::::::::::::::::::::::::::::::::::::::::::::::::::::::::::::::::::::::::::::::::::::::::::::::::::::::::::::::::::::::::::::::::::::::::::::::::::::::::::::::::::::::::::::::::::::::::::::::::::::::::::::::::::::::::::::::::::::::::::::::::::::::::::::::::::::::::::::::::::::::::::::::::::::::::::::::::::::::::::::::::::::::::::::::::::::::::::::::::::::::::::::::::::::::::::::::::::::::::::::

@D0950.29_106433

GATGAACGCTAGCGACAGGCCTAACACATGCAAGTCGAGGGGTAGCACAAGGTAGTAATACTGAGGTGACGACCGGCGCACGGGTGAGTAACGCGTATGCAACCTACCTGTAAGAGTGGGATAGCCTCTCGAAAGAGAGATTAATACCGCATAATACCATTTCACTGCATGGTGAGATGGTTAAAGATTTATTGCTTACAGATGGGCATGCGTAACATTAGCTAGTTGGTGAGGTAACGGCTCACCAAGGCAACGATGTTTAGGGGTTCTGAGAGGAAGGTCCCCCACACTGGTACTGAGACACGGACCAGACTCCTACGGGAGGCAGCAGTGAGGAATATTGGTCAATGGACGAGAGTCTGAACCAGCCAAGTCGCGTGAAGGATGAAGGTCTTATGGATTGTAAACTTCTTTTATACGGGAATAAAAAGAGCCACGTGTGGCTTATTGCATGTACCGTATGAATAAGGATCGGCTAACTCCGTGCCAGCAGCCGCGGTAACTGGACTGCCAAGG

+

::::::::::::::::::::::::::::::::::::::::::::::::::::::::::::::::::::::::::::::::::::::::::::::::::::::::::::::::::::::::::::::::::::::::::::::::::::::::::::::::::::::::::::::::::::::::::::::::::::::::::::::::::::::::::::::::::::::::::::::::::::::::::::::::::::::::::::::::::::::::::::::::::::::::::::::::::::::::::::::::::::::::::::::::::::::::::::::::::::::::::::::::::::::::::::::::::::::::::::::::::::::::::::::::::::::::::::::::::::::::::::::::::::::::::::::::::::::::::::::::::::::::::::::::::::::::::::::::::::

@D0950.29_106451

GATGAACGCTAGCGACAGGCCTAACACATGCAAGTCGAGGGGTAGCACAAGGTAGTAATACTGAGGTGACGACCGGCGCACGGGTGAGTAACGCGTATGCAACCTGCCTGTAAGAGTGGGATAGCCTCTCGAAAGAGAGATTAATACCGCATGATACTATGAAGCCGCATGGTTTTATAGTTAAAGATTTATTGCTTACAGATGGGCATGCGTAACATTAGCTTGTTGGTGAGGTAACGGCTCACCAAGGCAACGATGTTTAGGGGTTCTGAGAGGAAGGTCCCCCACACTGGTACTGAGACACGGACCAGACTCCTACGGGAGGCAGCAGTGAGGAATATTGGTCAATGGACGAGAGTCTGAACCAGCCAAGTCGCGTGAAGGATGAAGGTCTTATGGATTGTAAACTTCTTTTATACGGGAATAAAAATGCCACGAGTGGCATATTGCATGTACCGTATGAATAAGGATCGGCTAACTCCGTGCCAGCAGCCGCGGTAA

+

:::::::::::::::::::::::::::::::::::::::::::::::::::::::::::::::::::::::::::::::::::::::::::::::::::::::::::::::::::::::::::::::::::::::::::::::::::::::::::::::::::::::::::::::::::::::::::::::::::::::::::::::::::::::::::::::::::::::::::::::::::::::::::::::::::::::::::::::::::::::::::::::::::::::::::::::::::::::::::::::::::::::::::::::::::::::::::::::::::::::::::::::::::::::::::::::::::::::::::::::::::::::::::::::::::::::::::::::::::::::::::::::::::::::::::::::::::::::::::::::::::::::::::::::::::::

@D0950.29_106456

GATGAACGCTGGCGGCGTGCTTAACACATGCAAGTCGAACGAAGGTTAAGGAGCTTGCTCCTTGATACTTAGTGGCGGACGGGTGAGTAACGCGTGGGTAACCTACCCTATGCAGGGGGATAACGTTTGGAAACGAACGCTAATACCGCATAAACTATCGGTAGTCGCATGACTATTATAGCAAAGATTTATCAGCATAGGATGGACCCGCGTTGGATTAGCTAGTTGGTGAGATAACAGCCCACCAAGGCAACGATCCATAGCCGGCCTGAGAGGGTGAACGGCCACATTGGGACTGAGACACGGCCCAAACTCCTACGGGAGGCAGCAGTGGGGAATATTGCACAATGGGCGAAAGCCTGATGCAGCAACGCCGCGTGAAGGATGAAGGTCTTCGGATTGTAAACTTCTATCAGTAGGGAAGAAAGGTAACTTCGGTTACCCTGACGGTACCTAACTAAGAAGCTCCGGCTAACTACGTGCCAGCAGCCGCGGT

+

::::::::::::::::::::::::::::::::::::::::::::::::::::::::::::::::::::::::::::::::::::::::::::::::::::::::::::::::::::::::::::::::::::::::::::::::::::::::::::::::::::::::::::::::::::::::::::::::::::::::::::::::::::::::::::::::::::::::::::::::::::::::::::::::::::::::::::::::::::::::::::::::::::::::::::::::::::::::::::::::::::::::::::::::::::::::::::::::::::::::::::::::::::::::::::::::::::::::::::::::::::::::::::::::::::::::::::::::::::::::::::::::::::::::::::::::::::::::::::::::::::::::::::::::

@D0950.29_106473

GATGAACGCTAGCGACAGGCCTAACACATGCAAGTCGAGGGGTAGCACAAGGTAGTAATACTGAGGTGACGACCGGCGCACGGGTGAGTAACGCGTATGCAACCTACCTGTAAGAGTGGGATAGCCTCTCGAAAGAGAGATTAATACCGCATAATACCATTTCACTGCATGGTGAGATGGTTAAAGATTTATTGCTTACAGATGGGCATGCGTAACATTAGCTAGTTGGTGAGGTAACGGCTCACCAAGGCAACGATGTTTAGGGGTTCTGAGAGGAAGGTCCCCCACACTGGTACTGAGACACGGACCAGACTCCTACGGGAGGCAGCAGTGAGGAATATTGGTCAATGGACGAGAGTCTGAACCAGCCAAGTCGCGTGAAGGATGAAGGTCTTATGGATTGTAAACTTCTTTTATACGGGAATAAAAATGCCACGTGTGGCATATTGCATGTACCGTATGAATAAGGATCGGCTAACTCCGTGCCAGCAGCCGCGGTAA

+

:::::::::::::::::::::::::::::::::::::::::::::::::::::::::::::::::::::::::::::::::::::::::::::::::::::::::::::::::::::::::::::::::::::::::::::::::::::::::::::::::::::::::::::::::::::::::::::::::::::::::::::::::::::::::::::::::::::::::::::::::::::::::::::::::::::::::::::::::::::::::::::::::::::::::::::::::::::::::::::::::::::::::::::::::::::::::::::::::::::::::::::::::::::::::::::::::::::::::::::::::::::::::::::::::::::::::::::::::::::::::::::::::::::::::::::::::::::::::::::::::::::::::::::::::::::

@D0950.29_106489

GATGAACGCTAGCGACAGGCTTAACACATGCAAGTCGAGGGGCAGCACAAGGTAGCAATACTGAGGTGGCGACCGGCGCACGGGTGAGTAACGCGTATGCAACCTACCTCTTAGCGGGGGATAACCCGGCGAAAGTCGGACTAATACCGCATAATACTCTTTCTCCGCATGGAGGGAGATTTAAAGATTAATTGCTAAGAGATGGGGATGCGTTCCATTAGATAGTAGGCGGGTAACGCCCACCTAGTCAACGATGGATAGGGGTTCTGAGAGGAAGGTCCCCCACATTGGAACTGAGACACGGTCCAAACTCCTACGGGAGGCAGCAGTGAGGAATATTGGTCAATGGACGTAAGTCTGAACCAGCCAAGTAGCGTGAAGGATGAAGGCTCTATGGGTCGTAAACTTCTTTTATAAAGGAATAAAGTATGCCACGTGTGGTGTTTTTGTATGTA

+

:::::::::::::::::::::::::::::::::::::::::::::::::::::::::::::::::::::::::::::::::::::::::::::::::::::::::::::::::::::::::::::::::::::::::::::::::::::::::::::::::::::::::::::::::::::::::::::::::::::::::::::::::::::::::::::::::::::::::::::::::::::::::::::::::::::::::::::::::::::::::::::::::::::::::::::::::::::::::::::::::::::::::::::::::::::::::::::::::::::::::::::::::::::::::::::::::::::::::::::::::::::::::::::::::::::::::::::::::::::::::::::::::::::::

@D0950.29_106587

GATGAACGCTAGCGACAGGCCTAACACATGCAAGTCGAGGGGTAGCACAAGGTAGCAATACTGAGGTGACGACCGGCGCACGGGTGAGTAACGCGTATGCAACCTACCTGTAAGAGTGGGATAGCCTCTCGAAAGAGAGATTAATACCGCATAATACCATTTCACTGCATGGTGAGATGGTTAAAGATTTATTGCTTACAGATGGGCATGCGTAACATTAGCTAGTTGGTGAGGTAACGGCTCACCAAGGCAACGATGTTTAGGGGTTCTGAGAGGAAGGTCCCCCGACACTGGTACTGAGACACGGACCAGACTCCTACGGGAGGCAGCAGTGAGGAATATTGGTCAATGGACGAGAGTCTGAACCAGCCAAGTCGCGTGAAGGATGAAGGTCTTATGGATTGTAAACTTCTTTTATACGGGAATAAAAATGCCACGTGTGGCATATTGCATGTACCGTATGAATAAGGATCGGCTAACTCCGTGCCAGCAGCCGCGGTAA

+

::::::::::::::::::::::::::::::::::::::::::::::::::::::::::::::::::::::::::::::::::::::::::::::::::::::::::::::::::::::::::::::::::::::::::::::::::::::::::::::::::::::::::::::::::::::::::::::::::::::::::::::::::::::::::::::::::::::::::::::::::::::::::::::::::::::::::::::::::::::::::::::::::::::::::::::::::::::::::::::::::::::::::::::::::::::::::::::::::::::::::::::::::::::::::::::::::::::::::::::::::::::::::::::::::::::::::::::::::::::::::::::::::::::::::::::::::::::::::::::::::::::::::::::::::::::

@D0950.29_106608

GATGAACGCTAGCGACAGGCCTAACACATGCAAGTCGAGGGGTAGCACAAGGGAGCTTGCTTCTGAGGTGACGACCGGCGCACGGGTGAGTAACGCGTATGCAACCTGCCTATAAGAAGGGGATAGCCTCTCGAAAGAGAGATTAATACCGTATAACACTATGAAGCCGCATGGTTTTATAGTTAAAGATTTATTGCTTATAGATGGGCATGCGTAACATTAGCTAGTTGGTAAGGTAACGGCTTACCAAGGCAACGATGTTTAGGGGTTCTGAGAGGAAGGTCCCCCACACTGGTACTGAGACACGGACCAGACTCCTACGGGAGGCAGCAGTGAGGAATATTGGTCAATGGACGAGAGTCTGAACCAGCCAAGTCGCGTGAAGGATGAAGGTCTTATGGATTGTAAACTTCTTTTATACGGGGAAATAAAAAACTACCACGTGTGGTATATTGGCAATGTACCGTATGAATAAGGATCGGCTAACTCCGTGCCAGCAGCCGC

+

::::::::::::::::::::::::::::::::::::::::::::::::::::::::::::::::::::::::::::::::::::::::::::::::::::::::::::::::::::::::::::::::::::::::::::::::::::::::::::::::::::::::::::::::::::::::::::::::::::::::::::::::::::::::::::::::::::::::::::::::::::::::::::::::::::::::::::::::::::::::::::::::::::::::::::::::::::::::::::::::::::::::::::::::::::::::::::::::::::::::::::::::::::::::::::::::::::::::::::::::::::::::::::::::::::::::::::::::::::::::::::::::::::::::::::::::::::::::::::::::::::::::::::::::::::::::

@D0950.29_106609

GATGAACGCTAGCTACAGGCTTAACACATGCAAGTCGAGGGGTAGCATGAAACTTAGCAATAAGTTTTGATGACGACCGGCGCACGGGTGAGTAACACGTATCCAACCTGCCTTTTACTCATGGATAGCCTTCTGAAAAGAAGATTAATACATGATGGTATTCAGAGTTTTCATGGACACTGAATTAAAGATTTTATCGGTAAGAGATGGGGATGCGTTCCATTAGATAGTAGGCGGGGTAACGGCCCACCTAGTCAACGATGGATAGGGGTTCTGAGAGGAAGGTCCCCCACATTGGAACTGAGACACGGTCCAAACGTCCGTACGGGAGGCAGCAGTGAGGAATATTGGTCAATGGACGTAAGTCTGAACCAGCCAAGTAGCGTGAAGGATGAAGGCTCTATGGGTCGTAAACTTCTTTTTATAAAA

+

:::::::::::::::::::::::::::::::::::::::::::::::::::::::::::::::::::::::::::::::::::::::::::::::::::::::::::::::::::::::::::::::::::::::::::::::::::::::::::::::::::::::::::::::::::::::::::::::::::::::::::::::::::::::::::::::::::::::::::::::::::::::::::::::::::::::::::::::::::::::::::::::::::::::::::::::::::::::::::::::::::::::::::::::::::::::::::::::::::::::::::::::::::::::::::::::::::::::::::::::::::::::::::::::::::::::::::::

@D0950.29_106654

GATGAACGCTAGCTACAGGCTTAACACATGCAAGTCGAGGGGTAGCATGAAACTTAGCAATAAGTTTTGATGACGACCGGCGCACGGGTGAGTAACACGTATCCAACCTGCCTTTTACTCATGGATAGCCTTCTGAAAAGAAGATTAATACATGATGGTATTCAGAGTTTTCATGGACACTGAATTAAAGATTTATCGGTAAGAGATGGGGATGCGTTCCATTAGATAGTAGGTCGGGGTAACGGCCCTACCTAGTCAACGATGGATAGGGGTTCTGAGAGGAAGGTCCCCCACATTGGAACTGAGACACGGTCCAAACGTCCTACGGGAGGCAGCAGTGAGGAATATTGGTCAATGGACGTAAGTTTGAACCAGCCAAGTAGCGTGAA

+

:::::::::::::::::::::::::::::::::::::::::::::::::::::::::::::::::::::::::::::::::::::::::::::::::::::::::::::::::::::::::::::::::::::::::::::::::::::::::::::::::::::::::::::::::::::::::::::::::::::::::::::::::::::::::::::::::::::::::::::::::::::::::::::::::::::::::::::::::::::::::::::::::::::::::::::::::::::::::::::::::::::::::::::::::::::::::::::::::::::::::::::::::::::::::::::::::::::

@D0950.29_106694

ATTGAACGCTGGCGGCAGGCCTAACACATGCAAGTCGAACGGTAGCACAGAGAGCTTGCTCTTGGGTGACGAGTGGCGGACGGGTGAGTAATGTCTGGGAAACTGCCCGATGGAGGGGGATAACTACTGGAAACGGTAGCTAATACCGCATAATGTCGCAAGACCAAAGAGGGGGACCTTCGGGCCTCTTGCCATCGGATGTGCCCAGATGGGATTAGCTAGTAGGTGGGGTAACGGCTCACCTAGGCGACGATCCCTAGCTGGTCTGAGAGGATGACCAGCCACACTGGAACTGAGACACGGTCCAGACTCCTACGGGAGGCAGCAGTGGGGAATATTGCACAATGGGCGCAAGCCTGATGCAGCCATGCCGCGTGTATGAAGAAGGCCTTCGGGTTGTAAAGTACTTTCAGCGAGGAGGAAGGCGTTGTGGTTAATAACCGCAGCGATTGACGTTACTCGCAGAAGAAGCACCGGCTAACTCCGTGCCAGCAGCCGCGGTAA

+

::::::::::::::::::::::::::::::::::::::::::::::::::::::::::::::::::::::::::::::::::::::::::::::::::::::::::::::::::::::::::::::::::::::::::::::::::::::::::::::::::::::::::::::::::::::::::::::::::::::::::::::::::::::::::::::::::::::::::::::::::::::::::::::::::::::::::::::::::::::::::::::::::::::::::::::::::::::::::::::::::::::::::::::::::::::::::::::::::::::::::::::::::::::::::::::::::::::::::::::::::::::::::::::::::::::::::::::::::::::::::::::::::::::::::::::::::::::::::::::::::::::::::::::::::::::::

@D0950.29_106774

GATGAACGCTAGCTACAGGCTTAACACATGCAAGTCGAGGGGTAGCATGAAACTTAGCAATAAGTTTTGATGACGACCGGCGCACGGGTGAGTAACACGTATCCAACCTGCCTTTTACTCATGGATAGCCTTCTGAAAAGAAGATTAATACATGATGGTATTCAGAGTTTTCATGGACACTGAATTAAAGATTTTATCGGTAAGAGATGGGGATGCGTTCCATTAGATAGTAGGCGGGGTAACGGCCCACCTAGTCAACGATGGATAGGGGTTCTGAGAGGAAGGTCCCCCACATTGGAACTGAGACACGGTCCAAACGTCCTACGGGAGGCAGCAGTGAGGAATATTGGTCAATGGACGTAAGTCTGAACCAGCCAAGTAGCGTGAAGGATGAAGGCTCTATGGGTCGTAAACTTCTTTTTATAAAAGGAAATAAAGTATGCCACGTGTGGTGTTTTTGTATGTACTTTATGAATAAGGATCGGCTAACTCCGTGCCAGCAGCCGCGGT

+

::::::::::::::::::::::::::::::::::::::::::::::::::::::::::::::::::::::::::::::::::::::::::::::::::::::::::::::::::::::::::::::::::::::::::::::::::::::::::::::::::::::::::::::::::::::::::::::::::::::::::::::::::::::::::::::::::::::::::::::::::::::::::::::::::::::::::::::::::::::::::::::::::::::::::::::::::::::::::::::::::::::::::::::::::::::::::::::::::::::::::::::::::::::::::::::::::::::::::::::::::::::::::::::::::::::::::::::::::::::::::::::::::::::::::::::::::::::::::::::::::::::::::::::::::::::::::::::

@D0950.29_106804

GATGAACGCTAGCTACAGGCTTAACACATGCAAGTCGAGGGGTAGCATGAAACTTAGCAATAAGTTTTGATGACGACCGGCGCACGGGTGAGTAACATGTATCCAACCTGCCTTTTACTCATGGATAGCCTTCTGAAAAGAAGATTAATACATGATGGTATTCAGAGTTTTCATGGACACTGAATTAAAGATTTTATCGGTAAGAGATGGGGATGCGTTCCATTAGATAGTAGGCGGGGTAACGGCCCACCTAGTCAACGATGGATAGGGGTTCTGAGAGGAAGGTCCCCCACATTGGAACTGAGACACGGTCCAAACGTCCTACGGGAGGCAGCAGTGAGGAATATTGGTCAATGGACGTAAGTCTGAACCAGCCAAGTAGCGTGAAGGATGAAGGCTCTATGGGTCGTAAACTTCTTTTATAAAAGGAATAAAGTATGCCACGTGTGGTGTTTTTGTATGTACTTTATGAATAAGGATCGGCTAACTCCGTGCCAGCAGCCGC

+

:::::::::::::::::::::::::::::::::::::::::::::::::::::::::::::::::::::::::::::::::::::::::::::::::::::::::::::::::::::::::::::::::::::::::::::::::::::::::::::::::::::::::::::::::::::::::::::::::::::::::::::::::::::::::::::::::::::::::::::::::::::::::::::::::::::::::::::::::::::::::::::::::::::::::::::::::::::::::::::::::::::::::::::::::::::::::::::::::::::::::::::::::::::::::::::::::::::::::::::::::::::::::::::::::::::::::::::::::::::::::::::::::::::::::::::::::::::::::::::::::::::::::::::::::::::::::

@D0950.29_106808

GATGAACGCTGGCGGCGTGCTTAACACATGCAAGTCGAACGAAGCACTTTGAAGAGCTTGCTCTTTAAAGTGACTGAGTGGCGGACGGGTGAGTAACGCGTGGGTAACCTGCCTCATACAGGGGGATAACAGTTAGAAATGACTGCTAACACCGCATAACCCGCTAGTGTCGCATGACACAGACGGAAAATATTTATAGGTATGAGATGGGCCCGCGTCTGATTAGCTAGTTGGTGGGGTAACGGCCTACCAAGGCAACGATCAGTAGCCGACTTGAGAGAGTGATCGGCCACATTGGGACTGAGACACGGCCCAAACTCCTACGGGAGGCAGCAGTGGGGAATATTGGACAATGGGGGAAACCCTGATCCAGCGACGCCGCGTGAGTGAAGAAGTATTTCGGTATGTAAAGCTCTATCAGCAGGGGAAGATAATGACAGTACCTGACTAAGAAGCCCCGGCTAACTACGTGCCAGCAGCCGCGGTAA

+

::::::::::::::::::::::::::::::::::::::::::::::::::::::::::::::::::::::::::::::::::::::::::::::::::::::::::::::::::::::::::::::::::::::::::::::::::::::::::::::::::::::::::::::::::::::::::::::::::::::::::::::::::::::::::::::::::::::::::::::::::::::::::::::::::::::::::::::::::::::::::::::::::::::::::::::::::::::::::::::::::::::::::::::::::::::::::::::::::::::::::::::::::::::::::::::::::::::::::::::::::::::::::::::::::::::::::::::::::::::::::::::::::::::::::::::::::::::::::::::::::::::::

@D0950.29_106867

GATGAACGCTAGCTACAGGCTTAACACATGCAAGTCGAGGGGTAGCATGAAACTTAGCAATAAGTTTTGATGACGACCGGCGCACGGGTGAGTAACACGTATCCAACCTGCCTTTTACTCATGGATAGCCTTCTGAAAAGAAGATTAATACATGATGGTATTCAGAGTTTTCATGGACACTGAATTAAAGATTTATCGGTAAGAGATGGGGATGCGTTCCATTAGATAGTAGGCGGGGTAACGGCCCACCTAGTCAACGATGGATAGGGGTTCTGAGAGGAAGGTCCCCACATTGAACTGAGACACGGTCCAAACGTCCTACGGGAGGCAGCAGTGAGGAATATTGGTCAATGGACGTAAGTCTGAACCAGCCAAGTAGCGTGAAGGATGAAGGCTC

+

:::::::::::::::::::::::::::::::::::::::::::::::::::::::::::::::::::::::::::::::::::::::::::::::::::::::::::::::::::::::::::::::::::::::::::::::::::::::::::::::::::::::::::::::::::::::::::::::::::::::::::::::::::::::::::::::::::::::::::::::::::::::::::::::::::::::::::::::::::::::::::::::::::::::::::::::::::::::::::::::::::::::::::::::::::::::::::::::::::::::::::::::::::::::::::::::::::::::::::::

@D0950.29_10694

GATGAACGCTAGCTACAGGCTTAACACATGCAAGTCGAGGGGTAGCATGAAACTTAGCAATAAGTTTTGATGACGACCGGCGCACGGGTGAGTAACACGTATCCAACCTGCCTTTTACTCATGGATAGCCTTCTGAAAAGAAGATTAATACATGATGGTATTCAGAGTTTCATGGACACTGAATTAAAGATTTATCGGTAAGAGATGGGGATGCGTTCCATTAGATAGTAGGCGGGGTAACGGCCCACCTAGTCAACGATGGATAGGGGTTCCTGAGAGGAAGGTCCCCCACATTGGAACTGAGACACGGTCCAAACGTCCGTACGGGAGGCAGCAGTGAGGAATATTGTCAATGGACGTAAGTCTGAACCAGCCAAGTAGCGTGAAGGATGAAGGCTCTATGGGTCGTAAACTTCTTTTATAAAGGAATAAA

+

:::::::::::::::::::::::::::::::::::::::::::::::::::::::::::::::::::::::::::::::::::::::::::::::::::::::::::::::::::::::::::::::::::::::::::::::::::::::::::::::::::::::::::::::::::::::::::::::::::::::::::::::::::::::::::::::::::::::::::::::::::::::::::::::::::::::::::::::::::::::::::::::::::::::::::::::::::::::::::::::::::::::::::::::::::::::::::::::::::::::::::::::::::::::::::::::::::::::::::::::::::::::::::::::::::::::::::::::::

@D0950.29_10699

GATGAACGCTAGCGACAGGCCATAAGCACATGCAAGGTCGAGGGGTAGCACAAGGAAGCTTGCTTCTGAGGTGACGACCGGCGCACGGGTGAGTAACGCGTATGCAACCTACCTGTAAGAGTGGGATAGCCTCTCGAAAGAGAGATTAATACCGCATAATACCATTTCACTGCATGGTGAGATGGTTAAAGATTTATTGCTTACAGATGGGCATGCGTAACATTAGCTAGTTGGTGAGGTAACGGCTCACCAAGGCAACGATGTTTAGGGGTTCTGAGAGGAAGGTCCCCCACACTGGTACTGAGACACGGACCAGACTCCTACGGGAGGCAGCAGTGAGGAATATTGGTCAATGGACGAGAGTCTGAACCAGCCAAGTCGCGTGAAGGATGAAGGTCTTAT

+

::::::::::::::::::::::::::::::::::::::::::::::::::::::::::::::::::::::::::::::::::::::::::::::::::::::::::::::::::::::::::::::::::::::::::::::::::::::::::::::::::::::::::::::::::::::::::::::::::::::::::::::::::::::::::::::::::::::::::::::::::::::::::::::::::::::::::::::::::::::::::::::::::::::::::::::::::::::::::::::::::::::::::::::::::::::::::::::::::::::::::::::::::::::::::::::::::::::::::::::::::

@D0950.29_106994

GATGAACGCTGGCGGCGTGCTTAACACATGCAAGTCGAACGAAGCACTTTGAAGAGCTTGCTCTTTAAAGTGACTGAGTGGCGGACGGGTGAGTAACGCGTGGGTAACCTGCCTCATACAGGGGGATAACAGTTAGAAATGACTGCTAACACCGCATAACCCGCTAGTGTCGCATGACACAGACGGAAAATATTTATAGGTATGAGATGGGCCCGCGTCTGATTACGCTAGTTGGTGGGGTAACGGCCTACCAAGGCAACGATCAGTAGCCGACTTGAGAGAGTGATCGGCCACATTGGGACTGAGACACGGCCCAAACTCCTACGGGAGGCAGCAGTGGGGAATATTGGACAATGGGGGAAACCCTGATCCAGCGACGCCGCGTGAGTGAAAGAAGTATTTTCGGGTATGTAAAAGCTCTATCAGCA

+

::::::::::::::::::::::::::::::::::::::::::::::::::::::::::::::::::::::::::::::::::::::::::::::::::::::::::::::::::::::::::::::::::::::::::::::::::::::::::::::::::::::::::::::::::::::::::::::::::::::::::::::::::::::::::::::::::::::::::::::::::::::::::::::::::::::::::::::::::::::::::::::::::::::::::::::::::::::::::::::::::::::::::::::::::::::::::::::::::::::::::::::::::::::::::::::::::::::::::::::::::::::::::::::::::::::::::::

@D0950.29_107022

GATGAACGCTAGCGACAGGCCTAACACATGCAAGTCGAGGGGCAGCAGGGGGTAGCAATACTCCGCTGGCGACCGGCGCACGGGTGAGTAACGCGTATGCAACCTACCTATCAGAGGGGAATAACCCGGCGAAAGTCGGACTAATACCGCATAATACTTTTTTCCTGCATGGGGAGAGAGTTAAAGATTTATCGCTGATGGATGGGCATGCGTTCCATTAGGTAGTTGGTAGAGGTAACGGCCTACCAAGCCATCGATGGATAGGGGTTCTGAGAGGAAGGTCCCCCACATTGGAACTGAGACACGGTCCAAACTCCTACGGGAGGCAGCAGTGAGGAATATTGGTCAATGGACGAGAGTCTGAACCAGCCAAGTCGCGTGAAGGAAGAAGGTTCTATGGATTGTAAACTTCTTTTATAGGGGAATAAAGTTAGGGACGTGTCCCTATTTGTATGTACCCTATGAATAAGCATCGGCTAACTCCGTGCCAGCAGCCG

+

:::::::::::::::::::::::::::::::::::::::::::::::::::::::::::::::::::::::::::::::::::::::::::::::::::::::::::::::::::::::::::::::::::::::::::::::::::::::::::::::::::::::::::::::::::::::::::::::::::::::::::::::::::::::::::::::::::::::::::::::::::::::::::::::::::::::::::::::::::::::::::::::::::::::::::::::::::::::::::::::::::::::::::::::::::::::::::::::::::::::::::::::::::::::::::::::::::::::::::::::::::::::::::::::::::::::::::::::::::::::::::::::::::::::::::::::::::::::::::::::::::::::::::::::::

@D0950.29_107090

GATGAACGCTGGCGGCGTGCTTAACACATGCAAGTCGAACGAAGCAACTTTCTTGCTTGCAAGAAAGTTGACTGAGTGGCGGACGGGTGAGTAACGCGTGGGTAACCTGCCTCATACAGGGGGATAACAGTTAGAAATGACTGCTAACACCGCATAACCCGCTAGTGTCGCATGACACAGACGGAAAATATTTATAGGTATGAGATGGGCCCGCGTCTGATTAGCTAGTTGGTGGGGTAACGGCCTACCAAGGCAACGATCAGTAGCCGACTTGAGAGAGTGATCGGCCACATTGGGACTGAGACACGGCCCAAACTCCTACGGGAGGCAGCAGTGGGGAATATTGGACAATGGGGGAAACCCTGATCCAGCGACGCCGCGTGAGTGAAGAAGTATTTCGGTATGTAAAGCTCTATCAGCAGGGGAAGATAATGACAGTACCTGACTAAGAAGCCCCGGCTAACTACGTGCCAGCAGCCGCGGTAA

+

::::::::::::::::::::::::::::::::::::::::::::::::::::::::::::::::::::::::::::::::::::::::::::::::::::::::::::::::::::::::::::::::::::::::::::::::::::::::::::::::::::::::::::::::::::::::::::::::::::::::::::::::::::::::::::::::::::::::::::::::::::::::::::::::::::::::::::::::::::::::::::::::::::::::::::::::::::::::::::::::::::::::::::::::::::::::::::::::::::::::::::::::::::::::::::::::::::::::::::::::::::::::::::::::::::::::::::::::::::::::::::::::::::::::::::::::::::::::::::::::::::::

@D0950.29_107097

GATGAACGCTGGCGGCGTGCTTAACACATGCAAGTCGAACGAAGCGCTGGAGGAGCTTGCTCCAAAGGTGACTGAGTGGCGGACGGGTGAGTAACGCGTGGGTAACCTGCCTTACACTGGGGGATAACAGTTGGAAACGACTGCTAATACCGCATAAGCGCACAGTATTGCATGATACAGTGTGAAAAACTCCGGTGGTGTAAGATGGACCCGCGTCTGATTAGCTAGTTGGTGAGGTAATGGCTCACCAAGGCAACGATCAGTAGCCGGCTTGAGAGAGTGAACGGCCACATTGGGACTGAGACACGGCCCAAACTCCTACGGGAGGCAGCAGTGGGGAATATTGCACAATGGGGGAAACCCTGATGCAGCAACGCCGCGTGAGTGAAGAAGTATTTCGGTATGTAAAGCTCTATCAGCAGGGAAGATAATGACGGTACCTGACTAAGAAGCCCCGGCTAACTACGTGCCAGCAGCCGCGGTAA

+

:::::::::::::::::::::::::::::::::::::::::::::::::::::::::::::::::::::::::::::::::::::::::::::::::::::::::::::::::::::::::::::::::::::::::::::::::::::::::::::::::::::::::::::::::::::::::::::::::::::::::::::::::::::::::::::::::::::::::::::::::::::::::::::::::::::::::::::::::::::::::::::::::::::::::::::::::::::::::::::::::::::::::::::::::::::::::::::::::::::::::::::::::::::::::::::::::::::::::::::::::::::::::::::::::::::::::::::::::::::::::::::::::::::::::::::::::::::::::::::::::::::

@D0950.29_107129

ATTGAACGCTGGCGGCATGCCTTACACATGCAAGTCGAACGGTAACAGGTCTTCGGATGCTGACGAGTGGCGAACGGGTGAGTAATACATCGGAACGTGCCCGATCGTGGGGGATAACGAAGCGAAAGCTTTGCTAATACCGCATACGATCTACGGATGAAAGCAGGGGACCGCAAGGCCTTGCGCGAACGGAGCGGCCGATGGCAGATTAGGTAGTTGGTGGGATAAAAGCTTACCAAGCCGACGATCTGTAGCTGGTCTGAGAGGACGACCAGCCACACTGGGACTGAGACACGGCCCAGACTCCTACGGGAGGCAGCAGTGGGGAATTTTGGACAATGGGCGAAAGCCTGATCCAGCCATGCCGCGTGCAGGATGAAGGCCTTCGGGTTGTAAACTGCTTTTGTACGGAACGAAAAGACTCTGGTTAATACCTGGGGTCCATGACGGTACCGTAAGAATAAGCACCGGCTAACTACGTGCCAGCAGCCGCGGTAA

+

::::::::::::::::::::::::::::::::::::::::::::::::::::::::::::::::::::::::::::::::::::::::::::::::::::::::::::::::::::::::::::::::::::::::::::::::::::::::::::::::::::::::::::::::::::::::::::::::::::::::::::::::::::::::::::::::::::::::::::::::::::::::::::::::::::::::::::::::::::::::::::::::::::::::::::::::::::::::::::::::::::::::::::::::::::::::::::::::::::::::::::::::::::::::::::::::::::::::::::::::::::::::::::::::::::::::::::::::::::::::::::::::::::::::::::::::::::::::::::::::::::::::::::::::::

@D0950.29_107133

GATGAACGCTAGCGACAGGCCTAACACATGCAAGTCGAGGGGCAGCGGGGGAGTAGCAATACTTCTGCCGGCGACCGGCGCACGGGTGAGTAACACGTATGCAACCTGCCCATAACAGGGGGATAATCGGAAGAAATTCCGTCTAATACCGCGTAATCCCGGATTTTCACATGAGAAATCGGGTAAAGAAGCAATTCGGTTATGGATGGGCATGCGGAACATTAGGTAGTTGGTGAGGTAACGGCTCACCAAGCCGACGATGTATAGGGGTTCTGAGAGGAAGGTCCCCCACACTGGTACTGAGACACGGACCAGACTCCTACGGGAGGCAGCAGTGAGGAATATTGCACAATGGGGGAAACCCTGATGCAGCAACGCCGCGTGAGTGATGACGGCCTTCGGGTTGTAAAACTCTGTCTTTGGGGGACGATAATGACGGTACCCAAGGAGGAAGCCACGGCTAACTACGTGCCAGCAGCCGCGGTAA

+

:::::::::::::::::::::::::::::::::::::::::::::::::::::::::::::::::::::::::::::::::::::::::::::::::::::::::::::::::::::::::::::::::::::::::::::::::::::::::::::::::::::::::::::::::::::::::::::::::::::::::::::::::::::::::::::::::::::::::::::::::::::::::::::::::::::::::::::::::::::::::::::::::::::::::::::::::::::::::::::::::::::::::::::::::::::::::::::::::::::::::::::::::::::::::::::::::::::::::::::::::::::::::::::::::::::::::::::::::::::::::::::::::::::::::::::::::::::::::::::::::::::::

@D0950.29_107155

GATGAACGCTGGCGGCGTGCTTAACACATGCAAGTCGAACGAAGCACTTTGAAGAGCTTGCTCTTTAAAGTGACTGAGTGGCGGACGGGTGAGTAACGCGTGGGTAACCTGCCTCATACAGGGGGATAACAGTTAGAAATGGACTGCTAACACCGCATAACCCGCTAGTGTCGCATGACACAGACGGAAAATATTTATAGGTATGAGATGGGCCCGCGTCTGATTAGCTAGTTGGTGGGGTAACGGCCTACCAAGGCAACGATCAGTAGCCGACTTGAGAGAGTGATCGGCCACATTGGGACTGAGACACGGCCCAAACTCCTACGGGAGGCAGCAGTGGGGAATATTGGACAATGGGGGAAACCCTGATCCAGCGACGCCGCGTGAGTGAAGAAGTATTTCGGTATGTAAAGCTCTATCAGCAGGGAAGATAATGACAGTACCTGACTAAGAAGCCCCGGCTAACTACGTGCCAGCAGCCGCGGTAA

+

::::::::::::::::::::::::::::::::::::::::::::::::::::::::::::::::::::::::::::::::::::::::::::::::::::::::::::::::::::::::::::::::::::::::::::::::::::::::::::::::::::::::::::::::::::::::::::::::::::::::::::::::::::::::::::::::::::::::::::::::::::::::::::::::::::::::::::::::::::::::::::::::::::::::::::::::::::::::::::::::::::::::::::::::::::::::::::::::::::::::::::::::::::::::::::::::::::::::::::::::::::::::::::::::::::::::::::::::::::::::::::::::::::::::::::::::::::::::::::::::::::::::

@D0950.29_107251

GATGAACGCTAGCGACAGGCCTAACACATGCAAGTCGAGGGGTAGCACAAGGAAGCTTGCTTCTGAGGTGACGACCGGCGCACGGGTGAGTAACGCGTATGCAACCTGCCTATAAGAAGGGGATAGCCTCTCGAAAGAGAGATTAATACCGTATAACACTATGAAACCGCATGGTTTTACAGTTAAAGATTTATTGCTTATAGATGGGCATGCGTAACATTAGCTAGTTGGTGAGGTAACGGCTCACCAAGGCAACGATGTTTAGGGGTTCTGAGAGGAAGGTCCCCCACACTGGTACTGAGACACGGACCAGACTCCTACGGGAGGCAGCAGTGAGGAATATTGCGCAATGGGGGCAAACCCTGACGCAGCAACGCCGCGTGAATGAAGAAGGCCTTAGGGTTGTAAAGTTCTG

+

:::::::::::::::::::::::::::::::::::::::::::::::::::::::::::::::::::::::::::::::::::::::::::::::::::::::::::::::::::::::::::::::::::::::::::::::::::::::::::::::::::::::::::::::::::::::::::::::::::::::::::::::::::::::::::::::::::::::::::::::::::::::::::::::::::::::::::::::::::::::::::::::::::::::::::::::::::::::::::::::::::::::::::::::::::::::::::::::::::::::::::::::::::::::::::::::::::::::::::::::::::::::::::::::

@D0950.29_107380

GATGAACGCTGGCGGCGTGCTTAACACATGCAAGTCGAACGAAGCACTTAACTTAGAATCTTCGGATGAAGAGTTTTGTGACTTAGTGGCGGACGGGTGAGTAACGCGTGGGTAACCTGCCTTATACTGGGGGATAACAGTTAGAAATGACTGCTAACACCGCATAACCCGCTAGTGTCGCATGACACAGACGGAAAATATTTATAGGTATGAGATGGGCCCGCGTCTGATTAGCTAGTTGGTGGGGTAAGCGGCCTACCAAGGCAACGATCAGTAGCCGACTTGAGAGAGTGATCGGCCACATTGGGACTGAGACACGGCCCAAACTCCTACGGGAGGCAGCAGTGGGGAATATTGGACAATGGGGGAAACCCTGATCCAGCGACGCCGCGTGAGTGAAGAAGTATTTCGGTATGTAAAGCTCTATCAGCAGGGAAGATAATGACAGTACCTGACT

+

:::::::::::::::::::::::::::::::::::::::::::::::::::::::::::::::::::::::::::::::::::::::::::::::::::::::::::::::::::::::::::::::::::::::::::::::::::::::::::::::::::::::::::::::::::::::::::::::::::::::::::::::::::::::::::::::::::::::::::::::::::::::::::::::::::::::::::::::::::::::::::::::::::::::::::::::::::::::::::::::::::::::::::::::::::::::::::::::::::::::::::::::::::::::::::::::::::::::::::::::::::::::::::::::::::::::::::::::::::::::::::::::::::::::::

@D0950.29_107381

GATGAACGCTAGCGACAGGCCTAACACATGCAAGTCGAGGGGTAGCACAAGGTAGCAATACTGAGGTGACGACCGGCGCACGGGTGAGTAACGCGTATGCAACCTACCTGTAAGAGTGGGATAGCCTCTCGAAAGAGAGATTAATACCGCATAATACCATTTCACTGCATGGTGAGATGGTTAAAGATTTATTGCTTACAGATGGGCATGCGTAACATTAGCTAGTTGGTGAGGTAACGGCTCACCAAGGCAACGATGTTTAGGGGTTCTGAGAGGAAGGTCCCCCGACACTGGTACTGAGACACGGACCAGACTCCTACGGGAGGCAGCAGTGAGGAATATTGGTCAATGGACGAGAGTCTGAACCAGCCAAGTCGCGTGAAGGATGAAGGTCTTATGGATTGTAAACTTCTTTTATACGGGAATAAAAATGCCACGTGTGGCATATTGCATGTACCGTATGAATAAGGATCGGCTAACTCCGTGCCAGCAGCCGCGGTAA

+

::::::::::::::::::::::::::::::::::::::::::::::::::::::::::::::::::::::::::::::::::::::::::::::::::::::::::::::::::::::::::::::::::::::::::::::::::::::::::::::::::::::::::::::::::::::::::::::::::::::::::::::::::::::::::::::::::::::::::::::::::::::::::::::::::::::::::::::::::::::::::::::::::::::::::::::::::::::::::::::::::::::::::::::::::::::::::::::::::::::::::::::::::::::::::::::::::::::::::::::::::::::::::::::::::::::::::::::::::::::::::::::::::::::::::::::::::::::::::::::::::::::::::::::::::::::

@D0950.29_107400

GATGAACGCTAGCTACAGGCTTAACACATGCAAGTCGAGGGGTAGCATGAAACTTAGCAATAAGTTTTGATGACGACCGGCGCACGGGTGAGTAACACGTATCCAACCTGCCTTTTACTCATGGATAGCCTTCTGAAAAGAAGATTAATACATGATGGTATTCAGAGTTTTCATGGACACTGAATTAAAGATTTTATCGGTAAAGAGATGGGGAATGCGTTCCATTAGATAGTAGGCTGGGGTAACGGCCCACCTAGTCGAACGATCGGATAGGGGTTCTGAGAGGAAGGTCCCCACATTGGAACTGAGACACGGTCCAAACGTCCTACGGGAGGCAGCAGTGAGGAATATTGGTCAATGGACGTAAGTCTGAACCAGCCAAGTAGCGTGAAGGATGAAGGCTCTA

+

::::::::::::::::::::::::::::::::::::::::::::::::::::::::::::::::::::::::::::::::::::::::::::::::::::::::::::::::::::::::::::::::::::::::::::::::::::::::::::::::::::::::::::::::::::::::::::::::::::::::::::::::::::::::::::::::::::::::::::::::::::::::::::::::::::::::::::::::::::::::::::::::::::::::::::::::::::::::::::::::::::::::::::::::::::::::::::::::::::::::::::::::::::::::::::::::::::::::::::::::::::::

@D0950.29_107484

GATGAACGCTGGCGGCGTGCTTAACACATGCAAGTCGAACGAAGCAACTTTCTTGCTTGCAAGAAAGTTGACTGAGTGGCGGACGGGTGAGTAACGCGTGGGTAACCTGCCTCATACAGGGGGATAACAGTTAGAAATGACTGCTAACACCGCATAACCCGCTAGTGTCGCATGACACGGACGGAAAATATTTATAGGTATGAGATGGGCCCGCGTCTGATTAGCTAGTTGGTGGGGTAACGGCCTACCAAGGCAACGATCAGTAGCCGACTTGAGAGAGTGATCGGCCACATTGGGACTGAGACACGGCCCAAACTCCTACGGGAGGCAGCAGTGGGGAATATTGGACAATGGGGGAAAGCCCTGATCCAGCCGACGCCGCGTGAGTGAAGAAGTATTTCGGTATGTAAAGCTCTATCAGCAGGGGAAGATAATGACAGTACCTGACTAAGAAGCCCCGGCTAACTACGTGCCAGCAGCCGCGGTAA

+

::::::::::::::::::::::::::::::::::::::::::::::::::::::::::::::::::::::::::::::::::::::::::::::::::::::::::::::::::::::::::::::::::::::::::::::::::::::::::::::::::::::::::::::::::::::::::::::::::::::::::::::::::::::::::::::::::::::::::::::::::::::::::::::::::::::::::::::::::::::::::::::::::::::::::::::::::::::::::::::::::::::::::::::::::::::::::::::::::::::::::::::::::::::::::::::::::::::::::::::::::::::::::::::::::::::::::::::::::::::::::::::::::::::::::::::::::::::::::::::::::::::::

@D0950.29_107539

GATGAACGCTGGCGGCGTGCTTAACACATGCAAGTCGAACGAAGCACTTTGAAGAGCTTGCTCTTTAAAGTGACTGAGTGGCGGACGGGTGAGTAACGCGTGGGTAACCTGCCTCATACAGGGGGATAACAGTTAGAAATGACTGCTAACACCGCATAACCCGCTAGTGTCGCATGACACAGACGGAAAATATTTATAGGTATGAGATGGGCCCGCGTCTGATTAGCTAGTTGGTGGGGTAACGGCCTACCAAGGCAACGATCAGTAGCCGACTTGAGAGAGTGATCGGCCACATTGGGACTGAGACACGGCCCAAACTCCTACGGGAGGCAGCAGTGGGGAATATTGGACAATGGGGGAAACCCTGATCCAGCGACGCCCGCGTGAGTGAAAGAAGTATTTTCGGTATGTAAAGCTCTATCAGCAGGGGAAAGATAATGACAGTACCTGACTAAGAAGCCCCGGCTAACTACGTGCCAGCAGCCGCGGTAA

+

::::::::::::::::::::::::::::::::::::::::::::::::::::::::::::::::::::::::::::::::::::::::::::::::::::::::::::::::::::::::::::::::::::::::::::::::::::::::::::::::::::::::::::::::::::::::::::::::::::::::::::::::::::::::::::::::::::::::::::::::::::::::::::::::::::::::::::::::::::::::::::::::::::::::::::::::::::::::::::::::::::::::::::::::::::::::::::::::::::::::::::::::::::::::::::::::::::::::::::::::::::::::::::::::::::::::::::::::::::::::::::::::::::::::::::::::::::::::::::::::::::::::::::

@D0950.29_107543

GATGAACGCTAGCGACAGGCCTAACACATGCAAGTCGAGGGGTAGCACAAGGTAGCAATACTGAGGTGACGACCGGCGCACGGGTGAGTAACGCGTATGCAACCTACCTGTAAGAGTGGGATAGCCTCTCGAAAGAGAGATTAATACCGCATAATACCATTTCACTGCATGGTGAGATGGTTAAAGATTTATTGCTTACAGATGGGCATGCGTAACATTAGCTTGTTGGTGAGGTAACGGCTCACCAAGGCAACGATGTTTAGGGGTTCTGAGAGGAAGGTCCCCCACACTGGTACTGAGACACGGACCAGACTCCTACGGGAGGCAGCAGTGAGGAATATTGGTCAATGGACGAGAGTCTGAACCAGCCAAGTCGCGTGAAGGATGAAGGTCTTATGGATTGTAACTTCTTTTATACGGGAATAAAAATGCCACGTGTGGCATATTGCATGTACCGTATGAATAAGGATCGGCTAACTCCGTGCCAGCAGCCGCGGTAA

+

::::::::::::::::::::::::::::::::::::::::::::::::::::::::::::::::::::::::::::::::::::::::::::::::::::::::::::::::::::::::::::::::::::::::::::::::::::::::::::::::::::::::::::::::::::::::::::::::::::::::::::::::::::::::::::::::::::::::::::::::::::::::::::::::::::::::::::::::::::::::::::::::::::::::::::::::::::::::::::::::::::::::::::::::::::::::::::::::::::::::::::::::::::::::::::::::::::::::::::::::::::::::::::::::::::::::::::::::::::::::::::::::::::::::::::::::::::::::::::::::::::::::::::::::::::

@D0950.29_107550

GATGAACGCTAGCTACAGGCTTAACACATGCAAGTCGAGGGGTAGCATGAAACTTAGCAATAAGTTTTGATGACGACCGGCGCACGGGTGAGTAACACGTATCCAACCTGCCTTTTACTCATGGATAGCCTTCTGAAAAGAAGATTAATACATGATGGTATTCAGAGTTTTCATGGACACTGAATTAAAGATTTATCGGTAAGAGATGGGGATGCGTTCCATTAGATAGTAGGCGGGGTAACGGCTCACCTAGGCGACGATCCCTAGCTGGTCTGAGAGGATGACCAGCCACACTGGAACTGAGACACGGTCCGAGACTCCTACGGGAGGCAGCAGTGGGGAATATTGCACAATGGGCGCAAGCCTGATGCAGCCATGCCGCGTGTATGAAGAAGGCCTTCGGGTTGTAAAGTACTTTCAGCGAGGAGGAAGGTGTTGAGGTTAATAACCTCAGCAATTGACGTTACTCGCAGAAGAAGCACCGGCTAACTCCGTGCCAGCAGCCG

+

::::::::::::::::::::::::::::::::::::::::::::::::::::::::::::::::::::::::::::::::::::::::::::::::::::::::::::::::::::::::::::::::::::::::::::::::::::::::::::::::::::::::::::::::::::::::::::::::::::::::::::::::::::::::::::::::::::::::::::::::::::::::::::::::::::::::::::::::::::::::::::::::::::::::::::::::::::::::::::::::::::::::::::::::::::::::::::::::::::::::::::::::::::::::::::::::::::::::::::::::::::::::::::::::::::::::::::::::::::::::::::::::::::::::::::::::::::::::::::::::::::::::::::::::::::::::::

@D0950.29_107738

GATGAACGCTAGCGACAGGCCTAACACATGCAAGTCGAGGGGTAGCACAAGGTAGCAATACTGAGGTGACGACCGGCGCACGGGTGAGTAACGCGTATGCAACCTACCTGTAAGAGTGGGATAGCCTCTCGAAAGAGAGATTAATACCGCATAATACCATTTCACTGCATGGTGAGATGGTTAAAGATTTATTGCTTACAGATGGGCATGCGTAACATTAGCTAGTTGGTGAGGTAACGGCTCACCAAGGCAACGATGTTTAGGGGTTCTGAGAGGAAGGTCCCCCACACTGGTACTGAGACACGGACCAGACTCCTACGGGAGGCAGCAGTGAGGAATATTGGTCAATGGACGAGAGTCTGAACCAGCCAAGTCGCGTGAAGGAAGAAGGTTCTATGGATTGTAAACTTCTTTTATAGGGGAATAAAGTGAGGAACGTGTTCCTTTTTGTATGTACCCTATGAATAAGCATCGGCTAACTCCGTGCCAGCAGCCGCGG

+

:::::::::::::::::::::::::::::::::::::::::::::::::::::::::::::::::::::::::::::::::::::::::::::::::::::::::::::::::::::::::::::::::::::::::::::::::::::::::::::::::::::::::::::::::::::::::::::::::::::::::::::::::::::::::::::::::::::::::::::::::::::::::::::::::::::::::::::::::::::::::::::::::::::::::::::::::::::::::::::::::::::::::::::::::::::::::::::::::::::::::::::::::::::::::::::::::::::::::::::::::::::::::::::::::::::::::::::::::::::::::::::::::::::::::::::::::::::::::::::::::::::::::::::::::::

@D0950.29_107759

GATGAACGCTAGCTACAGGCTTAACACATGCAAGTCGAGGGGTAGCATGAAACTTAGCAATAAGTTTTGATGACGACCGGCGCACGGGTGAGTAACACGTATCCAACCTGCCTTTTACTCATGGATAGCCTTCTGAAAAGAAGATTAATACATGATGGTATTCAGAGTTTTCATGGACACTGAATTAAAGATTTTATCGGTAAGAGATGGGGATGCGTTCCATTAGATAGTAGGCGGGGTAACGGCCCACCTAGTCAACGATGGATAGGGGTTCTGAGAGGAAGGTCCCCCACATTGGAACTGAGACACGGTCCAAACTCCTACGGGAGGCAGCAGTGAGGAATATTGGTCAATGGACGTAAGTCTGAACCAGCCAAGTAGCGTGAAGGATGAAGGCTCTATGGGTCGTAAACTTCTTTTTATAAAAGGAATAAAGTATGCCACGTGTGGTGTTTTTGTATGTACTTTATG

+

:::::::::::::::::::::::::::::::::::::::::::::::::::::::::::::::::::::::::::::::::::::::::::::::::::::::::::::::::::::::::::::::::::::::::::::::::::::::::::::::::::::::::::::::::::::::::::::::::::::::::::::::::::::::::::::::::::::::::::::::::::::::::::::::::::::::::::::::::::::::::::::::::::::::::::::::::::::::::::::::::::::::::::::::::::::::::::::::::::::::::::::::::::::::::::::::::::::::::::::::::::::::::::::::::::::::::::::::::::::::::::::::::::::::::::::::::::::::

@D0950.29_1078

GACGAACGCTGGCGGCGTGCCTAACACATGCAAGTCGAGCGATGAAGTTCTTCGGAACGGATTAGCGGCGGACGGGTGAGTAACACGTGGGTAACCTACCTGTAAGAGTGGGATAGCCTCTCGAAAGAGAGATTAATACCGCATAATACCATTTCACTGCATGGTGAGATGGTTAAAGATTTATTGCTTACAGATGGGCATGCGTAACATTAGCTAGTTGGTGAGGTAACGGCTCACCAAGGCAACGATGTTTAGGGGTTCTGAGAGGAAGGTCCCCACACTGGTACTGAGACACGGACCAGACTCCTACGGGAGGCAGCAGTGAGGAATATTGGTCAATGGACGAGAGTCTGAACCAGCCAAGTCGCGTGAAGGATGAAGGTCTTATGGATTGTAAACTTCTTTTATACGGGGAAATAAAAAACTGCCACGTGTGGCA

+

:::::::::::::::::::::::::::::::::::::::::::::::::::::::::::::::::::::::::::::::::::::::::::::::::::::::::::::::::::::::::::::::::::::::::::::::::::::::::::::::::::::::::::::::::::::::::::::::::::::::::::::::::::::::::::::::::::::::::::::::::::::::::::::::::::::::::::::::::::::::::::::::::::::::::::::::::::::::::::::::::::::::::::::::::::::::::::::::::::::::::::::::::::::::::::::::::::::::::::::::::::::::::::::::::::::::::::::::::::::::

@D0950.29_107820

GATGAACGCTAGCGACAGGCCTAACACATGCAAGTCGAGGGGTAGCACAAGGTAGCAATACTGAGGTGACGACCGGCGCACGGGTGAGTAACGCGTATGCAACCTACCTGTAAGAGTGGGATAGCCTCTCGAAAGAGAGATTAATACCGCATAATACCATTTCACTGCATGGTGAGATGGTTAAAGATTTATTGCTTACAGATGGGCATGCGTAACATTAGCTAGTTGGTGAGGTAACGGCTCACCAAGGCAACGATGTTTAGGGGTTCTGAGAGGAAGGTCCCCCCACACTGGTACTGAGACACGGACCAGACTCCTACGGGAGGCAGCAGTGAGGAAATATTGGTCAATGGACGAGAGTCTGAACCAGCCAAGTCGCGTGAAAGGATGAAAGGTCTTATGGATTGTAAAACTTCTTTTATACGGGAATAAAAAATGCCACGTGTGGCATATTGCATGTACCGTATGAATAAGGATCGGCTAAACTCCGTGCCAGCAGCCGCGGTAA

+

::::::::::::::::::::::::::::::::::::::::::::::::::::::::::::::::::::::::::::::::::::::::::::::::::::::::::::::::::::::::::::::::::::::::::::::::::::::::::::::::::::::::::::::::::::::::::::::::::::::::::::::::::::::::::::::::::::::::::::::::::::::::::::::::::::::::::::::::::::::::::::::::::::::::::::::::::::::::::::::::::::::::::::::::::::::::::::::::::::::::::::::::::::::::::::::::::::::::::::::::::::::::::::::::::::::::::::::::::::::::::::::::::::::::::::::::::::::::::::::::::::::::::::::::::::::::::::

@D0950.29_10784

GATGAACGCTGGCGGCGTGCTTAACACATGCAAGTCGAACGAAGCACTTTGAAGAGCTTGCTCTTTAAAGTGACTGAGTGGCGGACGGGTGAGTAACGCGTGGGTAACCTGCCTCATACAGGGGGATAACAGTTAGAAATGACTGCTAACACCGCATAACCCGCTAGTGTCGCATGACACGGACGGAAAATATTTATAGGTATGAGATGGGCCCGCGTCTGATTAGCTAGTTGGTGGGGTAAAGGCCTACCAAGGCAACGATCAGTAGCCGACTTGAGAGAGTGATCGGCCACATTGGGACTGAGACACGGCCCAAACTCCTACGGGAGGCAGCAGTGGGGAATATTGGACAATGGGGCAACCCTGATCCAGCGACGCCGCGTGAGTGAAGAAGTATTTCGGTATGTAAAGCTCTATCAGCAGGGAAGATAATGACAGTACCTGACTAAGAAGCCCCGGCTAACTACGTGCCAGCAGCCGCGGTAA

+

::::::::::::::::::::::::::::::::::::::::::::::::::::::::::::::::::::::::::::::::::::::::::::::::::::::::::::::::::::::::::::::::::::::::::::::::::::::::::::::::::::::::::::::::::::::::::::::::::::::::::::::::::::::::::::::::::::::::::::::::::::::::::::::::::::::::::::::::::::::::::::::::::::::::::::::::::::::::::::::::::::::::::::::::::::::::::::::::::::::::::::::::::::::::::::::::::::::::::::::::::::::::::::::::::::::::::::::::::::::::::::::::::::::::::::::::::::::::::::::::::::::

@D0950.29_107947

GATGAACGCTAGCTACAGGCTTAACACATGCAAGTCGAGGGGTAGCATGAAACTTAGCAATAAGTTTTGATGACGACCGGCGCACGGGTGAGTAACACGTATCCAACCTGCCTTTTACTCATGGATAGCCTTCTGAAAAGAAGATTAATACATGATGGTATTCAGAGTTTTCATGGACACTGAATTAAAGATTTATCGGTAAGAGATGGGGATGCGTTCCATTAGATAGTAGGCGGGGTAACGGCCCACCTAGTCAACGATGGATAGGGGTTCTGAGAGGAAGGTCCCCCACATTGGAACTGAGACACGGTCCAAACGTCCTACGGGAGGCAGCAGTGAGGAATATTGGTCAATGGACGTAAGTCTGAACCAGCCAAGTAGCGTGAAGGATGAAGGCTCTATGGGTCGTAAACTTCTTTTTATAAAAGGAATAAAGTATGCCACGTGTGGTGTTTTGTATGTACTTTATGAATAAGGATCGGCTAACTCCGTGCCAGCAGCCGC

+

::::::::::::::::::::::::::::::::::::::::::::::::::::::::::::::::::::::::::::::::::::::::::::::::::::::::::::::::::::::::::::::::::::::::::::::::::::::::::::::::::::::::::::::::::::::::::::::::::::::::::::::::::::::::::::::::::::::::::::::::::::::::::::::::::::::::::::::::::::::::::::::::::::::::::::::::::::::::::::::::::::::::::::::::::::::::::::::::::::::::::::::::::::::::::::::::::::::::::::::::::::::::::::::::::::::::::::::::::::::::::::::::::::::::::::::::::::::::::::::::::::::::::::::::::::::::

@D0950.29_108048

GATGAACGCTAGCGACAGGTCTAACACATGCAAGTCGAGGGGTAGCACAAGGAAGCTTGCTTCTGAGGTGACGACCGGCGCACGGGTGAGTAACGCGTATGCAACCTACCTGTAAGAGTGGGATAGCCTCTCGAAAGAGAGATTAATACCGCATAATACCATTTTACTGCATGGTGAGATGGTTAAAGATTTATTGCTTACAGATGGGCATGCGTAACATTAGCTAGTTGGTGAGGTAACGGCTCACCAAGGCAACGATGTTTAGGGGTTCTGAGAGGAAGGTCCCCCACACTGGTACTGAGACACGGACCAGACTCCTACGGGAGGCAGCAGTGAGGAATATTGGTCAATGGACGAGAGTCTGAACCAGCCAAGTCGCGTGAAGGATGAAGGTCTTATGGATTGTAAACTTCTTTTATACGGGAATAAAAATGCCACGTGTGGCATATTGCATGTACCGTATGAATAAGGATCGGCTAACTCCGTGCCAGCAGCCGCGGTAA

+

:::::::::::::::::::::::::::::::::::::::::::::::::::::::::::::::::::::::::::::::::::::::::::::::::::::::::::::::::::::::::::::::::::::::::::::::::::::::::::::::::::::::::::::::::::::::::::::::::::::::::::::::::::::::::::::::::::::::::::::::::::::::::::::::::::::::::::::::::::::::::::::::::::::::::::::::::::::::::::::::::::::::::::::::::::::::::::::::::::::::::::::::::::::::::::::::::::::::::::::::::::::::::::::::::::::::::::::::::::::::::::::::::::::::::::::::::::::::::::::::::::::::::::::::::::::::

@D0950.29_10805

GATGAACGCTAGCGACAGGCCTAACACATGCAAGTCGAGGGGTAGCACAAGGAAGCTTGCTTCTGAGGTGACGACCGGCGCACGGGTGAGTAACGCGTATGCAACCTGCCTATAAGAAGGGGATAGCCTCTCGAAAGAGAGATTAATACCGTATAACACTATGAAGCCGCATGGTTTTATAGTTAAAGATTTATTGCTTATAGATGGGCATGCGTAACATTAGCTAGTTGGTGAGGTAACGGCTCACCAAGGCAACGATGGATAGGGGTTCTGAGAGGAAGGTCCCCCACATTGGAACTGAGACACGGTCCAAACTCCTACGGGAGGCAGCAGTGAGGAATATTGGTCAATGGACGTAAGTCTGAACCAGCCAAGTAGCGTGAAGGATGAAGGCTCTATGGGTCGTAAACTTCTTTTATAAAGGAATAAAGTATGCCACGTGTGGTGTTTTGTATGTACTTTTATGAATAAGGGTTCGGCTAACTCCGTGCCAGCAGCCGCGGTAA

+

::::::::::::::::::::::::::::::::::::::::::::::::::::::::::::::::::::::::::::::::::::::::::::::::::::::::::::::::::::::::::::::::::::::::::::::::::::::::::::::::::::::::::::::::::::::::::::::::::::::::::::::::::::::::::::::::::::::::::::::::::::::::::::::::::::::::::::::::::::::::::::::::::::::::::::::::::::::::::::::::::::::::::::::::::::::::::::::::::::::::::::::::::::::::::::::::::::::::::::::::::::::::::::::::::::::::::::::::::::::::::::::::::::::::::::::::::::::::::::::::::::::::::::::::::::::::::

@D0950.29_108065

GATGAACGCTAGCGACAGGCCTAACACATGCAAGTCGAGGGGTAGCACAAGGTAGCAATACTGAGGTGACGACCGGCGCACGGGTGAGTAACGCGTATGCAACCTACCTGTAAGAGTGGGATAGCCTCTCGAAAGAGAGATTAATACCGCATAATACCATTTTACTGCATGGTGAGATGGTTAAAGATTTATTGCTTACAGATGGGCATGCGTAACATTAGCTAGTTGGTGAGGTAACGGCTCACCAAGGCAACGATGTTTAGGGGTTCTGAGAGGAAGGTCCCCCACACTGGTACTGAGACACGGGACCAGACTCCTACGGGAGGCAGCAGTGAGGAATATTGGTCAATGGACGAGAGTCTGAACCAGCCAAGTCGCGTGAAGGATGAAGGTCTTATGGATTGTAAACTTCTTTTATACGGGAATAAAAATGCCACGTGTGGCATATTGCATGTACCGTATGAATAAGGATCGGCTAACTCCGTGCCAGCAGCCGCGGTAA

+

::::::::::::::::::::::::::::::::::::::::::::::::::::::::::::::::::::::::::::::::::::::::::::::::::::::::::::::::::::::::::::::::::::::::::::::::::::::::::::::::::::::::::::::::::::::::::::::::::::::::::::::::::::::::::::::::::::::::::::::::::::::::::::::::::::::::::::::::::::::::::::::::::::::::::::::::::::::::::::::::::::::::::::::::::::::::::::::::::::::::::::::::::::::::::::::::::::::::::::::::::::::::::::::::::::::::::::::::::::::::::::::::::::::::::::::::::::::::::::::::::::::::::::::::::::::

@D0950.29_108069

GATGAACGCTGGCGGCGTGCTTAACACATGCAAGTCGAACGAAGCACTTTGAAGAGCTTGCTCTTTAAAGTGACTGAGTGGCGGACGGGTGAGTAACGCGTGGGTAACCTGCCTCATACAGGGGGATAACAGTTAGAAATGACTGCTAACACCGCATAACCCGCTAGTGTCGCATGACACAGACGGAAAATATTTATAGGTATGAGATGGGCCCGCGTCTGATTAGCTAGTTGGTGGGGTAACGGCCTACCAAGGCAACGATCAGTAGCCGACTTGAGAGAGTGATCGGCCACATTGGGACTGAGACACGGCCCAAACTCCTACGGGAGGCAGCAGTGGGGAATATTGGACAATGGGGGAAACCCTGATCCAGCGACGCCGCGTGAGTGAAGAAGTATTTCGGTATGTAAAGCTCTATCAGCAGGGGAAGATAATGACAGTACCTGACTAAGAAGCCCCGGCTAACTACGTGCCAGCAGCCGCGGTAA

+

::::::::::::::::::::::::::::::::::::::::::::::::::::::::::::::::::::::::::::::::::::::::::::::::::::::::::::::::::::::::::::::::::::::::::::::::::::::::::::::::::::::::::::::::::::::::::::::::::::::::::::::::::::::::::::::::::::::::::::::::::::::::::::::::::::::::::::::::::::::::::::::::::::::::::::::::::::::::::::::::::::::::::::::::::::::::::::::::::::::::::::::::::::::::::::::::::::::::::::::::::::::::::::::::::::::::::::::::::::::::::::::::::::::::::::::::::::::::::::::::::::::::

@D0950.29_108115

GACGAACGCTGGCGGCGCGCCTAACACATGCAAGTCGAACGGAGTTATTTTGACAGATTCTTTCGGGATGAAGATAAATTAACTTAGTGGCGGACGGGTGAGTAACACGTGAGCAACCTGCCTTACAGAGGGGGATAACGTTTGGAAACGAACGCTAATACCGCATAACATTATTAAATCGCATGATTTGATAATCAAAGGAGAAATCCGCTGAAAGATGGGCTCGCGTCTGATTAGATAGTTGGTGAGGTAATGGCTCACCAAGTCGACGATCAGTAGCCGGACTGAGAGGTTGAACGGCCACATTGGGACTGAGACACGGCCCAGACTCCTACGGGAGGCAGCAGTGGGGAATATTGCACAATGGGGGAAACCCTGATGCAGCGACGCCGCGTGAGGGAAGAAGGTTTTCGGATTGTAAACCTCTGTCTTCAGGGACGATAATGACGGTACCTGAGGAGGAAGCCACGGCTAACTACGTGCCAGCAGCCGCGGTAA

+

::::::::::::::::::::::::::::::::::::::::::::::::::::::::::::::::::::::::::::::::::::::::::::::::::::::::::::::::::::::::::::::::::::::::::::::::::::::::::::::::::::::::::::::::::::::::::::::::::::::::::::::::::::::::::::::::::::::::::::::::::::::::::::::::::::::::::::::::::::::::::::::::::::::::::::::::::::::::::::::::::::::::::::::::::::::::::::::::::::::::::::::::::::::::::::::::::::::::::::::::::::::::::::::::::::::::::::::::::::::::::::::::::::::::::::::::::::::::::::::::::::::::::::::::::

@D0950.29_108164

GATGAACGCTGGCGGCGTGCTTAACACATGCAAGTCGAACGAAGCACTTTGAAGAGCTTGCTCTTTAAAGTGACTGAGTGGCGGACGGGTGAGTAACGCGTGGGTAACCTGCCTCATACAGGGGGATAACAGGTTAGAAATGACTGCTAACACCGCATAACCCGCTAGCATCGCATGATGCAGACGGAAAATATTTATAGGTATGAGATGGGCCCGCGTCTGATTAGCTAGTTGGTGGGGTAACGGCCTACCAAGGCAACGATCAGTAGCCGACTTGAGAGAGTGATCGGCCACATTGGGACTGAGACACGGGCCCAAACTCCTACGGGAGGCAGCAGTGGGGAATATTGGACAATGGGGGAAACCCTGATCCAGCGACGCCGCGTGAGTGAAGAAGTATTTTCGGTATGTAAAGCTCTATCAGCAGGGGAAGATGATGACAGGTACCTGACTAAGAAGCCCCGGCTAACTACGTGCCAGCAGCCGCGGTAA

+

::::::::::::::::::::::::::::::::::::::::::::::::::::::::::::::::::::::::::::::::::::::::::::::::::::::::::::::::::::::::::::::::::::::::::::::::::::::::::::::::::::::::::::::::::::::::::::::::::::::::::::::::::::::::::::::::::::::::::::::::::::::::::::::::::::::::::::::::::::::::::::::::::::::::::::::::::::::::::::::::::::::::::::::::::::::::::::::::::::::::::::::::::::::::::::::::::::::::::::::::::::::::::::::::::::::::::::::::::::::::::::::::::::::::::::::::::::::::::::::::::::::::::::

@D0950.29_1082

GATGAACGCTAGCGACAGGCCTAACACATGCAAGTCGAGGGGTAGCACAAGGAAGCTTGCTTCTGAGGTGACGACCGGCGCACGGGTGAGTAACGCGTATGCAACCTGCCTATAAGAAGGGGATAGCCTCTCGAAAGAGAGATTAATACCGTATAACACTATGAAGCCGCATGGTTTTACAGTTAAAGATTTATTGCTTATAGATGGGCATGCGTAACATTAGCTAGTTGGTGAGGTAACGGCTCACCAAGGCAACGATGTTTAGGGGTTCTGAGAGGAAGGTCCCCCACACTGGTACTGAGACACGGACCAGACTCCTACGGGAGGCAGCAGTGAGGAATATTGGTCAATGGACGAGAGTCTGAACCAGCCAAGTCGCGTGAAGGATGAAGGTCTTATGGATTGTAAACTTCTTTTATACGGGAATAAAAACGAGCCACGTGTGGCTTATTGCATGTACCGTATGAATAAGGATCGGCTAACTCCGTGCCAGCAGCCGC

+

::::::::::::::::::::::::::::::::::::::::::::::::::::::::::::::::::::::::::::::::::::::::::::::::::::::::::::::::::::::::::::::::::::::::::::::::::::::::::::::::::::::::::::::::::::::::::::::::::::::::::::::::::::::::::::::::::::::::::::::::::::::::::::::::::::::::::::::::::::::::::::::::::::::::::::::::::::::::::::::::::::::::::::::::::::::::::::::::::::::::::::::::::::::::::::::::::::::::::::::::::::::::::::::::::::::::::::::::::::::::::::::::::::::::::::::::::::::::::::::::::::::::::::::::::::

@D0950.29_108260

GATGAACGCTAGCGACAGGCCTAACACATGCAAGTCGAGGGGTAGCACAAGGAAGCTTGCTTCTGAGGTGACGACCGGCGCACGGGTGAGTAACGCGTATGCAACCTGCCTATAAGAAGGGGATAGCCTCTCGAAAGAGAGATTAATACCGTATAACACTATGAGATCGCATGGTTTTATAGTTAAAGATTTATTGCTTATAGATGGGCATGCGTAACATTAGCTTGTTGGTGAGGTAACGGCTCACCAAGGCAACGATGTTTAGGGGTTCTGAGAGGAAGGTCCCCCACACTGGTACTGAGACACGGACCAGACTCCTACGGGAGGCAGCAGTGAGGAATATTGGTCAATGGACGAGAGTCTGAACCAGCCAAGTCGCGTGAAGGATGAAGGTCTTATGGATTGTAAACTTCTTTTATACGGGGAATAAAAAATACCACGTGTGGTATATTGCATGTACCGTATGAATAAGGATCGGCTAACTCCGTGCCAGCAGCCGC

+

::::::::::::::::::::::::::::::::::::::::::::::::::::::::::::::::::::::::::::::::::::::::::::::::::::::::::::::::::::::::::::::::::::::::::::::::::::::::::::::::::::::::::::::::::::::::::::::::::::::::::::::::::::::::::::::::::::::::::::::::::::::::::::::::::::::::::::::::::::::::::::::::::::::::::::::::::::::::::::::::::::::::::::::::::::::::::::::::::::::::::::::::::::::::::::::::::::::::::::::::::::::::::::::::::::::::::::::::::::::::::::::::::::::::::::::::::::::::::::::::::::::::::::::::::::

@D0950.29_108292

ATTGAACGCTGGCGGCAGGCCTAACACATGCAAGTCGAACGGTAGCACAGAGGAGCTTGCTCCTTGGGTGACGAGTGGCGGACGGGTGAGTAATGTCTGGGAAACTGCCCGATGGAGGGGGATAACTACTGGAAACGGTAGCTAATACCGCATAACGTCGCAAGACCAAAGAGGGGGACCTTCGGGCCTCTTGCCATCGGATGTGCCCAGATGGGATTAGCTAGTAGGTGGGGTAACGGCTCACCTAGGCGACGATCCCTAGCTGGTCTGAGAGGATGACCAGCCACACTGGAACTGAGACACGGTCCAGACTCCTACGGGAGGCAGCAGTGGGGAATATTGCACAATGGGGGAAACCCTGATGCAGCGACGCCGCGTGAAGGATGAAGTATTTCGGTATGTAAACTTCTATCAGCAGGGAAGAAAATGACGGTACCTGACTAAGAAGCCCCGGCTAACTACGTGCCAGCAGCCGCGGTAA

+

:::::::::::::::::::::::::::::::::::::::::::::::::::::::::::::::::::::::::::::::::::::::::::::::::::::::::::::::::::::::::::::::::::::::::::::::::::::::::::::::::::::::::::::::::::::::::::::::::::::::::::::::::::::::::::::::::::::::::::::::::::::::::::::::::::::::::::::::::::::::::::::::::::::::::::::::::::::::::::::::::::::::::::::::::::::::::::::::::::::::::::::::::::::::::::::::::::::::::::::::::::::::::::::::::::::::::::::::::::::::::::::::::::::::::::::::::::::::::::::::::

@D0950.29_108323

GATGAACGCTAGCTACAGGCTTAACACATGCAAGTCGAGGGGTAGCATGAAACTTAGCAATAAGTTTTGATGACGACCGGCGCACGGGTGAGTAACACGTATCCAACCTGCCTTTTACTCATGGATAGCCTTCTGAAAAGAAGATTAATACATGATGGTATTCAGAGTTTTCATGGACACTGAATTAAAGATTTTATCGGTAAGAGATGGGGATGCGTTCCATTAGATAGTAGGCGGGGTAACGGCCCACCTAGTCAACGATGGATAGGGGTTCTGAGAGGAAGGTCCCCCACATTGGAACTGAGACACGGTCCAAACTCCTACGGGAGGCAGCAGTGAGGAATATTGGTCAATGGACGTAAGTCTGAACCAGCCAAGTAGCGTGAAGGATGAAGGCTCTATGGGTCGTAAACTTCTTTTTATAAAAGGAATAAAGTATGCCACGTGTGGTGTTTTTGTATGTACTTTATG

+

:::::::::::::::::::::::::::::::::::::::::::::::::::::::::::::::::::::::::::::::::::::::::::::::::::::::::::::::::::::::::::::::::::::::::::::::::::::::::::::::::::::::::::::::::::::::::::::::::::::::::::::::::::::::::::::::::::::::::::::::::::::::::::::::::::::::::::::::::::::::::::::::::::::::::::::::::::::::::::::::::::::::::::::::::::::::::::::::::::::::::::::::::::::::::::::::::::::::::::::::::::::::::::::::::::::::::::::::::::::::::::::::::::::::::::::::::::::::

@D0950.29_108335

GATGAACGCTAGCTACAGGCTTAACACATGCAAGTCGAGGGGTAGCATGAAACTTAGCAATAAGTTTTGATGACGACCGGCGCACGGGTGAGTAACACGTATCCAACCTGCCTTTTACTCATGGATAGCCTTCTGAAAAGAAGATTAATACATGATGGTATTCAGAGTTTTCATGGACACTGAATTAAAGATTTTATCGGTAAGAGATGGGGATGCGTTCCATTAGATAGTAGGCTGGGGTAACGGCCCACCTAGTCGAACCGATCGGATAGGGGTTCTGAGAGGAAGGTCCCCCACATTGGAACTGAGACACGGTCCAAACGTCCGTACGGGAGGCAGCAGTGAGGGAATATTGGTCAATGGACGTAAGTCTGAACCAGCCAAGTAGCGTGAAGGATGAAGGCTC

+

::::::::::::::::::::::::::::::::::::::::::::::::::::::::::::::::::::::::::::::::::::::::::::::::::::::::::::::::::::::::::::::::::::::::::::::::::::::::::::::::::::::::::::::::::::::::::::::::::::::::::::::::::::::::::::::::::::::::::::::::::::::::::::::::::::::::::::::::::::::::::::::::::::::::::::::::::::::::::::::::::::::::::::::::::::::::::::::::::::::::::::::::::::::::::::::::::::::::::::::::::::::

@D0950.29_10834

GACGAACGCTGGCGGCGTGCCTAACACATGCAAGTCGAGCGATGAAGTTCTTCGGAACGGATTAGCGGCGGACGGGTGAGTAACACGTGGGTAACCTGCCTTGTAGAGGGGGATAGCCTTCCGAAAGGAAGATTAATACCGCATAACATCTTTTTATCGCATGGTAGAAAGATCAAAGGAGCAATCCGCTACAAGATGGACCCGCGGCGCATTAGCTAGTTGGTGAGGTAACGGCTCACCAAGGCGACGATGCGTAGCCGACCTGAGAGGGTGATCGGCCACATTGGAACTGAGACACGGTCCAGACTCCTACGGGAGGCAGCAGTGGGGAATATTGCGCAATGGGGGAAACCCTGACGCAGCAACGCCGCGTGAATGAAGAAGGCCTTAGGGTTGTAAAGTTCTGTTTACGGGGACGATAATGACGGTACCCGTGGAGGAAGCCACGGCTAACTACGTGCCAGCAGCCGCGGTAA

+

::::::::::::::::::::::::::::::::::::::::::::::::::::::::::::::::::::::::::::::::::::::::::::::::::::::::::::::::::::::::::::::::::::::::::::::::::::::::::::::::::::::::::::::::::::::::::::::::::::::::::::::::::::::::::::::::::::::::::::::::::::::::::::::::::::::::::::::::::::::::::::::::::::::::::::::::::::::::::::::::::::::::::::::::::::::::::::::::::::::::::::::::::::::::::::::::::::::::::::::::::::::::::::::::::::::::::::::::::::::::::::::::::::::::::::::::::::::::::::

@D0950.29_108342

GACGAACGCTAGCGACAGGCCTAACACATGCAAGTCGAGGGGGTAGCACAAGGAAGCTTGCTTCTGAGGTGACGACCGGCGCACGGGTGAGTAACGCGTATGCAACCTGCCTATAAGAAGGGGATAGCCTCTCGAAAGAGAGATTAATACCGTATAACACTATGAAGCCGCATGGTTTTATGGTTAAAGATTTATTGCTTATAGATGGGCATGCGTAACATTAGCTAGTTGGTGAGGTAACGGCTCACCAAGGCAACGATGTTTAGGGGTTCTGAGAGGAAGGTCCCCCACACTGGTACTGAGACAGGGGACCAGACTCCTACGGGAGGCAGCAGTGAGGGAATATTGGTCAATGGACGAGAGTCTGAACCAGCCAAGTCGCGTGAAGGATGAAGGTCTTATGGATTGTAAACTTCTTTTATACGGGGAAATAAAAAACG

+

::::::::::::::::::::::::::::::::::::::::::::::::::::::::::::::::::::::::::::::::::::::::::::::::::::::::::::::::::::::::::::::::::::::::::::::::::::::::::::::::::::::::::::::::::::::::::::::::::::::::::::::::::::::::::::::::::::::::::::::::::::::::::::::::::::::::::::::::::::::::::::::::::::::::::::::::::::::::::::::::::::::::::::::::::::::::::::::::::::::::::::::::::::::::::::::::::::::::::::::::::::::::::::::::::::::::::::::::::::::::

@D0950.29_108347

GATGAACGCTAGCTACAGGCTTAACACATGCAAGTCGAGGGGTAGCATGAAACTTAGCAATAAGTTTTGATGACGACCGGCGCACGGGTGAGTAACACGTATCCAACCTGCCTTTTACTCATGGATAGCCTTCTGAAAAGAAGATTAATACATGATGGTATTCAGAGTTTTCATGGACACTGAATTAAAGATTTTATCGGTAAGAGATGGGGATGCGTTCCATTAGATAGTAGGCGGGGTAACGGCCCACCTAGTCAACGATGGATAGGGGTTCTGAGAGGAAGGTCCCCACATTGGAACTGAGACACGGTCCAAACGTCCTACGGGAGGCAGCAGTGAGGAATATTGGTCAATGGACGTAAGTCTGAACCAGCCAAGTAGCGTGAAGGATGAAGGCTCTATGGGTCGTAAACTTCTTTTTATAAAAGG

+

:::::::::::::::::::::::::::::::::::::::::::::::::::::::::::::::::::::::::::::::::::::::::::::::::::::::::::::::::::::::::::::::::::::::::::::::::::::::::::::::::::::::::::::::::::::::::::::::::::::::::::::::::::::::::::::::::::::::::::::::::::::::::::::::::::::::::::::::::::::::::::::::::::::::::::::::::::::::::::::::::::::::::::::::::::::::::::::::::::::::::::::::::::::::::::::::::::::::::::::::::::::::::::::::::::::::::::::

@D0950.29_108390

GACGAACGCTGGCGGCGTGCCTAACACATGCAAGTCGAGCGATGAAGTTCTTCGGAACGGATTAGCGGCGGACGGGTGAGTAACACGTGGGTAACCTGCCTTGTAGAGGGGGATAGCCTTCCGAAAGGAAGATTAATACCGCATAACATCTTTTTATCGCATGGTAGAAAGATCAAAGGAGTAATCCGCTACAAGATGGACCCGCGGCGCATTAGCTAGTTGGTGAGGTAACGGCTCACCAAGGCGACGATGCGTAGCCGACCTGAGAGGGTGATCGGCCACATTGGAACTGAGACACGGTCCAGACTCCTACGGGAGGCAGCAGTGGGGAATATTGCACAATGGGGGAAACCCTGATGCAGCAACGCCGCGTGAGTGATGAAGGCCTTCGGGTTGTAAAGCTCTGTCTTCAGGGACGATAATGACGGTACCTGAGGAGGAAGCCACGGCTAACTACGTGCCAGCAGCCGCGGTAA

+

::::::::::::::::::::::::::::::::::::::::::::::::::::::::::::::::::::::::::::::::::::::::::::::::::::::::::::::::::::::::::::::::::::::::::::::::::::::::::::::::::::::::::::::::::::::::::::::::::::::::::::::::::::::::::::::::::::::::::::::::::::::::::::::::::::::::::::::::::::::::::::::::::::::::::::::::::::::::::::::::::::::::::::::::::::::::::::::::::::::::::::::::::::::::::::::::::::::::::::::::::::::::::::::::::::::::::::::::::::::::::::::::::::::::::::::::::::::::::::

@D0950.29_108452

GATGAACGCTAGCTACAGGCTTAACACATGCAAGTCGAGGGGTAGCATGAAACTTAGCAATAAGTTTTGATGACGACCGGCGCACGGGTGAGTAACACGTATCCAACCTGCCTTTTACTCATGGATAGCCTTCTGAAAAGAAGATTAATACATGATGGTATTCAGAGTTTTCATGGACACTGAATTAAAGATTTATCGGTAAGAGATGGGGATGCGTTCCATTAGATAGTAGGCGGGGTAACGGCCCACCTAGTCAACGATGGATAGGGGTTCTGAGAGGAAGGTCCCCCAGCATTGGAACTGAGACACGGTCCAAACTCCTACGGGAGGCAGCAGTGAGGAATATTGGACAATGGGGGAAACCCTGATCCAGCGACGCCGCGTGAGTGAAGAAGTATTTCGGTATGTAAAGCTCTATCAGCAGGGAAGATAATGACAGTACCTGACTAAGAAGCCCCGGCTAACTACGTGCCAGCAGCCGCGGTAA

+

:::::::::::::::::::::::::::::::::::::::::::::::::::::::::::::::::::::::::::::::::::::::::::::::::::::::::::::::::::::::::::::::::::::::::::::::::::::::::::::::::::::::::::::::::::::::::::::::::::::::::::::::::::::::::::::::::::::::::::::::::::::::::::::::::::::::::::::::::::::::::::::::::::::::::::::::::::::::::::::::::::::::::::::::::::::::::::::::::::::::::::::::::::::::::::::::::::::::::::::::::::::::::::::::::::::::::::::::::::::::::::::::::::::::::::::::::::::::::::::::::::::::

@D0950.29_108466

GATGAACGCTAGCTACAGGCTTAACACATGCAAGTCGAGGGGTAGCATGAAACTTAGCAATAAGTTTTGATGACGACCGGCGCACGGGTGAGTAACACGTATCCAACCTGCCTTTTACTCATGGATAGCCTTCTGAAAAGAAGATTAATACATGATGGTATTCAGAGTTTTCATGGACACTGAATTAAAGATTTATCGGTAAGAGATGGGGATGCGTTCCATTAGATAGTAGGCGGGGTAACGGCCCACCTAGTCAACGATGGATAGGGGTTCTGAGAGGAAGGTCCCCACATTGGAACTGAGACACGGTCCAAACTCCTACGGGAGGCAGCAGTGAGGAATATTGGTCAATGGACGTAAGTCTGAACCAGCCAAGTAGCGTGAAGGATGAAGGCTCTATGGGTCGTAAACTTCTTTTTATAAAAGGAATAAAGTATGCCACGTGTGGTGTTTTTGTATGTACTTTATGAATAAGGAT

+

::::::::::::::::::::::::::::::::::::::::::::::::::::::::::::::::::::::::::::::::::::::::::::::::::::::::::::::::::::::::::::::::::::::::::::::::::::::::::::::::::::::::::::::::::::::::::::::::::::::::::::::::::::::::::::::::::::::::::::::::::::::::::::::::::::::::::::::::::::::::::::::::::::::::::::::::::::::::::::::::::::::::::::::::::::::::::::::::::::::::::::::::::::::::::::::::::::::::::::::::::::::::::::::::::::::::::::::::::::::::::::::::::::::::::::::::::::::::::::::

@D0950.29_108475

GATGAACGCTAGCTACAGGCTTAACACATGCAAGTCGAGGGGTAGCATGAAACTTAGCAATAAGTTTTGATGACGACCGGCGCACGGGTGAGTAACACGTATCCAACCTGCCTTTTACTCATGGATAGCCTTCTGAAAAGAAGATTAATACATGATGGTATTCAGAGTTTTCATGGACACTGAATTAAAGATTTTATCGGTAAGAGATGGGGATGCGTTCCATTAGATAGTAGGCGGGGTAACGGCCCACCTAGTCAACGATGGATAGGGGTTCTGAGAGGAAGGTCCCCCACATTGGAACTGAGACACGGTCCAAACTCCTACGGGAGGCAGCAGTGAGGAATATTGGTCAATGGACGTAAGTCTGAACCAGCCAAGTAGCGTGAAGGATGAAGGCTCTATGGGTCGTAAACTTCTTTTTATAAAAGGAATAAAGTATGCCACGTGTGGTGTTTTTGTATGTACTTTATGAATAAGGATCGGCTAACTCCGTGCCAGCAGCCGC

+

:::::::::::::::::::::::::::::::::::::::::::::::::::::::::::::::::::::::::::::::::::::::::::::::::::::::::::::::::::::::::::::::::::::::::::::::::::::::::::::::::::::::::::::::::::::::::::::::::::::::::::::::::::::::::::::::::::::::::::::::::::::::::::::::::::::::::::::::::::::::::::::::::::::::::::::::::::::::::::::::::::::::::::::::::::::::::::::::::::::::::::::::::::::::::::::::::::::::::::::::::::::::::::::::::::::::::::::::::::::::::::::::::::::::::::::::::::::::::::::::::::::::::::::::::::::::::

@D0950.29_108534

GATGAACGCTAGCTACAGGCTTAACACATGCAAGTCGAGGGGTAGCATGAAACTTAGCAATAAGTTTTGATGACGACCGGCGCACGGGTGAGTAACACGTATCCAACCTGCCTTTTACTCATGGATAGCCTTCTGAAAAGAAGATTAATACATGATGGTATTCAGAGTTTTCATGGACACTGAATTAAAGATTTTATCGGTAAGAGATGGGGATGCGTTCCATTAGATAGTAGGCGGGGTAACGGCCCACCTAGTCAACGATGGATAGGGGTTCTGAGAGGAAGGTCCCCCACATTGGAACTGAGACACGGTCCAAACTCCGTACGGGAGGCAGCAGTGAGGAATATTGGTCAATGGACGTAAGTCTGAACCAGCCAAGTAGCGTGAAGGATGAAGGCTCTATGGGTCGTAAACTTCTTTTTATAAAAGGAATAAAGTATGCCACGTGTGGTGTTTTTGTATGTACTTTATGAATAAGGATCGGCTAACTCCGTGCCAGCAGCCG

+

:::::::::::::::::::::::::::::::::::::::::::::::::::::::::::::::::::::::::::::::::::::::::::::::::::::::::::::::::::::::::::::::::::::::::::::::::::::::::::::::::::::::::::::::::::::::::::::::::::::::::::::::::::::::::::::::::::::::::::::::::::::::::::::::::::::::::::::::::::::::::::::::::::::::::::::::::::::::::::::::::::::::::::::::::::::::::::::::::::::::::::::::::::::::::::::::::::::::::::::::::::::::::::::::::::::::::::::::::::::::::::::::::::::::::::::::::::::::::::::::::::::::::::::::::::::::::

@D0950.29_108572

GATGAACGCTAGCGACAGGCTTAACACATGCAAGTCGAGGGGCAGCACAAGGTAGCAATACTGAGGTGGCGACCGGCGCACGGGTGAGTAACGCGTATGCAACCTACCTCTTAGCGGGGGATAACCCGGCGAAAGTCGGACTAATACCGCATAATACTCTTTCTCCGCATGGAGGAAGATTTTAAAGATTAATTGCTAAGAGATGGGCATGCGTTCCATTAGGTAGTTGGTAGAGGTAACGGCCTACCAAGCCATCGATGGATAGGGGTTCTGAGAGGAAGGTCCCCCACACTGGTACTGAGACACGGACCAGACTCCTACGGGAGGCAGCAGTGAGGAATATTGGTCAATGGACGAGAGTCTGAACCAGCCAAGTCGCGTGAAGGAAGAAGGTTCTATGGATTGTAAACTTCTTTTATAGGGGAATAAAGTGAGGAACGTGTTCCTTTTTGGTATGTACCCTATGAATAAGCATTCGGCTAACTCCGTGCCAGCAGCCG

+

::::::::::::::::::::::::::::::::::::::::::::::::::::::::::::::::::::::::::::::::::::::::::::::::::::::::::::::::::::::::::::::::::::::::::::::::::::::::::::::::::::::::::::::::::::::::::::::::::::::::::::::::::::::::::::::::::::::::::::::::::::::::::::::::::::::::::::::::::::::::::::::::::::::::::::::::::::::::::::::::::::::::::::::::::::::::::::::::::::::::::::::::::::::::::::::::::::::::::::::::::::::::::::::::::::::::::::::::::::::::::::::::::::::::::::::::::::::::::::::::::::::::::::::::::::

@D0950.29_108595

GATGAACGCTAGCGACAGGCCTAACACATGCAAGTCGAGGGGTAGCACAAGGTAGCAATACTGAGGTGACGACCGGCGCACGGGTGAGTAACGCGTATGCAACCTACCTGTAAGAGTGGGATAGCCTCTCGAAAGAGAGATTAATACCGCATAATACCATTTCACTGCATGGTGAGATGGTTAAAGATTTATTGCTTACAGATGGGCATGCGTAACATTAGCTAGTTGGTGAGGTAACGGCTCACCAAGGCAACGATGTTTAGGGGTTCTGAGAGGAAGGTCCCCCACACTGGTACTGAGACACGGACCAGACTCCTACGGGAGGCAGCAGTGAGGAATATTGGTCAATGGACGAGAGTCTGAACCAGCCAAGTCGCGTGAAGGATGAAGGTCTTATGGATTGTAAACTTCTTTTATACGGGAATAAAAATGCCACGTGTGGCATATTGCATGTACCGTATGAATAAGGATCGGCTAACTCCGTGCCAGCAGCCGCGGTAA

+

:::::::::::::::::::::::::::::::::::::::::::::::::::::::::::::::::::::::::::::::::::::::::::::::::::::::::::::::::::::::::::::::::::::::::::::::::::::::::::::::::::::::::::::::::::::::::::::::::::::::::::::::::::::::::::::::::::::::::::::::::::::::::::::::::::::::::::::::::::::::::::::::::::::::::::::::::::::::::::::::::::::::::::::::::::::::::::::::::::::::::::::::::::::::::::::::::::::::::::::::::::::::::::::::::::::::::::::::::::::::::::::::::::::::::::::::::::::::::::::::::::::::::::::::::::::

@D0950.29_10866

GATGAACGCTGGCGGCATGCCTTACACATGCAAGTCGAACGGTAACAGGTCTTCGGATGCTGACGAGTGGCGAACGGGTGAGTAATACATCGGAACGTGCCCGATCGTGGGGATAACGAAGCGAAAGCTTTGCTAATACCGCATACGATCTACGGATGAAAGCAGGGGACCGCAAGGCCTTGCGCGAACGGAGCGGCCGATGGCAGATTAGGTAGTTGGTGGGATAAAAGCTTACCAAGCCGACGATCTGTAGCTGGTCTGAGAGGACGACCAGCCACACTGGGACTGAGACACGGCCCAGACTCCTACGGGAGGCAGCAGTGGGGAATTTTGGACAATGGGCGAAAGCCTGATCCAGCCATGCCGCGTGCAGGATGAAGGCCTTCGGTTGTAAACTGCTTTTGTACGGAACGAAAGACTCTGGTTAATACCTGGGGTCCATGACGGTACCGTAAGAATAAGCACCGGCTAACTACGTGCCAGCAGCCGCGGTAA

+

:::::::::::::::::::::::::::::::::::::::::::::::::::::::::::::::::::::::::::::::::::::::::::::::::::::::::::::::::::::::::::::::::::::::::::::::::::::::::::::::::::::::::::::::::::::::::::::::::::::::::::::::::::::::::::::::::::::::::::::::::::::::::::::::::::::::::::::::::::::::::::::::::::::::::::::::::::::::::::::::::::::::::::::::::::::::::::::::::::::::::::::::::::::::::::::::::::::::::::::::::::::::::::::::::::::::::::::::::::::::::::::::::::::::::::::::::::::::::::::::::::::::::::::::

@D0950.29_108691

GATGAACGCTAGCTACAGGCTTAACACATGCAAGTCGAGGGGTAGCATGAAACTTAGCAATAAGTTTTGATGACGACCGGCGCACGGGTGAGTAACGCGTATGCAACCTGCCTATAAGAAGGGGATAGCCTCTCGAAAGAGAGATTAATACCGTATAACACTATGAAGCCGCATGGTTTTATAGTTAAAGATTTATTGCTTATAGATGGGCATGCGTAACATTAGCTAGTTGGTGAGGTAACGGCTCACCAAGGCAACGATGTTTAGGGGTTCTGAGAGGAAGGTCCCCCACACTGGTACTGAGACACGGACCAGACTCCTACGGGAGGCAGCAGTGAGGAATATTGGTCAATGGACGAGAGTCTGAACCAGCCAAGTCGCGTGAAGGATGAAGGTCTTATGGATTGTAAACTTCTTTTATAAAGGAATAAAGTATGCCACGTGTGGTGTTTTGTATGTACTTTATGAATAAGGATCGGCTAACTCCGTGCCAGCAGCCG

+

::::::::::::::::::::::::::::::::::::::::::::::::::::::::::::::::::::::::::::::::::::::::::::::::::::::::::::::::::::::::::::::::::::::::::::::::::::::::::::::::::::::::::::::::::::::::::::::::::::::::::::::::::::::::::::::::::::::::::::::::::::::::::::::::::::::::::::::::::::::::::::::::::::::::::::::::::::::::::::::::::::::::::::::::::::::::::::::::::::::::::::::::::::::::::::::::::::::::::::::::::::::::::::::::::::::::::::::::::::::::::::::::::::::::::::::::::::::::::::::::::::::::::::::::::::

@D0950.29_108715

GATGAACGCTAGCTACAGGCTTAACACATGCAAGTCGAGGGGTAGCATGAAACTTAGCAATAAGTTTTGATGACGACCGGCGCACGGGTGAGTAACACGTATCCAACCTGCCTTTTACTCATGGATAGCCTTCTGAAAAGAAGATTAATACATGATGGTATTCAGAGTTTTCATGGACACTGAATTAAAGATTTTATCGGTAAAGAGATGGGGAATGCGTTCCATTAGATAGTAGGCGGGGTAACGGCCCACCTAGTCAACGATGGATAGGGGTTCTGAGAGGAAGGTCCCCACATTGGAACTGAGACACGGTGCCAAACTCCTACGGGAGGCAGCAGTGAGGAATATTGGTCAATGGACGTAAGTCTGAACCAGCCAAAGTAGCGTGAAGGATGAAGGCTCTATGGGTCGTAAAACTTCTTTTTATAAAAGGAATAAAGTATGCCACGTGTGGTGTTTTTGTATGT

+

:::::::::::::::::::::::::::::::::::::::::::::::::::::::::::::::::::::::::::::::::::::::::::::::::::::::::::::::::::::::::::::::::::::::::::::::::::::::::::::::::::::::::::::::::::::::::::::::::::::::::::::::::::::::::::::::::::::::::::::::::::::::::::::::::::::::::::::::::::::::::::::::::::::::::::::::::::::::::::::::::::::::::::::::::::::::::::::::::::::::::::::::::::::::::::::::::::::::::::::::::::::::::::::::::::::::::::::::::::::::::::::::::::::::::::::::::::

@D0950.29_108736

GACGAACGCTGGCGGCGTGCCTAACACATGCAAGTCGAGCGAGTTGATCCCTTCGGGGTGAAGCTAGCGGCGGACGGGTGAGTAACACGTGGGCAACCTGCCTCATAGAGGGGAATAGCCTTCCGAAAGGAAGATTAATACCGCATAAGATTGTAGCTTCGCATGAAGTAGCAATTAAAGGAGCAATCCGCTATGAGATGGGCCCCGCGGGCGCATTAGCTAGTTGGTGAGGTAACGGCTCACCAAGGCGACGATGCGTAGCCGACCTGAGAGGGTGATCGGCCACATTGGGACTGAGGACACGGCCCAGACTCCTACGGGAGGCAGCAGTGGGGAATATTGCACAATGGGGGAAACCCTGATGCAGCAACGCCGCGTGAGTGATGAC

+

::::::::::::::::::::::::::::::::::::::::::::::::::::::::::::::::::::::::::::::::::::::::::::::::::::::::::::::::::::::::::::::::::::::::::::::::::::::::::::::::::::::::::::::::::::::::::::::::::::::::::::::::::::::::::::::::::::::::::::::::::::::::::::::::::::::::::::::::::::::::::::::::::::::::::::::::::::::::::::::::::::::::::::::::::::::::::::::::::::::::::::::::::::::::::::::::::::

@D0950.29_108849

GATGAACGCTAGCGACAGGCCTAACACATGCAAGTCGAGGGGTAGCACAAGGTAGCAATACTGAGGTGACGACCGGCGCACGGGTGAGTAACGCGTATGCAACCTGCCTGTAAGAGTGGGATAGCCTCTCGAAAGAGAGATTAATACCGCATAATACCATTTCACTGCATGGTGAGATGGTTAAAGATTTATTGCTTACAGATGGGCATGCGTAACATTAGCTAGTTGGTGAGGTAACGGCTCACCAAGGCAACGATGTTTAGGGGTTCTGAGAGGAAGGTCCCCCACACTGGTACTGAGACACGGACCAGACTCCTACGGGAGGCAGCAGTGAGGAATATTGGTCAATGGACGAGAGTCTGAACCAGCCAAGTCGCGTGAAGGATGAAGGTCTTATGGATTGTAAACTTCTTTTATACGGGAATAAAAATGCCACGTGTGGCATATTGCATGTACCGTATGAATAAGGATCGGCTAACTCCGTGCCAGCAGCCGCGGTAA

+

:::::::::::::::::::::::::::::::::::::::::::::::::::::::::::::::::::::::::::::::::::::::::::::::::::::::::::::::::::::::::::::::::::::::::::::::::::::::::::::::::::::::::::::::::::::::::::::::::::::::::::::::::::::::::::::::::::::::::::::::::::::::::::::::::::::::::::::::::::::::::::::::::::::::::::::::::::::::::::::::::::::::::::::::::::::::::::::::::::::::::::::::::::::::::::::::::::::::::::::::::::::::::::::::::::::::::::::::::::::::::::::::::::::::::::::::::::::::::::::::::::::::::::::::::::::

@D0950.29_108880

GATGAACGCTGGCGGCGTGCTTAACACATGCAAGTCGAACGAAGCACTTAAGGAGCTTGCTCCAAAAGTGACTGAGTGGCGGACGGGTGAGTAACGCGTGGGTAACCTGCCTTACACTGGGGGATAACAGTTGGAAACGACTGCTAATACCGCATAAGCGCACAGTATTGCATGATACAGTGTGAAAAACTCCGGTGGTGTAAGATGGACCCGCGTCTGATTAGCTAGTTGGTGAGGTAATGGCTCACCAAGGCAACGATCAGTAGCCGGCTTGAGAGAGTGAACGGCCACATTGGGACTGAGACACGGCCCAAACTCCTACGGGAGGCAGCAGTGGGGAATATTGCACAATGGGGGAAACCCTGATGCAGCAACGCCGCGTGAGTGAAGAAGTATTTCGGTATGTAAAGCTCTATCAGCAGGGAAGATAATGACGGTACCTGACTAAGAAGCCCCCGGCTAACTACGTGCCAGCAGCCG

+

::::::::::::::::::::::::::::::::::::::::::::::::::::::::::::::::::::::::::::::::::::::::::::::::::::::::::::::::::::::::::::::::::::::::::::::::::::::::::::::::::::::::::::::::::::::::::::::::::::::::::::::::::::::::::::::::::::::::::::::::::::::::::::::::::::::::::::::::::::::::::::::::::::::::::::::::::::::::::::::::::::::::::::::::::::::::::::::::::::::::::::::::::::::::::::::::::::::::::::::::::::::::::::::::::::::::::::::::::::::::::::::::::::::::::::::::::::::::::::::::

@D0950.29_108908

GATGAACGCTAGCGACAGGCCTAACACATGCAAGTCGAGGGGTAGCACAAGGGAGCTTGCTTCTGAGGTGACGACCGGCGCACGGGTGAGTAACGCGTATGCAACCTGCCTATAAGAAGGGGATAGCCTCTCGAAAGAGAGATTAATACCGTATAACACTATGAAGCCGCATGGTTTTATAGTTAAAGATTTATTGCTTATAGATGGGCATGCGTAACATTAGCTAGTTGGTAAGGTAACGGCTTACCAAGGCAACGATGTTTAGGGGTTCTGAGAGGAAGGTCCCCCACACTGGTACTGAGACACGGACCAGACTCCTACGGGAGGCAGCAGTGAGGAATATTGGTCAATGGACGAGAGTCTGAACCAGCCAAGTCGCGTGAAGGATGAAGGTCTTATGGATTGTAAACTTCTTTTATACGGGGAAATAAAAAACTACCACGTGTTGGTATATTGGCATGTACCGTATGAATAAGGATCGGCTAACTCCGTGCCAGCAGCCG

+

:::::::::::::::::::::::::::::::::::::::::::::::::::::::::::::::::::::::::::::::::::::::::::::::::::::::::::::::::::::::::::::::::::::::::::::::::::::::::::::::::::::::::::::::::::::::::::::::::::::::::::::::::::::::::::::::::::::::::::::::::::::::::::::::::::::::::::::::::::::::::::::::::::::::::::::::::::::::::::::::::::::::::::::::::::::::::::::::::::::::::::::::::::::::::::::::::::::::::::::::::::::::::::::::::::::::::::::::::::::::::::::::::::::::::::::::::::::::::::::::::::::::::::::::::::::::

@D0950.29_109009

GATGAACGCTAGCTACAGGCTTAACACATGCAAGTCGAGGGGTAGCATGAAACTTAGCAATAAGTTTTGATGACGACCGGCGCACGGGTGAGTAACACGTATCCAACCTGCCTTTTACTCATGGATAGCCTTCTGAAAAGAAGATTAATACATGATGGTATTCAGAGTTTTCATGGACACTGAATTAAAGATTTATCGGTAAGAGATGGGGATGCGTTCCATTAGATAGTAGGCGGGGTAACGGCCCACCTAGTCAACGATGGATAGGGGTTCTGAGAGGAAGGTCCCCCACATTGGAACTGAGACACGGTCCAAACGTCCTACGGGAGGCAGCAGTGAGGAATATTGGTCAATGGACGTAAGTCTGAACCAGCCAAGTAGCGTGAAGGATGAAGGCTCTATGGGTCGTAAACTTCTTTTATAAAAGGAATAAAGTATGCCACGTGTGGTGTTTTTGTATGTACTTTATGAATAAGGATCGGCTAACTCCGTGCCAGCAGCCGC

+

::::::::::::::::::::::::::::::::::::::::::::::::::::::::::::::::::::::::::::::::::::::::::::::::::::::::::::::::::::::::::::::::::::::::::::::::::::::::::::::::::::::::::::::::::::::::::::::::::::::::::::::::::::::::::::::::::::::::::::::::::::::::::::::::::::::::::::::::::::::::::::::::::::::::::::::::::::::::::::::::::::::::::::::::::::::::::::::::::::::::::::::::::::::::::::::::::::::::::::::::::::::::::::::::::::::::::::::::::::::::::::::::::::::::::::::::::::::::::::::::::::::::::::::::::::::::

@D0950.29_109036

GATGAACGCTGGCGGCGTGCTTAACACATGCAAGTCGAACGAAGCACTTTGAAGAGCTTGCTCTTTAAAGTGACTGAGTGGCGGACGGGTGAGTAACGCGTGGGTAACCTGCCTCATACAGGGGGATAACAGTTAGAAATGACTGCTAACACCGCATAACCCGCTAGTGCGCATGACACAGACGGAAAATATTTATAGGTATGAGATGGGCCCGCGTCTGATTACGCTAGTTGGTGGGGTAACGGCCTACCAAGGCAACGATCAGTAGCCGACTTGAGAGAGTGATCGGCCACATTGGGACTGAGACACGGCCCAAACTCCTACGGGAGGCAGCAGTGGGGAATATTGGACAATGGGGGAAACCCTGATCCAGCGACGCCGCGTGAGTGAAGAAGTATTTCGGTATGTAAAGCTCTATCAGCAGGGGAAAGATAATGACAGTACCTGACTAAGAAGCCCCGGCTAACTACGTGCCAGCAGCCGCGGTAA

+

:::::::::::::::::::::::::::::::::::::::::::::::::::::::::::::::::::::::::::::::::::::::::::::::::::::::::::::::::::::::::::::::::::::::::::::::::::::::::::::::::::::::::::::::::::::::::::::::::::::::::::::::::::::::::::::::::::::::::::::::::::::::::::::::::::::::::::::::::::::::::::::::::::::::::::::::::::::::::::::::::::::::::::::::::::::::::::::::::::::::::::::::::::::::::::::::::::::::::::::::::::::::::::::::::::::::::::::::::::::::::::::::::::::::::::::::::::::::::::::::::::::::::

@D0950.29_109068

GATGAACGCTAGCGACAGGCCTAACACATGCAAGTCGAGGGGTAGCACAAGGAAGCTTGCTTCTGAGGTGACGACCGGCGCACGGGTGAGTAACGCGTATGCAACCTACCTGTAAGAGTGGGATAGCCTCTCGAAAGAGAGATTAATACCGCATAATACCATTTCACTGCATGGTGAGATGGTTAAAGATTTATTGCTTACAGATGGGCATGCGTAACATTAGCTAGTTGGTGAGGTAACGGCTCACCAAGGCAACGATGTTTAGGGGTTCTGAGAGGAAGGTCCCCCACACTGGTACTGAGACACGGACCAGACTCCTACGGGAGGCAGCAGTGAGGAATATTGGTCAATGGACGAGAGTCTGAACCAGCCAAGTCGCGTGAAGGATGAAGGTCTTATGGATTGTAAACTTCTTTTATACGGGAATAAAAATGCCACGTGTGGCATATTGCATGTACCGTATGAATAAGGATCGGCTAACTCCGTGCCAGCAGCCGCGGTAA

+

:::::::::::::::::::::::::::::::::::::::::::::::::::::::::::::::::::::::::::::::::::::::::::::::::::::::::::::::::::::::::::::::::::::::::::::::::::::::::::::::::::::::::::::::::::::::::::::::::::::::::::::::::::::::::::::::::::::::::::::::::::::::::::::::::::::::::::::::::::::::::::::::::::::::::::::::::::::::::::::::::::::::::::::::::::::::::::::::::::::::::::::::::::::::::::::::::::::::::::::::::::::::::::::::::::::::::::::::::::::::::::::::::::::::::::::::::::::::::::::::::::::::::::::::::::::::

@D0950.29_109077

GATGAACGCTAGCGACAGGCCTAACACATGCAAGTCGAGGGGTAGCACAAGGTAGCAATACTGAGGTGACGACCGGCGCACGGGTGAGTAACGCGTATGCAACCTACCTGTAAGAGTGGGATAGCCTCTCGAAAGAGAGATTAATACCGCATAATACCATTTCACTGCATGGTGAGATGGTTAAAGATTTATTGCTTACAGATGGGCATGCGTAACATTAGCTAGTTGGTGAGGTAACGGCTCACCAAGGCAACGATGTTTAGGGGTTCTGAGAGGAAGGTCCCCCACACTGGTACTGAGACACGGACCAGACTCCTACGGGAGGCAGCAGTGAGGAATATTGGTCAATGGACGAGAGTCTGAACCAGCCAAGTCGCGTGAAGGATGAAGGTCTTATGGATTGTAAACTTCTTTTATACGGGAATAAAAATGCCACGTGTGGCATATTGCATGTACCGTATGAATAAGGATCGGCTAACTCCGTGCCAGCAGCCGC

+

::::::::::::::::::::::::::::::::::::::::::::::::::::::::::::::::::::::::::::::::::::::::::::::::::::::::::::::::::::::::::::::::::::::::::::::::::::::::::::::::::::::::::::::::::::::::::::::::::::::::::::::::::::::::::::::::::::::::::::::::::::::::::::::::::::::::::::::::::::::::::::::::::::::::::::::::::::::::::::::::::::::::::::::::::::::::::::::::::::::::::::::::::::::::::::::::::::::::::::::::::::::::::::::::::::::::::::::::::::::::::::::::::::::::::::::::::::::::::::::::::::::::::::::::

@D0950.29_109118

GATGAACGCTGGCGGCGTGCTTAACACATGCAAGTCGAACGAAGCAGCTTTCTTGCTTGCAAGAAAGCTGACTTAGTGGCGGACGGGTGAGTAACGCGTGGGTAACCTGCCTCATACAGGGGATAACAGTTGGAAACGACTGCTAAGACCGCATAACCCGCTAGTGTCGCATGACACGGACGGAAAATATTTTATAGGTATGAGATGGGCCCGCCGTCTGATTAGCCTAGTTGGTAACGGTAACGGCTTACCAAGGCGACGATCAGTAGCCGACTTGAGAGAGTGATCGGCCACATTGGGACTGAGACACGGCCCAAACTCCTACGGGAGGCAGCAGTGGGGAATATTGGACAATGGGGGAAACCCTGATCCAGCGACGCCGCGTGAGTGAAGAAGTATTTCGGTATGTAAAGCTCTATCAGCAGGGAAGATAATGACAGTACCTGAC

+

::::::::::::::::::::::::::::::::::::::::::::::::::::::::::::::::::::::::::::::::::::::::::::::::::::::::::::::::::::::::::::::::::::::::::::::::::::::::::::::::::::::::::::::::::::::::::::::::::::::::::::::::::::::::::::::::::::::::::::::::::::::::::::::::::::::::::::::::::::::::::::::::::::::::::::::::::::::::::::::::::::::::::::::::::::::::::::::::::::::::::::::::::::::::::::::::::::::::::::::::::::::::::::::::::::::::::::::::::::::::::::::::

@D0950.29_109138

GATGAACGCTGGCGGCGTGCTTAACACATGCAAGTCGAACGAAGCACTTTGAAGAGCTTGCTCTTTAAAGTGACTGAGTGGCGGACGGGTGAGTAACGCGTGGGTAACCTGCCTCATACAGGGGGATAACAGTTAGAAATGACTGCTAACACCGCATAACCCGCTAGTGTCGCATGACACAGACGGAAAATATTTATAGGTATGAGATGGGCCCGCGTCTGATTAGCTAGTTGGTGGGGTAACGGCCTACCAAGGCAACGATCAGTAGCCGACTTGAGAGAGTGATCGGCCACATTGGGACTGAGACACGGCCCAAACTCCTACGGGAGGCAGCAGTGGGGAATATTGGACAATGGGGGAAACCCTGATCCAGCGACGCCGCGTGAGTGAAGAAGTATTTCGGTATGTAAAGCTCTATCAGCAGGGAAGATAATGACAGTACCTGACTAAGAAGCCCCGGCTAACTACGTGCCAGCAGCCGCGGTAA

+

:::::::::::::::::::::::::::::::::::::::::::::::::::::::::::::::::::::::::::::::::::::::::::::::::::::::::::::::::::::::::::::::::::::::::::::::::::::::::::::::::::::::::::::::::::::::::::::::::::::::::::::::::::::::::::::::::::::::::::::::::::::::::::::::::::::::::::::::::::::::::::::::::::::::::::::::::::::::::::::::::::::::::::::::::::::::::::::::::::::::::::::::::::::::::::::::::::::::::::::::::::::::::::::::::::::::::::::::::::::::::::::::::::::::::::::::::::::::::::::::::::::::

@D0950.29_109167

GATGAACGCTAGCTACAGGCTTAACACATGCAAGTCGAGGGGTAGCATGAAACTTAGCAATAAGTTTTGATGACGACCGGCGCACGGGTGAGTAACACGTATCCAACCTGCCTTTTACTCATGGATAGCCTTCTGAAAAGAAGATTAATACATGATGGTATTCAGAGTTTTCATGGACACTGAATTAAAGATTTTATCGGTAAGAGATGGGGATGCGTTCCATTAGATAGTAGGCGGGGTAACGGCCCACCTAGTCAACGATGGATAGGGGTTCTGAGAGGAAGGTCCCCCACATTGGAACTGAGACACGGTCCAAACGTCCTACGGGAGGCAGCAGTGAGGAATATTGGTCAATGGACGTAAGTCTGAACCAGCCAAGTAGCGTGAAGGATGAAGGCTCTATGGGTCGTAAACTTCTTTTTATAAAAGG

+

::::::::::::::::::::::::::::::::::::::::::::::::::::::::::::::::::::::::::::::::::::::::::::::::::::::::::::::::::::::::::::::::::::::::::::::::::::::::::::::::::::::::::::::::::::::::::::::::::::::::::::::::::::::::::::::::::::::::::::::::::::::::::::::::::::::::::::::::::::::::::::::::::::::::::::::::::::::::::::::::::::::::::::::::::::::::::::::::::::::::::::::::::::::::::::::::::::::::::::::::::::::::::::::::::::::::::::::

@D0950.29_109201

GATGAACGCTAGCGACAGGCCTAACACATGCAAGTCGAGGGGTAGCACAAGGTAGCAATACTGAGGTGACGACCGGCGCACGGGTGAGTAACGCGTATGCAACCTACCTGTAAGAGTGGGATAGCCTCTCGAAAGAGAGATTAATACCGCATAATACCATTTCACTGCATGGTGAGATGGTTAAAGATTTATTGCTTACAGATGGGCATGCGTAACATTAGCTTGTTGGTGAGGTAACGGCTCACCAAGGCAACGATGTTTAGGGGTTCTGAGAGGAAGGTCCCCCACACTGGTACTGAGACACGGGACCAGACTCCTACGGGAGGCAGCAGTGAGGAATATTGGTCAATGGACGAGAGTCTGAACCAGCCAAGTCGCGTGAAGGATGAAGGTCTTATGGATTGTAAACTTCTTTTATACGGGAATAAAAAAGGCCACGTGTGGTTTATTGCATGTACCGTATGAATAAGGATCGGCT

+

::::::::::::::::::::::::::::::::::::::::::::::::::::::::::::::::::::::::::::::::::::::::::::::::::::::::::::::::::::::::::::::::::::::::::::::::::::::::::::::::::::::::::::::::::::::::::::::::::::::::::::::::::::::::::::::::::::::::::::::::::::::::::::::::::::::::::::::::::::::::::::::::::::::::::::::::::::::::::::::::::::::::::::::::::::::::::::::::::::::::::::::::::::::::::::::::::::::::::::::::::::::::::::::::::::::::::::::::::::::::::::::::::::::::::::::::::::::::::::::

@D0950.29_109207

GATGAACGCTAGCGACAGGCCTAACACATGCAAGTCGAGGGGTAGCACAAGGTAGCAATACTGAGGTGACGACCGGCGCACGGGTGAGTAACGCGTATGCAACCTACCTGTAAGAGTGGGATAGCCTCTCGAAAGAGAGATTAATACCGCATAATACCATTTCACTGCATGGTGAGATGGTTAAAGATTTATTGCTTACAGATGGGCATGCGTAACATTAGCTAGTTGGTGAGGTAACGGCTCACCAAGGCAACGATGTTTAGGGGTTCTGAGAGGAAGGTCCCCCACACTGGTACTGAGACACGGACCAGACTCCTACGGGAGGCAGCAGTGAGGAATATTGGTCAATGGGCGAGAGCCTGAACCAGCCAAGTAGCGTGAAGGATGAAGGCTCTATGGGTCGTAAACTTCTTTTATAAAGGAATAAAGTGAGCCACGTGTGGCTTTTTGTATGTACTTTATGAATAAGGATCGGCTAACTCCGTGCCAGCAGCCGCGGTAA

+

::::::::::::::::::::::::::::::::::::::::::::::::::::::::::::::::::::::::::::::::::::::::::::::::::::::::::::::::::::::::::::::::::::::::::::::::::::::::::::::::::::::::::::::::::::::::::::::::::::::::::::::::::::::::::::::::::::::::::::::::::::::::::::::::::::::::::::::::::::::::::::::::::::::::::::::::::::::::::::::::::::::::::::::::::::::::::::::::::::::::::::::::::::::::::::::::::::::::::::::::::::::::::::::::::::::::::::::::::::::::::::::::::::::::::::::::::::::::::::::::::::::::::::::::::::::

@D0950.29_109288

GATGAACGCTAGCTACAGGCTTAACACATGCAAGTCGAGGGGTAGCATGAAACTTAGCAATAAGTTTTGATGACGACCGGCGCACGGGTGAGTAACACGTATCCAACCTGCCTTTTACTCATGGATAGCCTTCTGAAAAGAAGATTAATACATGATGGTATTCAGAGTTTTCATGGACACTGAATTAAAGATTTTATCGGTAAGAGATGGGGATGCGTTCCATTAGATAGTAGGCGGGGTAACGGCCCACCTAGTCAACGATGGATAGGGGTTCTGAGAGGAAGGTCCCCCACATTGGAACTGAGACACGGTCCAAACGTCCTACGGGAGGCAGCAGTGAGGAATATTGGTCAATGGACGTAAGTCTGAACCAGCCAAGTAGCGTGAAGGATGAAGGCTCTATGGGTCGTAAACTTCTTTTTA

+

:::::::::::::::::::::::::::::::::::::::::::::::::::::::::::::::::::::::::::::::::::::::::::::::::::::::::::::::::::::::::::::::::::::::::::::::::::::::::::::::::::::::::::::::::::::::::::::::::::::::::::::::::::::::::::::::::::::::::::::::::::::::::::::::::::::::::::::::::::::::::::::::::::::::::::::::::::::::::::::::::::::::::::::::::::::::::::::::::::::::::::::::::::::::::::::::::::::::::::::::::::::::::::::::::::::::

@D0950.29_109289

GATGAACGCTGGCGGCGTGCTTAACACATGCAAGTCGAACGAAGCACTTTGAAGAGCTTGCTCTTTGAAGTGACTGAGTGGCGGACGGGTGAGTAACGCGTGGGTAACCTGCCTTACACTGGGGGACTAACAGTTAGAAATGACTGCTAACACCCGCATAACCCGCTAGTGTCGCATGACACAGACGGAAAATATTTATAGGTATGAGATGGGCCCGCGTCTGATTAGCTAGTTGGTGGGTAACGGCCTACCAAGGCAACGAGTCAGTAGCCGACTTGAGAGAGTGATCGGCCACATTGGGACTGAGACACGGCCCAAACTCCTACGGGAGGCAGCAGTGGGGAATATTGGACAATGGGGGAAACCCTGATCCAGCGACGCCGGCGTAGTGAAGAAGTATTTTCGGTATGTAAAGCTCTATCAGCAGGGAAGATAATGACAGTACCTGACTAAGAAGCCCCCGGCTAA

+

::::::::::::::::::::::::::::::::::::::::::::::::::::::::::::::::::::::::::::::::::::::::::::::::::::::::::::::::::::::::::::::::::::::::::::::::::::::::::::::::::::::::::::::::::::::::::::::::::::::::::::::::::::::::::::::::::::::::::::::::::::::::::::::::::::::::::::::::::::::::::::::::::::::::::::::::::::::::::::::::::::::::::::::::::::::::::::::::::::::::::::::::::::::::::::::::::::::::::::::::::::::::::::::::::::::::::::::::::::::::::::::::::::::::::::::::::::

@D0950.29_109292

GATGAACGCTAGCTACAGGCTTAACACATGCAAGTCGAGGGGTAGCATGAAACTTAGCAATAAGTTTTGATGACGACCGGCGCACGGGTGAGTAACACGTATCCAACCTGCCTTTTACTCATGGATAGCCTTCTGAAAAGAAGATTAATACATGATGGTATTCAGAGTTTTCATGGACACTGAATTAAAGATTTTATCGGTAAGAGATGGGGATGCGTTCCATTAGATAGTAGGCGGGGTAACGGCCCACCTAGTCAACGATGGATAGGGGTTCTGAGAGGAAGGTCCCCCACATTGGAACTGAGACACGGTCCAAACGTCCTACGGGAGGCAGCAGTGAGGAATATTGGTCAATGGACGTAAGTCTGAACCAGCCAAGTAGCGTGAAGGATGAAGGCTCTATGGGTCGTAAACTTCTTTTTATAAAA

+

::::::::::::::::::::::::::::::::::::::::::::::::::::::::::::::::::::::::::::::::::::::::::::::::::::::::::::::::::::::::::::::::::::::::::::::::::::::::::::::::::::::::::::::::::::::::::::::::::::::::::::::::::::::::::::::::::::::::::::::::::::::::::::::::::::::::::::::::::::::::::::::::::::::::::::::::::::::::::::::::::::::::::::::::::::::::::::::::::::::::::::::::::::::::::::::::::::::::::::::::::::::::::::::::::::::::::::

@D0950.29_109302

GATGAACGCTGGCGGCGTGCTTAACACATGCAAGTCGAACGAAGCACTTAAGGAGCTTGCTCCAAAGGTGACTGAGTGGCGGACGGGTGAGTAACGCGTGGGTAACCTGCCTTACACTGGGGGATAACAGTTGGAAACGACTGCTAATACCGCATAAGCGCACAGTATTGCATGATACAGTGTGAAAAACTCCGGTGGTGTAAGATGGACCCGCGTCTGATTAGCTAGTTGGTGAGGTAATGGCTCACCAAGGCAACGATCAGTAGCCGGCTTGAGAGAGTGAACGGCCACATTGGGACTGAGACACGGCCCAAACTCCTACGGGAGGCAGCAGTGGGAATATTGCACAATGGGGGAAACCCTGATGCAGCAACGCCGCGTGAGTGAAGAAGTATTTGCGGTATGTAAAGCTCTATCAGCAGGGAAGATAATGACGGTACCTGACTAAGAAGCCCCCGGCTAACTACGTGCCAGCAGCCG

+

::::::::::::::::::::::::::::::::::::::::::::::::::::::::::::::::::::::::::::::::::::::::::::::::::::::::::::::::::::::::::::::::::::::::::::::::::::::::::::::::::::::::::::::::::::::::::::::::::::::::::::::::::::::::::::::::::::::::::::::::::::::::::::::::::::::::::::::::::::::::::::::::::::::::::::::::::::::::::::::::::::::::::::::::::::::::::::::::::::::::::::::::::::::::::::::::::::::::::::::::::::::::::::::::::::::::::::::::::::::::::::::::::::::::::::::::::::::::::::::::

@D0950.29_109358

GATGAACGCTAGCGACAGGCCTAACACATGCAAGTCGAGGGGTAGCACAAGGAAGCTTGCTTCTGAGGTGACGACCGGCGCACGGGTGAGTAACGCGTATGCAACCTACCTGTAAGAGTGGGATAGCCTCTCGAAAGAGAGATTAATACCGCATAATACCATTTCACTGCATGGTGAGATGGTTAAAGATTTATTGCTTACAGATGGGCATGCGTAACATTAGCTAGTTGGTGAGGTAACGGCTCACCAAGGCAACGATGTTTAGGGGTTCTGAGAGGAAGGTCCCCCGACACTGGTACTGAGACACGGACCAGACTCCTACGGGAGGCAGCAGTGAGGAATATTGGTCAATGGACGAGAGTCTGAACCAGCCAAGTCGCGTGAAGGATGAAGGTCTTATGGATTGTAAACTCTTTTATACGGGAATAAAAATGCCACGTGTGGCATATTGCATGTACCGTATGAATAAGGATCGGCTAACTCCGTGCCAGCAGCCGCGGTAA

+

:::::::::::::::::::::::::::::::::::::::::::::::::::::::::::::::::::::::::::::::::::::::::::::::::::::::::::::::::::::::::::::::::::::::::::::::::::::::::::::::::::::::::::::::::::::::::::::::::::::::::::::::::::::::::::::::::::::::::::::::::::::::::::::::::::::::::::::::::::::::::::::::::::::::::::::::::::::::::::::::::::::::::::::::::::::::::::::::::::::::::::::::::::::::::::::::::::::::::::::::::::::::::::::::::::::::::::::::::::::::::::::::::::::::::::::::::::::::::::::::::::::::::::::::::::::::

@D0950.29_109394

GATGAACGCTAGCGACAGGCCTAACACATGCAAGTCGAGGGGTAGCACAAGGTAGCAATACTGAGGTGACGACCGGCGCACGGGTGAGTAACGCGTATGCAACCTACCTGTAAGAGTGGGATAGCCTCTCGAAAGAGAGATTAATACCGCATAATACCATTTCACTGCATGGTGAGATGGTTAAAGATTTATTGCTTACAGATGGGCATGCGTAACATTAGCTAGTTGGTGAGGTAACGGCTCACCAAGGCAACGATGTTTAGGGGTTCTGAGAGGAAGGTCCCCCACACTGGTACTGAGACACGGACCAGACTCCTACGGGAGGCAGCAGTGAGGAATATTGGTCAATGGACGAGAGTCTGAACCAGCCAAGTCGCGTGAAGGATGAAGGTCTTATGGATTGTAAACCTCTTTTGTCAGGGAGCAACGACATCCACGAGTGGGTGAATGAGAGTACCTGAAGAAAAAGCATCGGCTAACTCCGTGCCAGCAGCCGCGGT

+

::::::::::::::::::::::::::::::::::::::::::::::::::::::::::::::::::::::::::::::::::::::::::::::::::::::::::::::::::::::::::::::::::::::::::::::::::::::::::::::::::::::::::::::::::::::::::::::::::::::::::::::::::::::::::::::::::::::::::::::::::::::::::::::::::::::::::::::::::::::::::::::::::::::::::::::::::::::::::::::::::::::::::::::::::::::::::::::::::::::::::::::::::::::::::::::::::::::::::::::::::::::::::::::::::::::::::::::::::::::::::::::::::::::::::::::::::::::::::::::::::::::::::::::::::::

@D0950.29_1094

GATGAACGCTAGCGACAGGCCTAACACATGCAAGTCGAGGGGTAGCACAAGGAAGCTTGCTTCTGAGGTGACGACCGGCGCACGGGTGAGTAACGCGTATGCAACCTACCTGTAAGAGTGGGATAGCCTCTCGAAAGAGAGATTAATACCGCATAATACCATTTCACTGCATGGTGAGATGGTTAAAGATTTATTGCTTACAGATGGGCATGCGTAACATTAGCTAGTTGGTGAGGTAACGGCTCACCAAGGCAACGATGTTTAGGGGTTCTGAGAGGAAGGTCCCCCGACACTGGTACTGAGACACGGACCAGACTCCTACGGGAGGCAGCAGTGAGGAATATTGGTCAATGGACGAGAGTCTGAACCAGCCAAGTCGCGTGAAGGATGAAGGTCTTATGGATTGTAAACCTTCTTTTATACGGGAATAAAAAAGTGCCACGTGTGGCATATTGCATGTACCGTATGAATAAGGATCGGCTAACTCCGTGCCAGCAGCCGCGGTAA

+

:::::::::::::::::::::::::::::::::::::::::::::::::::::::::::::::::::::::::::::::::::::::::::::::::::::::::::::::::::::::::::::::::::::::::::::::::::::::::::::::::::::::::::::::::::::::::::::::::::::::::::::::::::::::::::::::::::::::::::::::::::::::::::::::::::::::::::::::::::::::::::::::::::::::::::::::::::::::::::::::::::::::::::::::::::::::::::::::::::::::::::::::::::::::::::::::::::::::::::::::::::::::::::::::::::::::::::::::::::::::::::::::::::::::::::::::::::::::::::::::::::::::::::::::::::::::::::

@D0950.29_109420

GATGAACGCTAGCGACAGGCCTAACACATGCAAGTCGAGGGGTAGCACAAGGTAGTAATACTGAGGTGACGACCGGCGCACGGGTGAGTAACGCGTATGCAACCTACCTGTAAGAGTGGGATAGCCTCTCGAAAGAGAGATTAATACCGCATAATACCATTTCACTGCATGGTGAGATGGTTAAAGATTTATTGCTTACAGATGGGCATGCGTAACATTAGCTAGTTGGTGAGGTAACGGCTCACCAAGGCAACGATGTTTAGGGGTTCTGAGAGGAAGGTCCCCCACACTGGAACTGAGACACGGTCCAAACTCCTACGGGAGGCAGCAGTGAGGAATATTGGTCAATGGACGTAAGTCTGAACCAGCCAAGTAGCGTGAAGGATGAAGGCTCTATGGGTCGTAAACTTCTTTTATAAAGGAATAAAGTATGCCACGTGTGGTGTTTTGTATGTACTTTATGAATAAGGATCGGCT

+

:::::::::::::::::::::::::::::::::::::::::::::::::::::::::::::::::::::::::::::::::::::::::::::::::::::::::::::::::::::::::::::::::::::::::::::::::::::::::::::::::::::::::::::::::::::::::::::::::::::::::::::::::::::::::::::::::::::::::::::::::::::::::::::::::::::::::::::::::::::::::::::::::::::::::::::::::::::::::::::::::::::::::::::::::::::::::::::::::::::::::::::::::::::::::::::::::::::::::::::::::::::::::::::::::::::::::::::::::::::::::::::::::::::::::::::::::::::::::::::

@D0950.29_109650

GATGAACGCTAGCGACAGGCCTAACACATGCAAGTCGAGGGGTAGCACAAGGTAGCAATACTGAGGTGACGACCGGCGCACGGGTGAGTAACGCGTATGCAACCTACCTGTAAGAGTGGGATAGCCTCTCGAAAGAGAGATTAATACCGCATAATACCATTTTACTGCATGGTGAGATGGTTAAAGATTTGTTGCTTACAGATGGGCATGCGTAACATTAGCTAGTTGGTGAGGTAACGGCTCACCAAGGCAACGATGTTTAGGGGTTCTGAGAGGAAGGTCCCCCACACTGGTACTGAGACACGGACCAGACTCCTACGGGAGGCAGCAGTGAGGAATATTGGTCAATGGACGAGAGTCTGAACCAGCCAAGTCGCGTGAAGGATGAAGGTCTTATGGATTGTAAACTTCTTTTATACGGGAATAAAAAATGCCACGTGTGGCATATTGCATGTACCGTATGAATAAGGATCGGCTAACTCCGTGCCAGCAGCCGCGGTAA

+

::::::::::::::::::::::::::::::::::::::::::::::::::::::::::::::::::::::::::::::::::::::::::::::::::::::::::::::::::::::::::::::::::::::::::::::::::::::::::::::::::::::::::::::::::::::::::::::::::::::::::::::::::::::::::::::::::::::::::::::::::::::::::::::::::::::::::::::::::::::::::::::::::::::::::::::::::::::::::::::::::::::::::::::::::::::::::::::::::::::::::::::::::::::::::::::::::::::::::::::::::::::::::::::::::::::::::::::::::::::::::::::::::::::::::::::::::::::::::::::::::::::::::::::::::::::

@D0950.29_109713

GACGAACGCTGGCGGCATGCCTAACACATGCAAGTCGAACGGAGTTAAGAAGCTTGCTTCTTAACTTAGTGGCGGACGGGTGAGTAACGCGTGAGTAACCTGCCTTTCAGAGGGGATGAACGATCTTGAAAAGAACGCTAATACCGCATAAGATTGTAGAGCCGCATGGTTTAGCAATCAAAGGAGTAATCCGCTGAAAGATGGACTCGCGTCCGATTAGCTAGTTGGTGAGATAAAGGCCCACCAAGGCGACGATCGGTAGCCGGACTGAGAGGTTGAACGGCCACATTGGGACTGAGACACGGCCCAGACTCCTACGGGAGGCAGCAGTGGGGAGTATTGCACAATTGGGGAAAGCCCCTGATGCAGGCAATGCGCGTGAAGGAAGAGGTCTTCGGATTGTAAACTTTTGTCC

+

:::::::::::::::::::::::::::::::::::::::::::::::::::::::::::::::::::::::::::::::::::::::::::::::::::::::::::::::::::::::::::::::::::::::::::::::::::::::::::::::::::::::::::::::::::::::::::::::::::::::::::::::::::::::::::::::::::::::::::::::::::::::::::::::::::::::::::::::::::::::::::::::::::::::::::::::::::::::::::::::::::::::::::::::::::::::::::::::::::::::::::::::::::::::::::::::::::::::::::::::::::::::::::::::

@D0950.29_109772

GATGAACGCTAGCGACAGGCCTAACACATGCAAGTCGAGGGGTAGCACAAGGTAGCAATACTGAGGTGACGACCGGCGCACGGGTGAGTAACGCGTATGCAACCTACCTGTAAGAGTGGGATAGCCTCTCGAAAGAGAGATTAATACCGCATAATACCATTTCACTGCATGGTGAGATGGTTAAAGATTTATTGCTTACAGATGGGCATGCGTAACATTAGCTAGTTGGTGAGGTAACGGCTCACCAAGGCAACGATGTTTAGGGGTTCTGAGAGGAAGGTCCCCCGACACTGGTACTGAGACACGGACCAGACTCCTACGGGAGGCAGCAGTGAGGAATATTGGTCAATGGACGAGAGTCTGAACCAGCCAAGTCGCGTGAAGGATGAAGGTCTTATGGATTGTAAACTTCTTTTATACGGGAATAAAAAGAGCCACGTGTGGTTTATTGCATGTACCGTAATGAATAAGGATCGGCTAACTCCGTGCCAGCAGCCGCGTAA

+

:::::::::::::::::::::::::::::::::::::::::::::::::::::::::::::::::::::::::::::::::::::::::::::::::::::::::::::::::::::::::::::::::::::::::::::::::::::::::::::::::::::::::::::::::::::::::::::::::::::::::::::::::::::::::::::::::::::::::::::::::::::::::::::::::::::::::::::::::::::::::::::::::::::::::::::::::::::::::::::::::::::::::::::::::::::::::::::::::::::::::::::::::::::::::::::::::::::::::::::::::::::::::::::::::::::::::::::::::::::::::::::::::::::::::::::::::::::::::::::::::::::::::::::::::::::::

@D0950.29_109778

GACGAACGCTGGCGGCGTGCCTAACACATGCAAGTCGAGCGAGTTGATCCCTTCGGGGTGAAGCTAGCGGCGGACGGGTGAGTAACGCGTGGGTAACCTGCCTTACACTGGGGGATAACAGTTGGAAACGACTGCTAATACCGCATAAGCGCACAGTATTGCATGATACAGTGTGAAAAACTCCGGTGGTGTAAGATGGACCCGCGTCTGATTAGCTAGTTGGTGAGGTAATGGCTCACCAAGGCAACGATCAGTAGCCGGCTTGAGAGAGTGAACGGCCACATTGGGACTGAGACACGGCCCAAACTCCTACGGGAGGCAGCAGTGGGAATATTGCACAATGGGGGAAACCCTGATGCAGCAACGCCGCGTGAGTGAAGAAGTATTTCGGTATGTAAAGCTCTATCAGCAGGGAAGATAATGACGGTACCTGACTAAGAAGCCCCCGGCTAACTACGTGCCAGCAGCCG

+

::::::::::::::::::::::::::::::::::::::::::::::::::::::::::::::::::::::::::::::::::::::::::::::::::::::::::::::::::::::::::::::::::::::::::::::::::::::::::::::::::::::::::::::::::::::::::::::::::::::::::::::::::::::::::::::::::::::::::::::::::::::::::::::::::::::::::::::::::::::::::::::::::::::::::::::::::::::::::::::::::::::::::::::::::::::::::::::::::::::::::::::::::::::::::::::::::::::::::::::::::::::::::::::::::::::::::::::::::::::::::::::::::::::::::::::::::::::

@D0950.29_109809

ATTGAACGCTGGCGGCATGCCTTACACATGCAAGTCGAACGGTAACAGGTCTTCGGATGCTGACGAGTGGCGAACGGGTGAGTAATACATCGGAACGTGCCCGATCGTGGGGGATAACGAAGCGAAAGCTTTGCTAATACCGCATACGATCTACGGATGAAAGCAGGGGACCGCAAGGCCTTGCGCGAACGGAGCGGCCGATGGCAGATTAGGTAGTTGGTGGGATAAAAGCTTACCAAGCCGACGATCTGTAGCTGGTCTGAGAGGACGACCAGCCACACTGGGACTGAGACACGGCCCAGACTCCTACGGGAGGCAGCAGTGGGGAATTTTGGACAATGGGCGAAAGCCTGATCCAGCCATGCCGCGTGCAGGATGAAGGCCTCGGGTTGTAAACTGCTTTTGTACGGAACGAAAAGACTCTGGTTAATACCTGGGGGTTCCATGACGGTACCGTAAGAATAAGCACCGGCTAACTACGTGCCAGCAGCCGCGGTAA

+

:::::::::::::::::::::::::::::::::::::::::::::::::::::::::::::::::::::::::::::::::::::::::::::::::::::::::::::::::::::::::::::::::::::::::::::::::::::::::::::::::::::::::::::::::::::::::::::::::::::::::::::::::::::::::::::::::::::::::::::::::::::::::::::::::::::::::::::::::::::::::::::::::::::::::::::::::::::::::::::::::::::::::::::::::::::::::::::::::::::::::::::::::::::::::::::::::::::::::::::::::::::::::::::::::::::::::::::::::::::::::::::::::::::::::::::::::::::::::::::::::::::::::::::::::::

@D0950.29_109877

GATGAACGCTGGCGGCGTGCTTAACACATGCAAGTCGAACGAAGCAACTTTCTTGCTTGCAAGAAAGTTGACTGAGTGGCGGACGGGTGAGTAACGCGTGGGTAACCTGCCTCATACAGGGGGATAACAGTTAGAAATGACTGCTAACACCGCATAACCCGCTAGCATCGCATGATGCAGACGGAAAATATTTATAGGTATGAGATGGGCCCGCGTCTGATTAGCTAGTTGGTGGGGTAACAGCCCTACCAAGGCAACGATCAGTAGCCGACTTGAGAGAGTGATCGGCCACATTGGGACTGAGACACGGCCCAAACTCCTACGGGAGGCAGCAGTGGGGAATATTGGACAATGGGGGAAACCCTGATCCAGCGACGCCGCGTGAGTGAAGAAGTATTTCGGTATGTAAAGCTCTATCAGCAGGGAAGATAATGACAGTACCTGACTAAGAAGCCCCCGGCTAACTACGTGCCAGCAGCCGC

+

::::::::::::::::::::::::::::::::::::::::::::::::::::::::::::::::::::::::::::::::::::::::::::::::::::::::::::::::::::::::::::::::::::::::::::::::::::::::::::::::::::::::::::::::::::::::::::::::::::::::::::::::::::::::::::::::::::::::::::::::::::::::::::::::::::::::::::::::::::::::::::::::::::::::::::::::::::::::::::::::::::::::::::::::::::::::::::::::::::::::::::::::::::::::::::::::::::::::::::::::::::::::::::::::::::::::::::::::::::::::::::::::::::::::::::::::::::::::::::::::::

@D0950.29_109893

GATGAACGCTAGCGACAGGCCTAACACATGCAAGTCGAGGGGTAGCACAAGGAAGCTTGCTTCTGAGGTGACGACCGGCGCACGGGTGAGTAACGCGTATGCAACCTACCTGTAAGAGTGGGATAGCCTCTCGAAAGAGAGATTAATACCGCATAATACCATTTCACTGCATGGTGAGATGGTTAAAGATTTATTGCTTACAGATGGGCATGCGTAACATTAGCTAGTTGGTGAGGTAACGGCTCACCAAGGCAACGATGTTTAGGGGTTCTGAGAGGAAGGTCCCCCACACTGGTACTGAGACACGGACCAGACTCCTACGGGAGGCAGCAGTGAGGAATATTGGTCAATGGACGAGAGTCTGAACCAGCCAAGTCGCGTGAAGGATGAAGGTCTTATGGATTGTAAACTTCTTTTATACGGGAATAAAAATGCCACGTGTGGCATATTGCATGTACCGTATGAATAAGGATCGGCTAACTCCGTGCCAGCAGCCGCGGTAA

+

:::::::::::::::::::::::::::::::::::::::::::::::::::::::::::::::::::::::::::::::::::::::::::::::::::::::::::::::::::::::::::::::::::::::::::::::::::::::::::::::::::::::::::::::::::::::::::::::::::::::::::::::::::::::::::::::::::::::::::::::::::::::::::::::::::::::::::::::::::::::::::::::::::::::::::::::::::::::::::::::::::::::::::::::::::::::::::::::::::::::::::::::::::::::::::::::::::::::::::::::::::::::::::::::::::::::::::::::::::::::::::::::::::::::::::::::::::::::::::::::::::::::::::::::::::::::

@D0950.29_109905

GATGAACGCTAGCTACAGGCTTAACACATGCAAGTCGAGGGGTAGCATGAAACTTAGCAATAAGTTTTGATGACGACCGGCGCACGGGTGAGTAACACGTATCCAACCTGCCTTTTACTCATGGATAGCCTTCTGAAAAGAAGATTAATACATGATGGTATTCAGAGTTTTCATGGACACTGAATTAAAGATTTATCGGTAAGAGATGGGGATGCGTTCCATTAGATAGTAGGCGGGGTAACGGCCCACCTAGTCAACGATGGATAGGGGTTCTGAGAGGAAGGTCCCCCACATTGGAACTGAGACACGGTCCAAACTCCTACGGGAGGCAGCAGTGAGGAATATTGGTCAATGGACGTAAGTCTGAACCAGCCAAGTAGCGTGAAGGATGAAGGCTCTATGGGTCGTAAACTTCTTTTTATAAAAGGAATAAAGTATGCCACGTGTGGTGTTTTTGTATGTACTTTATGAATAAGGATCGGCTAACTCCGTGCCAGCAGCCGC

+

::::::::::::::::::::::::::::::::::::::::::::::::::::::::::::::::::::::::::::::::::::::::::::::::::::::::::::::::::::::::::::::::::::::::::::::::::::::::::::::::::::::::::::::::::::::::::::::::::::::::::::::::::::::::::::::::::::::::::::::::::::::::::::::::::::::::::::::::::::::::::::::::::::::::::::::::::::::::::::::::::::::::::::::::::::::::::::::::::::::::::::::::::::::::::::::::::::::::::::::::::::::::::::::::::::::::::::::::::::::::::::::::::::::::::::::::::::::::::::::::::::::::::::::::::::::::

@D0950.29_10995

ATTGAACGCTGGCGGCAGGCCTAACACATGCAAGTCGAACGGTAGCACAGAGGAGCTTGCTCCTTGGGTGACGAGTGGCGGACGGGTGAGTAATGTCTGGGAAACTGCCCGATGGAGGGGGATAACTACTGGAAACGGTAGCTAATACCGCATAACGTCGCAAGACCAAAGAGGGGGACCTTCGGGCCTCTTGCCATCGGATGTGCCCAGATGGGATTAGCTAGTAGGTGGGGTAACGGCTCACCTAGGCGACGATCCCTAGCTGGTCTGAGAGGATGACCAGCCACACTGGAACTGAGACACGGTCCAGACTCCTACGGGAGGCAGCAGTGGGGAATATTGCACAATGGGCGCAAGCCTGATGCAGCCATGCCGCGTGTATGAAGAAGGCCTTCGGGTTGTAAAGTACTTTCAGCGAGGAGGAAGGTGTTGAGGTTAATAACCTCAGCAATTGACGTTACTCGCAGAAGAAGCACCGGCTAACTCCGTGCCAGCAGCCGCGGTAA

+

::::::::::::::::::::::::::::::::::::::::::::::::::::::::::::::::::::::::::::::::::::::::::::::::::::::::::::::::::::::::::::::::::::::::::::::::::::::::::::::::::::::::::::::::::::::::::::::::::::::::::::::::::::::::::::::::::::::::::::::::::::::::::::::::::::::::::::::::::::::::::::::::::::::::::::::::::::::::::::::::::::::::::::::::::::::::::::::::::::::::::::::::::::::::::::::::::::::::::::::::::::::::::::::::::::::::::::::::::::::::::::::::::::::::::::::::::::::::::::::::::::::::::::::::::::::::::

@D0950.29_109997

GATGAACGCTAGCTACAGGCTTAACACATGCAAGTCGAGGGGTAGCATGAAACTTAGCAATAAGTTTTGATGACGACCGGCGCACGGGTGAGTAACACGTATCCAACCTGCCTTTTACTCATGGATAGCCTTCTGAAAAGAAGATTAATACATGATGGTATTCAGAGTTTTCATGGACACTGAATTAAAGATTTTATCGGTAAGAGATGGGGAATGCGTTCCATTAGATAGTAGGCTGGGGTAACGGCCCACCTAGTCGAACGATCGGATAGGGGTTCTGAGAGGAAGGTCCCCCACATTGGAACTGAGACACGGTCCAAACGTCCTACGGGAGGCAGCAGTGAGGAATATTGGTCAATGGACGTAAGTCTGAACCAGCCAAGTAGCGTGAAGGATGAAGGCTCTATGGGTCGTAAACTTCTTTTTATAAAAGGAATAAAGTATGCCACGTGTGGTGTTTTTGTATGTACTTTATGAATAAGGATCGGCTAACTCCGTGCCAGCAGCCGC

+

::::::::::::::::::::::::::::::::::::::::::::::::::::::::::::::::::::::::::::::::::::::::::::::::::::::::::::::::::::::::::::::::::::::::::::::::::::::::::::::::::::::::::::::::::::::::::::::::::::::::::::::::::::::::::::::::::::::::::::::::::::::::::::::::::::::::::::::::::::::::::::::::::::::::::::::::::::::::::::::::::::::::::::::::::::::::::::::::::::::::::::::::::::::::::::::::::::::::::::::::::::::::::::::::::::::::::::::::::::::::::::::::::::::::::::::::::::::::::::::::::::::::::::::::::::::::::::::

@D0950.29_110023

GATGAACGCTGGCGGCGTGCTTAACACATGCAAGTCGAACGAAGCACTTAAGGAGCTTGCTCCAAAGGTGACTGAGTGGCGGACGGGTGAGTAACGCGTGGGTAACCTGCCTTACACTGGGGGATAACAGTTGGAAACGACTGCTAATACCGCATAAGCGCACAGTATTGCATGATACAGTGTGAAAAACTCCGGTGGTGTAAGATGGACCCGCGTCTGATTAGCTAGTTGGTGAGGTAATGGCTCACCAAGGCAACGATCAGTAGCCGGCTTGAGAGAGTGAACGGCCACATTGGGACTGAGACACGGCCCAAACTCCTACGGGAGGCAGCAGTGGGGAATATTGCACAATGGGGGAAACCCTGATGCAGCAACGCCGCGTGAGTGAAGAAGTATTTCGGTATGTAAAGCTCTATCAGCAGGGAAGATAATGACGGTACCTGACTAAGAAGCCCCCGGCTAACTACGTGCCAGCAGCCGC

+

:::::::::::::::::::::::::::::::::::::::::::::::::::::::::::::::::::::::::::::::::::::::::::::::::::::::::::::::::::::::::::::::::::::::::::::::::::::::::::::::::::::::::::::::::::::::::::::::::::::::::::::::::::::::::::::::::::::::::::::::::::::::::::::::::::::::::::::::::::::::::::::::::::::::::::::::::::::::::::::::::::::::::::::::::::::::::::::::::::::::::::::::::::::::::::::::::::::::::::::::::::::::::::::::::::::::::::::::::::::::::::::::::::::::::::::::::::::::::::::::::

@D0950.29_110026

GATGAACGCTAGCTACAGGCTTAACACATGCAAGTCGAGGGGTAGCATGAAACTTAGCAATAAGTTTTGATGACGACCGGCGCACGGGTGAGTAACACGTATCCAACCTGCCTTTTACTCATGGATAGCCTTCTGAAAAGAAGATTAATACATGATGGTATTCAGAGTTTTCATGGACACTGAATTAAAGATTTTATCGGTAAGAGATGGGGATGCGTTCCATTAGATAGTAGGCGGGGTAACGGCCCACCTAGTCAACGATGGATAGGGGTTCTGAGAGGAAGGTCCCCCACATTGGAACTGAGACACGGTCCAAACGTCCTACGGGAGGCAGCAGTGAGGAATATTGGTCAATGGACGTAAGTCTGAACCAGCCAAGTAGCGTGAAGGATGAAGGCTCTATGGGTCGTAAACTTCTTTTTATAAAAGGGAATAAAGTATGCCACCGTGTGGTGTTTTTGTATGTACTTTATGAATAAGGATCGGCTAACTCCGTGCCAGCAGCCGC

+

::::::::::::::::::::::::::::::::::::::::::::::::::::::::::::::::::::::::::::::::::::::::::::::::::::::::::::::::::::::::::::::::::::::::::::::::::::::::::::::::::::::::::::::::::::::::::::::::::::::::::::::::::::::::::::::::::::::::::::::::::::::::::::::::::::::::::::::::::::::::::::::::::::::::::::::::::::::::::::::::::::::::::::::::::::::::::::::::::::::::::::::::::::::::::::::::::::::::::::::::::::::::::::::::::::::::::::::::::::::::::::::::::::::::::::::::::::::::::::::::::::::::::::::::::::::::::::

@D0950.29_11008

GATGAACGCTAGCGACAGGCCTAACACATGCAAGTCGAGGGGTAGCACAAGGTAGCAATACTGAGGTGACGACCGGCGCACGGGTGAGTAACGCGTATGCAACCTACCTGTAAGAGTGGGATAGCCTCTCGAAAGAGAGATTAATACCGCATAATACCATTTCACTGCATGGTGAGATGGTTAAAGATTTATTGCTTACAGATGGGCATGCGTAACATTAGCTAGTTGGTGAGGTAACGGCTCACCAAGGCAACGATGTTTAGGGGTTCTGAGAGGAAGGTCCCCCACACTGGTACTGAGACACGGACCAGACTCCTACGGGAGGCAGCAGTGAGGAATATTGGTCAATGGACGAGAGTCTGAACCAGCCAAGTCGCGTGAAGGATGAAGGTCTTATGGATTGTAAACTTCTTTTATACGGGAATAAAAAGAGCCACGTGTGGCTTATTGCATGTACCGTATGAATAAGGATCGGCTAACTCCGTGCCAGCAGCCGCGGTAA

+

::::::::::::::::::::::::::::::::::::::::::::::::::::::::::::::::::::::::::::::::::::::::::::::::::::::::::::::::::::::::::::::::::::::::::::::::::::::::::::::::::::::::::::::::::::::::::::::::::::::::::::::::::::::::::::::::::::::::::::::::::::::::::::::::::::::::::::::::::::::::::::::::::::::::::::::::::::::::::::::::::::::::::::::::::::::::::::::::::::::::::::::::::::::::::::::::::::::::::::::::::::::::::::::::::::::::::::::::::::::::::::::::::::::::::::::::::::::::::::::::::::::::::::::::::::::

@D0950.29_110080

GATGAACGCTGGCGGCGTGCTTAACACATGCAAGTCGAACGAAGCAACTTTCTTGCTTGCAAGAAAGTTGACTGAGTGGCGGACGGGTGAGTAACGCGTGGGTAACCTGCCTCATACAGGGGGATAACAGTTAGAAATGACTGCTAACACCGCATAACCCGCTAGTGTCGCATGACACGGACGGAAAATATTTATAGGTATGAGATGGGCCCGCGTCTGATTACGCTAGTTGGTGGGGTAACGGCCTACCAAGGCAACGATCAGTAGCCGACTTGAGAGAGTGATCGGCCACATTGGGGACTGAGACACGGCCCAAACTCCTACGGGAGGCAGCAGTGGGGAATATTGGACAATGGGGGAAACCCTGATCCAGCGACGCCGCGTGAGTGAAGAAGTATTTCGGTATGTAAAGCTCTATCAGCAGGGGAAAGATAAATGACAGTACCTGACTAAGAAGCCCCGGCTAACTACGTGCCAGCAGCCGCGGTAA

+

::::::::::::::::::::::::::::::::::::::::::::::::::::::::::::::::::::::::::::::::::::::::::::::::::::::::::::::::::::::::::::::::::::::::::::::::::::::::::::::::::::::::::::::::::::::::::::::::::::::::::::::::::::::::::::::::::::::::::::::::::::::::::::::::::::::::::::::::::::::::::::::::::::::::::::::::::::::::::::::::::::::::::::::::::::::::::::::::::::::::::::::::::::::::::::::::::::::::::::::::::::::::::::::::::::::::::::::::::::::::::::::::::::::::::::::::::::::::::::::::::::::::::

@D0950.29_110100

GATGAACGCTAGCGACAGGCCTAACACATGCAAGTCGAGGGGTAGCACAAGGTAGTAATACTGAGGTGACGACCGGCGCACGGGTGAGTAACGCGTATGCAACCTACCTGTAAGAGTGGGATAGCCTCTCGAAAGAGAGATTAATACCGCATAATACCATTTCACTGCATGGTGAGATGGTTAAAGATTTATTGCTTACAGATGGGCATGCGTAACATTAGCTAGTTGGTGAGGTAACGGCTCACCAAGGCAACGATGTTTAGGGGTTCTGAGAGGAAGGTCCCCCACACTGGTACTGAGACACGGACCAGACTCCTACGGGAGGCAGCAGTGAGGAATATTGGTCAATGGACGAGAGTCTGAACCAGCCAAGTCGCGTGAAGGATGAAGGTCTTATGGATTGTAAACTTCTTTTATACGGGAATAAAAATGCCACGTGTGGCATATTGCATGTACCGTATGAATAAGGATCGGCTAACTCCGTGCCAGCAGCCGCGGTAA

+

:::::::::::::::::::::::::::::::::::::::::::::::::::::::::::::::::::::::::::::::::::::::::::::::::::::::::::::::::::::::::::::::::::::::::::::::::::::::::::::::::::::::::::::::::::::::::::::::::::::::::::::::::::::::::::::::::::::::::::::::::::::::::::::::::::::::::::::::::::::::::::::::::::::::::::::::::::::::::::::::::::::::::::::::::::::::::::::::::::::::::::::::::::::::::::::::::::::::::::::::::::::::::::::::::::::::::::::::::::::::::::::::::::::::::::::::::::::::::::::::::::::::::::::::::::::

@D0950.29_110225

ATTGAACGCTGGCGGCATGCCTTACACATGCAAGTCGAACGGTAACAGGTCTTCGGATGCTGACGAGTGGCGAACGGGTGAGTAATACATCGGAACGTGCCCGATCGTGGGGGATAACGAAGCGAAAGCTTTGCTAATACCGCATACGATCTACGGATGAAAGCAGGGGACCGCAAGGCCTTGCGCGAACGGAGCGGCCGATGGCAGATTAGGTAGTTGGTGGGATAAAAGCTTACCAAGCCGACGATCTGTAGCTGGTCTGAGAGGACGACCAGCCACACTGGGACTGAGACACGGCCCAGACTCCTACGGGAGGCAGCAGTGGGGAATTTTGGACAATGGGCGAAAGCCTGATCCAGCCATGCCGCGTGCAGGATGAAGGCCTTCGGGTTGTAAACTGCTTTTGTACGGAACGAAAAGACTCTGGTTAATACCTGGGGTCCATGACGGTACCGTAAGAATAAGCACCGGCTAACTACGTGCCAGCAGCCGCGGTAA

+

::::::::::::::::::::::::::::::::::::::::::::::::::::::::::::::::::::::::::::::::::::::::::::::::::::::::::::::::::::::::::::::::::::::::::::::::::::::::::::::::::::::::::::::::::::::::::::::::::::::::::::::::::::::::::::::::::::::::::::::::::::::::::::::::::::::::::::::::::::::::::::::::::::::::::::::::::::::::::::::::::::::::::::::::::::::::::::::::::::::::::::::::::::::::::::::::::::::::::::::::::::::::::::::::::::::::::::::::::::::::::::::::::::::::::::::::::::::::::::::::::::::::::::::::::

@D0950.29_110314

ATTGAACGCTGGCGGCAGGCCTAACACATGCAAGTCGAACGGTAGCACAGAGAGCTTGCTCTTGGGTGACGAGTGGCGGACGGGTGAGTAATGTCTGGGAAACTGCCCGATGGAGGGGGATAACTACTGGAAACGGTAGCTAATACCGCATAACGTCGCAAGACCAAAGAGGGGGACCTTCGGGCCTCTTGCCATCGGATGTGCCCAGATGGGATTAGCTAGTAGGTGGGGTAACGGCTCACCTAGGCGACGATCCCTAGCTGGTCTGAGAGGATGACCAGCCACACTGGAACTGAGACACGGTCCAGACTCCTACGGGAGGCAGCAGTGGGGAATATTGCACAATGGGCGCAAGCCTGATGCAGCCATGCCGCGTGTATGAAGAAGGCCTTCGGGTTGTAAAGTACTTTCAGCGAGGAGGAAGGCGGTGAGGTTAATAACCTCATCGATTGACGTTACTCGCAGAAGAAGCACCGGCTAACTCCGTGCCAGCAGCCGCGGTAA

+

::::::::::::::::::::::::::::::::::::::::::::::::::::::::::::::::::::::::::::::::::::::::::::::::::::::::::::::::::::::::::::::::::::::::::::::::::::::::::::::::::::::::::::::::::::::::::::::::::::::::::::::::::::::::::::::::::::::::::::::::::::::::::::::::::::::::::::::::::::::::::::::::::::::::::::::::::::::::::::::::::::::::::::::::::::::::::::::::::::::::::::::::::::::::::::::::::::::::::::::::::::::::::::::::::::::::::::::::::::::::::::::::::::::::::::::::::::::::::::::::::::::::::::::::::::::::

@D0950.29_110338

GATGAACGCTGGCGGCGTGCTTAACACATGCAAGTCGAACGAAGCACTTTGAAGAGCTTGCTCTTTAAAGTGACTGAGTGGCGGACGGGTGAGTAACGCGTGGGTAACCTGCCTCATACAGGGGGATAACAGTTAGAAATGACTGCTAACACCGCATAACCCGCTAGCATCGCATGATGCAGACGGAAAAATATTTATAGGTATGAGATGGGCCCGCGTCTGATTAGCTAGTTGGTGGGGTAACGGCCTACCAAGGCAACGATCAGTAGCCGACTTGAGAGAGTGATCGGCCACATTGGGACTGAGACACGGCCCAAACTCCTACGGGAGGCAGCAGTGGGGAATATTGGACAATGGGGGAAACCCTGATCCAGCGACGCCGCGTGAGTGAAAGAAGTATTTTCGGTATGTAAAAGCTCTATCAGCAGGGGAAAGATGATGACAGTACCTGACTAAAGAAGCCCCGGCTAACTACGTGCCAGCAGCCGCGGTAA

+

::::::::::::::::::::::::::::::::::::::::::::::::::::::::::::::::::::::::::::::::::::::::::::::::::::::::::::::::::::::::::::::::::::::::::::::::::::::::::::::::::::::::::::::::::::::::::::::::::::::::::::::::::::::::::::::::::::::::::::::::::::::::::::::::::::::::::::::::::::::::::::::::::::::::::::::::::::::::::::::::::::::::::::::::::::::::::::::::::::::::::::::::::::::::::::::::::::::::::::::::::::::::::::::::::::::::::::::::::::::::::::::::::::::::::::::::::::::::::::::::::::::::::::::

@D0950.29_11039

GATGAACGCTGGCGGCGTGCTTAACACATGCAAGTCGAACGAAGCACTTAACTTAGAATCTTCGGAGGAAGAGTTTTGTGACTTAGTGGCGGACGGGTGAGTAACGCGTGGGTAACCTGCCTTATACTGGGGGATAACAGTTAGAAATGACTGCTAATACCGCATAAGCGCACAGTATCACATGATACAGTGCGAAAAACTCCGGTGGTATAAGATGGACCCGCGTCTGATTAGCTAGTTGGTAAGGTAACGGCTTACCAAGGCGACGATCAGTAGCCGACTTGAGAGAGTGATCGGCCACATTGGGACTGAGACACGGCCCAAACTCCTACGGGAGGCAGCAGTGGGGAATATTGCACAATGGGCGAAAGCCTGATGCAGCGACGCCGCGTGAAGGATGAAGTATTTCGGTACGTAAACTTCTATCAGCAAGGAAGATAATGACGGTACTTGACTAAGAAGCCCCCGGCTAACTACGTGCCAGCACCGC

+

::::::::::::::::::::::::::::::::::::::::::::::::::::::::::::::::::::::::::::::::::::::::::::::::::::::::::::::::::::::::::::::::::::::::::::::::::::::::::::::::::::::::::::::::::::::::::::::::::::::::::::::::::::::::::::::::::::::::::::::::::::::::::::::::::::::::::::::::::::::::::::::::::::::::::::::::::::::::::::::::::::::::::::::::::::::::::::::::::::::::::::::::::::::::::::::::::::::::::::::::::::::::::::::::::::::::::::::::::::::::::::::::::::::::::::::::::::::::::::::::::::::::::

@D0950.29_1104

GATGAACGCTAGCTACAGGCTTAACACATGCAAGTCGAGGGGTAGCAGGGTAGCAATACCGCTGACGACCGGCGCACGGGTGAGTAACACGTATCCAACCTACCATTCACACGGGGATAGCCTTTCGAAAGAAAGATTAATACCCGATCGTATAGCGAGAGGGCATCCTTTTGCTATTAAAGAATTTCGGTGTTTGATGGGGATGCGTTCCATTAGATTGTTGGTGAGGTAACGGCTCACCAAGTCTTCGATGGATAGGGGTTCTGAGAGGAAGGTCCCCCACATTGGAACTGAGACACGGTCCAAACTCCTACGGGAGGCAGCAGTGAGGAATATTGGTCAATGGACGAGAGTCTGAACCAGCCAAGTAGCGTGAAGGATGAAGGCCCCTATGGGTCGTAAACTTCTTTTATAGGGGAATAAACGTGCGGAACGTGTTCCGCTTTGTATGTACCCTACGAATAAGGATCGGCTAACTCCGTGCCAGCAGCCGCGGTAA

+

:::::::::::::::::::::::::::::::::::::::::::::::::::::::::::::::::::::::::::::::::::::::::::::::::::::::::::::::::::::::::::::::::::::::::::::::::::::::::::::::::::::::::::::::::::::::::::::::::::::::::::::::::::::::::::::::::::::::::::::::::::::::::::::::::::::::::::::::::::::::::::::::::::::::::::::::::::::::::::::::::::::::::::::::::::::::::::::::::::::::::::::::::::::::::::::::::::::::::::::::::::::::::::::::::::::::::::::::::::::::::::::::::::::::::::::::::::::::::::::::::::::::::::::::::::

@D0950.29_110404

GATGAACGCTAGCTACAGGCTTAACACATGCAAGTCGAGGGGTAGCATGAAACTTAGCAATAAGTTTTGATGACGACCGGCGCACGGGTGAGTAACACGTATCCAACCTGCCTTTTACTCATGGATAGCCTTCTGAAAAGAAGATTAATACATGATGGTATTCAGAGTTTTCATGGACACTGAATTAAAGATTTATCGGTAAGAGATGGGGATGCGTTCCATTAGATAGTAGGCGGGGTAACGGCCCACCTAGTCAACATGGATAGGGGTTCTGAGAGGAAGGTCCCCCACATTGGAATCTGAGGACACGGTCCAAACTCCTACGGGAGGCCAGCAGTGAGGAATATTGGTCAATGGACGTAAGTCTGAACCAGCCAAGTAGCGTGAA

+

::::::::::::::::::::::::::::::::::::::::::::::::::::::::::::::::::::::::::::::::::::::::::::::::::::::::::::::::::::::::::::::::::::::::::::::::::::::::::::::::::::::::::::::::::::::::::::::::::::::::::::::::::::::::::::::::::::::::::::::::::::::::::::::::::::::::::::::::::::::::::::::::::::::::::::::::::::::::::::::::::::::::::::::::::::::::::::::::::::::::::::::::::::::::::::::::::::

@D0950.29_110415

GATGAACGCTGGCGGCGTGCTTAACACATGCAAGTCGAACGAAGCACTTAAGGAGCTTGCTCCAAAGTGACTGAGTGGCGGACGGGTGAGTAACGCGTGGGTAACCTGCCTTACACTGGGGGATAACAGTTGGAAACGACTGCTAATACCGCATAAGCGCACAGTATTGCATGATACAGTGTGAAAAACTCCGGTGGTGTAAGATGGACCCGCGTCTGATTAGCTAGTTGGTGAGGTAATGGCTCACCAAGGCAACGATCAGTAGCCGGCTTGAGAGAGTGAACGGCCACATTGGGACTGAGACACGGCCCAAACTCCTACGGGAGGCAGCAGTGGGAATATTGCACAATGGGGGAAACCCTGATGCAGCAACGCCGCGTGAGTGAAGAAGTATTTCGGTATGTAAAGCTCTATCAGCAGGAAGATAATGACGGTACCTGACTAAGAAGCCCCCGGCTAACTACGTGCCAGCAGCCGC

+

::::::::::::::::::::::::::::::::::::::::::::::::::::::::::::::::::::::::::::::::::::::::::::::::::::::::::::::::::::::::::::::::::::::::::::::::::::::::::::::::::::::::::::::::::::::::::::::::::::::::::::::::::::::::::::::::::::::::::::::::::::::::::::::::::::::::::::::::::::::::::::::::::::::::::::::::::::::::::::::::::::::::::::::::::::::::::::::::::::::::::::::::::::::::::::::::::::::::::::::::::::::::::::::::::::::::::::::::::::::::::::::::::::::::::::::::::::::::::::::

@D0950.29_110483

GATGAACGCTAGCTACAGGCTTAACACATGCAAGTCGAGGGGTAGCATGAAACTTAGCAATAAGTTTTGATGACGACCGGCGCACGGGTGAGTAACACGTATCCAACCTGCCTTTTACTCATGGATAGCCTTCTGAAAAGAAGATTAATACATGATGGTATTCAGAGTTTTCATGGACACTGAATTAAAGATTTTATCGGTAAGAGATGGGGATGCGTTCCATTAGATAGTAGGCGGGGTAACGGCCCACCTAGTCAACGATGGATAGGGGTTCTGAGAGGAAGGTCCCCCACATTGGAACTGAGACACGGTCCAAACTCCTACGGGAGGCAGCAGTGAGGAATATTGGTCAATGGACGTAAGTCTGAACCAGCCAAGTAGCGTGAAGGATGAAGGCTCTATGGGTCGTAAACTTCTTTTTATAAAAGGAATAAAGATGCCACGTGTGGTGTTTTGTATGTACTTTATGAATAAGGATCGGCTAAACTCCGTGCCAGCAGCCGC

+

::::::::::::::::::::::::::::::::::::::::::::::::::::::::::::::::::::::::::::::::::::::::::::::::::::::::::::::::::::::::::::::::::::::::::::::::::::::::::::::::::::::::::::::::::::::::::::::::::::::::::::::::::::::::::::::::::::::::::::::::::::::::::::::::::::::::::::::::::::::::::::::::::::::::::::::::::::::::::::::::::::::::::::::::::::::::::::::::::::::::::::::::::::::::::::::::::::::::::::::::::::::::::::::::::::::::::::::::::::::::::::::::::::::::::::::::::::::::::::::::::::::::::::::::::::::::

@D0950.29_110492

GATGAACGCTGGCGGCGTGCTTAACACATGCAAGTCGAACGAAGCACTTTGAAGAGCTTGCTCTTTAAAGTGACTGAGTGGCGGACGGGTGAGTAACGCGTGGGTAACCTGCCTCATACAGGGGGATAACAGTTAGAAATGACTGCTAACACCGCATAACCCGCTAGTGTCGCATGACACAGACGGAAAATATTTATAGGTATGAGATGGGCCCGCGTCTGATTAGCTAGTTGGTGGGGTAACGGCCTACCAAGGCAACGATCAGTAGCCGACTTGAGAGAGTGATCGGCCACATTGGGACTGAGACACGGCCCAAACTCCTACGGGAGGCAGCAGTGGGGAATATTGGACAATGGGGGAAACCCTGATCCAGCGACGCCGCGTGAGTGAAGAAGTATTTCGGTATGTAAAGCTCTATCAGCAGGGGAAGATAATGACAGTACCTGACTAAGAAGCCCCGGCTAACTACGTGCCAGCAGCCGCGGTAA

+

::::::::::::::::::::::::::::::::::::::::::::::::::::::::::::::::::::::::::::::::::::::::::::::::::::::::::::::::::::::::::::::::::::::::::::::::::::::::::::::::::::::::::::::::::::::::::::::::::::::::::::::::::::::::::::::::::::::::::::::::::::::::::::::::::::::::::::::::::::::::::::::::::::::::::::::::::::::::::::::::::::::::::::::::::::::::::::::::::::::::::::::::::::::::::::::::::::::::::::::::::::::::::::::::::::::::::::::::::::::::::::::::::::::::::::::::::::::::::::::::::::::::

@D0950.29_110494

GATGAACGCTAGCTACAGGCTTAACACATGCAAGTCGAGGGGTAGCATGAAACTTAGCAATAAGTTTTGATGACGACCGGCGCACGGGTGAGTAACACGTATCCAACCTGCCTTTTACTCATGGATAGCCTTCTGAAAAGAAGATTAATACATGATGGTATTCAGAGTTTTCATGGACACTGAATTAAAGATTTTATCGGTAAGAGATGGGGATGCGTTCCATTAGATAGTAGGCGGGGTAACGGCCCACCTAGTCAACGATGGATAGGGGTTCTGAGAGGAAGGTCCCCCACATTGGAACTGAGACACGGTCCAAACGTCCTACGGGAGGCAGCAGTGAGGAATATTGGTCAATGGACGTAAGTCTGAACCAGCCAAGTAGCGTGAAGGATGAAGGCTCTATGGGTCGTAAACTTCTTTTTATAAAA

+

::::::::::::::::::::::::::::::::::::::::::::::::::::::::::::::::::::::::::::::::::::::::::::::::::::::::::::::::::::::::::::::::::::::::::::::::::::::::::::::::::::::::::::::::::::::::::::::::::::::::::::::::::::::::::::::::::::::::::::::::::::::::::::::::::::::::::::::::::::::::::::::::::::::::::::::::::::::::::::::::::::::::::::::::::::::::::::::::::::::::::::::::::::::::::::::::::::::::::::::::::::::::::::::::::::::::::::

@D0950.29_110498

GATGAACGCTAGCGACAGGCCTAACACATGCAAGTCGAGGGGTAGCACAAGGTAGCAATACTGAGGTGACGACCGGCGCACGGGTGAGTAACGCGTATGCAACCTACCTGTAAGAGTGGGATAGCCTCTCGAAAGAGAGATTAATACCGCATAATACCATTTCACTGCATGGTGAGATGGTTAAAGATTTATTGCTTACAGATGGGCATGCGTAACATTAGCTAGTTGGTGAGGTAACGGCTCACCAAGGCAACGATGTTTAGGGGTTCTGAGAGGAAGGTCCCCCACACTGGTACTGAGACACGGACCAGACTCCTACGGGAGGCAGCAGTGAGGAATATTGGTCAATGGACGAGAGTCTGAACCAGCCAAGTCGCGTGAAGGATGAAGGTCTTATGGATTGTAAACTTCTTTTATACGGGAATAAAAATGCCACGTGTGGCATATTGCATGTACCGTATGAATAAGGATCGGCTAACTCCGTGCCAGCAGCCGCGGTAA

+

:::::::::::::::::::::::::::::::::::::::::::::::::::::::::::::::::::::::::::::::::::::::::::::::::::::::::::::::::::::::::::::::::::::::::::::::::::::::::::::::::::::::::::::::::::::::::::::::::::::::::::::::::::::::::::::::::::::::::::::::::::::::::::::::::::::::::::::::::::::::::::::::::::::::::::::::::::::::::::::::::::::::::::::::::::::::::::::::::::::::::::::::::::::::::::::::::::::::::::::::::::::::::::::::::::::::::::::::::::::::::::::::::::::::::::::::::::::::::::::::::::::::::::::::::::::

@D0950.29_110500

ATTGAACGCTGGCGGCAGGCCTAACACATGCAAGTCGAACGGTAGCACAGAGAGCTTGCTCTTGGGTGACGAGTGGCGGACGGGTGAGTAATGTCTGGGAAACTGCCCGATGGAGGGGGATAACTACTGGAAACGGTAGCTAATACCGCATAACGTCGCAAGACCAAAGAGGGGGACCTTCGGGCCTCTTGCCATCGGATGTGCCCAGATGGGATTAGCTAGTAGGTGGGGTAACGGCTCACCTAGGCGACGATCCCTAGCTGGTCTGAGAGGATGACCAGCCACACTGGAACTGAGACACGGTCCAGACTCCTACGGGAGGCAGCAGTGGGGAATATTGCACAATGGGCGCAAGCCTGATGCAGCCATGCCGCGTGTATGAAGAAGGCCTTCGGGTTGTAAAGTACTTTCAGCGAGGAGGAAGGCGGTGAGGTTAATAACCTCATCGATTGACGTTACTCGCAGAAGAAGCACCGGCTAACTCCGTGCCAGCAGCCGCGGTAA

+

::::::::::::::::::::::::::::::::::::::::::::::::::::::::::::::::::::::::::::::::::::::::::::::::::::::::::::::::::::::::::::::::::::::::::::::::::::::::::::::::::::::::::::::::::::::::::::::::::::::::::::::::::::::::::::::::::::::::::::::::::::::::::::::::::::::::::::::::::::::::::::::::::::::::::::::::::::::::::::::::::::::::::::::::::::::::::::::::::::::::::::::::::::::::::::::::::::::::::::::::::::::::::::::::::::::::::::::::::::::::::::::::::::::::::::::::::::::::::::::::::::::::::::::::::::::::

@D0950.29_110596

GATGAACGCTGGCGGCGTGCTTAACACATGCAAGTCGAACGAAGCACTTAAGGAGCTTGCTCCAAAAGTGACTGAGTGGCGGACGGGTGAGTAACGCGTGGGTAACCTGCCTTACACTGGGGGATAACAGTTGGAAACGACTGCTAATACCGCATAAGCGCACAGTATTGCATGATACAGTGTGAAAAACTCCGGTGGTGTAAGATGGACCCGCGTCTGATTAGCTAGTTGGTGAGGTAATGGCTCACCAAGGCAACGATCAGTAGCCGGCTTGAGAGAGTGAACGGCCACATTGGGACTGAGACACGGCCCAAACTCCTACGGGAGGCAGCAGTGGGGAATATTGCACAATGGGGGAAACCCTGATGCAGCAACGCCGCGTGAGTGAAGAAGTATTTGCGGTATGTAAAGCTCTATCAGCAGGGAAGATAATGACGGTACCTGACTAAGAAGCCCCCGGCTAACTACGTGCCAGCAGCCGC

+

::::::::::::::::::::::::::::::::::::::::::::::::::::::::::::::::::::::::::::::::::::::::::::::::::::::::::::::::::::::::::::::::::::::::::::::::::::::::::::::::::::::::::::::::::::::::::::::::::::::::::::::::::::::::::::::::::::::::::::::::::::::::::::::::::::::::::::::::::::::::::::::::::::::::::::::::::::::::::::::::::::::::::::::::::::::::::::::::::::::::::::::::::::::::::::::::::::::::::::::::::::::::::::::::::::::::::::::::::::::::::::::::::::::::::::::::::::::::::::::::::

@D0950.29_110601

GATGAACGCTAGCTACAGGCTTAACACATGCAAGTCGAGGGGTAGCATGAAACTTAGCAATAAGTTTTGATGACGACCGGCGCACGGGTGAGTAACACGTATCCAACCTGCCTTTTACTCATGGATAGCCTTCTGAAAAGAAGATTAATACATGATGGTATTCAGAGTTTTCATGGACACTGAATTAAAGATTTTATCGGTAAGAGATGGGGATGCGTTCCATTAGATAGTAGGCGGGGTAACGGCCCACCTAGTCAACGATGGATAGGGGTTCTGAGAGGAAGGTCCCCCACATTGGAACTGAGACACGGTCCAAACGTCCTACGGGAGGCAGCAGTGAGGAATATTGGTCAATGGACGTAAGTCTGAACCAGCCAAGTAGCGTGAAGGATGAAGGCTCTATGGGTCGTAAACTTCTTTTTATAAAAGGAATAAAGTATGCCACGTGTGGTGTTTTTGTATGTAACTTTATGAATAAGGATCGGCTAACTCCGTGCCAGCAGCCGC

+

:::::::::::::::::::::::::::::::::::::::::::::::::::::::::::::::::::::::::::::::::::::::::::::::::::::::::::::::::::::::::::::::::::::::::::::::::::::::::::::::::::::::::::::::::::::::::::::::::::::::::::::::::::::::::::::::::::::::::::::::::::::::::::::::::::::::::::::::::::::::::::::::::::::::::::::::::::::::::::::::::::::::::::::::::::::::::::::::::::::::::::::::::::::::::::::::::::::::::::::::::::::::::::::::::::::::::::::::::::::::::::::::::::::::::::::::::::::::::::::::::::::::::::::::::::::::::::

@D0950.29_110711

GATGAACGCTGGCGGCGTGCTTAACACATGCAAGTCGAGCGAGGAATCACCTTCGGGTGTGAACTAGCGGCGGACGGGTGAGTAACACGTGGGCAACCTGCCTTACAGAGGGGGATAGCCTTCCGAAAGGAAGATTAATACCGCATATTATGAGTTTTCTGCATGGGGAATTCATGAAAGGAGTAATCCGCTGTAAGATGGGCCCGCGGCGCATTAGCTAGTTGGTGAGGTAACGGCTCACCAAGGCGACGATGCGTAGCCGACCTGAGAGGGTGATCGGCCACATTGGGACTGAGACACGGCCCAGACTCCTACGGGAGGCAGCAGTGGGGAATATTGCACAATGGGGGAAACCCTGATGCAGCAACGCCGCGTGAGTGATGAAGGCCTTCGGGTTGTAAAGCTCTGTCTTCA

+

::::::::::::::::::::::::::::::::::::::::::::::::::::::::::::::::::::::::::::::::::::::::::::::::::::::::::::::::::::::::::::::::::::::::::::::::::::::::::::::::::::::::::::::::::::::::::::::::::::::::::::::::::::::::::::::::::::::::::::::::::::::::::::::::::::::::::::::::::::::::::::::::::::::::::::::::::::::::::::::::::::::::::::::::::::::::::::::::::::::::::::::::::::::::::::::::::::::::::::::::::::::::::::::

@D0950.29_110719

GATGAACGCTGGCGGCGTGCTTAACACATGCAAGTCGAACGAAGCACTTTGAAGAGCTTGCTCTTTAAAGTGACTGAGTGGCGGACGGGTGAGTAACGCGTGGGTAACCTGCCTCATACAGGGGGATAACAGTTAGAAATGACTGCTAACACCGCATAACCCGCTAGTGTCGCATGACACAGACGGAAAATATTTATAGGTATGAGATGGGCCCGCGTCTGATTAGCTAGTTGGTGGGGTAACGGCCTACCAAGGCAACGATCAGTAGCCGACTTGAGAGAGTGATCGGCCACATTGGGACTGAGACACGGCCCAAACTCCTACGGGAGGCAGCAGTGGGGAATATTGGACAATGGGGGAAACCCTGATCCAGCGACGCCGCGTGAGTGAAGAAGTATTTCGGTATGTAAAGCTCTATCAGCAGGGGAAGATAATGACAGTACCTGACTAAGAAGCCCCGGCTAACTACGTGCCAGCAGCCGCGGTAA

+

::::::::::::::::::::::::::::::::::::::::::::::::::::::::::::::::::::::::::::::::::::::::::::::::::::::::::::::::::::::::::::::::::::::::::::::::::::::::::::::::::::::::::::::::::::::::::::::::::::::::::::::::::::::::::::::::::::::::::::::::::::::::::::::::::::::::::::::::::::::::::::::::::::::::::::::::::::::::::::::::::::::::::::::::::::::::::::::::::::::::::::::::::::::::::::::::::::::::::::::::::::::::::::::::::::::::::::::::::::::::::::::::::::::::::::::::::::::::::::::::::::::::

@D0950.29_110773

GATGAACGCTAGCGACAGGCCTAACACATGCAAGTCGAGGGGTAGCACAAGGTAGCAATACTGAGGTGACGACCGGCGCACGGGTGAGTAACGCGTATGCAACCTACCTGTAAGAGTGGGATAGCCTCTCGAAAGAGAGATTAATACCGCATAATACCATTTCACTGCATGGTGAGATGGTTAAAGATTTATTGCTTACAGATGGGCATGCGTAACATTAGCTAGTTGGTGAGGTAACGGCTCACCAAGGCAACGATGTTTAGGGGTTCTGAGAGGAAGGTCCCCCACACTGGTACTGAGACACGGACCAGACTCCTACGGGAGGCAGCAGTGAGGAATATTGGTCAATGGACGAGAGTCTGAACCAGCCAAAGTCGCGTGAAGGATGAAGGTCTTATGGATTGTAAACTTCTTTTATACGGGAATAAAAAATGCCACGTGTGGCATATTGCATGTACCGTATGAATAAGGATCGGCT

+

::::::::::::::::::::::::::::::::::::::::::::::::::::::::::::::::::::::::::::::::::::::::::::::::::::::::::::::::::::::::::::::::::::::::::::::::::::::::::::::::::::::::::::::::::::::::::::::::::::::::::::::::::::::::::::::::::::::::::::::::::::::::::::::::::::::::::::::::::::::::::::::::::::::::::::::::::::::::::::::::::::::::::::::::::::::::::::::::::::::::::::::::::::::::::::::::::::::::::::::::::::::::::::::::::::::::::::::::::::::::::::::::::::::::::::::::::::::::::::::

@D0950.29_110931

GATGAACGCTAGCGACAGGCCTAACACATGCAAGTCGAGGGGTAGCACAAGGAAGCTTGCTTCTGAGGTGACGACCGGCGCACGGGTGAGTAACGCGTATGCAACCTGCCTATAAGAAGGGGATAGCCTCTCGAAAGAGAGATTAATACCGTATAACACTATGAAGCCGCATGGTTTTATAGTTAAAGATTTATTGCTTATAGATGGGCATGCGTAACATTAGCTAGTTGGTGAGGTAACGGCTCACCAAGGCAACGATGTTTAGGGGTTCTGAGAGGAAGGTCCCCCACACTGGTACTGAGACACGGACCAGACTCCTACGGGAGGCAGCAGTGAGGAATATTGGTCAATGGACGAGAGTCTGAACCAGCCAAGTCGCGTGAAGGAAGAAGGTTCTATGGATTGTAAACTTCTTTTATAGGGGAATAAAGTGAGGAACGTGTTCCTTTTTGTATGTACCCTATGAATAAGCATCGGCTAACTCCGTGCCAGCAGCCGCGGTAA

+

::::::::::::::::::::::::::::::::::::::::::::::::::::::::::::::::::::::::::::::::::::::::::::::::::::::::::::::::::::::::::::::::::::::::::::::::::::::::::::::::::::::::::::::::::::::::::::::::::::::::::::::::::::::::::::::::::::::::::::::::::::::::::::::::::::::::::::::::::::::::::::::::::::::::::::::::::::::::::::::::::::::::::::::::::::::::::::::::::::::::::::::::::::::::::::::::::::::::::::::::::::::::::::::::::::::::::::::::::::::::::::::::::::::::::::::::::::::::::::::::::::::::::::::::::::::::

@D0950.29_110933

ATTGAACGCTGGCGGCAGGCCTAACACATGCAAGTCGAACGGTAGCACAGAGGAGCTTGCTCCTTGGGTGACGAGTGGCGGACGGGTGAGTAATGTCTGGGAAACTGCCCGATGGAGGGGGATAACTACTGGAAACGGTAGCTAATACCGCATAACGTCGCAAGACCAAAGAGGGGGACCTTCGGGGCCTCTTGCCATCGGATGTGCCCAGATGGGATTAGCTAGTAGGTGGGGTAACGGCTCACCTAGGCCGACGATCCCTAGCTGGTCTGAGAGGATGACCAGCCACGACTGGAACTGAGACACGGTCCAGACTCCTACGGGAGGCAGCAGTGGGGAATATTGCACAATGGGCGCAAGCCTGATGCAGCCATGCCGCGTGTATGAAGAAGGCCTTCGGGTTGTAAATACTTTCAGCGAGGAGGAAGGTGTTGAGGTTAATAACCTGAGCAATTGACGTTACTCGCAGAAGAAGCACCGGCTAACTCCGTGCCAGCAGCCGCGGT

+

::::::::::::::::::::::::::::::::::::::::::::::::::::::::::::::::::::::::::::::::::::::::::::::::::::::::::::::::::::::::::::::::::::::::::::::::::::::::::::::::::::::::::::::::::::::::::::::::::::::::::::::::::::::::::::::::::::::::::::::::::::::::::::::::::::::::::::::::::::::::::::::::::::::::::::::::::::::::::::::::::::::::::::::::::::::::::::::::::::::::::::::::::::::::::::::::::::::::::::::::::::::::::::::::::::::::::::::::::::::::::::::::::::::::::::::::::::::::::::::::::::::::::::::::::::::::::

@D0950.29_110943

GATGAACGCTAGCGACAGGCCTAACACATGCAAGTCGAGGGGTAGCACAAGGAAGCTTGCTTCTGAGGTGACGACCGGCGCACGGGTGAGTAACGCGTATGCAACCTACCTGTAAGAGTGGGATAGCCTCTCGAAAGAGAGATTAATACCGCATAATACCATTTCACTGCATGGTGAGATGGTTAAAGATTTATTGCTTACAGATGGGCATGCGTAACATTAGCTAGTTGGTGAGGTAACGGCTCACCAAGGCAACGATGTTTAGGGGTTCTGAGAGGAAGGTCCCCCACACTGGTACTGAGACACGGGACCAGACTCCTACGGGAGGCAGCAGTGAGGAATATTGGTCAATGGACGAGAGTCTGAACCAGCCAAGTCGCGTGAAGGATGAAGGTCTTATGGATTGTAAACTTCTTTTATACGGGAATAAAAATGCCACGTGTGGCATATTGCATGTACCGTATGAATAAGGATCGGCTAACTCCGTGCCAGCAGCCGCGGTAA

+

::::::::::::::::::::::::::::::::::::::::::::::::::::::::::::::::::::::::::::::::::::::::::::::::::::::::::::::::::::::::::::::::::::::::::::::::::::::::::::::::::::::::::::::::::::::::::::::::::::::::::::::::::::::::::::::::::::::::::::::::::::::::::::::::::::::::::::::::::::::::::::::::::::::::::::::::::::::::::::::::::::::::::::::::::::::::::::::::::::::::::::::::::::::::::::::::::::::::::::::::::::::::::::::::::::::::::::::::::::::::::::::::::::::::::::::::::::::::::::::::::::::::::::::::::::::::

@D0950.29_110958

GATGAACGCTAGCGACAGGCCTAACACATGCAAGTCGAGGGGTAGCACAAGGAAGCTTGCTTCTGAGGTGACGACCGGCGCACGGGTGAGTAACGCGTATGCAACCTACCTGTAAGAGTGGGATAGCCTCTCGAAAGAGAGATTAATACCGCATAATACCATTTCACTGCATGGTGAGATGGTTAAAGATTTATTGCTTACAGATGGGCATGCGTAACATTAGCTAGTTGGTGAGGTAACGGCTCACCAAGGCAACGATGTTTAGGGGTTCTGAGAGGAAGGTCCCCCACACTGGTACTGAGACACGGACCAGACTCCTACGGGAGGCAGCAGTGAGGAATATTGGTCAATGGACGAGAGTCTGAACCAGCCAAGTCGCGTGAAGGATGAAGGTCTTATGGATTGTAAACTTCTTTTATACGGGAATAAAAAGAGCCACGTGTGGCTTATTGCATGTACCGTATGAATAAGGATCGGCT

+

:::::::::::::::::::::::::::::::::::::::::::::::::::::::::::::::::::::::::::::::::::::::::::::::::::::::::::::::::::::::::::::::::::::::::::::::::::::::::::::::::::::::::::::::::::::::::::::::::::::::::::::::::::::::::::::::::::::::::::::::::::::::::::::::::::::::::::::::::::::::::::::::::::::::::::::::::::::::::::::::::::::::::::::::::::::::::::::::::::::::::::::::::::::::::::::::::::::::::::::::::::::::::::::::::::::::::::::::::::::::::::::::::::::::::::::::::::::::::::::::

@D0950.29_110997

GATGAACGCTAGCTACAGGCTTAACACATGCAAGTCGAGGGGTAGCATGAAACTTAGCAATAAGTTTTGATGACGACCGGCGCACGGGTGAGTAACACGTATCCAACCTGCCTTTTACTCATGGATAGCCTTCTGAAAAGAAGATTAATACATGATGGTATTCAGAGTTTTCATGGACACTGAATTAAAGATTTTATCGGTAAGAGATGGGGATGCGTTCCATTAGATAGTAGGCGGGGTAACGGCCCACCTAGTCAACGATGGATAGGGGTTCTGAGAGGAAGGTCCCCCACATTGGAACTGAGACACGGTCCAAACGTCCTACGGGAGGCAGCAGTGAGGAATATTGGTCAATGGACGTAAGTCTGAACCAGCCAAGTAGCGTGAAGGATGAAGGCTCTATGGGTCGTAAACTTCTTTTTATAAAAGG

+

::::::::::::::::::::::::::::::::::::::::::::::::::::::::::::::::::::::::::::::::::::::::::::::::::::::::::::::::::::::::::::::::::::::::::::::::::::::::::::::::::::::::::::::::::::::::::::::::::::::::::::::::::::::::::::::::::::::::::::::::::::::::::::::::::::::::::::::::::::::::::::::::::::::::::::::::::::::::::::::::::::::::::::::::::::::::::::::::::::::::::::::::::::::::::::::::::::::::::::::::::::::::::::::::::::::::::::::

@D0950.29_111013

GATGAACGCTAGCGACAGGCCTAACACATGCAAGTCGAGGGGCAGCGAGAGAGTAGCAATACTTTTGTCGGCGACCGGCGCACGGGTGAGTAACACGTATGCAACCTGCCCATAACAGGGGGATAATCGGAAGAAATTCCGTCTAATACCGCGTAACCCTGCATTATCTCATGATAACGCAGGTAAAGAAGCAATTCGGTTATGGATGGGCATGCGGAACATTAGGTAGTTGGTGAGGTAACGGCTCACCAAGCCGACGATGTATAGGGGTTCTGAGAGGAAGGTCCCCCACACTGGTACTGAGACACGGACCAGACTCCTACGGGAGGCAGCAGTGAGGAATATTGGTCAATGGGCGCGAGCCTGAACCAGCCAAGTCGCGTGAAGGATGAAGGTTCTATGGATTGTAAACTTCTTTTGTCAGGGGAACAAAGAGCTCACGAGTGAGCAGATGAGTGTACCTGAAGAAAAGCATCGGCTAACTCCGTGCCAGCAGCCGCGGTAA

+

:::::::::::::::::::::::::::::::::::::::::::::::::::::::::::::::::::::::::::::::::::::::::::::::::::::::::::::::::::::::::::::::::::::::::::::::::::::::::::::::::::::::::::::::::::::::::::::::::::::::::::::::::::::::::::::::::::::::::::::::::::::::::::::::::::::::::::::::::::::::::::::::::::::::::::::::::::::::::::::::::::::::::::::::::::::::::::::::::::::::::::::::::::::::::::::::::::::::::::::::::::::::::::::::::::::::::::::::::::::::::::::::::::::::::::::::::::::::::::::::::::::::::::::::::::::::::

@D0950.29_11104

GATGAACGCTAGCGACAGGCCTAACACATGCAAGTCGAGGGGTAGCACAAGGTAGTAATACTGAGGTGACGACCGGCGCACGGGTGAGTAACGCGTATGCAACCTACCTGTAAGAGTGGGATAGCCTCTCGAAAGAGAGATTAATACCGCATAATACCATTTCACTGCATGGTGAGATGGTTAAAGATTTATTGCTTACAGATGGGCATGCGTAACATTAGCTAGTTGGTGAGGTAACGGCTCACCAAGGCAACGATGTTTAGGGGTTCTGAGAGGAAGGTCCCCCGACACTGGTACTGAGACACGGACCAGACTCCTACGGGAGGCAGCAGTGAGGAATATTGGTCAATGGACGAGAGTCTGAACCAGCCAAGTCGCGTGAAGGATGAAGGTCTTATGGATTGTAAACTTCTTTTATACGGGAATAAAAATGCCACGTGTGGCATATTGCATGTACCGTATGAATAAGGATCGGCTAACTCCGTGCCAGCAGCCGCGGTAA

+

::::::::::::::::::::::::::::::::::::::::::::::::::::::::::::::::::::::::::::::::::::::::::::::::::::::::::::::::::::::::::::::::::::::::::::::::::::::::::::::::::::::::::::::::::::::::::::::::::::::::::::::::::::::::::::::::::::::::::::::::::::::::::::::::::::::::::::::::::::::::::::::::::::::::::::::::::::::::::::::::::::::::::::::::::::::::::::::::::::::::::::::::::::::::::::::::::::::::::::::::::::::::::::::::::::::::::::::::::::::::::::::::::::::::::::::::::::::::::::::::::::::::::::::::::::::

@D0950.29_111070

GATGAACGCTAGCTACAGGCTTAACACATGCAAGTCGAGGGGTAGCATGAAACTTAGCAATAAGTTTTGATGACGACCGGCGCACGGGTGAGTAACACGTATCCAACCTGCCTTTTACTCATGGATAGCCTTCTGAAAAGAAGATTAATACATGATGGTATTCAGAGTTTTCATGGACACTGAATTAAAGATTTTATCGGTAAGAGATGGGGATGCGTTCCATTAGATAGTAGGCGGGGTAACGGCCCACCTAGTCAACGATGGATAGGGGTTCTGAGAGGAAGGTCCCCCACATTGGAACTGAGACACGGTCCAAACTCCTACGGGAGGCAGCAGTGAGGAATATTGGTCAATGGACGTAAGTCTGAACCAGCCAAGTAGCGTGAAGGATGAAGGCTCTATGGGTCGTAAACTTCTTTTTATAAAA

+

:::::::::::::::::::::::::::::::::::::::::::::::::::::::::::::::::::::::::::::::::::::::::::::::::::::::::::::::::::::::::::::::::::::::::::::::::::::::::::::::::::::::::::::::::::::::::::::::::::::::::::::::::::::::::::::::::::::::::::::::::::::::::::::::::::::::::::::::::::::::::::::::::::::::::::::::::::::::::::::::::::::::::::::::::::::::::::::::::::::::::::::::::::::::::::::::::::::::::::::::::::::::::::::::::::::::::::

@D0950.29_111133

GATGAACGCTAGCGACAGGCCTAACACATGCAAGTCGAGGGGTAGCACAAGGTAGTAATACTGAGGTGACGACCGGCGCACGGGTGAGTAACGCGTATGCAACCTACCTGTAAGAGTGGGATAGCCTCTCGAAAGAGAGATTAATACCGCATAATACCATTTCACTGCATGGTGAGATGGTTAAAGATTTATTGCTTACAGATGGGCATGCGTAACATTAGCTAGTTGGTGAGGTAACGGCTCACCAAGGCAACGATGTTTAGGGGTTCTGAGAGGAAGGTCCCCCGACACTGGTACTGAGACACGGACCAGACTCCTACGGGAGGCAGCAGTGAGGAATATTGGTCAATGGACGAGAGTCTGAACCAGCCAAGTCGCGTGAAGGATGAAGGTCTTATGGATTGTAAACTTCTTTTATACGGGAATAAAAATGCCACGTGTGGCATATTGCATGTACCGTATGAATAAGGATCGGCTAACTCCGTGCCAGCAGCCGCGGTAA

+

::::::::::::::::::::::::::::::::::::::::::::::::::::::::::::::::::::::::::::::::::::::::::::::::::::::::::::::::::::::::::::::::::::::::::::::::::::::::::::::::::::::::::::::::::::::::::::::::::::::::::::::::::::::::::::::::::::::::::::::::::::::::::::::::::::::::::::::::::::::::::::::::::::::::::::::::::::::::::::::::::::::::::::::::::::::::::::::::::::::::::::::::::::::::::::::::::::::::::::::::::::::::::::::::::::::::::::::::::::::::::::::::::::::::::::::::::::::::::::::::::::::::::::::::::::::

@D0950.29_111153

GATGAACGCTGGCGGCGTGCTTAACACATGCAAGTCGAACGAACTGCGAGGAGCTTGCTCCTCAAAGTTAGTGGCGGACGGGTGAGTAACGCGTGGGTAACCTGCCCTATAGAGGGGGATAACGTTTGGAAACGAACGCTAATACCGCATAAACTATCGATGACTGCATGGTCATTATAGTAAAGATTTATCGCTATAGGATGGACCCGCGTTGGATTAGCTAGTTGGTGAGATAACAGCCCACCAAGGCGACGATCCATAGCCGGCCTGAGAGGGTGAACGGCCACATTGGGACTGAGACACGGCCCAAACTCCTACGGGAGGCAGCAGTGGGGAATATTGCACAATGGGCGAAAGCCTGATGCAGCGACGCCGCGTGAAGGAAGAAGGTCTTCGGATTGTAAACTTCTATCAGCAGGGAAGAATAAATGACGGTACCTGACTAAGAAGCTCCGGCTAACTACGTGCCAGCAGCCGCGGT

+

:::::::::::::::::::::::::::::::::::::::::::::::::::::::::::::::::::::::::::::::::::::::::::::::::::::::::::::::::::::::::::::::::::::::::::::::::::::::::::::::::::::::::::::::::::::::::::::::::::::::::::::::::::::::::::::::::::::::::::::::::::::::::::::::::::::::::::::::::::::::::::::::::::::::::::::::::::::::::::::::::::::::::::::::::::::::::::::::::::::::::::::::::::::::::::::::::::::::::::::::::::::::::::::::::::::::::::::::::::::::::::::::::::::::::::::::::::::::::::::::::

@D0950.29_111224

GATGAACGCTAGCTACAGGCTTAACACATGCAAGTCGAGGGTAGCATGAAACTTAGCAATAAGTTTTGATGACGACCGGCGCACGGGTGAGTAACACGTATCCAACCTGCCTTTTACTCATGGATAGCCTTCTGAAAAGAAGATTAATACATGATGGTATTCAGAGTTTTCATGGACACTGAATTAAAGATTTTATCGGTAAGAGATGGGGATGCGTTCCATTGATAGTAGGCGGGGTAACGGCCCACCTAGTCAACGATGGATAGGGGTTCTGAGAGGAAGGTCCCCACATTGGAACTGAGACACGGTCCAAACGTCCTACGGGAGGCAGCAGTGAGGAATATTGGTCAATGGACGTAAGTCTGAACCAGCCAAGTAGCGTGAAGGATGAAGGCTCTATGGGTCGTAAACTTCTTTTATAAAAGGAATAAAGTATGCCACGTGTGGTGTTTTTGGTATGTACTTTATGAATAAGGATCGGCTAACTCCGTGCCAGCAAGCCGC

+

::::::::::::::::::::::::::::::::::::::::::::::::::::::::::::::::::::::::::::::::::::::::::::::::::::::::::::::::::::::::::::::::::::::::::::::::::::::::::::::::::::::::::::::::::::::::::::::::::::::::::::::::::::::::::::::::::::::::::::::::::::::::::::::::::::::::::::::::::::::::::::::::::::::::::::::::::::::::::::::::::::::::::::::::::::::::::::::::::::::::::::::::::::::::::::::::::::::::::::::::::::::::::::::::::::::::::::::::::::::::::::::::::::::::::::::::::::::::::::::::::::::::::::::::::::::::

@D0950.29_111237

GATGAACGCTAGCGACAGGCCTAACACATGCAAGTCGAGGGGTAGCACAAGGTAGTAATACTGAGGTGACGACCGGCGCACGGGTGAGTAACGCGTATGCAACCTACCTGTAAGAGTGGGATAGCCTCTCGAAAGAGAGATTAATACCGCATAATACCATTTCACTGCATGGTGAGATGGTTAAAGATTTATTGCTTACAGATGGGCATGCGTAACATTAGCTAGTTGGTGAGGTAACGGCTCACCAAGGCAACGATGTTTAGGGGTTCTGAGAGGAAGGTCCCCCACACTGGTACTGAGACACGGACCAGACTCCTACGGGAGGCAGCAGTGAGGAAATATTGGTCAATGGACGAGAGTCTGAACCAGCCAAGTCGCGTGAAAGGATGAAAGGTCTTATGGATTGTAAACTTCTTTTATACGGGAATAAAAAATGCCACGTGTGGCATATTGCATGTACCGTATGAATAAAGGATCGGCTAAACTCCGTGCCAGCAGCCGCGGTAA

+

:::::::::::::::::::::::::::::::::::::::::::::::::::::::::::::::::::::::::::::::::::::::::::::::::::::::::::::::::::::::::::::::::::::::::::::::::::::::::::::::::::::::::::::::::::::::::::::::::::::::::::::::::::::::::::::::::::::::::::::::::::::::::::::::::::::::::::::::::::::::::::::::::::::::::::::::::::::::::::::::::::::::::::::::::::::::::::::::::::::::::::::::::::::::::::::::::::::::::::::::::::::::::::::::::::::::::::::::::::::::::::::::::::::::::::::::::::::::::::::::::::::::::::::::::::::::::::

@D0950.29_111247

ATTGAACGCTGGCGGCATGCCTTACACATGCAAGTCGAACGGTAACAGGTCTTCGGATGCTGACGAGTGGCGAACGGGTGAGTAATACATCGGAACGTGCCCGATCGTGGGGGATAACGAAGCGAAAGCTTTGCTAATACCGCATACGATCTACGGATGAAAGCAGGGGACCGCAAGGCCTTGCGCGAACGGAGCGGCCGATGGCAGATTAGGTAGTTGGTGGGATAAAAGCTTACCAAGCCGACGATCTGTAGCTGGTCTGAGAGGACGACCAGCCACACTGGGACTGAGACACGGCCCAGACTCCTACGGGAGGCAGCAGTGGGGAATTTTGGACAATGGGCGAAAGCCTGATCCAGCCATGCCGCGTGCAGGATGAAGGCCTCGGGTTGTAAACTGCTTTTGTACGGAACGAAAAAGACTCTGGTTAATACCTGGGGGTCCATG

+

:::::::::::::::::::::::::::::::::::::::::::::::::::::::::::::::::::::::::::::::::::::::::::::::::::::::::::::::::::::::::::::::::::::::::::::::::::::::::::::::::::::::::::::::::::::::::::::::::::::::::::::::::::::::::::::::::::::::::::::::::::::::::::::::::::::::::::::::::::::::::::::::::::::::::::::::::::::::::::::::::::::::::::::::::::::::::::::::::::::::::::::::::::::::::::::::::::::::::::::::::::::::::::::::::::::::::::::::::::::::::::::::

@D0950.29_111258

ATTGAACGCTGGCGGCAGGCCTAACACATGCAAGTCGAACGGTAGCACAGAGAGCTTGCTCTTGGGTGACGAGTGGCGGACGGGTGAGTAATGTCTGGGAAACTGCCCGATGGAGGGGGATAACTACTGGAAACGGTAGCTAATACCGCATAATGTCGCAAGACCAAAGAGGGGGACCTTCGGGCCTCTTGCCATCGGATGTGCCCAGATGGGATTAGCTAGTAGGTGGGGTAACGGCTCACCTAGGCGACGATCCCTAGCTGGTCTGAGAGGATGACCAGCCACACTGGAACTGAGACACGGTCCAGACTCCTACGGGAGGCAGCAGTGGGGAATATTGCACAATGGGCGCAAGCCTGATGCAGCCATGCCGCGTGTATGAAGAAGGCCTTCGGGTTGTAAAGTACTTTCAGCGAGGAGGAAGGCGTTGTGGTTAATAACCGCAGCGATTGACGTTCTCGCAGAAGAAGCACCGGCTAACTCCGTGCCAGCAGCCGCGGTAA

+

:::::::::::::::::::::::::::::::::::::::::::::::::::::::::::::::::::::::::::::::::::::::::::::::::::::::::::::::::::::::::::::::::::::::::::::::::::::::::::::::::::::::::::::::::::::::::::::::::::::::::::::::::::::::::::::::::::::::::::::::::::::::::::::::::::::::::::::::::::::::::::::::::::::::::::::::::::::::::::::::::::::::::::::::::::::::::::::::::::::::::::::::::::::::::::::::::::::::::::::::::::::::::::::::::::::::::::::::::::::::::::::::::::::::::::::::::::::::::::::::::::::::::::::::::::::::

@D0950.29_111289

GATGAACGCTAGCGACAGGCCTAACACATGCAAGTCGAGGGGTAGCACAAGGTAGCAATACTGAGGTGACGACCGGCGCACGGGTGAGTAACGCGTATGCAACCTACCTGTAAGAGTGGGATAGCCTCTCGAAAGAGAGATTAATACCGCATAATACCATTTTACTGCATGGTGAGATGGTTAAAGATTTGTTGCTTACAGATGGGCATGCGTAACATTAGCTAGTTGGTGAGGTAACGGCTCACCAAGGCAACGATGTTTAGGGGTTCTGAGAGGAAGGTCCCCCACATTGGAACTGAGACACGGTCCAAACTCCTACGGGAGGCAGCAGTGAGGAATATTGGTCAATGGACGTAAGTCTGAACCAGCCAAGTAGCGTGAAGGATGAAGGCTCTATGGGTCGTAAACTTCTTTTATAAAGGAATAAAGTATGCCACGTGTGGTGTTTTGTATGTACTTTATGAATAAGGATCGGCTAACTCCGTGCCAGCAGCCGCGGTAA

+

::::::::::::::::::::::::::::::::::::::::::::::::::::::::::::::::::::::::::::::::::::::::::::::::::::::::::::::::::::::::::::::::::::::::::::::::::::::::::::::::::::::::::::::::::::::::::::::::::::::::::::::::::::::::::::::::::::::::::::::::::::::::::::::::::::::::::::::::::::::::::::::::::::::::::::::::::::::::::::::::::::::::::::::::::::::::::::::::::::::::::::::::::::::::::::::::::::::::::::::::::::::::::::::::::::::::::::::::::::::::::::::::::::::::::::::::::::::::::::::::::::::::::::::::::::::

@D0950.29_111291

GATGAACGCTGGCGGCGTGCTTAACACATGCAAGTCGAACGAAGCACTTTGAAGAGCTTGCTCTTTAAAGTGACTGAGTGGCGGACGGGTGAGTAACGCGTGGGTAACCTGCCTCATACAGGGGGATAACAGTTAGAAATGACTGCTAACACCGCATAACCCGCTAGCATCGCATGATGCAGACGGAAAATATTTATAGGGTATGAGATCGGGCCCGCGTCTGATTAGCTAGTTGGTGGGGTAACGGCCTACCAAGGCAACGATCAGTAGCCGACTTGAGAGAGTGAACGGCCACATTGGGACTGAGACACGGCCCAAACTCCTACGGGAGGCAGCAGTGGGGAATATTGCACAATGGGGGAAACCCTGATGCAGCGACGCCGCGTGAAGGAAGAAGTATTTCGGTATGTAAACTTCTATCAGCAGGGAAGAAAATGACGGTACCTGACTAAGAAGCCCCGGCTAACTACGTGCCAGCAGCCG

+

:::::::::::::::::::::::::::::::::::::::::::::::::::::::::::::::::::::::::::::::::::::::::::::::::::::::::::::::::::::::::::::::::::::::::::::::::::::::::::::::::::::::::::::::::::::::::::::::::::::::::::::::::::::::::::::::::::::::::::::::::::::::::::::::::::::::::::::::::::::::::::::::::::::::::::::::::::::::::::::::::::::::::::::::::::::::::::::::::::::::::::::::::::::::::::::::::::::::::::::::::::::::::::::::::::::::::::::::::::::::::::::::::::::::::::::::::::::::::::::::::::

@D0950.29_111294

GATGAACGCTAGCGACAGGCCTAACACATGCAAGTCGAGGGGTAGCACAAGGTAGCAATACTGAGGTGACGACCGGCGCACGGGTGAGTAACGCGTATGCAACCTACCTGTAAGAGTGGGATAGCCTCTCGAAAGAGAGATTAATACCGCATAATACCATTTCACTGCATGGTGAGATGGTTAAAGATTTATTGCTTACAGATGGGCATGCGTAACATTAGCTAGTTGGTGAGGTAACGGCTCACCAAGGCAACGATGTTTAGGGGTTCTGAGAGGAAGGTCCCCCACACTGGTACTGAGACACGGACCAGACTCCTACGGGAGGCAGCAGTGAGGAATATTGGTCAATGGACGAGAGTCTGAACCAGCCAAGTCGCGTGAAGGATGAAGGTCTTATGGATTGTAAACTTCTTTTATACGGGAATAAAAAGAGCCACGTGTGGTTTATTGCATGTACCGTATGAATAAGGATCGGCTAACTCCGTGCCAGCAGCCGCGGTAA

+

::::::::::::::::::::::::::::::::::::::::::::::::::::::::::::::::::::::::::::::::::::::::::::::::::::::::::::::::::::::::::::::::::::::::::::::::::::::::::::::::::::::::::::::::::::::::::::::::::::::::::::::::::::::::::::::::::::::::::::::::::::::::::::::::::::::::::::::::::::::::::::::::::::::::::::::::::::::::::::::::::::::::::::::::::::::::::::::::::::::::::::::::::::::::::::::::::::::::::::::::::::::::::::::::::::::::::::::::::::::::::::::::::::::::::::::::::::::::::::::::::::::::::::::::::::::

@D0950.29_111295

GATGAACGCTGGCGGCGTGCTTAACACATGCAAGTCGAACGAAGCACTTTGAAGAGCTTGCTCTTTAAAGTGACTGAGTGGCGGACGGGTGAGTAACGCGTGGGTAACCTGCCTCATACAGGGGGATAACAGTTAGAAATGACTGCTAACACCGCATAACCCGCTAGTGTCGCATGACACAGACGGAAAATATTTATAGGTATGAGATGGGCCCGCGTCTGATTACGCTAGTTGGTGGGGTAACGGCCTACCAAGGCAACGATCAGTAGCCGACTTGAGAGAGTGATCGGCCACATTGGGACTGAGACACGGCCCAAACTCCTACGGGAGGCAGCAGTGGGGAATATTGGACAATGGGGGAAACCCCTGATCCAGCGACGCCGCGTGAGTGAAGAAGTATTTCGGTATGTAAAGCTCTATCAGCAGGGGAAGATAATGACAGTACCTGACTAAGAAGCCCCGGCTAACTACGTGCCAGCAGCCGCGGTAA

+

::::::::::::::::::::::::::::::::::::::::::::::::::::::::::::::::::::::::::::::::::::::::::::::::::::::::::::::::::::::::::::::::::::::::::::::::::::::::::::::::::::::::::::::::::::::::::::::::::::::::::::::::::::::::::::::::::::::::::::::::::::::::::::::::::::::::::::::::::::::::::::::::::::::::::::::::::::::::::::::::::::::::::::::::::::::::::::::::::::::::::::::::::::::::::::::::::::::::::::::::::::::::::::::::::::::::::::::::::::::::::::::::::::::::::::::::::::::::::::::::::::::::::

@D0950.29_111297

GACAGACGCTGGCGGCGTGCTTAACACATGCAAGTCGAGCGATGAAGCTTCTTCGGAAGTGGATTAGCGGCGGACGGGTGAGTAACACGTGGGTAACCTGCCTGTAAGAGTGGGATAGCCTCTCGAAAGAGAGATTAATACCGCATAATACCATTTCACTGCATGGTGAGATGGTTAAAGATTTATTGCTTATAGATGGGCATGCGTAACATTAGCTAGTTGGTGAGGTAACGGCTCACCAAGGCAACGATGTTTAGGGGTTCTGAGAGGAAGGTCCCCCACACTGGTACTGAGACACGGACCAGACTCCTACGGGAGGCAGCAGTGAGGAATATTGGTCAATGGACGAGAGTCTGAACCAGCCAAGTCGCGTGAAGGATGAAGGTCTTATGGATTGTAAACTTCTTTTATACGGGGAAATAAAAAGAGCCACGTGTGGCTTATTGCATGTACCGTATGAATAAGGATCGGCTAACTCCGTGCCAGCAGCCG

+

::::::::::::::::::::::::::::::::::::::::::::::::::::::::::::::::::::::::::::::::::::::::::::::::::::::::::::::::::::::::::::::::::::::::::::::::::::::::::::::::::::::::::::::::::::::::::::::::::::::::::::::::::::::::::::::::::::::::::::::::::::::::::::::::::::::::::::::::::::::::::::::::::::::::::::::::::::::::::::::::::::::::::::::::::::::::::::::::::::::::::::::::::::::::::::::::::::::::::::::::::::::::::::::::::::::::::::::::::::::::::::::::::::::::::::::::::::::::::::::::::::::::::::

@D0950.29_111307

GATGAACGCTAGCGACAGGCCTAACACATGCAAGTCGAGGGGTAGCACAAGGAAGCTTGCTTCTGAGGTGACGACCGGCGCACGGGTGAGTAACGCGTATGCAACCTACCTGTAAGAGTGGGATAGCCTCTCGAAAGAGAGATTAATACCGCATAATACCATTTCACTGCATGGTGAGATGGTTAAAGATTTATCGCTGAAAGATGAGCTCGCGTCTGATTAGCTAGTTGGTAAGGTAATGGCTTACCAAGGCAACGATCAGTAGCCGGACTGAGAGGTTGAACGGCCACATTGGGACTGAGACACGGCCCAGACTCCTACGGGAGGCAGCAGTGGGGAATATTGCACAATGGAGGAAACTCTGATGCAGCGATGCCGCGTGAGGGAAGAAGGTTTTAGGATTGTAAACCTCTGTCTTCAGGGACGATAATGACGGTACCTGAGGAGGAAGCTCCGGCTAACTACGTGCCAGCAGCCGCGGTAA

+

::::::::::::::::::::::::::::::::::::::::::::::::::::::::::::::::::::::::::::::::::::::::::::::::::::::::::::::::::::::::::::::::::::::::::::::::::::::::::::::::::::::::::::::::::::::::::::::::::::::::::::::::::::::::::::::::::::::::::::::::::::::::::::::::::::::::::::::::::::::::::::::::::::::::::::::::::::::::::::::::::::::::::::::::::::::::::::::::::::::::::::::::::::::::::::::::::::::::::::::::::::::::::::::::::::::::::::::::::::::::::::::::::::::::::::::::::::::::::::::::::::

@D0950.29_111318

GATGAACGCTAGCTACAGGCTTAACACATGCAAGTCGAGGGGTAGCATGAAACTTAGCAATAAGTTTTGATGACGACCGGCGCACGGGTGAGTAACACGTATCCAACCTGCCTTTTACTCATGGATAGCCTTCTGAAAAGAAGATTAATACATGATGGTATTCAGAGTTTTCATGGACACTGAATTAAAGATTTTATCGGTAAGAGATGGGGATGCGTTCCATTAGATAGTAGGCGGGGTAACGGCCCACCTAGTCAACGATGGATAGGGGTTCTGAGAGGAAGGTCCCCCACATTGGAACTGAGACACGGTCCAAACGTCCTACGGGAGGCAGCAGTGAGGAATATTGGTCAATGGACGTAAGTCTGAACCAGCCAAGTAGCGTGAAGGATGAAGGCTCTATGGGTCGTAAACTTCTTTTTATAAAAGGAATAAAGTATGCCACGTGTGGTGTTTTTGTATGTACTTTATGAATAAGGATCGGCTAACTCCGTGCCAGCAGCC

+

::::::::::::::::::::::::::::::::::::::::::::::::::::::::::::::::::::::::::::::::::::::::::::::::::::::::::::::::::::::::::::::::::::::::::::::::::::::::::::::::::::::::::::::::::::::::::::::::::::::::::::::::::::::::::::::::::::::::::::::::::::::::::::::::::::::::::::::::::::::::::::::::::::::::::::::::::::::::::::::::::::::::::::::::::::::::::::::::::::::::::::::::::::::::::::::::::::::::::::::::::::::::::::::::::::::::::::::::::::::::::::::::::::::::::::::::::::::::::::::::::::::::::::::::::::::::

@D0950.29_111446

GATGAACGCTAGCGACAGGCCTAACACATGCAAGTCGAGGGGTAGCACAAGGAAGCTTGCTTCTGAGGTGACGACCGGCGCACGGGTGAGTAACGCGTATGCAACCTACCTGTAAGAGTGGGATAGCCTCTCGAAAGAGAGATTAATACCGCATAATACCATTTCACTGCATGGTGAGATGGTTAAAGATTTATTGCTTACAGATGGGCATGCGTAACATTAGCTAGTTGGTGAGGTAACGGCTCACCAAGGCAACGATGTTTAGGGGTTCTGAGAGGAAGGTCCCCCACACTGGTACTGAGACACGGACCAGACTCCTACGGGAGGCAGCAGTGAGGAATATTGGTCAATGGACGTAAGTCTGAACCAGCCAAGTAGCGTGAAGGATGAAGGCTCTATGGGTCGTAAACTTCTTTTATAAAGGAATAAAGTATGCCACGTGTGGTG

+

:::::::::::::::::::::::::::::::::::::::::::::::::::::::::::::::::::::::::::::::::::::::::::::::::::::::::::::::::::::::::::::::::::::::::::::::::::::::::::::::::::::::::::::::::::::::::::::::::::::::::::::::::::::::::::::::::::::::::::::::::::::::::::::::::::::::::::::::::::::::::::::::::::::::::::::::::::::::::::::::::::::::::::::::::::::::::::::::::::::::::::::::::::::::::::::::::::::::::::::::::::::::::::::::::::::::::::::::::::::::::::::::

@D0950.29_111455

GATGAACGCTGGCGGCGTGCTTAACACATGCAAGTCGAGCGAGGAATCACCTTCGGGTGTGAACTAGCGGCGGACGGGTGAGTAACACGTGGGCAACCTGCCTTACAGAGGGGGATAGCCTTCCGAAAGGAAGATTAATACCGCATATTATGAGTTTTCTGCATGGGGAATTCATGAAAGGAGCAATCCGCTGTAAGATGGGCCCGCGGCGCATTAGCTAGTTGGTGAGGTAACGGCTCACCAAGGCGACGATGCGTAGCCGACCTGAGAGGGTGATCGGCCACATTGGGACTGAGACACGGCCCAGACTCCTACGGGAGGCAGCAGTGGGGAATATTGCACAATGGGGGAAACCCTGATGCAGCAACGCCGCGTGAGTGATGAAGGCCTTCGGGTTGTAAAGCTCTGTCTTCAGGGACGATAATGACGGTACCTGAGGAGGAAGCCACGGCTAACTACGTGCCAGCAGCCGCGGTAA

+

::::::::::::::::::::::::::::::::::::::::::::::::::::::::::::::::::::::::::::::::::::::::::::::::::::::::::::::::::::::::::::::::::::::::::::::::::::::::::::::::::::::::::::::::::::::::::::::::::::::::::::::::::::::::::::::::::::::::::::::::::::::::::::::::::::::::::::::::::::::::::::::::::::::::::::::::::::::::::::::::::::::::::::::::::::::::::::::::::::::::::::::::::::::::::::::::::::::::::::::::::::::::::::::::::::::::::::::::::::::::::::::::::::::::::::::::::::::::::::::

@D0950.29_111465

GATGAACGCTAGCTACAGGCTTAACACATGCAAGTCGAGGGGTAGCATGAAACTTAGCAATAAGTTTTGATGACGACCGGCGCACGGGTGAGTAACACGTATCCAACCTGCCTTTTACTCATGGATAGCCTTCTGAAAAGAAGATTAATACATGATGGTATTCAGAGTTTTCATGGACACTGAATTAAAGATTTTATCGGTAAGAGATGGGGATGCGTTCCATTAGATAGTAGGCGGGGTAACGGCCCACCTAGTCAACGATGGATAGGGGTTCTGAGAGGAAGGTCCCCCACATTGGAACTGAGACACGGTCCAAACGTCCGTACGGGAGGCAGCAGTGAGGAATATTGTCAATGGACGTAAGTCTGAACCAGCCAAGTAGCGTGAAGGATGAAGGCTCTATGGGTCGTAAACTTCTTTTTATAAAAGGAATAAAGTATGCCACGTGTGGTGTTTTTGTATGTACTTTATGAATAAGGATCGGCT

+

::::::::::::::::::::::::::::::::::::::::::::::::::::::::::::::::::::::::::::::::::::::::::::::::::::::::::::::::::::::::::::::::::::::::::::::::::::::::::::::::::::::::::::::::::::::::::::::::::::::::::::::::::::::::::::::::::::::::::::::::::::::::::::::::::::::::::::::::::::::::::::::::::::::::::::::::::::::::::::::::::::::::::::::::::::::::::::::::::::::::::::::::::::::::::::::::::::::::::::::::::::::::::::::::::::::::::::::::::::::::::::::::::::::::::::::::::::::::::::::::::::::

@D0950.29_111477

GATGAACGCTGGCGGCGTGCTTAACACATGCAAGTCGAACGAAGCGCTGGAGGAGCTTGCTCCAAAGGTGACTGAGTGGCGGACGGGTGAGTAACGCGTGGGTAACCTGCCTTACACTGGGGGATAACAGTTGGAAACGACTGCTAATACCGCATAAGCGCACAGTATTGCATGATACAGTGTGAAAAACTCCGGTGGTGTGAAGATGGCACCCCGCGTCTGATTAGGCTAGTTGGTGAGGTAATCGGCTACCAAGGCAACGATCAGTAGCCGGCTTGAGGAGAGTGAACGGCCACATTGGGACTGAGACACGGCCCAAACTCCTACGGGAGGCAGCAGTGGGGGAATATTTGCAGCAATGGGGGAAACCCTTGATGCAGCAACGCCGCGTGAGTGAAGAAGTATTTCGGTATGTAAAGCTCT

+

:::::::::::::::::::::::::::::::::::::::::::::::::::::::::::::::::::::::::::::::::::::::::::::::::::::::::::::::::::::::::::::::::::::::::::::::::::::::::::::::::::::::::::::::::::::::::::::::::::::::::::::::::::::::::::::::::::::::::::::::::::::::::::::::::::::::::::::::::::::::::::::::::::::::::::::::::::::::::::::::::::::::::::::::::::::::::::::::::::::::::::::::::::::::::::::::::::::::::::::::::::::::::::::::::::::::

@D0950.29_111514

GATGAACGCTGGCGGCGTGCTTAACACATGCAAGTCGAACGAAGCACTTTGAAGAGCTTGCTCTTTAAAGTGACTGAGTGGCGGACGGGTGAGTAACGCGTGGGTAACCTGCCTCATACAGGGGGATAACAGTTAGAAATGGACCTGCTAACACCGCATAACCCGCTAGTGTCGCATGACACAGACGGAAAATATTTATAGGTATGAGATGGGCCCGCGTCTGATTAGCTAGTTGGTGGGGTAACGGCCTACCAAGGCAACGATCAGTAGCCGACTTGAGAGAGTGATCGGCCACATTGGGACTGAGACACGGCCCAAACTCCTACGGGAGGCAGCAGTGGGAATATTGGACAATGGGGGAAACCCTGATCCAGCGACGCCGCGTGAGTGAAGAAGTATTTCGGTATGTAAAGCTCTATCAGCAGGGGAAGATAATGACAGTACCTGACTAAGAAGCCCCGGCTAACTACGTGCCAGCAGCCGCGGTAA

+

:::::::::::::::::::::::::::::::::::::::::::::::::::::::::::::::::::::::::::::::::::::::::::::::::::::::::::::::::::::::::::::::::::::::::::::::::::::::::::::::::::::::::::::::::::::::::::::::::::::::::::::::::::::::::::::::::::::::::::::::::::::::::::::::::::::::::::::::::::::::::::::::::::::::::::::::::::::::::::::::::::::::::::::::::::::::::::::::::::::::::::::::::::::::::::::::::::::::::::::::::::::::::::::::::::::::::::::::::::::::::::::::::::::::::::::::::::::::::::::::::::::::::

@D0950.29_111524

GATGAACGCTAGCTACAGGCTTAACACATGCAAGTCGAGGGGTAGCATGAAACTTAGCAATAAGTTTTGATGACGACCGGCGCACGGGTGAGTAACACGTATCCAACCTGCCTTTTACTCATGGATAGCCTTCTGAAAAGAAGATTAATACATGATGGTATTCAGAGTTTTCATGGACACTGAATTAAAGATTTTATCGGTAAGAGATGGGGATGCGTTCCATTAGATAGTAGGCGGGGTAACGGCCCACCTAGTCAACGATGGATAGGGGTTCTGAGAGGAAGGTCCCCCACATTGGAACTGAGACACGGTCCAAACGTCCTACGGGAGGCAGCAGTGAGGAATATTGGTCAATGGACGTAAGTCTGAACCAGCCAAGTAGCGTGAAGGATGAAGGCTCTATGGGTCGTAAACTTCTTTTTATAAAAGGAATAAAGTATGCCACGTGTGGTGTTTTTGTATGTAACTTTATGAATAAGGATCGGCTAACTCCGTGCCAGCAGCCGC

+

:::::::::::::::::::::::::::::::::::::::::::::::::::::::::::::::::::::::::::::::::::::::::::::::::::::::::::::::::::::::::::::::::::::::::::::::::::::::::::::::::::::::::::::::::::::::::::::::::::::::::::::::::::::::::::::::::::::::::::::::::::::::::::::::::::::::::::::::::::::::::::::::::::::::::::::::::::::::::::::::::::::::::::::::::::::::::::::::::::::::::::::::::::::::::::::::::::::::::::::::::::::::::::::::::::::::::::::::::::::::::::::::::::::::::::::::::::::::::::::::::::::::::::::::::::::::::::

@D0950.29_111539

ATTGAACGCTGGCGGCATGCCTTACACATGCAAGTCGAACGGTAACAGGTCTTCGGATGCTGACGAGTGGCGAACGGGTGAGTAATACATCGGAACGTGCCCGATCGTGGGGATAACGAAGCGAAAGCTTTGCTAATACCGCATACGATCTACGGATGAAAGCAGGGGACCGCAAGGCCTTGCGCGAACGGAGCGGCCGATGGCAGATTAGGTAGTTGGTGGGATAAAAGCTTACCAAGCCGACGATCTGTAGCTGGTCTGAGAGGACGACCAGCCACACTGGGACTGAGACACGGCCCAGACTCCTACGGGAGGCAGCAGTGGGGAATTTTGGACAATGGGCGAAAGCCTGATCCAGCCATGCCGCGTGCAGGATGAAGGCCTTCGGGTTGTAAACTGCTTTTGTACGGAACGAAAGACTCTGGTTAATACCTGGGGTCCATGACGGTACCGTAAGAATAAGCACCGGCTAACTACGTGCCAGCAGCCGCGGTAA

+

::::::::::::::::::::::::::::::::::::::::::::::::::::::::::::::::::::::::::::::::::::::::::::::::::::::::::::::::::::::::::::::::::::::::::::::::::::::::::::::::::::::::::::::::::::::::::::::::::::::::::::::::::::::::::::::::::::::::::::::::::::::::::::::::::::::::::::::::::::::::::::::::::::::::::::::::::::::::::::::::::::::::::::::::::::::::::::::::::::::::::::::::::::::::::::::::::::::::::::::::::::::::::::::::::::::::::::::::::::::::::::::::::::::::::::::::::::::::::::::::::::::::::::::::

@D0950.29_111586

GATGAACGCTAGCTACAGGCTTAACACATGCAAGTCGAGGGGTAGCATGAAACTTAGCAATAAGTTTTGATGACGACCGGCGCACGGGTGAGTAACACGTATCCAACCTGCCTTTTACTCATGGATAGCCTTCTGAAAAGAAGATTAATACATGATGGTATTCAGAGTTTTCATGGACACTGAATTAAAGATTTTATCGGTAAGAGATGGGGATGCGTTCCATTAGATAGTAGGCGGGGTAACGGCCCACCTAGTCAACGATGGATAGGGGTTCTGAGAGGAAGGTCCCCCACATTGGAACTGAGACACGGTCCAAACGTCCTACGGGAGGCAGCAGTGAGGAATATTGGTCAATGGACGTAAGTCTGAACCAGCCAAGTAGCGTGAAGGATGAAGGCTCTATGGGTCGTAAACTTCTTTTTATAAAAGGAATAAAGTATGCCACGTGTGGTGTTTTTGTATGTACTTTATGAATAAGGATCGGCTAACTCCGTGCCAGCAGCCGC

+

::::::::::::::::::::::::::::::::::::::::::::::::::::::::::::::::::::::::::::::::::::::::::::::::::::::::::::::::::::::::::::::::::::::::::::::::::::::::::::::::::::::::::::::::::::::::::::::::::::::::::::::::::::::::::::::::::::::::::::::::::::::::::::::::::::::::::::::::::::::::::::::::::::::::::::::::::::::::::::::::::::::::::::::::::::::::::::::::::::::::::::::::::::::::::::::::::::::::::::::::::::::::::::::::::::::::::::::::::::::::::::::::::::::::::::::::::::::::::::::::::::::::::::::::::::::::::

@D0950.29_111668

GATGAACGCTGGCGGCGTGCTTAACACATGCAAGTCGAACGAAGCAGCTTTCTTGCTTGCAAGAAAGCTGACTTAGTGGCGGACGGGTGAGTAACGCGTGGGTAACCTGCCTCATACAGGGGGATAACAGTTGGAAACGACTGCTAAGACCGCATAACCCGCTAGTGTCGCATGACACGGACGGAAAATATTTTATAGGTATGAGATGGGCCCGCGTCTGATTAGCTAGTTGGTAAGGTAACGCTTACCAAGGCGACGATCAGTAGCCGACTTGAGAGAGTGATCGGCCACATTGGGACTGAGACACGGCCCAAACTCCTACGGGAGGCAGCAGTGGGGAATATTGGACAATGGGGGAAACCCTGATCCAGCGACGCCGCGTGAGTGAAGAAGTATTTCGGTATGTAAAGCTCTATCAGCAGGGAAGATAATGACAGTACCTGACTAAGAAGCCCCCGGCTAACTACGTGCCAGCAGCCGC

+

:::::::::::::::::::::::::::::::::::::::::::::::::::::::::::::::::::::::::::::::::::::::::::::::::::::::::::::::::::::::::::::::::::::::::::::::::::::::::::::::::::::::::::::::::::::::::::::::::::::::::::::::::::::::::::::::::::::::::::::::::::::::::::::::::::::::::::::::::::::::::::::::::::::::::::::::::::::::::::::::::::::::::::::::::::::::::::::::::::::::::::::::::::::::::::::::::::::::::::::::::::::::::::::::::::::::::::::::::::::::::::::::::::::::::::::::::::::::::::::::::

@D0950.29_111697

GACGAACGCTGGCGGCGCGCCTAACACATGCAAGTCGAACGGAGTTATTTTGACAGATTCTTTCGGGATGAAGATAAATTAACTTAGTGGCGGACGGGTGAGTAACACGTGAGCAACCTGCCTTACAGAGGGGGATAACGTTTGGAAACGAACGCTAATACCGCATAACATTATTAAATCGCATGATTTGATAATCAAAGGAGAATCCGCTGAAAGATGGGCTCGCGTCTGATTAGATAGTTGGTGAGGTAATGGCTCACCAAGTCGACGATCAGTAGCCGGACTGAGAGGTTGAACGGCCACATTGGGACTGAGACACGGCCCAGACTCCTACGGGAGGCAGCAGTGGGGAATATTGCACAATGGGGGAAACCCTGATGCAGCGACGCCGCGTGAGGGAAGAAGGTTTTCGGATTGTAAACCTCTGTCTTCAGGGACGATAATGACGGTACCTGAGGAGGAAGCCACGGCTAACTACGTGCCAGCAGCCGCGGTAA

+

:::::::::::::::::::::::::::::::::::::::::::::::::::::::::::::::::::::::::::::::::::::::::::::::::::::::::::::::::::::::::::::::::::::::::::::::::::::::::::::::::::::::::::::::::::::::::::::::::::::::::::::::::::::::::::::::::::::::::::::::::::::::::::::::::::::::::::::::::::::::::::::::::::::::::::::::::::::::::::::::::::::::::::::::::::::::::::::::::::::::::::::::::::::::::::::::::::::::::::::::::::::::::::::::::::::::::::::::::::::::::::::::::::::::::::::::::::::::::::::::::::::::::::::::::

@D0950.29_111706

ATTGAACGCTGGCGGCAGGCCTAACACATGCAAGTCGAACGGTAGCACAGAGGAGCTTGCTCCTTGGGTGACGAGTGGCGGACGGGTGAGTAATGTCTGGGAAACTGCCCGATGGAGGGGGATAACTACTGGAAACGGTAGCTAATACCGCATAACGTCGCAAGACCAAAGAGGGGGACCTTCGGGCCTCTTGCCATCGGATGTGCCCAGATGGGATTAGCTAGTAGGTGGGGTAACGGCTCACCTAGGCGACGATCCCTAGCTGGTCTGAGAGGATGACCAGCCACACTGGAACTGAGACACGGTCCAGACTCCTACGGGAGGCAGCAGTGGGGAATATTGCACAATGGGCGCAAGCCTGATGCAGCCATGCCGCGTGTATGAAGAAGGCCTTCGGGTTGTAAAGTACTTTCAGCGAGGAGGAAGGTGCTGTGGTTAATAACCGCAGTAATTGACGTTACTCGCAGAAGAAGCACCGGCTAACTCCGTGCCAGCAGCCGCGGTAA

+

::::::::::::::::::::::::::::::::::::::::::::::::::::::::::::::::::::::::::::::::::::::::::::::::::::::::::::::::::::::::::::::::::::::::::::::::::::::::::::::::::::::::::::::::::::::::::::::::::::::::::::::::::::::::::::::::::::::::::::::::::::::::::::::::::::::::::::::::::::::::::::::::::::::::::::::::::::::::::::::::::::::::::::::::::::::::::::::::::::::::::::::::::::::::::::::::::::::::::::::::::::::::::::::::::::::::::::::::::::::::::::::::::::::::::::::::::::::::::::::::::::::::::::::::::::::::::

@D0950.29_111837

GATGAACGCTGGCGGCGTGCTTAACACATGCAAGTCGAACGAAGCACTTAACTTAGAATCTTCGGAGGAAGAGTTTTGTGACTTAGTGGCGGACGGGTGAGTAACGCGTGGGTAACCTGCCTTATACTGGGGGATAACAGTTAGAAATGACTGCTAATACCGCATAAGCGCACAGTATCACATGATACAGTGCGAAAAACTCCGGTGGTATAAGATGGACCCGCGTCTGATTAGCTAGTTGGTAAGGTAACGGCTTACCAAGGCGACGATCAGTAGCCGACTTGAGAGAGTGATCGGCCACATTGGGACTGAGACACGGCCCAAACTCCTACGGGAGGCAGCAGTGGGGAATATTGCACAATGGGCGAAAGCCTGATGCAGCGACGCCGCGTGAAGGATGAAGTATTTCGGTACGTAAACTTCTATCAGCAAGGAAGATAATGACGGTACTTGACTAAGAAGCCCCCGGCTAACTACGTGCCAGCAGCCGC

+

:::::::::::::::::::::::::::::::::::::::::::::::::::::::::::::::::::::::::::::::::::::::::::::::::::::::::::::::::::::::::::::::::::::::::::::::::::::::::::::::::::::::::::::::::::::::::::::::::::::::::::::::::::::::::::::::::::::::::::::::::::::::::::::::::::::::::::::::::::::::::::::::::::::::::::::::::::::::::::::::::::::::::::::::::::::::::::::::::::::::::::::::::::::::::::::::::::::::::::::::::::::::::::::::::::::::::::::::::::::::::::::::::::::::::::::::::::::::::::::::::::::::::::

@D0950.29_111879

GATGAACGCTGGCGGCATGCCTTACACATGCAAGTCGAACGGTAACAGGTCTTCGGATGCTGACGAGTGGCGAACGGGTGAGTAATACATCGGAACGTGCCCGATCGTGGGGGATAACGAAGCGAAAGCTTTGCTAATACCGCATACGATCTACGGATGAAAGCAGGGGACCGCAAGGCCTTGCGCGAACGGAGCGGCCGATGGCAGATTAGGTAGTTGGTGGGATAAAAGCTTACCAAGCCGACGATCTGTAGCTGGTCTGAGAGGACGACCAGCCACACTGGGACTGAGACACGGCCCAGACTCCTACGGGAGGCAGCAGTGGGGAATTTTGGACAATGGGCGAAAGCCTGATCCAGCCATGCCGCGTGCAGGATGAAGGCCTTCGGGTTGTAAACTGCTTTTGTACGGAACGAAAAGACTCTGGTTAATACCTGGGGTCCATGACGGTACCGTAAGAATAAGCACCGGCTAACTACGTGCCAGCAGCCGCGGTAA

+

::::::::::::::::::::::::::::::::::::::::::::::::::::::::::::::::::::::::::::::::::::::::::::::::::::::::::::::::::::::::::::::::::::::::::::::::::::::::::::::::::::::::::::::::::::::::::::::::::::::::::::::::::::::::::::::::::::::::::::::::::::::::::::::::::::::::::::::::::::::::::::::::::::::::::::::::::::::::::::::::::::::::::::::::::::::::::::::::::::::::::::::::::::::::::::::::::::::::::::::::::::::::::::::::::::::::::::::::::::::::::::::::::::::::::::::::::::::::::::::::::::::::::::::::::

@D0950.29_111882

GATGAACGCTGGCGGCGTGCTTAACACATGCAAGTCGAGCGAGGAATCACCTTCGGGTGTGAACTAGCGGCGGACGGGTGAGTAACACGTGGGCAACCTGCCTTACAGAGGGGGATAGCCTTCCGAAAGGAAGATTAATACCGCATATTATGAGTTTTCTGCATGGGGAATTCATGAAAGGAGCAATCCGCTGTAAGATGGGCCCGCGGCGCATTAGCTAGTTGGTGAGGTAACGGCTCACCAAGGCGACGATGCGTAGCCGACCTGAGAGGGTGATCGGCCACATTGGGACTGAGACACGGCCCAGACTCCTACGGGAGGCAGCAGTGGGGAATATTGCACAATGGGGGAAACCCTGATGCAGCAACGCCGCGTGAGTGATGAAGGCCTTCGGGTTGTAAAGCTCTGTC

+

::::::::::::::::::::::::::::::::::::::::::::::::::::::::::::::::::::::::::::::::::::::::::::::::::::::::::::::::::::::::::::::::::::::::::::::::::::::::::::::::::::::::::::::::::::::::::::::::::::::::::::::::::::::::::::::::::::::::::::::::::::::::::::::::::::::::::::::::::::::::::::::::::::::::::::::::::::::::::::::::::::::::::::::::::::::::::::::::::::::::::::::::::::::::::::::::::::::::::::::::::::::::::

@D0950.29_111901

GATGAACGCTAGCTACAGGCTTAACACATGCAAGTCGAGGGGTAGCATGAAACTTAGCAATAAGTTTTGATGACGACCGGCGCACGGGTGAGTAACACGTATCCAACCTGCCTTTTACTCATGGATAGCCTTCTGAAAAGAAGATTAATACATGATGGTATTCAGAGTTTTCATGGACACTGAATTAAAGATTTTATCGGTAAGAGATGGGGATGCGTTCCATTAGATAGTAGGCGGGGTAACGGCCCACCTAGTCAACGATGGATAGGGGTTCTGAGAGGAAGGTCCCCACATTGGAACTGAGACACGGTCCAAACGTCCTACGGGAGGCAGCAGTGAGGAATATTGGTCAATGGACGTAAGTCTGAACCAGCCAAGTAGCGTGAAGGATGAAGGCTCTATGGGTCGTAAACTTCTTTTATAAAAGGAATAAAGTATGCCACGTGTGGTGTTTTGTATGTACTTTATGAATAAGGATCGGCTAACTCCGTGCCAGCAGCCGC

+

:::::::::::::::::::::::::::::::::::::::::::::::::::::::::::::::::::::::::::::::::::::::::::::::::::::::::::::::::::::::::::::::::::::::::::::::::::::::::::::::::::::::::::::::::::::::::::::::::::::::::::::::::::::::::::::::::::::::::::::::::::::::::::::::::::::::::::::::::::::::::::::::::::::::::::::::::::::::::::::::::::::::::::::::::::::::::::::::::::::::::::::::::::::::::::::::::::::::::::::::::::::::::::::::::::::::::::::::::::::::::::::::::::::::::::::::::::::::::::::::::::::::::::::::::::::::

@D0950.29_112001

GATGAACGCTGGCGGCGTGCTTAACACATGCAAGTCGAACGAAGCGCTGGAGGAGCTTGCTCCAAAGGTGACTGAGTGGCGGACGGGTGAGTAACGCGTGGGTAACCTGCCTTACACTGGGGGATAACAGTTGGAAACGACTGCTAATACCGCATAAGCGCACAGTATTGCATGATACAGTGTGAAAAACTCCGGTGGTGTAAGATGGACCCGCGTCTGATTAGCTAGTTGGTGAGGTAATGGCTCACCAAGGCAACGATCAGTAGCCGGCTTGAGAGAGTGAACGGCCACATTGGGACTGAGACACGGCCCAAACTCCTACGGGAGGCAGCAGTGGGGAATATTGCACAATGGGGGAAACCCTGATGCAGCAACGCCGCGTGAGTGAAGAAGTATTTCGGTATGTAAAGCTCTATCAGCAGGGAAGATAATGACGGTACCTGACTAAGAAGCCCCGGCTAACTACGTGCCAGCAGCCGCGGTAA

+

:::::::::::::::::::::::::::::::::::::::::::::::::::::::::::::::::::::::::::::::::::::::::::::::::::::::::::::::::::::::::::::::::::::::::::::::::::::::::::::::::::::::::::::::::::::::::::::::::::::::::::::::::::::::::::::::::::::::::::::::::::::::::::::::::::::::::::::::::::::::::::::::::::::::::::::::::::::::::::::::::::::::::::::::::::::::::::::::::::::::::::::::::::::::::::::::::::::::::::::::::::::::::::::::::::::::::::::::::::::::::::::::::::::::::::::::::::::::::::::::::::::

@D0950.29_112049

GATGAACGCTAGCTACAGGCTTAACACATGCAAGTCGAGGGGTAGCATGAAACTTAGCAATAAGTTTTGATGACGACCGGCGCACGGGTGAGTAACACGTATCCAACCTGCCTTTTACTCATGGATAGCCTTCTGAAAAGAAGATTAATACATGATGGTATTCAGAGTTTTCATGGACACTGAATTAAAGATTTTATCGGTAAGAGATGGGGGATGCGTTCCATTAGATAGTAGGCGGGGTAACGGCCCACCTAGTCAACGATGGATAGGGGTTCTGAGAGGAAGGTCCCCCACACTGGTACTGAGACACGGACCCAGGACGTCCTACGGGAGGCAGCAGTGAGGAATATTGGTCAATGGACGTAAGTCTGAACCAGCCAAGTAGCGTGAAGGATGAAGGCTCTATGGGTCGTAAAC

+

:::::::::::::::::::::::::::::::::::::::::::::::::::::::::::::::::::::::::::::::::::::::::::::::::::::::::::::::::::::::::::::::::::::::::::::::::::::::::::::::::::::::::::::::::::::::::::::::::::::::::::::::::::::::::::::::::::::::::::::::::::::::::::::::::::::::::::::::::::::::::::::::::::::::::::::::::::::::::::::::::::::::::::::::::::::::::::::::::::::::::::::::::::::::::::::::::::::::::::::::::::::::::::::::::

@D0950.29_112051

GATGAACGCTAGCGACAGGCCTAACACATGCAAGTCGAGGGGTAGCACAAGGAAGCTTGCTTCTGAGGTGACGACCGGCGCACGGGTGAGTAACGCGTATGCAACCTGCCTATAAGAAGGGGATAGCCTCTCGAAAGAGAGATTAATACCGTATAACACTATGAAATCGCATGGTTTTATAGTTAAAGATTTATTGCTTATAGATGGGCATGCGTAACATTAGCTAGTTGGTGAGGTAACGGCTCACCAAGGCAACGATGTTTAGGGGTTCTGAGAGGAAGGTCCCCCACACTGGTACTGAGACACGGACCAGACTCCTACGGGAGGCAGCAGTGAGGAATATTGGTCAATGGACGAGAGTCTGAACCAGCCAAGTCGCGTGAAGGATGAAGGTCTTATGGATTGTAAACTTCTTTTATACGGGGAAATAAAAAGAGCCACGTGTGGCTTATTGCATGTACCGTATGAATAAGGATCGGCTAACTCCGTGCCAGCAGCCG

+

::::::::::::::::::::::::::::::::::::::::::::::::::::::::::::::::::::::::::::::::::::::::::::::::::::::::::::::::::::::::::::::::::::::::::::::::::::::::::::::::::::::::::::::::::::::::::::::::::::::::::::::::::::::::::::::::::::::::::::::::::::::::::::::::::::::::::::::::::::::::::::::::::::::::::::::::::::::::::::::::::::::::::::::::::::::::::::::::::::::::::::::::::::::::::::::::::::::::::::::::::::::::::::::::::::::::::::::::::::::::::::::::::::::::::::::::::::::::::::::::::::::::::::::::::::

@D0950.29_112071

GATGAACGCTGGCGGCGTGCCTAACACATGCAAGTTGAGCGATTTACTTCGGTAAAGAGCGGCGGACGGGTGAGTAACGCGTGGGTAACCTACCCTGTACACACGGATAACATACCGAAAGGTATGCTAATACGGGATAACATATTTGAGAGGCATCTCTTAAATATCAAAGGTGAGCCAGTACAGGATGGACCCGCGTCTGATTAGCTAGTTGGTAAGGTAACGGCTTACCAAGGCGACGATCAGTAGCCGACCTGAGAGGGTGATCGGCCACATTGGAACTGAGACACGGTCCAAACTCCTACGGGAGGCAGCAGTGGGGAATATTGCACAATGGGCGAAAGCCTGATGCAGCAACGCCGCGTGAGTGATGAAGGCCTTCGGGTCGTAAAACTCTGTCCTCAAGGAAGATAATGACGGTACTTGAGGAGGAAGCCCCGGCTAA

+

:::::::::::::::::::::::::::::::::::::::::::::::::::::::::::::::::::::::::::::::::::::::::::::::::::::::::::::::::::::::::::::::::::::::::::::::::::::::::::::::::::::::::::::::::::::::::::::::::::::::::::::::::::::::::::::::::::::::::::::::::::::::::::::::::::::::::::::::::::::::::::::::::::::::::::::::::::::::::::::::::::::::::::::::::::::::::::::::::::::::::::::::::::::::::::::::::::::::::::::::::::::::::::::::::::::::::::::::::::::::::::::

@D0950.29_112115

GATGAACGCTAGCGACAGGCCTAACACATGCAAGTCGAGGGGCAGCGGGAGTGTAGCAATACACTTGCCGGCGACCGGCGCACGGGTGAGTAACACGTATGCGACCTACCCATAGCAGGGGGATAATCGGAAGAAATTCCGTCTAATACCGCGTAATAATTCAGATCTGCATGGATTTGAATTTAAAGGAGCAAATCCGGCTATGGATGGGCATGCGGGACATTAGCTAGTTGGCGGGGTAACGGCCCACCAAGGCTTCGATGTCTAGGGGTTCTGAGAGGAAGGTCCCCCACACTGGTACTGAGACACGGACCAGACTCCTACGGGAGGCAGCAGTGAGGAATATTGGTCAATGGTCGAGAGACTGAACCAGCCAAGTCGCGTGAGGGATGAAGGTTCTATGGATTGTAAACCTCTTTTGTCAGGGAGCAACGACATCCACGAGTGGGTGAATGAGAGTACCTGAAGAAAAGCATCGGCTAACTCCGTGCCAGCAGCCGCGGTAA

+

::::::::::::::::::::::::::::::::::::::::::::::::::::::::::::::::::::::::::::::::::::::::::::::::::::::::::::::::::::::::::::::::::::::::::::::::::::::::::::::::::::::::::::::::::::::::::::::::::::::::::::::::::::::::::::::::::::::::::::::::::::::::::::::::::::::::::::::::::::::::::::::::::::::::::::::::::::::::::::::::::::::::::::::::::::::::::::::::::::::::::::::::::::::::::::::::::::::::::::::::::::::::::::::::::::::::::::::::::::::::::::::::::::::::::::::::::::::::::::::::::::::::::::::::::::::::::

@D0950.29_112132

GATGAACGCTAGCTACAGGCTTAACACATGCAAGTCGAGGGGTAGCACAAGGAAGCTTGCTTCTGAGGTGACGACCGGCGCACGGGTGAGTAACGCGTATGCAACCTACCTGTAAGAGTGGGATAGCCTCTCGAAAGAGAGATTAATACCGCATAATACCATTTCACTGCATGGTGAGATGGTTAAAGATTTATTGCTTACAGATGGGCATGCGTAACATTAGCTAGTTGGTGAGGTAACGGCTCACCAAGGCAACGATGTTTAGGGGTTCTGAGAGGAAGGTCCCCCACACTGGTACTGAGACACGGGACCAGACTCCTACGGGAGGCAGCAGTGAGGAATATTGGTCAATGGACGAGAGTCTGAACCAGCCAAGTCGCGTGAAGGATGAAGGTCTTATGGATTGTAAACTTCTTTTATACGGGGAATAAAAATGCCACGTGTGGCATATTGCATGTACCGTATGAATAAGGATCGGCTAACTCCGTGCCAGCAGCCGCGGTAA

+

:::::::::::::::::::::::::::::::::::::::::::::::::::::::::::::::::::::::::::::::::::::::::::::::::::::::::::::::::::::::::::::::::::::::::::::::::::::::::::::::::::::::::::::::::::::::::::::::::::::::::::::::::::::::::::::::::::::::::::::::::::::::::::::::::::::::::::::::::::::::::::::::::::::::::::::::::::::::::::::::::::::::::::::::::::::::::::::::::::::::::::::::::::::::::::::::::::::::::::::::::::::::::::::::::::::::::::::::::::::::::::::::::::::::::::::::::::::::::::::::::::::::::::::::::::::::::

@D0950.29_112170

GATGAACGCTGGCGGCGTGCTTAACACATGCAAGTCGAGCGAGGAATCACCTTCGGGTGTGAACTAGCGGCGGACGGGTGAGTAACACGTGGGCAACCTGCCTTACAGAGGGGGATAGCCTTCCGAAAGGAAGATTAATACCGCATATTATGAGTTTTCTGCATGGGGAATTCATGAAAGGAGTAATCCGCTGTAAGATGGGCCCGCGGCGCATTAGCTAGTTGGTGAGGTAACGGCTCACCAAGGCGACGATGCGTAGCCGACCTGAGAGGGTGATCGGCCACATTGGGACTGAGACACGGCCCAGACTCCTACGGGAGGCAGCAGTGGGGAATATTGCACAATGGGGGAAACCCTGATGCAGCAACGCCGCGTGAGTGATGAAGGCCTTCGGGTTGTAAAGCTCTGTCTTCAGGGACGATAATGACGGTACCTGAGGAGGAAGCCACGGCTAACTACGTGCCAGCAGCCGCGGTAA

+

::::::::::::::::::::::::::::::::::::::::::::::::::::::::::::::::::::::::::::::::::::::::::::::::::::::::::::::::::::::::::::::::::::::::::::::::::::::::::::::::::::::::::::::::::::::::::::::::::::::::::::::::::::::::::::::::::::::::::::::::::::::::::::::::::::::::::::::::::::::::::::::::::::::::::::::::::::::::::::::::::::::::::::::::::::::::::::::::::::::::::::::::::::::::::::::::::::::::::::::::::::::::::::::::::::::::::::::::::::::::::::::::::::::::::::::::::::::::::::::

@D0950.29_112255

GATGAACGCTAGCTACAGGCTTAACACATGCAAGTCGAGGGGTAGCATGAAACTTAGCAATAAGTTTTGATGACGACCGGCGCACGGGTGAGTAACACGTATCCAACCTGCCTTTTACTCATGGATAGCCTTCTGAAAAGAAGATTAATACATGATGGTATTCAGAGTTTTCATGGACACTGAATTAAAGATTTTATCGGTAAGAGATGGGGATGCGTTCCATTAGATAGTAGGCTGGGGTAACGGCCCACCTAGTCGAACGATCGGATAGGGGTTCTGAGAGGAAGGTCCCCCACATTGGAACTGAGACACGGTCCAAACGTCCTACGGGAGGCAGCAGTGAGGAATATTGGTCAATGGACGTAAGTCTGAACCAGCCAAGTAGCGTGAAGGATGAAGGCTCTATGGGTCGTAAACTTCTTTTTATAAAAGGG

+

::::::::::::::::::::::::::::::::::::::::::::::::::::::::::::::::::::::::::::::::::::::::::::::::::::::::::::::::::::::::::::::::::::::::::::::::::::::::::::::::::::::::::::::::::::::::::::::::::::::::::::::::::::::::::::::::::::::::::::::::::::::::::::::::::::::::::::::::::::::::::::::::::::::::::::::::::::::::::::::::::::::::::::::::::::::::::::::::::::::::::::::::::::::::::::::::::::::::::::::::::::::::::::::::::::::::::::::::::

@D0950.29_112258

GATGAACGCTAGCGACAGGCCTAACACATGCAAGTCGAGGGGTAGCACAAGGTAGCAATACTGAGGTGACGACCGGCGCACGGGTGAGTAACGCGTATGCAACCTACCTGTAAGAGTGGGATAGCCTCTCGAAAGAGAGATTAATACCGCATAATACCATTTCACTGCATGGTGAGATGGTTAAAGATTTATTGCTTACAGATGGGCATGCGTAACATTAGCTAGTTGGTGAGGTAACGGCTCACCAAGGCAACGATGTTTAGGGGTTCTGAGAGGAAGGTCCCCCACACTGGTACTGAGACACGGACCAGACTCCTACGGGAGGCAGCAGTGAGGAATATTGGTCAATGGACGAGAGTCTGAACCAGCCAAGTCGCGTGAAAGGATGAAGGTCTTATGGATTGTAAACTTCTTTTATACGGGAATAAAAAATGCCACGTGTGGCATATTGCATGTACCGT

+

:::::::::::::::::::::::::::::::::::::::::::::::::::::::::::::::::::::::::::::::::::::::::::::::::::::::::::::::::::::::::::::::::::::::::::::::::::::::::::::::::::::::::::::::::::::::::::::::::::::::::::::::::::::::::::::::::::::::::::::::::::::::::::::::::::::::::::::::::::::::::::::::::::::::::::::::::::::::::::::::::::::::::::::::::::::::::::::::::::::::::::::::::::::::::::::::::::::::::::::::::::::::::::::::::::::::::::::::::::::::::::::::::::::::::::::

@D0950.29_112264

GATGAACGCTAGCTACAGGCTTAACACATGCAAGTCGAGGGGTAGCATGAAACTTAGCAATAAGTTTTGATGACGACCGGCGCACGGGTGAGTAACACGTATCCAACCTGCCTTTTACTCATGGATAGCCTTCTGAAAAGAAGATTAATACATGATGGTATTCAGAGTTTTCATGGACACTGAATTAAAGATTTTATCGGTAAGAGATGGGGATGCGTTCCATTAGATAGTAGGCGGGGTAACGGCCCACCTAGTCAACGATGGATAGGGGTTCTGAGAGGAAGGTCCCCCACATTGGAACTGAGACACGGTCCAAACGTCCTACGGGAGGCAGCAGTGAGAATATTGGTCAATGGACGTAAGTCTGAACCAGCCAAGTAGCGTGAAGGATGAAGGCTCTATGGGTCGTAAACTTCTTTTTATAAAAGGAATAAAGTATGCCACGTGTGG

+

::::::::::::::::::::::::::::::::::::::::::::::::::::::::::::::::::::::::::::::::::::::::::::::::::::::::::::::::::::::::::::::::::::::::::::::::::::::::::::::::::::::::::::::::::::::::::::::::::::::::::::::::::::::::::::::::::::::::::::::::::::::::::::::::::::::::::::::::::::::::::::::::::::::::::::::::::::::::::::::::::::::::::::::::::::::::::::::::::::::::::::::::::::::::::::::::::::::::::::::::::::::::::::::::::::::::::::::::::::::::::::::::::

@D0950.29_112268

GATGAACGCTGGCGGCGTGCTTAACACATGCAAGTCGAACGAAGCAACTCTCTTGCTTGCAAGAGAGTTGACTTAGTGGCGGACGGGTGAGTAACGCGTGGGTAACCTGCCTCATACAGGGGATAACAGTTGGAAACGACTGCTAAGACCGCATAACCCGCTAGTGTCGCATGACACGGACGGAAAATATTTTATAGGTATGAGATGGGCCCGCGTCTGATTAGCTAGTTGGTAAGGTAACGGCTTACCAAGGCGACGATCAGTAGCCGACTTGAGAGAGTGATCGGCCACATTGGGACTGAGACACGGCCCAAACTCCTACGGGAGGCAGCAGTGGGGAATATTGCACAATGGGGGAAACCCTGATGCAGCAACGCCGCGTGAGTGAAGAAGTATTTCGGTATGTAAAGCTCTATCAGCAGGGAAGATAATGACGGTACCTGACTAAGAAGCCCCCGGCTAACTA

+

::::::::::::::::::::::::::::::::::::::::::::::::::::::::::::::::::::::::::::::::::::::::::::::::::::::::::::::::::::::::::::::::::::::::::::::::::::::::::::::::::::::::::::::::::::::::::::::::::::::::::::::::::::::::::::::::::::::::::::::::::::::::::::::::::::::::::::::::::::::::::::::::::::::::::::::::::::::::::::::::::::::::::::::::::::::::::::::::::::::::::::::::::::::::::::::::::::::::::::::::::::::::::::::::::::::::::::::::::::::::::::::::::::::::::::::::::

@D0950.29_112300

GATGAACGCTAGCGACAGGCCTAACACATGCAAGTCGAGGGGTAGCACAAGGAAGCTTGCTTCTGAGGTGACGACCGGCGCACGGGTGAGTAACGCGTATGCAACCTGCCTATAAGAAGGGGATAGCCTCTCGAAAGAGAGATTAATACCGTATAACACTATGAAGCCGCATGGTTTTACAGTTAAAGATTTATTGCTTATAGATGGGCATGCGTAACATTAGCTAGTTGGTGAGGTAACGGCTCACCAAGGCAACGATGTTTAGGGGTTCTGAGAGGAAGGTCCCCCACACTGGTACTGAGACACGGACCAGACTCCTACGGGAGGCAGCAGTGAGGAATATTGGTCAATGGACGAGAGTCTGAACCAGCCAAGTCGCGTGAAGGATGAAGGTCTTATGGATTGTAAACTTCTTTTATACGGGGAAATAAAAAGAGCCACGTGTGGCTTATTGCATGTACCGTATGAATAAGGATCGGCTAACTCCGTGCCAGCAGCCGC

+

:::::::::::::::::::::::::::::::::::::::::::::::::::::::::::::::::::::::::::::::::::::::::::::::::::::::::::::::::::::::::::::::::::::::::::::::::::::::::::::::::::::::::::::::::::::::::::::::::::::::::::::::::::::::::::::::::::::::::::::::::::::::::::::::::::::::::::::::::::::::::::::::::::::::::::::::::::::::::::::::::::::::::::::::::::::::::::::::::::::::::::::::::::::::::::::::::::::::::::::::::::::::::::::::::::::::::::::::::::::::::::::::::::::::::::::::::::::::::::::::::::::::::::::::::::::

@D0950.29_112310

GATGAACGCTAGCGACAGGCCTAACACATGCAAGTCGAGGGGTAGCACAAGGAAGCTTGCTTCTGAGGTGACGACCGGCGCACGGGTGAGTAACGCGTATGCAACCTACCTGTAAGAGTGGGATAGCCTCTCGAAAGAGAGATTAATACCGCATAATACCATTTCACTGCATGGTGAGATGGTTAAAGATTTATTGCTTACAGATGGGCATGCGTAACATTAGCTAGTTGGTGAGGTAACGGCTCACCAAGGCAACGATGTTTAGGGGTTCTGAGAGGAAGGTCCCCCGACACTGGTACTGAGACACGGACCAGACTCCTACGGGAGGCAGCAGTGAGGAATATTGGTCAATGGACGAGAGTCTGAACCAGCCAAGTCGCGTGAAGGATGAAGGTCTTATGGATTGTAAACTTCTTTTATACGGGAATAAAAATGCCACGTGTGGTTTATTGCATGTACCGTATGAATAAGGATCGGCTAACTCCGTGCCAGCAGCCGCGGTAA

+

::::::::::::::::::::::::::::::::::::::::::::::::::::::::::::::::::::::::::::::::::::::::::::::::::::::::::::::::::::::::::::::::::::::::::::::::::::::::::::::::::::::::::::::::::::::::::::::::::::::::::::::::::::::::::::::::::::::::::::::::::::::::::::::::::::::::::::::::::::::::::::::::::::::::::::::::::::::::::::::::::::::::::::::::::::::::::::::::::::::::::::::::::::::::::::::::::::::::::::::::::::::::::::::::::::::::::::::::::::::::::::::::::::::::::::::::::::::::::::::::::::::::::::::::::::::::

@D0950.29_112325

GATGAACGCTAGCTACAGGCTTAACACATGCAAGTCGAGGGGTAGCATGAAACTTAGCAATAAGTTTTGATGACGACCGGCGCACGGGTGAGTAACACGTATCCAACCTGCCTTTTACTCATGGATAGCCTTCTGAAAAGAAGATTAATACATGATGGTATTCAGAGTTTTCATGGACACTGAATTAAAGATTTTATCGGTAAGAGATGGGGATGCGTTCCATTAGATAGTAGGCGGGGTAACGGCCCACCTAGTCAACGATGGATAGGGGTTCCTGAGAGGAAGGTCCCCCACATTGGAACTGAGACACGGTCCAAACGTCCTACGGGAGGCAGCAGTGAGGAATATTGGTCCAATGGACGTAAGTCTGAACCAGCCAAGTAGCGTGAAGGATGAAAGGCTCTATGGGTCGTAAACTTCTTTTTATAAAAGG

+

:::::::::::::::::::::::::::::::::::::::::::::::::::::::::::::::::::::::::::::::::::::::::::::::::::::::::::::::::::::::::::::::::::::::::::::::::::::::::::::::::::::::::::::::::::::::::::::::::::::::::::::::::::::::::::::::::::::::::::::::::::::::::::::::::::::::::::::::::::::::::::::::::::::::::::::::::::::::::::::::::::::::::::::::::::::::::::::::::::::::::::::::::::::::::::::::::::::::::::::::::::::::::::::::::::::::::::::::::

@D0950.29_112334

GATGAACGCTAGCGACAGGCCTAACACATGCAAGTCGAGGGGTAGCACAAGGTAGCAATACTGAGGTGACGACCGGCGCACGGGTGAGTAACGCGTATGCAACCTACCTGTAAGAGTGGGATAGCCTCTCGAAAGAGAGATTAATACCGCATAATACCATTTCACTGCATGGTGAGATGGTTAAAGATTTATTGCTTACAGATGGGCATGCGTAACATTAGCTTGTTGGTGAGGTAACGGCTCACCAAGGCAACGATGTTTAGGGGTTCTGAGAGGAAGGTCCCCCACACTGGTACTGAGACACGGACCAGACTCCTACGGGAGGCAGCAGTGAGGAATATTGGTCAATGGACGAGAGTCTGAACCAGCCAAGTCGCGTGAAGGATGAAGGTCTTATGGATTGTAAACTTCTTTTATACGGGAATAAAAAATGCCACGTGTGGCATATTGCATGTACC

+

::::::::::::::::::::::::::::::::::::::::::::::::::::::::::::::::::::::::::::::::::::::::::::::::::::::::::::::::::::::::::::::::::::::::::::::::::::::::::::::::::::::::::::::::::::::::::::::::::::::::::::::::::::::::::::::::::::::::::::::::::::::::::::::::::::::::::::::::::::::::::::::::::::::::::::::::::::::::::::::::::::::::::::::::::::::::::::::::::::::::::::::::::::::::::::::::::::::::::::::::::::::::::::::::::::::::::::::::::::::::::::::::::::::::::

@D0950.29_112341

GATGAACGCTAGCGACAGGCCTAACACATGCAAGTCGAGGGGTAGCACAAGGTAGTAATACTGAGGTGACGACCGGCGCACGGGTGAGTAACGCGTATGCAACCTACCTGTAAGAGTGGGATAGCCTCTCGAAAGAGAGATTAATACCGCATAATACCATTTCACTGCATGGTGAGATGGTTAAAGATTTATTGCTTACAGATGGGCATGCGTAACATTAGCTAGTTGGTGAGGTAACGGCTCACCAAGGCAACGATGTTTAGGGGTTCTGAGAGGAAGGTCCCCCACACTGGTACTGAGACACGGACCAGACTCCTACGGGAGGCAGCAGTGAGGAATATTGGTCAATGGACGAGAGTCTGAACCAGCCAAGTCGCGTGAAGGATGAAGGTCTTATGGATTGTAAACTTCTTTTATACGGGAATAAAAAATGCCACGTGTGGCATATTGCATGTACCGTATGAATAAGGATCGGCT

+

:::::::::::::::::::::::::::::::::::::::::::::::::::::::::::::::::::::::::::::::::::::::::::::::::::::::::::::::::::::::::::::::::::::::::::::::::::::::::::::::::::::::::::::::::::::::::::::::::::::::::::::::::::::::::::::::::::::::::::::::::::::::::::::::::::::::::::::::::::::::::::::::::::::::::::::::::::::::::::::::::::::::::::::::::::::::::::::::::::::::::::::::::::::::::::::::::::::::::::::::::::::::::::::::::::::::::::::::::::::::::::::::::::::::::::::::::::::::::::::

@D0950.29_112353

GATGAACGCTAGCTACAGGCTTAACACATGCAAGTCGAGGGGTAGCATGAAACTTAGCAATAAGTTTTGATGACGACCGGCGCACGGGTGAGTAACACGTATCCAACCTGCCTTTTACTCATGGATAGCCTTCTGAAAAGAAGATTAATACATGATGGTATTCAGAGTTTTCATGGACACTGAATTAAAGATTTTATCGGTAAGAGATGGGGATGCGTTCCATTAGATAGTAGGCGGGGTAACGGCCCACCTAGTCAACGATGGATAGGGGTTCTGAGAGGAAGGTCCCCACATTGGAACTGAGACACGGTCCAAACGTCCTACGGGAGGCAGCAGTGAGGAATATTGGTCAATGGACGTAAGTCTGAACCAGCCAAGTAGCGTGAAGGATGAAGGCTCTATGGGTCGTAAACTTCTTTTATAAAAGGAATAAAGTATGCCACGTGTGGTGTTTTTGTATGTACTTTATGAATAAGGATCGGCTAACTCCGTGCCAGCAGCCGC

+

::::::::::::::::::::::::::::::::::::::::::::::::::::::::::::::::::::::::::::::::::::::::::::::::::::::::::::::::::::::::::::::::::::::::::::::::::::::::::::::::::::::::::::::::::::::::::::::::::::::::::::::::::::::::::::::::::::::::::::::::::::::::::::::::::::::::::::::::::::::::::::::::::::::::::::::::::::::::::::::::::::::::::::::::::::::::::::::::::::::::::::::::::::::::::::::::::::::::::::::::::::::::::::::::::::::::::::::::::::::::::::::::::::::::::::::::::::::::::::::::::::::::::::::::::::::::

@D0950.29_112378

GATGAACGCTGGCGGCGTGCTTAACACATGCAAGTCGAACGAAGCAACTCTCTTGCTTGCAAGAGAGTTGACTTAGTGGCGGACGGGTGAGTAACGCGTGGGTAACCTGCCTCATACAGGGGATAACAGTTGGAAACGACTGCTAAGACCGCATAACCCGCTAGTGTCGCATGACACGGACGGAAAATATTTATAGGTATGAGATGGGCCCGCGTCTGATTAGCTAGTTGGTAAGGTAACGGCTTACCAAGGCGACGATCAGTAGCCGACTTGAGAGAGTGATCGGCCACATTGGGACTGAGACACGGCCCAAACTCCTACGGGAGGCAGCAGTGGGGAATATTGGACAATGGGGGAAACCCTGATCCAGCGACGCCGCGTGAGTGAAGAAGTATTTCGGTATGTAAAGCTCTATCAGCAGGGAAGATAATGACAGTACCTGACTAAGAAGCCCCCGGCTAACTACGTGCCAGCAGCCGC

+

::::::::::::::::::::::::::::::::::::::::::::::::::::::::::::::::::::::::::::::::::::::::::::::::::::::::::::::::::::::::::::::::::::::::::::::::::::::::::::::::::::::::::::::::::::::::::::::::::::::::::::::::::::::::::::::::::::::::::::::::::::::::::::::::::::::::::::::::::::::::::::::::::::::::::::::::::::::::::::::::::::::::::::::::::::::::::::::::::::::::::::::::::::::::::::::::::::::::::::::::::::::::::::::::::::::::::::::::::::::::::::::::::::::::::::::::::::::::::::::::

@D0950.29_112420

GATGAACGCTAGCGACAGGCCTAACACATGCAAGTCGAGGGGTAGCACAAGGTAGCAATACTGAGGTGACGACCGGCGCACGGGTGAGTAACGCGTATGCAACCTACCTGTAAGAGTGGGATAGCCTCTCGAAAGAGAGATTAATACCGCATAATACCATTTCACTGCATGGTGAGATGGTTAAAGATTTATTGCTTACAGATGGGCATGCGTAACATTAGCTAGTTGGTGAGGTAACGGCTCACCAAGGCAACGATCGTTTAGGGGTTCTGAGAGGAAGGTCCCCCACACTGGTACTGGAGACACGGACCAGACTCCTACGGGAGGCAGCAGTGAGGAATATTGGTCAATGGACGTAAGTCTGAACCAGCCAAGTAGCGTGAAGGATGAAGGCTCTATGGGTCGTAAACTTCTTTTATAAGGATAAAGTATGCCAACGTGTGGGTGTTTTGTATGTACTTTATGAAT

+

::::::::::::::::::::::::::::::::::::::::::::::::::::::::::::::::::::::::::::::::::::::::::::::::::::::::::::::::::::::::::::::::::::::::::::::::::::::::::::::::::::::::::::::::::::::::::::::::::::::::::::::::::::::::::::::::::::::::::::::::::::::::::::::::::::::::::::::::::::::::::::::::::::::::::::::::::::::::::::::::::::::::::::::::::::::::::::::::::::::::::::::::::::::::::::::::::::::::::::::::::::::::::::::::::::::::::::::::::::::::::::::::::::::::::::::::::::

@D0950.29_112478

GATGAACGCTGGCGGCGTGCCTAACACATGCAAGTCGAGCGATTCTCTTCGGAGAAGAGCGGCGGACGGGTGAGTAACGCGTGGGTAACCTGCCCTGTACACACGGATAACATACCGAAAGGTATGCTAATACGGGATAACATGAGAAAGTCGCATGGCTTTCTTATCAAAGCTCCGGCGGTACAGGATGGACCCGCGTCTGATTAGCTAGTTGGTAAGGTAACGGCTTACCAAGGCGACGATCAGTAGCCGACCTGAGAGGGTGATCGGCCACATTGGAACTGAGACACGGTCCAAACTCCTACGGGAGGCAGCAGTGGGGAATATTGCACAATGGGGGAAACCCTGATGCAGCAAACGCCGCGTGAGTGATGACGGCCTCGGGTTGTAAAA

+

:::::::::::::::::::::::::::::::::::::::::::::::::::::::::::::::::::::::::::::::::::::::::::::::::::::::::::::::::::::::::::::::::::::::::::::::::::::::::::::::::::::::::::::::::::::::::::::::::::::::::::::::::::::::::::::::::::::::::::::::::::::::::::::::::::::::::::::::::::::::::::::::::::::::::::::::::::::::::::::::::::::::::::::::::::::::::::::::::::::::::::::::::::::::::::::::::::::::::

@D0950.29_112569

ATTGAACGCTGGCGGCAGGCCTAACACATGCAAGTCGAACGGTAGCACAGAGAGCTTGCTCTTGGGTGACGAGTGGCGGACGGGTGAGTAATGTCTGGGAAACTGCCCGATGGAGGGGGATAACTACTGGAAACGGTAGCTAATACCGCATAATGTCGCAAGACCAAAGAGGGGGACCTTCGGGCCTCTTGCCATCGGATGTGCCCAGATGGGATTAGCTAGTAGGTGGGGTAATGGCTCACCTAGGCGACGATCCCTAGCTGGTCTGAGAGGATGACCAGCCACACTGGAACTGAGACACGGTCCAGACTCCTACGGGAGGCAGCAGTGGGGAATATTGCACAATGGGCGCAAGCCTGATGCAGCCATGCCGCGTGTATGAAGAAGGCCTTCGGGTTGTAAAGTACTTTCAGCGAGGAGGAAGGTGTTGAGGTTAATAACCTCAGCAATTGACGTTACTCGCAGAAGAAGCACCGGCTAACTCCGTGCCAGCAGCCGCGGTAA

+

::::::::::::::::::::::::::::::::::::::::::::::::::::::::::::::::::::::::::::::::::::::::::::::::::::::::::::::::::::::::::::::::::::::::::::::::::::::::::::::::::::::::::::::::::::::::::::::::::::::::::::::::::::::::::::::::::::::::::::::::::::::::::::::::::::::::::::::::::::::::::::::::::::::::::::::::::::::::::::::::::::::::::::::::::::::::::::::::::::::::::::::::::::::::::::::::::::::::::::::::::::::::::::::::::::::::::::::::::::::::::::::::::::::::::::::::::::::::::::::::::::::::::::::::::::::::

@D0950.29_112571

GATGAACGCTAGCGACAGGCCTAACACATGCAAGTCGAGGGGTAGCACAAGGAAGCTTGCTTCTGAGGTGACGACCGGCGCACGGGTGAGTAACGCGTATGCAACCTACCTGTAAGAGTGGGATAGCCTCTCGAAAGAGAGATTAATACCGCATAATACCATTTCACTGCATGGTGAGATGGTTAAAGATTTATTGCTTACAGATGGGCATGCGTAACATTAGCTAGTTGGTGAGGTAACGGCTCACCAAGGCAACGATGTTTAGGGGTTCTGAGAGGAAGGTCCCCCGACACTGGTACTGAGACACGGACCAGACTCCTACGGGAGGCAGCAGTGAGGAATATTGGTCAATGGACGAGAGTCTGAACCAGCCAAGTCGCGTGAAGGATGAAGGTCTTATGGATTGTAAACTTCTTTTATACGGGAATAAAAAATGCCCACGTGTGGCATATTGCATGTACCGTATGAATAAGGATCGGCTAACTCCGTGCCAGCAGCCGCGGTAA

+

::::::::::::::::::::::::::::::::::::::::::::::::::::::::::::::::::::::::::::::::::::::::::::::::::::::::::::::::::::::::::::::::::::::::::::::::::::::::::::::::::::::::::::::::::::::::::::::::::::::::::::::::::::::::::::::::::::::::::::::::::::::::::::::::::::::::::::::::::::::::::::::::::::::::::::::::::::::::::::::::::::::::::::::::::::::::::::::::::::::::::::::::::::::::::::::::::::::::::::::::::::::::::::::::::::::::::::::::::::::::::::::::::::::::::::::::::::::::::::::::::::::::::::::::::::::::::

@D0950.29_1126

ATTGAACGCTGGCGGCAGGCCTAACACATGCAAGTCGAACGGTAGCACAGAGAGCTTGCTCTCGGGTGACGAGTGGCGGACGGGTGAGTAATGTCTGGGAAACTGCCCGATGGAGGGGGATAACTACTGGAAACGGTAGCTAATACCGCATAACGTCGCAAGACCAAAGAGGGGACCTTCGGGCCTCTTGCCATCGGATGTGCCCAGATGGGATTAGCTAGTAGGTGGGGTAATGGCTCACCTAGGCGACGATCCCTAGCTGGTCTGAGAGGATGACCAGCCACACTGGAACTGAGACACGGTCCAGACTCCTACGGGAGGCAGCAGTGGGGAATATTGCACAATGGGCGCAAGCCTGATGCAGCCATGCCGCGTGTATGAAGAAGGCC

+

:::::::::::::::::::::::::::::::::::::::::::::::::::::::::::::::::::::::::::::::::::::::::::::::::::::::::::::::::::::::::::::::::::::::::::::::::::::::::::::::::::::::::::::::::::::::::::::::::::::::::::::::::::::::::::::::::::::::::::::::::::::::::::::::::::::::::::::::::::::::::::::::::::::::::::::::::::::::::::::::::::::::::::::::::::::::::::::::::::::::::::::::::::::::::::::::::::::

@D0950.29_112629

GATGAACGCTAGCGACAGGCCTAACACATGCAAGTCGAGGGGTAGCACAAGGAAGCTTGCTTCTGAGGTGACGACCGGCGCACGGGTGAGTAACGCGTATGCAACCTGCCTATAAGAAGGGGATAGCCTCTCGAAAGAGAGATTAATACCGTATAACACTATGAAACCGCATGGTTTTATAGTTAAAGATTTATTGCTTATAGATGGGCATGCGTAACATTAGCTAGTTGGTAAGGTCACGGCTTACCAAGGCAACGATGTTTAGGGGTTCTGAGAGGAAGGTCCCCCACACTGGTACTGAGACACGGACCAGACTCCTACGGGAGGCAGCAGTGAGGAATATTGGTCAATGGACGAGAGTCTGAACCAGCCAAGTCGCGTGAAGGATGAAGGTCTTATGGATTGTAAACTTCTTTTATACGGGGAAATAAAAAGAGCCACGTGTGGCTTATTGCATGTAGCGTATGAATAAGGATCGGCTAACTCCGTGCCAGCAGCCGC

+

:::::::::::::::::::::::::::::::::::::::::::::::::::::::::::::::::::::::::::::::::::::::::::::::::::::::::::::::::::::::::::::::::::::::::::::::::::::::::::::::::::::::::::::::::::::::::::::::::::::::::::::::::::::::::::::::::::::::::::::::::::::::::::::::::::::::::::::::::::::::::::::::::::::::::::::::::::::::::::::::::::::::::::::::::::::::::::::::::::::::::::::::::::::::::::::::::::::::::::::::::::::::::::::::::::::::::::::::::::::::::::::::::::::::::::::::::::::::::::::::::::::::::::::::::::::

@D0950.29_112639

GATGAACGCTAGCGACAGGCCTAACACATGCAAGTCGAGGGGTAGCACAAGGTAGTAATACTGAGGTGACGACCGGCGCACGGGTGAGTAACGCGTATGCAACCTACCTGTAAGAGTGGGATAGCCTCTCGAAAGAGAGATTAATACCGCATAATACCATTTCACTGCATGGTGAGATGGTTAAAGATTTATTGCTTACAGATGGGCATGCGTAACATTAGCTAGTTGGTGAGGTAACGGCTCACCAAGGCAACGATGTTTAGGGGTTCTGAGAGGAAGGTCCCCCACACTGGTACTGAGACACGGACCAGACTCCTACGGGAGGCAGCAGTGAGGAATATTGGTCAATGGACGAGAGTCTGAACCAGCCAAGTCGCGTGAAAGGATGAAGGTCTTATGGATTGTAAACTTCTTTTATACGGGAATAAAAAATGCCACGTGTGGCATATTGCATGTACCGTATGAATAAGGATCGGCT

+

::::::::::::::::::::::::::::::::::::::::::::::::::::::::::::::::::::::::::::::::::::::::::::::::::::::::::::::::::::::::::::::::::::::::::::::::::::::::::::::::::::::::::::::::::::::::::::::::::::::::::::::::::::::::::::::::::::::::::::::::::::::::::::::::::::::::::::::::::::::::::::::::::::::::::::::::::::::::::::::::::::::::::::::::::::::::::::::::::::::::::::::::::::::::::::::::::::::::::::::::::::::::::::::::::::::::::::::::::::::::::::::::::::::::::::::::::::::::::::::

@D0950.29_112648

GATGAACGCTGGCGGCGTGCTTAACACATGCAAGTCGAGCGAGGAATCACCTTCGGGTGTGAACTAGCGGCGGACGGGTGAGTAACACGTGGGCAACCTGCCTTACAGAGGGGGATAGCCTTCCGAAAGGAAGATTAATACCGCATATTATGAGTTTTCTGCATGGGGAATTCATGAAAGGAGTAATCCGCTGTAAGATGGGCCCGCGGCGCATTAGCTAGTTGGTGAGGTAACGGCTCACCAAGGCGACGATGCGTAGCCGACCTGAGAGGGTGATCGGCCACATTGGGACTGAGACACGGCCCAGACTCCTACGGGAGGCAGCAGTGGGGAATATTGCACAATGGGGGAAACCCTGATGCAGCAACGCCGCGTGAGTGAAGAAGTATTTCGGTATGTAAAGCTCTATCAGCAGGGAAGATAATGACAGTACCTGACTAAGAAGCCCCGGCTAACTACGTGCCAGCAGCCGCGGTAA

+

::::::::::::::::::::::::::::::::::::::::::::::::::::::::::::::::::::::::::::::::::::::::::::::::::::::::::::::::::::::::::::::::::::::::::::::::::::::::::::::::::::::::::::::::::::::::::::::::::::::::::::::::::::::::::::::::::::::::::::::::::::::::::::::::::::::::::::::::::::::::::::::::::::::::::::::::::::::::::::::::::::::::::::::::::::::::::::::::::::::::::::::::::::::::::::::::::::::::::::::::::::::::::::::::::::::::::::::::::::::::::::::::::::::::::::::::::::::::::::::

@D0950.29_112701

GATGAACGCTGGCGGCGTGCTTAACACATGCAAGTCGAACGAAGCAGCTTTCTTGCTTGCAAGAAAGCTGACTTAGTGGCGGACGGGTGAGTAACGCGTGGGTAACCTGCCTCATACAGGGGGATAACAGTTGGAAACGACTGCTAAGACCGCATAACCCGCTAGTGTCGCATGACACGGACGGAAAAATATTTATAGGTATGAGATGGGCCCGCGTCTGATTAGCTAGTTGGTAAGGTAACGGCTTACCAAGGCGACGATCAGTAGCCGACTTGAGAGAGTGATCGGCCACATTGGGACTGAGACACGGCCCAAACTCCTACGGGAGGCAGCAGTGGGGAATATTGGACAATGGGGGAAAACCCTGATCCAGCGACGCCGCGTGAGTGAAGAAAGTATTTTCGGTATGTAAAAGCTCTATCAGCAGGGAAAGATAATGACAGTACCTGACTAAAGAAAGCCCCCGGCTAAACTACGTGCCAGCAGCCGC

+

::::::::::::::::::::::::::::::::::::::::::::::::::::::::::::::::::::::::::::::::::::::::::::::::::::::::::::::::::::::::::::::::::::::::::::::::::::::::::::::::::::::::::::::::::::::::::::::::::::::::::::::::::::::::::::::::::::::::::::::::::::::::::::::::::::::::::::::::::::::::::::::::::::::::::::::::::::::::::::::::::::::::::::::::::::::::::::::::::::::::::::::::::::::::::::::::::::::::::::::::::::::::::::::::::::::::::::::::::::::::::::::::::::::::::::::::::::::::::::::::::::::::::

@D0950.29_112718

ATTGAACGCTGGCGGCATGCCTTACACATGCAAGTCGAACGGTAACAGGTCTTCGGATGCTGACGAGTGGCGAACGGGTGAGTAATACATCGGAACGTGCCCGATCGTGGGGGATAACGAAGCGAAAGCTTTGCTAATACCGCATACGATCTACGGATGAAAGCAGGGGACCGCAAGGCCTTGCGCGAACGGAGCGGCCGATGGCAGATTAGGTAGTTGGTGGGATAAAAGCTTACCAAGCCGACGATCTGTAGCTGGTCTGAGAGGACGACCAGCCACACTGGGACTGAGACACGGCCCAGACTCCTACGGGAGGCAGCAGTGGGGAATTTTGGACAATGGGCGAAAGCCTGATCCAGCCATGCCGCGTGCAGGATGAAGGCCTTCGGGTTGTAAACTGCTTTTGTACGGAACGAAAAGACTCTGGTTAATACCTGGGGGTCCATGACGGTACCGTAAGAATAAGCACCGGCTAACTACGTGCCAGCAGCCGCGGTAA

+

:::::::::::::::::::::::::::::::::::::::::::::::::::::::::::::::::::::::::::::::::::::::::::::::::::::::::::::::::::::::::::::::::::::::::::::::::::::::::::::::::::::::::::::::::::::::::::::::::::::::::::::::::::::::::::::::::::::::::::::::::::::::::::::::::::::::::::::::::::::::::::::::::::::::::::::::::::::::::::::::::::::::::::::::::::::::::::::::::::::::::::::::::::::::::::::::::::::::::::::::::::::::::::::::::::::::::::::::::::::::::::::::::::::::::::::::::::::::::::::::::::::::::::::::::::

@D0950.29_112769

GATGAACGCTAGCTACAGGCTTAACACATGCAAGTCGAGGGGTAGCATGAAACTTAGCAATAAGTTTTGATGACGACCGGCGCACGGGTGAGTAACACGTATCCAACCTGCCTTTTACTCATGGATAGCCTTCTGAAAAGAAGATTAATACATGATGGTATTCAGAGTTTTCATGGACACTGAATTAAAGATTTATCGGTAAGAGATGGGGATGCGTTCCATTAGATAGTAGGCGGGGTAACGGCCCACCTAGTCAACGATGGATAGGGGTTCTGAGAGGAAGGTCCCCCACACTGGTACTGAGACACGGACCAGACTCCTACGGGAGGCAGCAGTGAGGAATATTGGTCAATGGACGAGAGTCTGAACCAGCCAAGTCGCGTGAAGGATGAAGGTCTTATGGATTGTAAACTTC

+

:::::::::::::::::::::::::::::::::::::::::::::::::::::::::::::::::::::::::::::::::::::::::::::::::::::::::::::::::::::::::::::::::::::::::::::::::::::::::::::::::::::::::::::::::::::::::::::::::::::::::::::::::::::::::::::::::::::::::::::::::::::::::::::::::::::::::::::::::::::::::::::::::::::::::::::::::::::::::::::::::::::::::::::::::::::::::::::::::::::::::::::::::::::::::::::::::::::::::::::::::::::::::::::::

@D0950.29_112773

GATGAACGCTAGCGACAGGCCTAACACATGCAAGTCGAGGGGTAGCACAAGGTAGCAATACTGAGGTGACGACCGGCGCACGGGTGAGTAACGCGTATGCAACCTACCTGTAAGAGTGGGATAGCCTCTCGAAAGAGAGATTAATACCGCATAATACCATTTCACTGCATGGTGAGATGGTTAAAGATTTATTGCTTACAGATGGGCATGCGTAACATTAGCTAGTTGGTGAGGTAACGGCTCACCAAGGCAACGATGTTTAGGGGTTCTGAGAGGAAGGTCCCCCACACTGGTACTGAGACACGGACCAGACTCCTACGGGAGGCAGCAGTGAGGAATATTGGTCAATGGACGAGAGTCTGAACCAGCCAAGTCGCGTGAAAGGATGAAGGTCTTATGGATTGTAAACTTCTTTTATACGGGAATAAAAAATGCCACGTGTGGCATATTGCATGTACCGTATGAATAAGGATCGGCTAACTCCGTGCCAGCAGCCGCGGTAA

+

:::::::::::::::::::::::::::::::::::::::::::::::::::::::::::::::::::::::::::::::::::::::::::::::::::::::::::::::::::::::::::::::::::::::::::::::::::::::::::::::::::::::::::::::::::::::::::::::::::::::::::::::::::::::::::::::::::::::::::::::::::::::::::::::::::::::::::::::::::::::::::::::::::::::::::::::::::::::::::::::::::::::::::::::::::::::::::::::::::::::::::::::::::::::::::::::::::::::::::::::::::::::::::::::::::::::::::::::::::::::::::::::::::::::::::::::::::::::::::::::::::::::::::::::::::::::

@D0950.29_112783

GATGAACGCTAGCGACAGGCCTAACACATGCAAGTCGAGGGGTAGCACAAGGTAGCAATACTGAGGTGACGACCGGCGCACGGGTGAGTAACGCGTATGCAACCTACCTGTAAGAGTGGGATAGCCTCTCGAAAGAGAGATTAATACCGCATAATACCATTTCACTGCATGGTGAGATGGTTAAAGATTTATTGCTTACAGATGGGCATGCGTAACATTAGCTAGTTGGTGAGGTAACGGCTCACCAAGGCAACGATGTTTAGGGGTTCTGAGAGGAAGGTCCCCCGACACTGGTACTGAGACACGGACCAGACTCCTACGGGAGGCAGCAGTGAGGAATATTGGTCAATGGACGAGAGTCTGAACCAGCCAAGTCGCGTGAAGGATGAAGGTCTTATGGATTGTAAACTTCTTTTATACGGGAATAAAAATGCCACGTGTGGCATATTGCATGTACCGTATGAATAAGGATCGGCTAACTCCGTGCCAGCAGCCGCGGTAA

+

::::::::::::::::::::::::::::::::::::::::::::::::::::::::::::::::::::::::::::::::::::::::::::::::::::::::::::::::::::::::::::::::::::::::::::::::::::::::::::::::::::::::::::::::::::::::::::::::::::::::::::::::::::::::::::::::::::::::::::::::::::::::::::::::::::::::::::::::::::::::::::::::::::::::::::::::::::::::::::::::::::::::::::::::::::::::::::::::::::::::::::::::::::::::::::::::::::::::::::::::::::::::::::::::::::::::::::::::::::::::::::::::::::::::::::::::::::::::::::::::::::::::::::::::::::::

@D0950.29_11284

GATGAACGCTAGCTACAGGCTTAACACATGCAAGTCGAGGGGTAGCATGAAACTTAGCAATAAGTTTTGATGACGACCGGCGCACGGGTGAGTAACACGTATCCAACCTGCCTTTTACTCATGGATAGCCTTCTGAAAAGAAGATTAATACATGATGGTATTCAGAGTTTTCATGGACACTGAATTAAAGATTTTATCGGTAAGAGATGGGGATGCGTTCCATTAGATAGTAGGCGGGGTAACGGCCCACCTAGTCAACGATGGATAGGGGTTCTGAGAGGAAGGTCCCCCACATTGGAACTGAGACACGGTCCAAACGTCCTACGGGAGGCAGCAGTGAGGAATATTGGTCAATGGACGTAAGTCTGAACCAGCCAAGTAGCGTGAAGGATGAAGGCTCTATGGGTCGTAAACTTCTTTTTATAAAAGG

+

::::::::::::::::::::::::::::::::::::::::::::::::::::::::::::::::::::::::::::::::::::::::::::::::::::::::::::::::::::::::::::::::::::::::::::::::::::::::::::::::::::::::::::::::::::::::::::::::::::::::::::::::::::::::::::::::::::::::::::::::::::::::::::::::::::::::::::::::::::::::::::::::::::::::::::::::::::::::::::::::::::::::::::::::::::::::::::::::::::::::::::::::::::::::::::::::::::::::::::::::::::::::::::::::::::::::::::::

@D0950.29_112852

GATGAACGCTAGCGACAGGCCTAACACATGCAAGTCGAGGGGTAGCACAAGGAAGCTTGCTTCTGAGGTGACGACCGGCGCACGGGTGAGTAACGCGTATGCAACCTACCTGTAAGAGTGGGATAGCCTCTCGAAAGAGAGATTAATACCGCATAATACCATTTCACTGCATGGTGAGATGGTTAAAGATTTATTGCTTACAGATGGGCATGCGTAACATTAGCTAGTTGGTGAGGTAACGGCTCACCAAGGCAACGATGTTTAGGGGTTCTGAGAGGAAGGTCCCCCGACACTGGTACTGAGACACGGACCAGACTCCTACGGGAGGCAGCAGTGAGGAATATTGGTCAATGGACGAGAGTCTGAACCAGCCAAGTCGCGTGAAGGATGAAGGTCTTATGGATTGTAAACTTCTTTTATACGGGAATAAAAATGCCACGTGTGGCATATTGCATGTACCGTATGAATAAGGATCGGCTAACTCCGTGCCAGCAGCCGCGGT

+

::::::::::::::::::::::::::::::::::::::::::::::::::::::::::::::::::::::::::::::::::::::::::::::::::::::::::::::::::::::::::::::::::::::::::::::::::::::::::::::::::::::::::::::::::::::::::::::::::::::::::::::::::::::::::::::::::::::::::::::::::::::::::::::::::::::::::::::::::::::::::::::::::::::::::::::::::::::::::::::::::::::::::::::::::::::::::::::::::::::::::::::::::::::::::::::::::::::::::::::::::::::::::::::::::::::::::::::::::::::::::::::::::::::::::::::::::::::::::::::::::::::::::::::::::::::

@D0950.29_11287

GATGAACGCTAGCGACAGGCCTAACACATGCAAGTCGAGGGGTAGCACAAGGAAGCTTGCTTCTGAGGTGACGACCGGCGCACGGGTGAGTAACGCGTATGCAACCTACCTGTAAGAGTGGGATAGCCTCTCGAAAGAGAGATTAATACCGCATAATACCATTTCACTGCATGGTGAGATGGTTAAAGATTTATTGCTTACAGATGGGCATGCGTAACATTAGCTAGTTGGTGAGGTAACGGCTCACCAAGGCAACGATGTTTAGGGGTTCTGAGAGGAAGGTCCCCCACACTGGTACTGAGACACGGACCAGACTCCTACGGGAGGCAGCAGTGAGGAATATTGGTCAATGGACGAGAGTCTGAACCAGCCAAGTCGCGTGAAGGATGAAGGTCTTATGGATTGTAAACTTCTTTTATACGGGAATAAAAATGCCACGTGTGG

+

::::::::::::::::::::::::::::::::::::::::::::::::::::::::::::::::::::::::::::::::::::::::::::::::::::::::::::::::::::::::::::::::::::::::::::::::::::::::::::::::::::::::::::::::::::::::::::::::::::::::::::::::::::::::::::::::::::::::::::::::::::::::::::::::::::::::::::::::::::::::::::::::::::::::::::::::::::::::::::::::::::::::::::::::::::::::::::::::::::::::::::::::::::::::::::::::::::::::::::::::::::::::::::::::::::::::::::::::::::::::::::

@D0950.29_112870

GATGAACGCTAGCTACAGGCTTAACACATGCAAGTCGAGGGGTAGCATGAAACTTAGCAATAAGTTTTGATGACGACCGGCGCACGGGTGAGTAACACGTATCCAACCTGCCTTTTACTCATGGATAGCCTTCTGAAAAGAAGATTAATACATGATGGTATTCAGAGTTTTCATGGACACTGAATTAAAGATTTTATCGGTAAAGAGATGGGGATGCGTTCCATTAGATAGTAGGCGGGGTAACGGCCCACCTAGTCAACGATGGATAGGGGTTCTGAGAGGAAGGTCCCCACATTGGAACTGAGACACGGTCCAAACGTCCTACGGGAGGGCAGCAGTGAGGAATATTGGTCAATGGACGTAAGTCTGAACCAGCCAAGTAGCGTGAAGGATGAAGGCTCTATGGGTCGTAAAACTTCTTTTTATAAAAGG

+

::::::::::::::::::::::::::::::::::::::::::::::::::::::::::::::::::::::::::::::::::::::::::::::::::::::::::::::::::::::::::::::::::::::::::::::::::::::::::::::::::::::::::::::::::::::::::::::::::::::::::::::::::::::::::::::::::::::::::::::::::::::::::::::::::::::::::::::::::::::::::::::::::::::::::::::::::::::::::::::::::::::::::::::::::::::::::::::::::::::::::::::::::::::::::::::::::::::::::::::::::::::::::::::::::::::::::::::::

@D0950.29_112895

GACGAACGCTGGCGGCGTGCTTAACACATGCAAGTCGAGCGATGAAGCTTCTTCGGAAGTGGATTAGCGGCGGACGGGTGAGTAACACGTGGGTAACCTGGCCTCATAGAGGGGAATAGCCTTTCGAAAGGAAGATTAATACCGCATAGATTGTAATACCGCATGGTATAGCAATTAAAGGAGTAATCCGCTATGAGATTGGACCCGCGTCGCATTAGCTAGTTGGTGAGGTAACGGCTCACCAAGGCGACGATGCGTAGCCGACCTGAGAGGGTGATCGGCCACATTGGGACTGAGACACGGCCCAGACTCCTACGGGAGGCAGCAGTGGGGAATATTGCACAATGGGGGAAACCCTGATGCAGCAACGCCGCGTGAGTGATGACGGCCTTCGGGTTGTAAAACTCTGTCTTTGGGGACGATAATGACGGTA

+

:::::::::::::::::::::::::::::::::::::::::::::::::::::::::::::::::::::::::::::::::::::::::::::::::::::::::::::::::::::::::::::::::::::::::::::::::::::::::::::::::::::::::::::::::::::::::::::::::::::::::::::::::::::::::::::::::::::::::::::::::::::::::::::::::::::::::::::::::::::::::::::::::::::::::::::::::::::::::::::::::::::::::::::::::::::::::::::::::::::::::::::::::::::::::::::::::::::::::::::::::::::::::::::::::::::::::::::::::

@D0950.29_113009

GATGAACGCTAGCGACAGGCCTAACACATGCAAGTCGAGGGGTAGCACAAGGTAGAATACTGAGGTGACGACCGGCGCACGGGTGAGTAACGCGTATGCAACCTACCTGTAAGAGTGGGATAGCCTCTCGAAAGAGAGATTAATACCGCATAATACCATTTCACTGCATGGTGAGATGGTTAAAGATTTATTGCTTACAGATGGGCATGCGTAACATTAGCTATTGGTGAGGTAACGGCTCACCAAGGCAACGATGTTTAGGGGTTCTGAGAGGAAGGTCCCCCACACTGGTACTGAGACACGGACCAGACTCCTACGGGAGGCAGCAGTGAGGAATATTGGTCAATGGACGAGAGTCTGAACCAGCCAAGTCGCGTGAAGGATGAAGGTCTTATGGATTGTAAACTCTTTTATACGGGAATAAAAATGCCACGTGTGGCATATTGCATGTACCGTATGAATAAGGATCGGCTAACTCCGTGCCAGCAGCCGCGGTAACTGAA

+

:::::::::::::::::::::::::::::::::::::::::::::::::::::::::::::::::::::::::::::::::::::::::::::::::::::::::::::::::::::::::::::::::::::::::::::::::::::::::::::::::::::::::::::::::::::::::::::::::::::::::::::::::::::::::::::::::::::::::::::::::::::::::::::::::::::::::::::::::::::::::::::::::::::::::::::::::::::::::::::::::::::::::::::::::::::::::::::::::::::::::::::::::::::::::::::::::::::::::::::::::::::::::::::::::::::::::::::::::::::::::::::::::::::::::::::::::::::::::::::::::::::::::::::::::::::::

@D0950.29_113042

GACGAACGCTGGCGGCGTGCCTAACACATGCAAGTCGAGCGATGAAGTTCCTTCGGGAGTGGATTAGCGGCGGACGGGTGAGTAACACGTGGGTAACCTGCCTCATAGAGGGGAATAGCCTTTCGAAAGGAAGATTAATACCGCATAAGATTGTAGTACCGCATGGTACAGCAATTAAAGGAGTAATCCGCTATGAGATGGACCCGCGTCGCATTAGCTAGTTGGTGAGGTAACGGCTCACCAAGGCGACGATGCGTAGCCGACCTGAGAGGGTGATCGGCCACATTGGGACTGAGGACACGGCCCAGACTCCTACGGGAGGCAGCAGTGGGGAATATTGCACAATGGGGGAAACCCTGATGCAGCAACGCCGCGTGAGTGATGACGGTCTTCGGATTGTAAAGCTCTGTCTTTAGGGACGATAATGACGGTACCTAAGGAGGAAGCCACGGCTAACTACGTGCCAGCAGCCGCGGTAA

+

:::::::::::::::::::::::::::::::::::::::::::::::::::::::::::::::::::::::::::::::::::::::::::::::::::::::::::::::::::::::::::::::::::::::::::::::::::::::::::::::::::::::::::::::::::::::::::::::::::::::::::::::::::::::::::::::::::::::::::::::::::::::::::::::::::::::::::::::::::::::::::::::::::::::::::::::::::::::::::::::::::::::::::::::::::::::::::::::::::::::::::::::::::::::::::::::::::::::::::::::::::::::::::::::::::::::::::::::::::::::::::::::::::::::::::::::::::::::::::::::

@D0950.29_1131

GATGAACGCTAGCGACAGGCTTAACACATGCAAGTCGAGGGGCAGCACAAGGCAGCAATGCTGAGGTGGCGACCGGCGCACGGGTGAGTAACGCGTATGCAACCTACCTGTTAGCGGGGGATAGCCCGGCGAAAGTCGGATTAATACCGCATGATACTATGTATCCGCATGGATATATATTTAAAGGATTAATTGCTAACAGATGGGCATGCGTTCCATTAGGTAGTTGGTAGAGGTAACGGCCTACCAAGCCATCGATGGATAGGGGTTCTGAGAGGAAGGTCCCCCACACTGGTACTGAGACACGGACCAGACTCCTACGGGAGGCAGCAGTGAGGAATATTGGTCAATGGACGAGAGTCTGAACCAGCCAAGTCGCGTGAAGGATGAAGGTTCTATGGATTGTAAACTTCTTTTATAGGGGAATAAAGTGCAGGACGTTGTCCTGTTTTGTATGTACCCTACGAATAAGCATCGGCTAACTCCGTGCCAGCAGCCGCGGTAA

+

:::::::::::::::::::::::::::::::::::::::::::::::::::::::::::::::::::::::::::::::::::::::::::::::::::::::::::::::::::::::::::::::::::::::::::::::::::::::::::::::::::::::::::::::::::::::::::::::::::::::::::::::::::::::::::::::::::::::::::::::::::::::::::::::::::::::::::::::::::::::::::::::::::::::::::::::::::::::::::::::::::::::::::::::::::::::::::::::::::::::::::::::::::::::::::::::::::::::::::::::::::::::::::::::::::::::::::::::::::::::::::::::::::::::::::::::::::::::::::::::::::::::::::::::::::::::::

@D0950.29_113137

GATGAACGCTAGCGACAGGCTTAACACATGCAAGTCGAGGGGCAGCACAAGGTAGCAATACTGAGGTGGCGACCGGCGCACGGGTGAGTAACGCGTGGGTAACCTGCCTCATACAGGGGATAACAGTTAGAAATGACTGCTAACACCGCATAACCCGCTAGCATCGCATGATGCAGACGGAAAATATTTATAGGTATGAGATGGGCCCGCGTCTGATTAGCTAGTTGGTGGGGTAACGCCTACCAAGGCAACGATCAGTAGCCGACTTGAGAGAGTGATCGGCCACATTGGGACTGAGACACGGCCCAAACTCCTACGGGAGGCAGCAGTGGGGAATATTGGACAATGGGGGAAACCCTGATCCAGCGACGCCGCGTGAGTGAAGAAGTATTTCGGTATGTAAAGCTCTATCAGCAGGGAAGATAATGACAGTACCTGACTAAGAAGCCCCCGGCTAACTACGTGCCAGCAGCCG

+

:::::::::::::::::::::::::::::::::::::::::::::::::::::::::::::::::::::::::::::::::::::::::::::::::::::::::::::::::::::::::::::::::::::::::::::::::::::::::::::::::::::::::::::::::::::::::::::::::::::::::::::::::::::::::::::::::::::::::::::::::::::::::::::::::::::::::::::::::::::::::::::::::::::::::::::::::::::::::::::::::::::::::::::::::::::::::::::::::::::::::::::::::::::::::::::::::::::::::::::::::::::::::::::::::::::::::::::::::::::::::::::::::::::::::::::::::::::::::::

@D0950.29_113138

GATGAACGCTAGCGACAGGCCTAACACATGCAAGTCGAGGGGTAGCACAAGGAAGCTTGCTTCTGAGGTGACGACCGGCGCACGGGTGAGTAACGCGTATGCAACCTACCTATAAGAAGGGGATAGCCTCTCGAAAGAGAGATTAATACCGTATAACACTATGGAATCGCATGGTTTTATAGTTAAAGATTTATTGCTTATAGATGGGCATGCGTAACATTAGCTAGTTGGTAAGGTAACGGCTTACCAAGGCAACGATGTTTAGGGGTTCTGAGAGGAAGGTCCCCCACACTGGAACTGAGACACGGTCCAAACTCCTACGGGAGGCAGCAGTGAGGAATATTGGTCAATGGATGTAAGTCTGAACCAGCCAAGTAGCGTGAAGGATGAAGGCTCTATGGGTCGTAAACTTCTTTTATAAAAGGAATAAAGTATGCCACGTGTGGTGTTTTGTATGTACTTTATGAATAAGGATCGGCTAACTCCGTGCCAGCAGCCGC

+

::::::::::::::::::::::::::::::::::::::::::::::::::::::::::::::::::::::::::::::::::::::::::::::::::::::::::::::::::::::::::::::::::::::::::::::::::::::::::::::::::::::::::::::::::::::::::::::::::::::::::::::::::::::::::::::::::::::::::::::::::::::::::::::::::::::::::::::::::::::::::::::::::::::::::::::::::::::::::::::::::::::::::::::::::::::::::::::::::::::::::::::::::::::::::::::::::::::::::::::::::::::::::::::::::::::::::::::::::::::::::::::::::::::::::::::::::::::::::::::::::::::::::::::::::::

@D0950.29_113148

GATGAACGCTGGCGGCGTGCTTAACACATGCAAGTCGAACGAAGCACTTAACTTAGAATCTTCGGATGAAGAGTTTTGTGACTTAGTGGCGGACAGGTGAGTAACGCGTGGGTAACCTGCCTTATACTGGGGGATAACAGTTAGAAATGACTGCTAATACCGCATAAGCGCACAGTATCACATGATACAGTGCGAAAACTCCGGTGGTATAAGATGGACCCGCGTCTGATTAGCTAGTTGGTAAGGTAACGGCTTACCAAGGCGACGATCAGTAGCCGACTTGAGAGAGTGATCGGCCACATTGGGACTGAGACACGGCCCAAACTCCTACGGGAGGCAGCAGTGGGGAATATTGCACAATGGGCGAAAGCCTGATGCAGCGACGCCGCGTGAAGGATGAAGTATTTCGGTACGTAAAACTTCTATCAGCAAGGAAGATAATGACGGTACTTGACTAAGAAGCCCCGGCTAACTACGTGCCAGCAGCCGCGGTAA

+

:::::::::::::::::::::::::::::::::::::::::::::::::::::::::::::::::::::::::::::::::::::::::::::::::::::::::::::::::::::::::::::::::::::::::::::::::::::::::::::::::::::::::::::::::::::::::::::::::::::::::::::::::::::::::::::::::::::::::::::::::::::::::::::::::::::::::::::::::::::::::::::::::::::::::::::::::::::::::::::::::::::::::::::::::::::::::::::::::::::::::::::::::::::::::::::::::::::::::::::::::::::::::::::::::::::::::::::::::::::::::::::::::::::::::::::::::::::::::::::::::::::::::::::::

@D0950.29_113153

ATTGAACGCTGGCGGCATGCCTTACACATGCAAGTCGAACGGTAACAGGTCTTCGGATGCTGACGAGTGGCGAACGGGTGAGTAATACATCGGAACGTGCCCGATCGTGGGGGATAACGAAGCGAAAGCTTTGCTAATACCGCATACGATCTACGGATGAAAGCAGGGGACCGCAAGGCCTTGCGCGAACGGAGCGGCCGATGGCAGATTAGGTAGTTGGTGGGATAAAAGCTTACCAAGCCGACGATCTGTAGCTGGTCTGAGAGGACGACCAGCCACACTGGGACTGAGACACGGCCCAGACTCCTACGGGAGGCAGCAGTGGGGAATTTTGGACAATGGGCGAAAGCCTGATCCAGCCATGCCGCGTGCAGGATGAAGGCCTTCGGGTTGTAAACTGCTTTTGTACGGAACGAAAAGACTCTGGTTAATACCTGGGGGTCCATGACGGTACCGTAAGAATAAGCACCGGCTAACTACGTGCCAGCAGCCGCGGTAA

+

:::::::::::::::::::::::::::::::::::::::::::::::::::::::::::::::::::::::::::::::::::::::::::::::::::::::::::::::::::::::::::::::::::::::::::::::::::::::::::::::::::::::::::::::::::::::::::::::::::::::::::::::::::::::::::::::::::::::::::::::::::::::::::::::::::::::::::::::::::::::::::::::::::::::::::::::::::::::::::::::::::::::::::::::::::::::::::::::::::::::::::::::::::::::::::::::::::::::::::::::::::::::::::::::::::::::::::::::::::::::::::::::::::::::::::::::::::::::::::::::::::::::::::::::::::

@D0950.29_11317

GATGAACGCTAGCTACAGGCTTAACACATGCAAGTCGAGGGGTAGCATGAAACTTAGCAATAAGTTTTGATGACGACCGGCGCACGGGTGAGTAACACGTATCCAACCTGCCTTTTACTCATGGATAGCCTTCTGAAAAGAAGATTAATACATGATGGTATTCAGAGTTTTCATGGACACTGAATTAAAGATTTATCGGTAAGAGATGGGGATGCGTTCCATTAGATAGTAGGCGGGGTAACGGCCCACCTAGTCAACGATGGATAGGGGTTCTGAGAGGAAGGTCCCCCAGCATTGGACTGAGACACGGCCCAGACTCCTACGGGAGCAGCAGTGGGGAATATTGCACAATGGGGAAACCCTGATGCAGCAACGCCGCGTGAGTGATGAAGGCCTTCGGGTTGTAAAGCTCTGTCTTCAGGGACGATGAATGACGGTACCTGAGGAGGAAGCCACGGCTAACTACGTGCCAGCAGCCGCGGTAA

+

:::::::::::::::::::::::::::::::::::::::::::::::::::::::::::::::::::::::::::::::::::::::::::::::::::::::::::::::::::::::::::::::::::::::::::::::::::::::::::::::::::::::::::::::::::::::::::::::::::::::::::::::::::::::::::::::::::::::::::::::::::::::::::::::::::::::::::::::::::::::::::::::::::::::::::::::::::::::::::::::::::::::::::::::::::::::::::::::::::::::::::::::::::::::::::::::::::::::::::::::::::::::::::::::::::::::::::::::::::::::::::::::::::::::::::::::::::::::::::::::::::::

@D0950.29_113490

GATGAACGCTAGCGACAGGCCTAACACATGCAAGTCGAGGGGTAGCACAAGGTAGTAATACTGAGGTGACGACCGGCGCACGGGTGAGTAACGCGTATGCAACCTACCTGTAAGAGTGGGATAGCCTCTCGAAAGAGAGATTAATACCGCATAATACCATTTCACTGCATGGTGAGATGGTTAAAGATTTATTGCTTACAGATGGGCATGCGTAACATTAGCTAGTTGGTGAGGTAACGGCTCACCAAGGCAACGATGTTTAGGGGTTCTGAGAGGAAGGTCCCCCGACACTGGTACTGAGACACGGACCAGACTCCTACGGGAGGCAGCAGTGAGGAATATTGGTCAATGGACGAGAGTCTGAACCAGCCAAGTCGCGTGAAGGATGAAGGTCTTATGGATTGTAAACTTCTTTTATACGGGAATAAAAATGCCACGTGTGGCATATTGCATGTACCGTATGAATAAGGATCGGCTAACTCCGTGCCAGCAGCCGCGGTAA

+

::::::::::::::::::::::::::::::::::::::::::::::::::::::::::::::::::::::::::::::::::::::::::::::::::::::::::::::::::::::::::::::::::::::::::::::::::::::::::::::::::::::::::::::::::::::::::::::::::::::::::::::::::::::::::::::::::::::::::::::::::::::::::::::::::::::::::::::::::::::::::::::::::::::::::::::::::::::::::::::::::::::::::::::::::::::::::::::::::::::::::::::::::::::::::::::::::::::::::::::::::::::::::::::::::::::::::::::::::::::::::::::::::::::::::::::::::::::::::::::::::::::::::::::::::::::

@D0950.29_113623

GATGAACGCTGGCGGCGTGCTTAACACATGCAAGTCGAACGAAGCACTTAAGGAGCTTGCTCCAAAAGTGACTGAGTGGCGGACGGGTGAGTAACGCGTGGGTAACCTGCCTTACACTGGGGGATAACAGTTGGAAACGACTGCTAATACCGCATAAGCGCACAGTATTGCATGATACAGTGTGAAAAACTCCGGTGGTGTAAGATGGACCCGCGTCTGATTAGCTAGTTGGTGAGGTAATGGCTCACCAAGGCAACGATCAGTAGCCGGCTTGAGAGAGTGAACGGCCACATTGGGACTGAGACACGGCCCAAACTCCTACGGGAGGCAGCAGTGGGAATATTGCACAATGGGGGAAACCCTGATGCAGCAACGCCGCGTGAGTGAAGAAGTATTTGCGGTATGTAAAGCTCTATCAGCAGGGAAGATAATGACGGTACCTGACTAAGAAGCCCCCGGCTAACTACGTGCCAGCAGCCG

+

::::::::::::::::::::::::::::::::::::::::::::::::::::::::::::::::::::::::::::::::::::::::::::::::::::::::::::::::::::::::::::::::::::::::::::::::::::::::::::::::::::::::::::::::::::::::::::::::::::::::::::::::::::::::::::::::::::::::::::::::::::::::::::::::::::::::::::::::::::::::::::::::::::::::::::::::::::::::::::::::::::::::::::::::::::::::::::::::::::::::::::::::::::::::::::::::::::::::::::::::::::::::::::::::::::::::::::::::::::::::::::::::::::::::::::::::::::::::::::::::

@D0950.29_113670

GATGAACGCTGGCGGCGTGCTTAACACATGCAAGTCGAACGAAGCAGCTTTCTTGCTTGCAAGAAAGCTGACTTAGTGGCGGACGGGTGAGTAACGCGTGGGTAACCTGGCCTCATACAGGGGGATAACAGTTGGAAACGACTGCTAAGACCGCATAACCCGCTAGTGTCGCATGACACGGACGGAAAATATTTATAGGTATGAGATGGGCCCGCGTCTGATTAGCTAGTTGGTAAGGTAACGGCTTACCAAGGCGACGATCAGTAGCCGACTTGAGAGAGTGATCGGCCACATTGGGACTGAGACACGGCCCAAACTCCTACGGGAGGCAGCAGTGGGGAATATTGCACAATGGGGGAAACCCTGATGCAGCGACGCCGCGTGAAGGAAGAAGTATTTCGGTATGTAAACTTCTATCAGCAGGGAAGAAAATGACGGTACCTGACTAAGAAGCCCCGGCTAACTACGTGCCAGCAGCCGCGGTAA

+

::::::::::::::::::::::::::::::::::::::::::::::::::::::::::::::::::::::::::::::::::::::::::::::::::::::::::::::::::::::::::::::::::::::::::::::::::::::::::::::::::::::::::::::::::::::::::::::::::::::::::::::::::::::::::::::::::::::::::::::::::::::::::::::::::::::::::::::::::::::::::::::::::::::::::::::::::::::::::::::::::::::::::::::::::::::::::::::::::::::::::::::::::::::::::::::::::::::::::::::::::::::::::::::::::::::::::::::::::::::::::::::::::::::::::::::::::::::::::::::::::::::

@D0950.29_113671

GATGAACGCTGGCGGCGTGCTTAACACATGCAAGTCGAACGAAGCACTTAAGGAGCTTGCTCCAAAAGTGACTGAGTGGCGGACGGGTGAGTAACGCGTGGGTAACCTGCCTTACACTGGGGGATAACAGTTGGAAACGACTGCTAATACCGCATAAGCGCACAGTATTGCATGATACAGTGTGAAAAACTCCGGTGGTGTAAGATGGACCCGCGTCTGATTAGCTAGTTGGTGAGGTAATGGCTCACCAAGGCAACGATCAGTAGCCGGCTTGAGAGAGTGAACGGCCACATTGGGACTGAGACACGGCCCAAACTCCTACGGGAGGCAGCAGTGGGAATATTGCACAATGGGGGAAACCCTGATGCAGCAACGCCGCGTGAGTGAAGAAGTATTTCGGTATGTAAAGCTCTATCAGCAGGGAAGATAATGACGGTACCTGACTAAGAAGCCCCCGGCTAACTACGTGCCAGCAGCCGC

+

::::::::::::::::::::::::::::::::::::::::::::::::::::::::::::::::::::::::::::::::::::::::::::::::::::::::::::::::::::::::::::::::::::::::::::::::::::::::::::::::::::::::::::::::::::::::::::::::::::::::::::::::::::::::::::::::::::::::::::::::::::::::::::::::::::::::::::::::::::::::::::::::::::::::::::::::::::::::::::::::::::::::::::::::::::::::::::::::::::::::::::::::::::::::::::::::::::::::::::::::::::::::::::::::::::::::::::::::::::::::::::::::::::::::::::::::::::::::::::::::

@D0950.29_113679

GATGAACGCTAGCTACAGGCTTAACACATGCAAGTCGAGGGGTAGCATGAAACTTAGCAATAAGTTTTGATGACGACCGGCGCACGGGTGAGTAACACGTATCCAACCTGCCTTTTACTCATGGATAGCCTTCTGAAAAGAAGATTAATACATGATGGTATTCAGAGTTTTCATGGACACTGAATTAAAGATTTTATCGGTAAGAGATGGGGATGCGTTCCATTAGATAGTAGGCGGGGTAACGGCCCACCTAGTCAACGATGGATAGGGGTTCTGAGAGGAAGGTCCCCCACATTGGAACTGAGACACGGTCCAAACGTCCTACGGGAGGCAGCAGTGAGGAATATTGGTCAATGGACGTAAGTCTGAACCAGCCAAGTAGCGTGAAGGATGAAGGCTCTATGGGTCGTAAACTTCTTTTTATAAAAGGAATAAAGTATGCCACGTGTGGTGTTTTTGTATGTACTTTATGAATAAGGATCGGCTAACTCCGTGCCAGCAGCCGC

+

::::::::::::::::::::::::::::::::::::::::::::::::::::::::::::::::::::::::::::::::::::::::::::::::::::::::::::::::::::::::::::::::::::::::::::::::::::::::::::::::::::::::::::::::::::::::::::::::::::::::::::::::::::::::::::::::::::::::::::::::::::::::::::::::::::::::::::::::::::::::::::::::::::::::::::::::::::::::::::::::::::::::::::::::::::::::::::::::::::::::::::::::::::::::::::::::::::::::::::::::::::::::::::::::::::::::::::::::::::::::::::::::::::::::::::::::::::::::::::::::::::::::::::::::::::::::::

@D0950.29_113686

GATGAACGCTAGCTACAGGCTTAACACATGCAAGTCGAGGGGTAGCATGAAACTTAGCAATAAGTTTTGATGACGACCGGCGCACGGGTGAGTAACACGTATCCAACCTGCCTTTTACTCATGGATAGCCTTCTGAAAAGAAGATTAATACATGATGGTATTCAGAGTTTTCATGGACACTGAATTAAAGATTTATCGGTAAGAGATGGGGATGCGTTCCATTAGATAGTAGGCGGGGTAACGGCCCACCTAGTCAACGATGGATAGGGGTTCTGAGAGGAAGGTCCCCCACATTGGAACTGAGACACGGTGCCAAACTCCTACGGGAGGCCAGCAGTGAGGAATATTGGTCAATGGACGTAAGTCTGAACCAGCCAAGTAGCGTGAAGGATGAAGGCTCTATGGGTCGTAAACTT

+

::::::::::::::::::::::::::::::::::::::::::::::::::::::::::::::::::::::::::::::::::::::::::::::::::::::::::::::::::::::::::::::::::::::::::::::::::::::::::::::::::::::::::::::::::::::::::::::::::::::::::::::::::::::::::::::::::::::::::::::::::::::::::::::::::::::::::::::::::::::::::::::::::::::::::::::::::::::::::::::::::::::::::::::::::::::::::::::::::::::::::::::::::::::::::::::::::::::::::::::::::::::::::::::::

@D0950.29_113694

GATGAACGCTAGCGACAGGCCTAACACATGCAAGTCGAGGGGTAGCACAAGGTAGCAATACTGAGGTGACGACCGGCGCACGGGTGAGTAACGCGTATGCAACCTACCTGTAAGAGTGGGATAGCCTCTCGAAAGAGAGATTAATACCGCATAATACCATTTCACTGCATGGTGAGATGGTTAAAGATTTATTGCTTACAGATGGGCATGCGTAACATTAGCTAGTTGGTGAGGTAACGGCTCACCAAGGCAACGATGTTTAGGGGTTCTGAGAGGAAGGTCCCCCACACTGGTACTGAGACACGGACCAGACTCCTACGGGAGGCAGCAGTGAGGAATATTGGTCAATGGACGAGAGTCTGAACCAGCCAAGTCGCGTGAAGGATGAAGGTCTTATGGATTGTAAACTTCTTTTATACGGGAATAAAAATGCCACGTGTGGCATATTGCATGTACCGTATGAATAAGGATCGGCTAACTCCGTGCCAGCAGCCGCGGTAA

+

:::::::::::::::::::::::::::::::::::::::::::::::::::::::::::::::::::::::::::::::::::::::::::::::::::::::::::::::::::::::::::::::::::::::::::::::::::::::::::::::::::::::::::::::::::::::::::::::::::::::::::::::::::::::::::::::::::::::::::::::::::::::::::::::::::::::::::::::::::::::::::::::::::::::::::::::::::::::::::::::::::::::::::::::::::::::::::::::::::::::::::::::::::::::::::::::::::::::::::::::::::::::::::::::::::::::::::::::::::::::::::::::::::::::::::::::::::::::::::::::::::::::::::::::::::::

@D0950.29_11371

GATGAACGCTAGCGACAGGCCTAACACATGCAAGTCGAGGGGTAGCACAAGGAAGCTTGCTTCTGAGGTGACGACCGGCGCACGGGTGAGTAACGCGTATGCAACCTGCCTATAAGAAGGGGATAGCCTCTCGAAAGAGAGATTAATACCGTATAACACTATGAAGCCGCATGGTTTTACAGTTAAAGATTTATTGCTTATAGATGGGCATGCGTAACATTAGCTAGTTGGTGAGGTAACGGCTCACCAAGGCAACGATGTTTAGGGGTTCTGAGAGGAAGGTCCCCCACACTGGTACTGAGACACGGACCAGACTCCTACGGGAGGCAGCAGTGAGGAATATTGGTCAATGGACGAGAGTCTGAACCAGCCAAGTCGCGTGAAGGATGAAGGTCTTATGGATTGTAAACTTCTTTTATACGGGGAAATAAAAAGAGCCACGTGTGGCTTATTGCATGTACCGTATGAATAAGGATCGGCTAACTCCGTGCCAGCAGCC

+

:::::::::::::::::::::::::::::::::::::::::::::::::::::::::::::::::::::::::::::::::::::::::::::::::::::::::::::::::::::::::::::::::::::::::::::::::::::::::::::::::::::::::::::::::::::::::::::::::::::::::::::::::::::::::::::::::::::::::::::::::::::::::::::::::::::::::::::::::::::::::::::::::::::::::::::::::::::::::::::::::::::::::::::::::::::::::::::::::::::::::::::::::::::::::::::::::::::::::::::::::::::::::::::::::::::::::::::::::::::::::::::::::::::::::::::::::::::::::::::::::::::::::::::::::::

@D0950.29_11376

GATGAACGCTAGCGACAGGCCTAACACATGCAAGTCGAGGGGTAGCACAAGGTAGCAATACTGAGGTGACGACCGGCGCACGGGTGAGTAACGCGTATGCAACCTACCTGTAAGAGTGGGATAGCCTCTCGAAAGAGAGATTAATACCGCATAATACCATTTCACTGCATGGTGAGATGGTTAAAGATTTATTGCTTACAGATGGGCATGCGTAACATTAGCTAGTTGGTGAGGTAACGGCTCACCAAGGCAACGATGTTTAGGGGTTCTGAGAGGAAGGTCCCCCACATTGGAACTGAGACACGGTCCAAACTCCTACGGGAGGCAGCAGTGAGGAATATTGGTCAAATGGACGTAAAGTCTGAACCAGCCAAGTAGCGTGAAGGATGAAGGCTCTATGGGTCGTAAACTTCTTTTATAAAGGAATAAAAGTATGCCACGTGTGGTGTTTTGTATGTACTTTATGAATAAGGATCGGCTAAACTCCGTGCCAGCAGCCGCGGTAA

+

::::::::::::::::::::::::::::::::::::::::::::::::::::::::::::::::::::::::::::::::::::::::::::::::::::::::::::::::::::::::::::::::::::::::::::::::::::::::::::::::::::::::::::::::::::::::::::::::::::::::::::::::::::::::::::::::::::::::::::::::::::::::::::::::::::::::::::::::::::::::::::::::::::::::::::::::::::::::::::::::::::::::::::::::::::::::::::::::::::::::::::::::::::::::::::::::::::::::::::::::::::::::::::::::::::::::::::::::::::::::::::::::::::::::::::::::::::::::::::::::::::::::::::::::::::::::::

@D0950.29_113774

GATGAACGCTAGCTACAGGCTTAACACATGCAAGTCGAGGGGTAGCATGAAACTTAGCAATAAGTTTTGATGACGACCGGCGCACGGGTGAGTAACACGTATCCAACCTGCCTTTTACTCATGGATAGCCTTCTGAAAAGAAGATTAATACATGATGGTATTCAGAGTTTTCATGGACACTGAATTAAAGATTTATCGGTAAGAGATGGGGATGCGTTCCATTAGATAGTAGGCGGGGTAACGGCCCACCTAGTCAACGATGGATAGGGGTTCTGAGAGGAAGGTCCCCCACATTGGAACTGAGACACGGTCCAAACGTCCTACGGGAGGCAGCAGTGAGGAATATTGGTCAATGGACGTAAGTCTGAACCAGCCAAGTAGCGTGAAGGATGAAGGCTCTATGGGTCGTAAACTTCTTTTATAAAAGGAATAAAGTATGCCACGTGTGGTGTTTTGTATGTACTTTATGAATAAGGATCGGCTAACTCCGTGCCAGCAGCCGC

+

:::::::::::::::::::::::::::::::::::::::::::::::::::::::::::::::::::::::::::::::::::::::::::::::::::::::::::::::::::::::::::::::::::::::::::::::::::::::::::::::::::::::::::::::::::::::::::::::::::::::::::::::::::::::::::::::::::::::::::::::::::::::::::::::::::::::::::::::::::::::::::::::::::::::::::::::::::::::::::::::::::::::::::::::::::::::::::::::::::::::::::::::::::::::::::::::::::::::::::::::::::::::::::::::::::::::::::::::::::::::::::::::::::::::::::::::::::::::::::::::::::::::::::::::::::::::

@D0950.29_113783

ATTGAACGCTGGCGGCATGCCTTACACATGCAAGTCGAACGGTAACAGGTCTTCGGATGCTGACGAGTGGCGAACGGGTGAGTAATACATCGGAACGTGCCCGATCGTGGGGGATAACGAAGCGAAAGCTTTGCTAATACCGCATACGATCTACGGATGAAAGCAGGGGACCGCAAGGCCTTGCGCGAACGGAGCGGCCGATGGCAGATTAGGTAGTTGGTGGGATAAAAGCTTACCAAGCCGACGATCTGTAGCTGGTCTGAGAGGACGACCAGCCACACTGGGACTGAGACACGGCCCAGACTCCTACGGGAGGCAGCAGTGGGGAATTTTGGACAATGGGCGAAAGCCTGATCCAGCCATGCCGCGTGCAGGATGAAGGCCTTCGGGTTGTAAACTGCTTTTGTACGGAACGAAAAGACTCTGGTTAATACCTGGGGGTCCATGACGGTACCGTAAGAATAAGCACCGGCTAACTACGTGCCAGCAGCCGCGGTAA

+

:::::::::::::::::::::::::::::::::::::::::::::::::::::::::::::::::::::::::::::::::::::::::::::::::::::::::::::::::::::::::::::::::::::::::::::::::::::::::::::::::::::::::::::::::::::::::::::::::::::::::::::::::::::::::::::::::::::::::::::::::::::::::::::::::::::::::::::::::::::::::::::::::::::::::::::::::::::::::::::::::::::::::::::::::::::::::::::::::::::::::::::::::::::::::::::::::::::::::::::::::::::::::::::::::::::::::::::::::::::::::::::::::::::::::::::::::::::::::::::::::::::::::::::::::::

@D0950.29_113807

ATTGAACGCTGGCGGCAGGCCTAACACATGCAAGTCGAACGGTAGCACAGAGAGCTTGCTCTCGGGTGACGAGTGGCGGACGGGTGAGTAATGTCTGGGAAACTGCCCGATGGAGGGGGATAACTACTGGAAACGGTAGCTAATACCGCATAACGTCGCAAGACCAAAGAGGGGGACCTTCGGGCCTCTTGCCATCGGATGTGCCCAGATGGGATTAGCTAGTAGGTGGGGTAATGGCTCACCTAGGCGACGATCCCTAGCTGGTCTGAGAGGATGACCAGCCACACTGGAACTGAGACACGGTCCAGACTCCTACGGGAGGCAGCAGTGGGGAATATTGCACAATGGGCGCAAGCCTGATGCAGCCATGCCGCGTGTATGAAGAAGGCCTTCGGGTTGTAAAGTACTTTCAGCGAGGAGGAAGGCGTTGTGGTTAATAACCGCAGCGATTGACGTTACTCGCAGAAGAAGCACCGGCTAACTCCGTGCCAGCAGCCGCGGTAA

+

::::::::::::::::::::::::::::::::::::::::::::::::::::::::::::::::::::::::::::::::::::::::::::::::::::::::::::::::::::::::::::::::::::::::::::::::::::::::::::::::::::::::::::::::::::::::::::::::::::::::::::::::::::::::::::::::::::::::::::::::::::::::::::::::::::::::::::::::::::::::::::::::::::::::::::::::::::::::::::::::::::::::::::::::::::::::::::::::::::::::::::::::::::::::::::::::::::::::::::::::::::::::::::::::::::::::::::::::::::::::::::::::::::::::::::::::::::::::::::::::::::::::::::::::::::::::

@D0950.29_113837

GATGAACGCTGGCGGCGTGCTTAACACATGCAAGTCGAACGAAGCAGCTTTCTTGCTTGCAAGAAAGCTGACTTAGTGGCGGACGGGTGAGTAACGCGTGGGTAACCTGGCCTCATACAGGGGGATAACAGTTGGAAACGACTGCTAAGACCAGCATAACCCGCTAGTGTCGCATGACACGGACGGAAAATATTTATAGGTATGAGATGGGCCCGCGTCTGATTAGCTAGTTGGTAAGGTAACGGCTTACCAAGGCGACGATCAGTAGCCGACTTGAGAGAGTGATCGGCCACATTGGGACTGAGACACGGCCCAAACTCCTACGGGAGGCAGCAGTGGGGAATATTGCACAATGGGGGAAACCCTGATGCAGCAACGCCGCGTGAGTGATGACGGCCTTCGGGTTGT

+

::::::::::::::::::::::::::::::::::::::::::::::::::::::::::::::::::::::::::::::::::::::::::::::::::::::::::::::::::::::::::::::::::::::::::::::::::::::::::::::::::::::::::::::::::::::::::::::::::::::::::::::::::::::::::::::::::::::::::::::::::::::::::::::::::::::::::::::::::::::::::::::::::::::::::::::::::::::::::::::::::::::::::::::::::::::::::::::::::::::::::::::::::::::::::::::::::::::::::::::::::::::::

@D0950.29_113853

GATGAACGCTGGCGGCGTGCTTAACACATGCAAGTCGAACGAAGCGCTGGAGGAGCTTGCTCCAAAGGTGACTGAGTGGCGGACGGGTGAGTAACGCGTGGGTAACCTGCCTTACACTGGGGGATAACAGTTGGAAACGACTGCTAATACCGCATAAGCGCACAGTATTGCATGATACAGTGTGAAAACTCCGGTGGTGTAAGATGGACCCGCGTCTGATTAGCTAGTTGGTGAGGTAATGGCTCACCAAGGCAACGATCAGTAGCCGGCTTGAGAGAGTGAACGGCCACATTGGGACTGAGACACGGCCCGAAACTCCTACGGGAGGCAGCAGTGGGGAATATTGCACAATGGGGGAACCCTGATGCAGCAACGCCGCGTGAGTGAAGAAGTATTTCGGTATGTAAGCTCTATCAGCAGGGAAGATAATGACGGTACCTGACTAAGAGCCCGGCTAACTACGTGCCAGCAGCCGCGGTAA

+

:::::::::::::::::::::::::::::::::::::::::::::::::::::::::::::::::::::::::::::::::::::::::::::::::::::::::::::::::::::::::::::::::::::::::::::::::::::::::::::::::::::::::::::::::::::::::::::::::::::::::::::::::::::::::::::::::::::::::::::::::::::::::::::::::::::::::::::::::::::::::::::::::::::::::::::::::::::::::::::::::::::::::::::::::::::::::::::::::::::::::::::::::::::::::::::::::::::::::::::::::::::::::::::::::::::::::::::::::::::::::::::::::::::::::::::::::::::::::::::::::

@D0950.29_113965

GATGAACGCTGGCGGCGTGCTTAACACATGCAAGTCGAACGAAGCGCTGGAGGAGCTTGCTCCAAAGGTGACTGAGTGGCGGACGGGTGAGTAACGCGTGGGTAACCTGCCTTACACTGGGGGATAACAGTTGGAAACGACTGCTAATACCGCATAAGCGCACAGTATTGCATGATACAGTGTGAAAACTCCGGTGGTGTAAGATGGACCCGCGTCTGATTAGCTAGTTGGTGAGGTAATGGCTCACCAAGGCAACGATCAGTAGCCGGCTTGAGAGAGTGAACGGCCACATTGGGACTGAGACACGGCCCAAACTCCTACGGGAGGCAGCAGTGGGGAATATTGCACAATGGGGGAAACCCTGATGCAGCAACGCCGCGTGAGTGAAGAAGTATTTCGGTATGTAAAGCTCTATCAGCAGGGAAGATAATGACGGTACCTGACTAAGAAGCCCCGGCTAACTACGTGCCAGCAGCCGCGGTAA

+

::::::::::::::::::::::::::::::::::::::::::::::::::::::::::::::::::::::::::::::::::::::::::::::::::::::::::::::::::::::::::::::::::::::::::::::::::::::::::::::::::::::::::::::::::::::::::::::::::::::::::::::::::::::::::::::::::::::::::::::::::::::::::::::::::::::::::::::::::::::::::::::::::::::::::::::::::::::::::::::::::::::::::::::::::::::::::::::::::::::::::::::::::::::::::::::::::::::::::::::::::::::::::::::::::::::::::::::::::::::::::::::::::::::::::::::::::::::::::::::::::::

@D0950.29_114001

GATGAACGCTAGCGACAGGCCTAACACATGCAAGTCGAGGGGTAGCACAAGGTAGTAATACTGAGGTGACGACCGGCGCACGGGTGAGTAACGCGTATGCAACCTACCTGTAAGAGTGGGATAGCCTCTCGAAAGAGAGATTAATACCGCATAATACCATTTCACTGCATGGTGAGATGGTTAAAGATTTATTGCTTACAGATGGGCATGCGTAACATTAGCTAGTTGGTGAGGTAACGGCTCACCAAGGCAACGATGTTTAGGGGTTCTGAGAGGAAGGTCCCCCACACTGGTACTGAGACACGGACCAGACTCCTACGGGAGGCAGCAGTGAGGAATATTGGTCAATGGACGAGAGTCTGAACCAGCCAAGTCGCGTGAAGGATGAAGGTCTTATGGATTGTAAACTTCTTTTATACGGGAATAAAAATGCCACGTGTGGCATATTGCATGTACCGTATGAATAAGGATCGGCTAACTCCGTGCCAGCAGCCGCGGTAA

+

:::::::::::::::::::::::::::::::::::::::::::::::::::::::::::::::::::::::::::::::::::::::::::::::::::::::::::::::::::::::::::::::::::::::::::::::::::::::::::::::::::::::::::::::::::::::::::::::::::::::::::::::::::::::::::::::::::::::::::::::::::::::::::::::::::::::::::::::::::::::::::::::::::::::::::::::::::::::::::::::::::::::::::::::::::::::::::::::::::::::::::::::::::::::::::::::::::::::::::::::::::::::::::::::::::::::::::::::::::::::::::::::::::::::::::::::::::::::::::::::::::::::::::::::::::::

@D0950.29_114007

GATGAACGCTAGCGACAGGCCTAACACATGCAAGTCGAGGGGTAGCACAAGGTAGCAATACTGAGGTGACGACCGGCGCACGGGTGAGTAACGCGTATGCAACCTACCTGTAAGAGTGGGATAGCCTCTCGAAAGAGAGATTAATACCGCATAATACCATTTTACTGCATGGTGAGATGGTTAAAGATTTATTGCTTACAGATGGGCATGCGTAACATTAGCTAGTTGGTGAGGTAACGGCTCACCAAGGCAACGATGTTTAGGGGTTCTGAGAGGAAGGTCCCCCACACTGGTACTGAGACACGGACCAGACTCCTACGGGAGGCAGCAGTGAGGAATATTGGTCAATGGACGAGAGTCTGAACCAGCCAAGTCGCGTGAAGGATGAAGGCTCTATGGGTCGTAAACTTCTTTTATAAAGGAATAAAGTATGCCACGTGTGGTGTTTTGTATGTACTTTATGAATAAGGATCGGCTAACTCCGTGCCAGCAGCCGCGGTAA

+

::::::::::::::::::::::::::::::::::::::::::::::::::::::::::::::::::::::::::::::::::::::::::::::::::::::::::::::::::::::::::::::::::::::::::::::::::::::::::::::::::::::::::::::::::::::::::::::::::::::::::::::::::::::::::::::::::::::::::::::::::::::::::::::::::::::::::::::::::::::::::::::::::::::::::::::::::::::::::::::::::::::::::::::::::::::::::::::::::::::::::::::::::::::::::::::::::::::::::::::::::::::::::::::::::::::::::::::::::::::::::::::::::::::::::::::::::::::::::::::::::::::::::::::::::::::

@D0950.29_114017

GATGAACGCTAGCGACAGGCCTAACACATGCAAGTCGAGGGGTAGCACAGGGAAGCTTGCATTCTGAGGCGTGACGGACCGGCGCGACGGGTGAGTAACGCGTATGCAACCTGCCCATAACAGGGGGATAATCGGAAGAAATTCCGTCTAATACCGCGTAATCCCGGATTTTCACATGAGAAATCGGGTAAGAAGCAATTCGGTTATGGATGGGCATGCGGAACATTAGGTAGTTGGTGAGGTAACGGCTCACCAAGCCGACGATGTATAGGGGTTCTGAGAGGAAGGTCCCCCACACTGGTACTGAGACACGGACCAGACTCCTACGGGAGGCAGCAGTGAGGAATATTGGTCAATGGGCGCGAGCCTGAACCAGCCAAGTCGCGTGAAGGATGAAGGTTCTATGGATTGTAAACTTCTTTTGTCAGGGAACAAAGAGCCCATGTATGGGCAGATGAGTGTACCTGAAGAAAAAGCATCGGCTAACTCCGTGCCAGCAGCCGCGGTAA

+

:::::::::::::::::::::::::::::::::::::::::::::::::::::::::::::::::::::::::::::::::::::::::::::::::::::::::::::::::::::::::::::::::::::::::::::::::::::::::::::::::::::::::::::::::::::::::::::::::::::::::::::::::::::::::::::::::::::::::::::::::::::::::::::::::::::::::::::::::::::::::::::::::::::::::::::::::::::::::::::::::::::::::::::::::::::::::::::::::::::::::::::::::::::::::::::::::::::::::::::::::::::::::::::::::::::::::::::::::::::::::::::::::::::::::::::::::::::::::::::::::::::::::::::::::::::::::::::

@D0950.29_114101

GATGAACGCTAGCGACAGGCCTAACACATGCAAGTCGAGGGGTAGCACAAGGTAGCAATACTGAGGTGACGACCGGCGCACGGGTGAGTAACACGTATCCAACCTGCCTTTTACTCATGGATAGCCTTCTGAAAAAGAAGATTAATACATGATGGTATTCAGAGTTTTCATGGACACTGAATTAAAGATTTTATCGGTAAGAGATGGGGATGCGTTCCATTAGATAGTAGGCGGGGTAACGGCCCACCTAAGTCAACGATGGATAGGGGTTCTGAGAGGAAGGTCCCCCACATTGGAACTGAGACACGGTCCAAACGTCCTACGGGAGGCAGCAGTGAGGAATATTGGTCAATGGTCGAGAGACTGAACCAGCCAAGTCGCGTGAGGGATGAAGGTTCTATGGATTGTAAACCTCTTTTGTCAGGGAGCAACGACATCCACGAGTGGGTGAATGAGAGTACCTGAAGAAAAAGCATCGGCTAACT

+

:::::::::::::::::::::::::::::::::::::::::::::::::::::::::::::::::::::::::::::::::::::::::::::::::::::::::::::::::::::::::::::::::::::::::::::::::::::::::::::::::::::::::::::::::::::::::::::::::::::::::::::::::::::::::::::::::::::::::::::::::::::::::::::::::::::::::::::::::::::::::::::::::::::::::::::::::::::::::::::::::::::::::::::::::::::::::::::::::::::::::::::::::::::::::::::::::::::::::::::::::::::::::::::::::::::::::::::::::::::::::::::::::::::::::::::::::::::::::::::::::::::

@D0950.29_114125

GATGAACGCTAGCTACAGGCTTAACACATGCAAGTCGAGGGGTAGCATGAAACTTAGCAATAAGTTTTGATGACGACCGGCGCACGGGTGAGTAACACGTATCCAACCTGCCTTTTACTCATGGATAGCCTTCTGAAAAGAAGATTAATACATGATGGTATTCAGAGTTTTCATGGACACTGAATTAAAGATTTTATCGGTAAGAGATGGGGATGCGTTCCATTAGATAGTAGGCGGGGTAACGGCCCACCTAGTCAACGATGGATAGGGGTTCTGAGAGGAAGGTCCCCCACATTGGAACTGAGACACGGTCCAAACTCCTACGGGAGGCAGCAGTGAGGAATATTGGTCAATGGACGTAAGTCTGAACCAGCCAAGTAGCGTGAAGGATGAAGGCTCTATGGGTCGTAAACTTCTTTTTATAAAAGGAATAAAGAGCCACCGTGTGGTGTTTTTGTATGTACTTTATGAATAAAGGATCGGCTAAACTCCGTGCCAGCAGCCGC

+

::::::::::::::::::::::::::::::::::::::::::::::::::::::::::::::::::::::::::::::::::::::::::::::::::::::::::::::::::::::::::::::::::::::::::::::::::::::::::::::::::::::::::::::::::::::::::::::::::::::::::::::::::::::::::::::::::::::::::::::::::::::::::::::::::::::::::::::::::::::::::::::::::::::::::::::::::::::::::::::::::::::::::::::::::::::::::::::::::::::::::::::::::::::::::::::::::::::::::::::::::::::::::::::::::::::::::::::::::::::::::::::::::::::::::::::::::::::::::::::::::::::::::::::::::::::::::

@D0950.29_114214

GATGAACGCTAGCTACAGGCTTAACACATGCAAGTCGAGGGGTAGCATGAAACTTAGCAATAAGTTTGATGACGACCGGCGCACGGGTGAGTAACACGTATCCAACCTGCCTTTTACTCATGGATAGCCTTCTGAAAAGAAGATTAATACATGATGGTATTCAGAGTTTTCATGGACACTGAATTAAAGATTTTATCGGTAAGAGATGGGGATGCGTTCCATTAGATAGTAGGCGGGGTAACGGCCCACCTAGTCAACGATGGATAGGGGTTCTGAGAGGAAGGTCCCCCACATTGGAACTGAGACACGGTCCAAACGTCCTACGGGAGGCAGCAGTGAGGAATATTGGTCAATGGACGTAAGTCTGAACCAGCCAAGTCGCGTGAAGGATGAAGGCTCTA

+

:::::::::::::::::::::::::::::::::::::::::::::::::::::::::::::::::::::::::::::::::::::::::::::::::::::::::::::::::::::::::::::::::::::::::::::::::::::::::::::::::::::::::::::::::::::::::::::::::::::::::::::::::::::::::::::::::::::::::::::::::::::::::::::::::::::::::::::::::::::::::::::::::::::::::::::::::::::::::::::::::::::::::::::::::::::::::::::::::::::::::::::::::::::::::::::::::::::::::::::::::

@D0950.29_114276

GATGAACGCTAGCGACAGGCCTAACACATGCAAGTCGAGGGGTAGCACAAGGAAGCTTGCTTCTGAGGTGACGACCGGCGCACGGGTGAGTAACGCGTATGCAACCTACCTGTAAGAGTGGGATAGCCTCTCGAAAGAGAGATTAATACCGCATGATACTATGGAGCCGCATGGTTTTATAGTTAAAGATTTATTGCTTACAGATGGGCATGCGTAACATTAGCTAGTTGGTAAGGTAACGGCTTACCAAGGCAACGATGTTTAGGGGTTCTGAGAGGAAGGTCCCCCACACTGGTACTGAGACACGGACCAGACTCCTACGGGAGGCAGCAGTGAGGAATATTGGTCAATGGACGAGAGTCTGAACCAGCCAAGTCGCGTGAAGGATGAAGGTCTTATGGATTGTAAACTTCTTTTATGCGGGAATAAAAAATGCCACGTGTGGCATATTGCATGTACCGTATGAATAAGGATCGGCTAACTCCGTGCCAGCAGCCGCGGTAA

+

::::::::::::::::::::::::::::::::::::::::::::::::::::::::::::::::::::::::::::::::::::::::::::::::::::::::::::::::::::::::::::::::::::::::::::::::::::::::::::::::::::::::::::::::::::::::::::::::::::::::::::::::::::::::::::::::::::::::::::::::::::::::::::::::::::::::::::::::::::::::::::::::::::::::::::::::::::::::::::::::::::::::::::::::::::::::::::::::::::::::::::::::::::::::::::::::::::::::::::::::::::::::::::::::::::::::::::::::::::::::::::::::::::::::::::::::::::::::::::::::::::::::::::::::::::::::

@D0950.29_114339

GATGAACGCTGGCGGCGTGCTTAACACATGCAAGTCGAACGAAGCACTTAAGGAGCTTGCTCCAAAAGTGACTGAGTGGCGGACGGGTGAGTAACGCGTGGGTAACCTGCCTTACACTGGGGGATAACAGTTGGAAACGACTGCTAATACCGCATAAGCGCACAGTATTGCATGATACAGTGTGAAAAACTCCGGTGGTGTAAGATGGACCCGCGTCTGATTAGCTAGTTGGTGAGGTAATGGCTCACCAAGGCAACGATCAGTAGCCGGCTTGAGAGAGTGAACGGCCACATTGGGACTGAGACACGGCCCAAACTCCTACGGGAGGCAGCAGTGGGAATATTGCACAATGGGGGAAACCCTGATGCAGCAACGCCGCGTGAGTGAAGAAGTATTTGCGGTATGTAAAGCTCTATCAGCAGGGAAGATAATGACGGTACCTGACTAAGAAGCCCCCGGCTAACTACGTGCCAGCAGCCG

+

::::::::::::::::::::::::::::::::::::::::::::::::::::::::::::::::::::::::::::::::::::::::::::::::::::::::::::::::::::::::::::::::::::::::::::::::::::::::::::::::::::::::::::::::::::::::::::::::::::::::::::::::::::::::::::::::::::::::::::::::::::::::::::::::::::::::::::::::::::::::::::::::::::::::::::::::::::::::::::::::::::::::::::::::::::::::::::::::::::::::::::::::::::::::::::::::::::::::::::::::::::::::::::::::::::::::::::::::::::::::::::::::::::::::::::::::::::::::::::::::

@D0950.29_114388

GATGAACGCTGGCGGCGTGCTTAACACATGCAAGTCGAACGAAGCGCTGGAGGAGCTTGCTCCAAAGGTGACTGAGTGGCGGACGGGTGAGTAACGCGTGGGTAACCTGCCTTACACTGGGGGATAACAGTTGGAAACGACTGGCTAATACCGCATAAGCGCACAGTATTGCATGATACAGTGTGAAAAACTCCGGTGGTGTAAGATGGACCCCGCGTCTGATTAGCTAGTTGGTGAGGTAATGGCTCACCAAGGCAACGATCAGTAGCCGGCTTGAGAGAGTGAACGGCCACATTGGGACTGAGACACGGCCCAAACTCCTACCGGGAGGCAGCAGTGGGGAAATATTTGCACAATGGGGGAAACCCTGATGCAGCAACGCCGCGTGAGTGAAGAAGTATTTCGGTATGTAAACGCTCTATCAGCAGGGAAGATAATGACGGTACCTGACTAAGAAGCCCCGGCTAACTACGTGCCAGCAGCCGCGGTAA

+

:::::::::::::::::::::::::::::::::::::::::::::::::::::::::::::::::::::::::::::::::::::::::::::::::::::::::::::::::::::::::::::::::::::::::::::::::::::::::::::::::::::::::::::::::::::::::::::::::::::::::::::::::::::::::::::::::::::::::::::::::::::::::::::::::::::::::::::::::::::::::::::::::::::::::::::::::::::::::::::::::::::::::::::::::::::::::::::::::::::::::::::::::::::::::::::::::::::::::::::::::::::::::::::::::::::::::::::::::::::::::::::::::::::::::::::::::::::::::::::::::::::::::::

@D0950.29_114419

GATGAACGCTAGCTACAGGCTTAACACATGCAAGTCGAGGGGTAGCATGAAACTTAGCAATAAGTTTTGATGACGACCGGCGCACGGGTGAGTAACACGTATCCAACCTGCCTTTTACTCATGGATAGCCTTCTGAAAAGAAGATTAATACATGATGGTATTCAGAGTTTTCATGGACACTGAATTAAAGATTTATCGGTAAGAGATGGGGATGCGTTCCATTAGATAGTAGGCGGGGTAACGGCCCACCTAGTCAACATGGATAGGGGTTCTGAGAGGAAGGTCCCCCACATTGGAACTGAGACACGGTCCAAACGTCCTACGGGAGGCAGCAGTGAGGAATATTGGTCAATGGACGTAAGTCTGAACCAGCCAAGTAGCGTGAAGGATGAAGGCTC

+

::::::::::::::::::::::::::::::::::::::::::::::::::::::::::::::::::::::::::::::::::::::::::::::::::::::::::::::::::::::::::::::::::::::::::::::::::::::::::::::::::::::::::::::::::::::::::::::::::::::::::::::::::::::::::::::::::::::::::::::::::::::::::::::::::::::::::::::::::::::::::::::::::::::::::::::::::::::::::::::::::::::::::::::::::::::::::::::::::::::::::::::::::::::::::::::::::::::::::::::

@D0950.29_114431

GATGAACGCTAGCGACAGGCCTAACACATGCAAGTCGAGGGGTAGCACAAGGTAGCAATACTGAGGTGACGACCGGCGCACGGGTGAGTAACGCGTATGCAACCTACCTGTAAGAGTGGGATAGCCTCTCGAAAGAGAGATTAATACCGCATAATACCATTTCACTGCATGGTGAGATGGTTAAAGGATTTATTGCTTACAGATGGGCATGCGTAACATTAGCTAGTTGGTGAGGTAACGGCTCACCAAGGCAACGATGTTTAGGGGTTCTGAGAGGAAGGTCCCCCGACACTGGTACTGAGACACGGACCAGACTCCTACGGGAGGCAGCAGTGAGGAATATTGGTCAATGGACGAGAGTCTGAACCAGCCAAGTCGCGTGAAGGATGAAGGTCTTATGGATTGTAAACTTCTTTTATACGGGGAATAAAAATGCCACGTGTGGCATATTGCATGTACCGTATGAATAAGGATCGGCTAACTCCGTGCCAGCAGCCGCGGTAA

+

::::::::::::::::::::::::::::::::::::::::::::::::::::::::::::::::::::::::::::::::::::::::::::::::::::::::::::::::::::::::::::::::::::::::::::::::::::::::::::::::::::::::::::::::::::::::::::::::::::::::::::::::::::::::::::::::::::::::::::::::::::::::::::::::::::::::::::::::::::::::::::::::::::::::::::::::::::::::::::::::::::::::::::::::::::::::::::::::::::::::::::::::::::::::::::::::::::::::::::::::::::::::::::::::::::::::::::::::::::::::::::::::::::::::::::::::::::::::::::::::::::::::::::::::::::::::

@D0950.29_11450

GATGAACGCTGGCGGCGTGCTTAACACATGCAAGTCGAACGAAGCAACTATCTTGCTTGCAAGAGAGTTGACTTAGTGGCGGACGGGTGAGTAACGCGTGGGTAACCTGCCTCATACAGGGGATAACAGTTGGAAACGACTGCTAAGACCGCATAACCCGCTAGTGTCGCATGACACGGACGGAAAATATTTTATAGGTATGAGATGGGCCCGCGTCTGATTAGCTAGTTGGTAAGGTAACGGCTTACCAAGGCGACGATCAGTAGCCGACTTGAGAGAGTGATCGGCCACATTGGGACTGAGACACGGCCCAAACTCCTACGGGAGGCAGCAGTGGGGAATATTGGACAATGGGGGAAACCCTGATCCAGCGACGCCGCGTGAGTGAAGAAGTATTTCGGTATGTAAAGCTCTATCAGCAGGGAAGATAATGACAGTACCTGACTAAGAAGCCCCCGGCTAACTACGTGCCAGCAGCCG

+

::::::::::::::::::::::::::::::::::::::::::::::::::::::::::::::::::::::::::::::::::::::::::::::::::::::::::::::::::::::::::::::::::::::::::::::::::::::::::::::::::::::::::::::::::::::::::::::::::::::::::::::::::::::::::::::::::::::::::::::::::::::::::::::::::::::::::::::::::::::::::::::::::::::::::::::::::::::::::::::::::::::::::::::::::::::::::::::::::::::::::::::::::::::::::::::::::::::::::::::::::::::::::::::::::::::::::::::::::::::::::::::::::::::::::::::::::::::::::::::::

@D0950.29_11455

GATGAACGCTAGCGACAGGCCTAACACATGCAAGTCGAGGGGTAGCACAAGGTAGTAATACTGAGGTGACGACCGGCGCACGGGTGAGTAACGCGTATGCAACCTACCTGTAAGAGTGGGATAGCCTCTCGAAAGAGAGATTAATACCGCATAATACCATTTCACTGCATGGTGAGATGGTTAAAGATTTATTGCTTACAGATGGGCATGCGTAACATTAGCTAGTTGGTGAGGTAACGGCTCACCAAGGCAACGATGTTTAGGGGTTCTGAGAGGAAGGTCCCCCGACACTGGTACTGAGACACGGACCAGACTCCTACGGGAGGCAGCAGTGAGGAATATTGGTCAATGGACGAGAGTCTGAACCAGCCAAGTCGCGTGAAGGATGAAGGTCTTATGGATTGTAAACTTCTTTTATACGGGAATAAAAATGCCACGTGTGGCATATTGCATGTACCGTATGAATAAGGATCGGCTAACTCCGTGCCAGCAGCCGCGGTAA

+

::::::::::::::::::::::::::::::::::::::::::::::::::::::::::::::::::::::::::::::::::::::::::::::::::::::::::::::::::::::::::::::::::::::::::::::::::::::::::::::::::::::::::::::::::::::::::::::::::::::::::::::::::::::::::::::::::::::::::::::::::::::::::::::::::::::::::::::::::::::::::::::::::::::::::::::::::::::::::::::::::::::::::::::::::::::::::::::::::::::::::::::::::::::::::::::::::::::::::::::::::::::::::::::::::::::::::::::::::::::::::::::::::::::::::::::::::::::::::::::::::::::::::::::::::::::

@D0950.29_114579

GATGAACGCTAGCGACAGGCCTAACACATGCAAGTCGAGGGGTAGCACAAGGAAGCTTGCTTCTGAGGTGACGACCGGCGCACGGGTGAGTAACGCGTATGCAACCTACCTGTAAGAGTGGGATAGCCTCTCGAAAGAGAGATTAATACCGCATAATACCATTTCACTGCATGGTGAGATGGTTAAAGATTTATTGCTTACAGATGGGCATGCGTAACATTAGCTAGTTGGTGAGGTAACGGCTCACCAAGGCAACGATGTTTAGGGGTTCTGAGAGGAAGGTCCCCCACACTGGTACTGAGACACGGACCAGACTCCTACGGGAGGCAGCAGTGAGGAATATTGGTCAATGGACGAGAGTCTGAACCAGCCAAGTCGCGTGAAGGATGAAGGTCTTATGGATTGTAAACTTCTTTTATAAAGGAATAAAGTATGCCACGTGTGGTGTTTTTGTAATGTACTTTATGAATAAGGATCGGCTAACTCCGTGCCAGCAGCCGCGGTAA

+

::::::::::::::::::::::::::::::::::::::::::::::::::::::::::::::::::::::::::::::::::::::::::::::::::::::::::::::::::::::::::::::::::::::::::::::::::::::::::::::::::::::::::::::::::::::::::::::::::::::::::::::::::::::::::::::::::::::::::::::::::::::::::::::::::::::::::::::::::::::::::::::::::::::::::::::::::::::::::::::::::::::::::::::::::::::::::::::::::::::::::::::::::::::::::::::::::::::::::::::::::::::::::::::::::::::::::::::::::::::::::::::::::::::::::::::::::::::::::::::::::::::::::::::::::::::::::

@D0950.29_114598

ATTGAACGCTGGCGGCAGGCCTAACACATGCAAGTCGAACGGTAGCACAGAGAGCTTGCTCTCGGGTGACGAGTGGCGGACGGGTGAGTAATGTCTGGGAAACTGCCCGATGGAGGGGGATAACTACTGGAAACGGTAGCTAATACCGCATAACGTCGCAAGACCAAAGAGGGGGACCTTCGGGCCTCTTGCCATCGGATGTGCCCAGATGGGATTAGCTAGTAGGTGGGGTAACGGCTCACCTAGGCGACGATCCCTAGCTGGTCTGAGAGGATGACCAGCCACACTGGAACTGAGACACGGTCCAGACTCCTACGGGAGGCAGCAGTGGGGAATATTGCACAATGGGCGCAAGCCTGATGCAGCCATGCCGCGTGTATGAAGAAGGCCTTCGGGTTGTAAAGTACTTTCAGCGAGGAGGAAGGCATTAAGGTTAATAACTTTAGTGATTGACGTTACTCGCAGAAGAAGCACCGGCTAACTCCGTGCCAGCAGCCGCGGTAA

+

::::::::::::::::::::::::::::::::::::::::::::::::::::::::::::::::::::::::::::::::::::::::::::::::::::::::::::::::::::::::::::::::::::::::::::::::::::::::::::::::::::::::::::::::::::::::::::::::::::::::::::::::::::::::::::::::::::::::::::::::::::::::::::::::::::::::::::::::::::::::::::::::::::::::::::::::::::::::::::::::::::::::::::::::::::::::::::::::::::::::::::::::::::::::::::::::::::::::::::::::::::::::::::::::::::::::::::::::::::::::::::::::::::::::::::::::::::::::::::::::::::::::::::::::::::::::

@D0950.29_114636

GATGAACGCTAGCGACAGGCCTAACACATGCAAGTCGAGGGGCAACGGGAGTGTAGCAATACACTTGCCGGCGACCGGCGCACGGGTGAGTAACACGTATGCGACCTACCCATAGCAGGGGGATAATCGGAAGAAATTCCGTCTAATACCGCGTAATAATTCAGATCTGCATGGATTTGAATTTAAAGGAGCAATCCGGCTATGGATGGGCATGCGGGACATTAGCTAGTTGGCGGGGTAACGGCCCACCAAGGCTTCGATGTCTAGGGGTTCTGAGAGGAAGGTCCCCCACACTGGTACTGAGACACGGACCAGACTCCTACGGGAGGCAGCAGTGAGGAATATTGGTCAATGGTCGAGAGACTGAACCAGCCAAGTCGCGTGAGGGATGAAGGTTCTATGGATTGTAAACCTCTTTTGTCAGGGAGCAACGACATCCACGAGTGGGTGAATGAGAGTACCTGAAGAAAAGCATCGGCTAACTCCGTGCCAGCAGCCGCGGTAA

+

:::::::::::::::::::::::::::::::::::::::::::::::::::::::::::::::::::::::::::::::::::::::::::::::::::::::::::::::::::::::::::::::::::::::::::::::::::::::::::::::::::::::::::::::::::::::::::::::::::::::::::::::::::::::::::::::::::::::::::::::::::::::::::::::::::::::::::::::::::::::::::::::::::::::::::::::::::::::::::::::::::::::::::::::::::::::::::::::::::::::::::::::::::::::::::::::::::::::::::::::::::::::::::::::::::::::::::::::::::::::::::::::::::::::::::::::::::::::::::::::::::::::::::::::::::::::::

@D0950.29_114643

GATGAACGCTGGCGGCGTGCTTAACACATGCAAGTCGAACGAAGCAACTATCTTGCTTGCAAGAGAGTTGACTTAGTGGCGGACGGGTGAGTAACGTGTGGGTAACCTGCCTCATACAGGGGGATAACAGTTGGAAACGACTGCTAAGACCGCATAACCCGCTAGTGTCGCATGACACGGACGGAAAATATTTTATAGGTATGAGATGGGCCCGCGTCTGATTAGCTAGTTGGTAAGGTAACGGCTTACCAAGGCGACGATCAGTAGCCGACTTGAGAGAGTGATCGGCCACATTGGGACTGAGACACGGCCCAAACTCCTACGGGAGGCAGCAGTGGGGAATATTGGACAATGGGGGAAACCCTGATCCAGCGACGCCGCGTGAGTGAAGAAGTATTTCGGTATGTAAAGCTCTATCAGCAGGGAAGATAATGACAGTACCTGACTAAGAAGCCCCCGGCTAACTACGTGCCAGCAGCCG

+

:::::::::::::::::::::::::::::::::::::::::::::::::::::::::::::::::::::::::::::::::::::::::::::::::::::::::::::::::::::::::::::::::::::::::::::::::::::::::::::::::::::::::::::::::::::::::::::::::::::::::::::::::::::::::::::::::::::::::::::::::::::::::::::::::::::::::::::::::::::::::::::::::::::::::::::::::::::::::::::::::::::::::::::::::::::::::::::::::::::::::::::::::::::::::::::::::::::::::::::::::::::::::::::::::::::::::::::::::::::::::::::::::::::::::::::::::::::::::::::::::

@D0950.29_114658

GATGAACGCTAGCGACAGGCCTAACACATGCAAGTCGAGGGGTAGCACAAGGTAGCAATACTGAGGTGACGACCGGCGCACGGGTGAGTAACGCGTATGCAACCTACCTGTAAGAGTGGGATAGCCTCTCGAAAGAGAGATTAATACCGCATAATACCATTTCACTGCATGGTGAGATGGTTAAAGATTTATTGCTTACAGATGGGCATGCGTAACATTAGCTAGTTGGTGAGGTAACGGCTCACCAAGGCAACGATGTTTAGGGGTTCTGAGAGGAAGGTCCCCCACACTGGTACTGAGACACGGACCAGACTCCTACGGGAGGCAGCAGTGAGGAATATTGCACAATGGGCGAAAGCCTGATGCAGCGACGCCGCGTGAAGGAAGAAGGTCTTCGGATTGTAAACTTCTATCAGCAGGGAAGAATAAATGACGGTACCTGACTAAGAAGCTCCGGCTAACTACGTGCCAGCAGCCGCGGTAA

+

::::::::::::::::::::::::::::::::::::::::::::::::::::::::::::::::::::::::::::::::::::::::::::::::::::::::::::::::::::::::::::::::::::::::::::::::::::::::::::::::::::::::::::::::::::::::::::::::::::::::::::::::::::::::::::::::::::::::::::::::::::::::::::::::::::::::::::::::::::::::::::::::::::::::::::::::::::::::::::::::::::::::::::::::::::::::::::::::::::::::::::::::::::::::::::::::::::::::::::::::::::::::::::::::::::::::::::::::::::::::::::::::::::::::::::::::::::::::::::::::::::

@D0950.29_114731

GATGAACGCTAGCGACAGGCCTAACACATGCAAGTCGAGGGGTAGCACAAGGTAGTAATACTGAGGTGACGACCGGCGCACGGGTGAGTAACGCGTATGCAACCTACCTGTAAGAGTGGGATAGCCTCTCGAAAGAGAGATTAATACCGCATAATACCATTTCACTGCATGGTGAGATGGTTAAAGATTTATTGCTTACAGATGGGCATGCGTAACATTAGCTAGTTGGTGAGGTAACGGCTCACCAAGGCAACGATGTTTAGGGGTTCTGAGAGGAAGGTCCCCCACACTGGTACTGAGACACGGACCAGACTCCTACGGGAGGCAGCAGTGAGGAATATTGGTCAATGGACGAGAGTCTGAACCAGCCAAGTCGCGTGAAGGATGAAGGTCTTATGGATTGTAAACTTCTTTTATACGGGAATAAAAATGCCACGTGTGGCATATTGCATGTACCGTATGAATAAGGATCGGCTAACTCCGTGCCAGCAG

+

::::::::::::::::::::::::::::::::::::::::::::::::::::::::::::::::::::::::::::::::::::::::::::::::::::::::::::::::::::::::::::::::::::::::::::::::::::::::::::::::::::::::::::::::::::::::::::::::::::::::::::::::::::::::::::::::::::::::::::::::::::::::::::::::::::::::::::::::::::::::::::::::::::::::::::::::::::::::::::::::::::::::::::::::::::::::::::::::::::::::::::::::::::::::::::::::::::::::::::::::::::::::::::::::::::::::::::::::::::::::::::::::::::::::::::::::::::::::::::::::::::::::::::

@D0950.29_114773

GATGAACGCTGGCGGCGTGCTTAACACATGCAAGTCGAACGAAGCAGCTTTCTTGCTTGCAAGAAAGCTGACTTAGTGGCGGACGGGTGAGTAACGCGTGGGTAACCTGCCTCATACAGGGGGATAACAGTTGGAAACGACTGCTAAGACCGCATAACCCGCTAGTGTCGCATGACACGGACGGAAAAATATTTAATTTTGAACGAGTTAAAATTTGGTATGAGACTGGGCCCGCGTCTGATTAGCTAGTTGGTAAGGTAACGGCTTACCAAGGCGACGATCAGTAGCCGACTTGAGAGAGTGATCGGCCACATTGGGACTGAGACACGGCCCAAACTCCTACGGGAGGCAGCAGTGGGGAATATTGGACAATGGGGGAAACCCTGATCCAGCGACGCCGCGTGAGTGAAGAAGTATTTCGGTATGTAAAGCTCTATCAGCAGGGAAGATAATGACAGTACCTGACTAAGAAGCCCCCGGCTAA

+

::::::::::::::::::::::::::::::::::::::::::::::::::::::::::::::::::::::::::::::::::::::::::::::::::::::::::::::::::::::::::::::::::::::::::::::::::::::::::::::::::::::::::::::::::::::::::::::::::::::::::::::::::::::::::::::::::::::::::::::::::::::::::::::::::::::::::::::::::::::::::::::::::::::::::::::::::::::::::::::::::::::::::::::::::::::::::::::::::::::::::::::::::::::::::::::::::::::::::::::::::::::::::::::::::::::::::::::::::::::::::::::::::::::::::::::::::::::::::::::::::::

@D0950.29_114835

GATGAACGCTGGCGGCGTGCTTAACACATGCAAGTCGAACGAAGCAGCTTTCTTGCTTGCAAGAAAGCTGACTTAGTGGCGGACGGGTGAGTAACGCGTGGGTAACCTGCCTCATACAGGGGGATAACAGTTGGAAACGACTGCTAAGACCGCATAACCCGCTAGTGTCGCATGACACGGACGGAAAAATATTTTATAGGTATGAGATGGGCCCGCGTCTGATTAGCTAGTTGGTAAGGTAACGGCTTACCAAGGCGACGATCAGTAGCCGACTTGAGAGAGTGATCGGCCACATTGGGACTGAGACACGGCCCAAACTCCTACGGGAGGCAGCAGTGGGGAATATTGGACAATGGGGGAAACCCTGATCCAGCGACGCCGCGTGAGTGAAGAAGTATTTCGGTATGTAAAGCTCTATCAGCAGGGAAGATAATGACAGTACCTGACTAAGAAGCCCCCGGCTAACTACGTGCCAGCAGCCG

+

::::::::::::::::::::::::::::::::::::::::::::::::::::::::::::::::::::::::::::::::::::::::::::::::::::::::::::::::::::::::::::::::::::::::::::::::::::::::::::::::::::::::::::::::::::::::::::::::::::::::::::::::::::::::::::::::::::::::::::::::::::::::::::::::::::::::::::::::::::::::::::::::::::::::::::::::::::::::::::::::::::::::::::::::::::::::::::::::::::::::::::::::::::::::::::::::::::::::::::::::::::::::::::::::::::::::::::::::::::::::::::::::::::::::::::::::::::::::::::::::::

@D0950.29_114893

GATGAACGCTAGCTACAGGCTTAACACATGCAAGTCGAGGGGTAGCATGAAACTTAGCAATAAGTTTTGATGACGACCGGCGCACGGGTGAGTAACACGTATCCAACCTGCCTTTTACTCATGGATAGCCTTCTGAAAAGAAGATTAATACATGATGGTATTCAGAGTTTTCATGGACACTGAATTAAAGATTTTATCGGTAAGAGATGGGGATGCGTTCCATTAGATAGTAGGCGGGGTAACGGCCCACCTAGTCAACGATGGATAGGGGTTCTGAGAGGAAGGTCCCCACATTGGAACTGAGACACGGTCCAAACTCCTACGGGAGGCAGCAGTGAGGAATATTGGTCAATGGACGTAAGTCTGAACCAGCCAAGTAGCGTGAAGGATGAAGGCTCTATGGGTCGTAAACTTCTTTTATAAAAGGAATAAAGTATGCCACGTGTGGTGTTTTTGTATGTACTTTATGAATAAGGATCGGCTAACTCCGTGCCAGCAGCCGCGGT

+

::::::::::::::::::::::::::::::::::::::::::::::::::::::::::::::::::::::::::::::::::::::::::::::::::::::::::::::::::::::::::::::::::::::::::::::::::::::::::::::::::::::::::::::::::::::::::::::::::::::::::::::::::::::::::::::::::::::::::::::::::::::::::::::::::::::::::::::::::::::::::::::::::::::::::::::::::::::::::::::::::::::::::::::::::::::::::::::::::::::::::::::::::::::::::::::::::::::::::::::::::::::::::::::::::::::::::::::::::::::::::::::::::::::::::::::::::::::::::::::::::::::::::::::::::::::::::

@D0950.29_114950
[truncated: 51,247,952 more chars]
